# Selectivity control in hydrogenation through adaptive catalysis using ruthenium nanoparticles on a CO_2_-responsive support

**DOI:** 10.1038/s41557-021-00735-w

**Published:** 2021-07-05

**Authors:** Alexis Bordet, Sami El Sayed, Matthew Sanger, Kyle J. Boniface, Deepti Kalsi, Kylie L. Luska, Philip G. Jessop, Walter Leitner

**Affiliations:** 1 Max Planck Institute for Chemical Energy Conversion, Mülheim an der Ruhr, Germany; 2 Institut für Technische und Makromolekulare Chemie, RWTH Aachen University, Aachen, Germany; 3 Department of Chemistry, Queen’s University, Kingston, Ontario Canada

**Keywords:** Green chemistry, Catalysis

## Abstract

With the advent of renewable carbon resources, multifunctional catalysts are becoming essential to hydrogenate selectively biomass-derived substrates and intermediates. However, the development of adaptive catalytic systems, that is, with reversibly adjustable reactivity, able to cope with the intermittence of renewable resources remains a challenge. Here, we report the preparation of a catalytic system designed to respond adaptively to feed gas composition in hydrogenation reactions. Ruthenium nanoparticles immobilized on amine-functionalized polymer-grafted silica act as active and stable catalysts for the hydrogenation of biomass-derived furfural acetone and related substrates. Hydrogenation of the carbonyl group is selectively switched on or off if pure H_2_ or a H_2_/CO_2_ mixture is used, respectively. The formation of alkylammonium formate species by the catalytic reaction of CO_2_ and H_2_ at the amine-functionalized support has been identified as the most likely molecular trigger for the selectivity switch. As this reaction is fully reversible, the catalyst performance responds almost in real time to the feed gas composition.

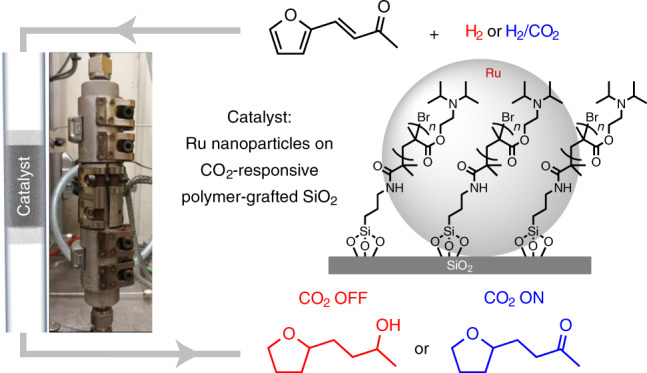

## Abstract (web summary)

A multifunctional catalytic system composed of ruthenium nanoparticles immobilized on a silica surface decorated with an amine-functionalized polymer is used for the hydrogenation of biomass-derived furfural acetone and related substrates. The presence or absence of CO_2_ in the gas feed alters the selectivity of the hydrogenation—producing either a ketone or a saturated alcohol, respectively—in a fully reversible manner.

## Main

**Fig. 1:**
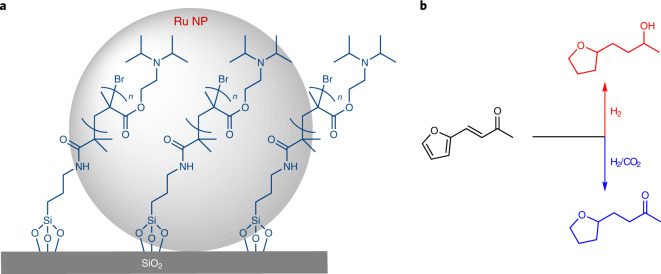
General strategy for the CO_2_-switchable hydrogenation of furfural acetone. **a**, Structure of the Ru@PGS catalyst composed of Ru NPs immobilized on silica modified with an amine-functionalized polymer. **b**, Model catalytic reaction: the hydrogenation of biomass-derived furfural acetone in the absence or presence of CO_2_ in the gas feed.

With the transition from fossil resources to a chemical value chain based on renewable energy and carbon resources, the development of new catalytic technologies to cope with the diversity and variation of feedstock quality is of crucial importance^1–8^. Extensive efforts are currently dedicated to the development of multifunctional catalytic systems able to achieve selective hydrogenation reactions of biomass-derived substrates and intermediates^9–21^. Although many of these catalysts present outstanding properties regarding their dedicated tasks, their performance is typically optimized for static operation under precisely defined parameters. Regional and temporary variations associated with renewable feedstock and energy supply are expected to require a larger degree of process flexibility, however^22,23^. The design and development of catalytic systems whose reactivity can be reversibly adjusted or may even respond adaptively to changes in feedstock composition provide a promising, yet difficult-to-achieve strategy in this context^24^. Known methods to ‘switch’ the reactivity of catalysts typically apply external stimuli, including temperature^25,26^, pH (ref. ^27^), solvent variation^24,28^, irradiation^24,28^ or redox processes^29–31^. Although these methods are able to generate two different states of a catalyst that exhibit different selectivities, the underlying physical and chemical elementary processes are mostly irreversible or associated with the generation of additional components that accumulate in the reaction mixture. We present here the molecular design of a catalyst that responds with a fully reversible selectivity switch to the presence of CO_2_ in the feed gas of a hydrogenation reaction (Fig. 1).

## Results and discussion

### General strategy

To enable this adaptivity, a novel bifunctional catalyst was prepared composed of ruthenium nanoparticles (Ru NPs) immobilized on tertiary amine-functionalized polymer-grafted silica (PGS). The design combined our previous experience of the preparation of Ru NPs for hydrogenation reactions^11,13,15,17^ with the use of PGS as a CO_2_-responsive material^32,33^. Furfural acetone (1), a biomass-derived platform chemical^34,35^, and similar compounds containing distinct reducible groups were chosen as substrates for hydrogenation in this case study (Fig. 1).

### Synthesis and characterization

The amine-functionalized PGS was prepared by molecular modification of commercial amorphous SiO_2_ particles (Brunauer–Emmett–Teller (BET) surface area = 285 m^2^ g^−1^) following a three-step synthetic method previously reported by us involving silanization and surface-initiated atom transfer radical polymerization^32,33^. The synthesis of Ru NPs immobilized on PGS was accomplished using our organometallic approach as a bottom-up synthesis method known to produce small and well-defined Ru NPs on functional supports^17,20^. In brief, metal loading was achieved by wet impregnation of PGS with a solution of [Ru(2-methylallyl)_2_(cod)] (cod, cycloocta-1,5-diene) in dichloromethane. After removal of the solvent in vacuo, the dried powder was subjected to an atmosphere of H_2_ (25 bar) at 100 °C for 18 h, giving the desired material, denoted as Ru@PGS (Fig. 1; for synthetic details, see Methods and the Synthesis of Ru@PGS section in the Supplementary Information).

**Fig. 2:**
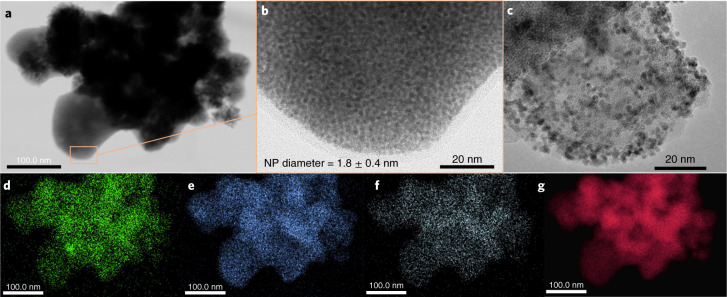
Characterization of the Ru@PGS catalyst by electron microscopy. **a**–**c**, TEM images of Ru@PGS. The image in **b** is a magnification of the region highlighted by the rectangle in **a**, and **c** shows a magnified image of a different zone of the material. **d**–**g**, HAADF-STEM-EDX elemental mapping images of Ru@PGS: Si (**d**), N (**e**), Br (**f**) and Ru (**g**). The structure of the catalyst is shown in Fig. 1a. These images show that the Ru@PGS material contains small Ru NPs (diameter = 1.8 ± 0.4 nm) that are well-dispersed over the support. In addition, the grafted polymer covers the silica support homogeneously.

Characterization of Ru@PGS by transmission electron microscopy (TEM; Fig. 2a,b) confirmed the formation of small and well-dispersed Ru NPs (diameter = 1.8 ± 0.4 nm). In addition, elemental mapping using high-angle annular dark-field scanning transmission electron microscopy with energy-dispersive X-ray spectroscopy (HAADF-STEM-EDX; Fig. 2c–g) showed that both the polymer (N and Br mapping, Fig. 2e,f) and the Ru NPs (Ru mapping, Fig. 2g) are homogeneously dispersed on the support (Si mapping, Fig. 2d), indicating that they are in close proximity. Rapid decomposition of the organometallic precursor associated with fast nucleation^36^ and stabilization by spatial confinement on the SiO_2_ support through steric interactions with the polymer chains^37^ are presumably important factors for the well-defined particle formation. A Ru loading of 0.82 mmol g^−1^ on the PGS (8.2 wt%) was determined using scanning electron microscopy with energy dispersive X-ray spectroscopy (SEM-EDX), consistent with the theoretical value (7.5 wt%). The value obtained from inductively coupled plasma with atom emission spectroscopy (ICP-AES) was a little lower (5.4 wt%), presumably due to the difficulty of fully dissolving the Ru at this relatively high metal loading. The BET surface area of the silica decreased upon polymer grafting from 285 to 55 m^2^ g^−1^, and then increased slightly to 84 m^2^ g^−1^ upon immobilization of the Ru NPs on the support (Supplementary Table 1), consistent with previous reports^38^.

Solid-state ^29^Si NMR analysis of the PGS and Ru@PGS showed the presence of tetra- and trifunctionalized Si centres on the polymer-bound SiO_2_ surface (Supplementary Fig. 1a,b). The solid-state ^13^C NMR spectra of the PGS and Ru@PGS revealed no major differences after the immobilization of the Ru NPs on the support, indicating that the polymer structure is stable under the conditions used for NP synthesis (Supplementary Fig. 1c,d). This is further supported by the fact that the nitrogen content remained unchanged upon Ru NP generation (Supplementary Table 1). In addition, neither [Ru(2-methylallyl)_2_(cod)] nor Ru@PGS showed any activity toward the hydrogenolysis of amide or ester groups in model substrates under these conditions, confirming their inability to cleave the polymer structure (Supplementary Table 2). The amount of accessible amine in the PGS and Ru@PGS was determined to be 1.28 and 1.14 mmol g^−1^, respectively. Thermogravimetric analysis performed under argon showed that the Ru@PGS material starts to lose mass and decomposes at around 200 °C (Supplementary Fig. 2). The material was applied successfully in catalysis at temperatures up to 150 °C, and no signs of decomposition or deactivation were observed at 100 °C for a time on stream of 12 h under continuous-flow conditions (vide infra).

### Catalytic study

**Fig. 3:**
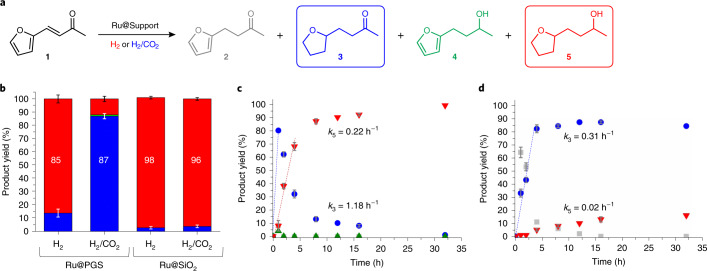
Hydrogenation of furfural acetone (1) under batch conditions. **a**, Reaction sequence for the hydrogenation of furfural acetone (1). **b**, Product distribution after hydrogenation of furfural acetone (1) under H_2_ or H_2_/CO_2_ using Ru@PGS or Ru@SiO_2_. Blue represents product 3 and red represents product 5. **c**,**d**, Reaction time profiles for the hydrogenation of furfural acetone under H_2_ (**c**) and H_2_/CO_2_ (**d**) using Ru@PGS as catalyst. Grey squares, product 2; blue circles, product 3; green triangles, product 4; red triangles, product 5. Reaction conditions: Ru catalyst (0.026 mmol), substrate (0.65 mmol, 25 equiv.), butan-1-ol (0.5 ml), H_2_ (15 bar), H_2_/CO_2_ (30 bar, 1:1 ratio), 80 °C, 16 h. The conversion was >99%. The compositions of the reaction mixtures were determined by gas chromatography with flame ionization detection (GC-FID) using tetradecane as internal standard. Experiments were repeated two to four times. Mean values are given and the error bars represent standard deviations. These results show a selectivity switch with the Ru@PGS catalyst when the gas phase composition is changed from pure H_2_ to H_2_/CO_2_, with the activity towards C=O hydrogenation becoming very low. In contrast, no influence of the presence of CO_2_ was observed when using Ru@SiO_2_ as catalyst. Source data

The hydrogenation of furfural acetone (1) has been the subject of various studies towards the production of fuel components or chemicals from biomass^3,39,40^. The reaction process starts with C=C bond hydrogenation to form 4-(2-furyl)butan-2-one (2). Next, hydrogenation of the furan ring leads to 4-(tetrahydro-2-furyl)butan-2-one (3), followed by reduction of the C=O group to produce 4-(tetrahydro-2-furyl)butan-2-ol (5; Fig. 3a). The alternative sequence, with hydrogenation of the ketone in 2 to produce 4-(2-furyl)butan-2-ol (4) prior to the reduction of the furan ring, is less preferred over ruthenium catalysts. The presence of several distinct reducible groups thus provides a valuable chemical probe to evaluate the variations in activity and selectivity of hydrogenation catalysts. The catalytic reactions were performed under batch conditions using stainless-steel high-pressure reactors with magnetic stir bars. After parameter screening (Supplementary Table 3), the standard reaction conditions were defined as Ru@PGS (7.5 wt%) with 4 mol% Ru per substrate (1 mol% per reducible group) in butan-1-ol as solvent at *T* = 80 °C for *t* = 16 h. As feed gas, either pure H_2_ (15 bar) or a mixture of H_2_ and CO_2_ (30 bar total pressure, 1:1 ratio) was used to assess the potential influence of CO_2_ as a molecular trigger. Using Ru@PGS as catalyst under pure H_2_, furfural acetone (1) was hydrogenated to the saturated alcohol 5 in high yield (85%), showing a reactivity typical of Ru NP catalysts^13^.

Varying the total pressure of pure H_2_ from 60 to 15 bar did not influence substantially the product distribution (Supplementary Table 3, entries 7 and 13). Applying a mixture of H_2_ and CO_2_ changed the selectivity drastically, however, leading to the production of the saturated ketone 3 in high yield (87%; Fig. 3b). In sharp contrast, the use of Ru NPs immobilized on non-functionalized SiO_2_ as reference (Ru@SiO_2_) resulted in the exclusive formation of 5 (>96% yield) irrespective of the composition of the gas phase. See the ‘Synthesis of Ru@SiO_2_’ section in the Supplementary Information for synthetic details. Increasing the amount of substrate to 100 equivalents led to the observation of butan-2-ones 2 and 3, and confirmed the inability of CO_2_ to influence the activity and selectivity of Ru@SiO_2_ (Supplementary Table 4). For both catalysts under standard conditions, mixing H_2_ with Ar gave similar results as under pure H_2_, demonstrating that the simple dilution of H_2_ with an inert gas is not sufficient to initiate the selectivity switch (Supplementary Table 5).

The striking effect of the presence of CO_2_ on the performance of the Ru@PGS catalyst is evidenced in the product yield–time profiles of the hydrogenation of 1 recorded under H_2_ or H_2_/CO_2_ atmosphere (Fig. 3c,d and Supplementary Table 6 for the complete data set). With pure H_2_, the hydrogenation of the C=C double bond and the aromatic ring was fast (initial rate constant for the formation of 3, *k*_3_ = 1.18 h^−1^), followed by the hydrogenation of the C=O group with an apparent rate constant of *k*_5_ = 0.22 h^−1^. Thus, the fully hydrogenated product 5 was formed in high yield (89%) already after 8 h. In the presence of H_2_/CO_2_ the double bond and the furan ring were hydrogenated at somewhat lower rates (*k*_3_ = 0.31 h^−1^), producing 3 in high yield (84%) after 8 h. Most importantly, C=O hydrogenation was shut down almost completely (*k*_5_ = 0.02 h^−1^), leading to less than 20% of 5 even after 35 h reaction time. Between 5 and 20 h of reaction, the ratio of 3 to 5 remained nearly constant at 85:15.

This excellent switch in selectivity, suppressing C=O hydrogenation through the addition of CO_2_ to the hydrogen feed gas, was explored under batch conditions using other ketone-containing furan derivatives as substrates. Satisfyingly, the hydrogenation selectivity could be controlled using CO_2_ as the molecular trigger for these substrates as well, leading to the selective production of either saturated alcohols under H_2_ or saturated ketones under H_2_/CO_2_ under otherwise identical conditions (Table 1).

**Table 1:**
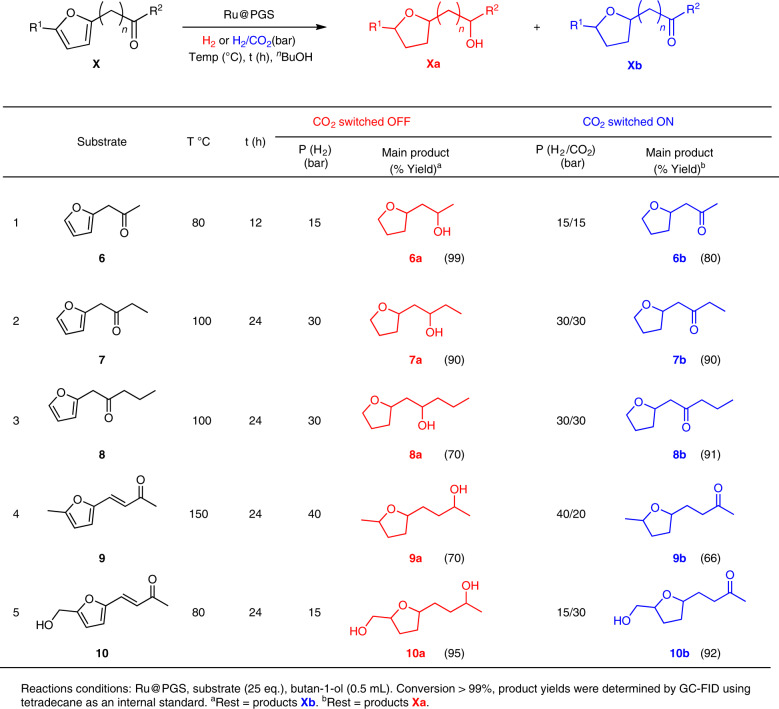
Hydrogenation of ketone-containing furan derivatives using Ru@PGS as catalyst under H_2_ or H_2_/CO_2_

**Fig. 4:**
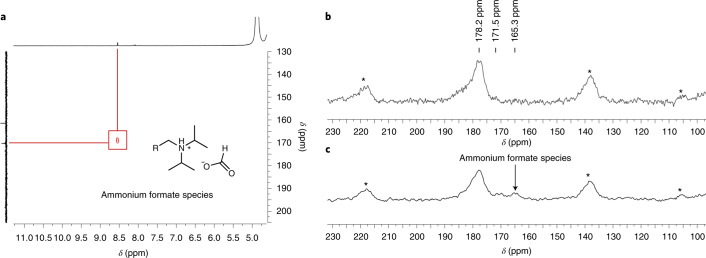
NMR characterization of the Ru@PGS catalyst. **a**, 2D HSQC NMR (400 MHz, MeOD) spectrum of the Ru@PGS catalyst after reaction with H_2_/CO_2_. **b**, Solid-state ^13^C CP-MAS NMR (125.7 MHz) spectrum before reaction with H_2_/CO_2_. **c**, Solid-state ^13^C CP-MAS NMR (125.7 MHz) spectrum after reaction with H_2_/CO_2_, showing the presence of ammonium formate species. The asterisks (*) indicate spinning side bands. These spectra evidence the formation of ammonium formate species on the catalyst when hydrogenation reactions are performed using H_2_/CO_2_.

To investigate molecular changes on the surface of the Ru@PGS catalyst in the presence of carbon dioxide as possible reasons for the drastic reactivity switch, the catalyst was treated with H_2_/CO_2_ (30 bar, 1:1) under the standard conditions (80 °C, 16 h) but in absence of any substrate. Deuterated methanol was used as the solvent to allow acquisition of the ^1^H and ^13^C{^1^H} NMR spectra of the reaction mixture containing the material in suspension. A ^13^C signal at 161.5 ppm (Supplementary Fig. 3) indicated the presence of an ammonium bicarbonate species, as expected for PGS materials^41,42^. In addition, however, the ^1^H and ^13^C NMR spectra showed the appearance of strong signals at 8.5 and 169.5 ppm, respectively, which are characteristic of ammonium formate species (Supplementary Figs. 3 and 4)^43,44^. The two-dimensional (2D) heteronuclear single quantum coherence (HSQC) NMR spectrum confirmed the correlation of the two signals (Fig. 4a). Substituting H_2_ for D_2_ resulted in a 1:1:1 triplet splitting of the ^13^C signal at 169.5 ppm, proving that the ammonium formate species is indeed formed by the hydrogenation of CO_2_ (Supplementary Fig. 5). Solid-state ^13^C cross polarization-magic angle spinning (CP-MAS) NMR analysis of the Ru@PGS material before and after reaction with H_2_/CO_2_ revealed a new signal at 165.3 ppm after the reaction, which confirmed the presence of an ammonium formate species also on the spent catalyst (Fig. 4b,c). These data demonstrate that the Ru@PGS catalyst is active in the hydrogenation of CO_2_ to formic acid, which is stabilized as ammonium formate species on the amine-decorated support.

The possible role of formate species in the observed selectivity switch was evaluated through reference experiments using diisopropyl(ethyl)ammonium formate (DIPEF) as a molecular model for the surface modification (Supplementary Table 7). Adding DIPEF to the catalytic reaction conducted with Ru@PGS as catalyst but with pure H_2_ showed a similar suppression of the hydrogenation activity as with H_2_/CO_2_. Most importantly, the addition of DIPEF also steered the hydrogenation of 1 over Ru@SiO_2_ selectively towards the ketone 3 (71%, with only only 9% of 5), whereas CO_2_ alone had no effect in this case. These data clearly demonstrate that ammonium formate species such as those formed under the H_2_/CO_2_ atmosphere on the catalyst surface are able to alter the hydrogenation performance of supported ruthenium particles. Although an additional contribution from the observed bicarbonate species cannot be excluded, we conclude that CO_2_ hydrogenation to formate on the catalyst surface plays a key role in the selectivity switch observed under H_2_/CO_2_. One possible hypothesis for this behaviour is that the ammonium formate interacts with the surface of the Ru NPs, preferentially blocking the active sites for the hydrogenation of the more polar C=O group rather than the C=C hydrogenation sites.

**Fig. 5:**
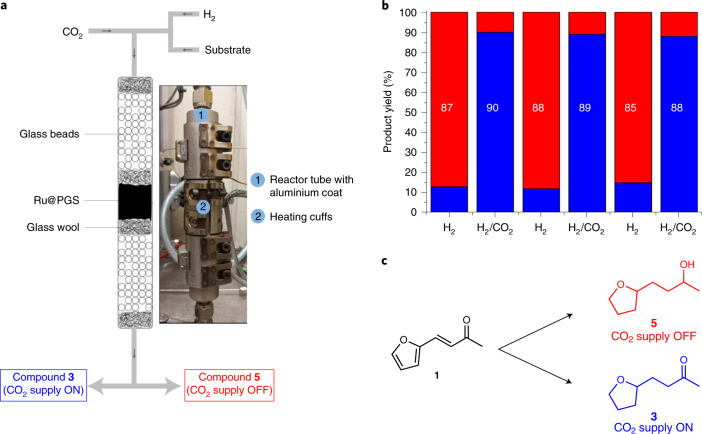
Switchability tests under continuous-flow conditions. **a**, Schematic representation and photograph of the continuous-flow set-up. **b**, Hydrogenation of furfural acetone (1) using Ru@PGS as catalyst with H_2_ and then with H_2_/CO_2_ (red, product 5; blue, product 3). **c**, Selectivity switch controlled by the feed gas composition. Reaction conditions: Ru@PGS (2.0 g, 1.08 mmol), substrate (0.025 M in butan-1-ol, flow rate = 0.5 ml min^−1^, residence time = 6.4 min), 20 bar H_2_ or 40 bar H_2_/CO_2_ (1:1; gas flow rate = 35 ml min^−1^), 100 °C. The conversion was >99%. The compositions of the reaction mixtures were determined by GC-FID using tetradecane as an internal standard. These results demonstrate that the selectivity switch is fully reversible, and that switching back and forth between the gas compositions allows alternating production of the saturated alcohol or ketone in high yields. Source data

As the hydrogenation of CO_2_ to formic acid is known to be an equilibrium reaction^45–48^, we evaluated the reversibility of the formate-induced selectivity switch between the formation of 5 versus 3 under batch conditions using the Ru@PGS catalyst in alternating cycles of H_2_ and H_2_/CO_2_ as feed gas. Under the standard conditions, the reactions were found to produce consistently high yields of 5 (73–85%) under H_2_ or 3 (84–89%) under H_2_/CO_2_ in six consecutive runs (Supplementary Fig. 6). Based on these promising results, we implemented the Ru@PGS material in a fully adaptive catalytic system for the hydrogenation of furfural acetone (1). The experiments involved passing a solution of 1 (0.025 M in butan-1-ol) over a packed bed of Ru@PGS (2.0 g) in an in-house built continuous-flow reactor (Fig. 5a and Supplementary Figs. 7 and 8). After screening of the process parameters, the reaction conditions were fixed at 100 °C with a substrate flow of 0.5 ml min^−1^ (residence time = 6.4 min) and a gas flow (H_2_ or H_2_/CO_2_) of 35 ml min^−1^ (Supplementary Table 8). The hydrogenation of furfural acetone (1) was performed for 2 h with pure H_2_ (20 bar) before switching to H_2_/CO_2_ (40 bar, 1:1) as feed gas for the next 2 h and then back again. Only a short time without substrate delivery was applied between each switch to allow for adjustment of the formate equilibrium (see Methods and the Supplementary Information for details). The feed gas was exchanged five times to evaluate the ability of the catalyst to adapt repeatedly in real time to produce the two different products 3 and 5 under continuous-flow conditions (Fig. 5b).

Under the standard conditions (100 °C, H_2_ (20 bar) or H_2_/CO_2_ (40 bar, 1:1), substrate flow = 0.5 ml min^−1^ (residence time = 6.4 min), 2 h), the reaction changed in a fully reversibly manner to the composition of the feed gas, enabling the system to switch back and forth to produce alternately 5 and 3, respectively, in high yields (85–90%; Fig. 5b). The selectivity towards the formation of products 3 and 5 could be further improved by optimizing the reaction conditions individually for each production mode (H_2_ or H_2_/CO_2_), reaching excellent product yields of 99 and 93% for 3 and 5, respectively (Supplementary Table 8). TEM characterization of the Ru@PGS material after 12 h on stream did not show notable change in the size and dispersion of the Ru NPs (1.9 ± 0.3 nm; Supplementary Fig. 9). In addition, elemental mapping by HAADF-STEM-EDX evidenced that the polymer and the Ru NPs were still homogeneously distributed over the support (Supplementary Fig. 9e–h). Elemental analysis (SEM-EDX and inductively coupled plasma, ICP) showed that no substantial ruthenium leaching occurred. ICP and BET analysis evidenced a reduced nitrogen content (~30%) and increased surface area (177 m^2^ g^−1^), indicating some leaching from the PGS material (Supplementary Table 9). This may be attributed to the loss of non-covalently attached polymer in the early phase of the process, because the performance of the catalyst remained stable throughout the experiment.

## Conclusion

In summary, we have developed a catalytic system that allows a switch in the product selectivity of a hydrogenation reaction with the absence or presence of CO_2_ in the feed gas. The design of the catalyst exploited a molecular approach to surface modification and metal nanoparticle formation, depositing Ru NPs by an organometallic approach on amine-functionalized PGS. The key elements are the CO_2_-responsiveness of the surface-attached tertiary amine-functionalized polymer together with the catalytic activity of ruthenium nanoparticles. With the resulting material, the formation of products from the hydrogenation of furfural acetone and other ketone-containing furan derivatives could be controlled to yield selectively either the saturated alcohol or ketone with pure H_2_ or H_2_/CO_2_, respectively. As the selectivity switch is induced by the reversible hydrogenation of CO_2_, there is no accumulation of any additional material on the catalyst or in the product stream, allowing waste-free control of the reaction outcome simply by turning on or off the CO_2_ supply. In the general context of flexible plant design, this offers potential for two different operating modes without any changes to the catalyst or the reactor unit. The built-in responsiveness would allow the system even to adapt to different feed gas compositions, for example, pure H_2_ from water electrolysis or H_2_/CO_2_ from biomass reforming.

The formation of alkylammonium formate species at the amine-functionalized support in the presence of CO_2_ is believed to play a role in promoting the formation of the ketone over the saturated alcohol, possibly by preferentially blocking the active sites for the hydrogenation of the more polar C=O group rather than the C=C hydrogenation sites. However, the design principle of a CO_2_-responsive surface as support for catalytically active metal nanoparticles or complexes is more generally applicable. We hope that extension of the concept to other metals and other reversible functional groups will open many new opportunities for the development of adaptive catalytic systems to enable flexible production schemes on the basis of renewable feedstock and energy supply.

## Methods

### Safety warning

High-pressure experiments with compressed H_2_ must be carried out only with the appropriate equipment and under rigorous safety precautions.

### General

Unless otherwise stated, the Ru NPs were immobilized on the PGS material (Ru@PGS) under an inert atmosphere (argon) using standard Schlenk techniques or in a glove box. Furfural acetone (1) was purified by sublimation prior to use (white crystals). [Ru(2-methylallyl)_2_(cod)] was obtained from Umicore. Synthetic air (20.5 vol% O_2_, the rest N_2_, no hydrocarbon) was purchased from Westfalen. Catalyst solutions and substrates were prepared in air, but were flushed with H_2_ and/or CO_2_ prior to performing catalytic reactions. All other chemicals and solvents were purchased from commercial sources and used without purification.

### Synthesis of the Ru@PGS catalyst

[Ru(2-methylallyl)_2_(cod)] (128 mg, 0.401 mmol) was dissolved in DCM (10 ml) and added to a suspension of PGS (500 mg) in DCM (10 ml). The reaction mixture was stirred at room temperature for 1 h. After solvent removal at room temperature and drying the impregnated PGS in vacuo for 1 h, the powder was loaded into a 20-ml high-pressure autoclave and subjected to an atmosphere of H_2_ (25 bar) at 100 °C for 18 h. Under this reducing environment, the impregnated PGS transformed from light orange to black, indicating the immobilization of the Ru NPs on the PGS.

### Hydrogenation of furfural acetone (1) with H_2_

The Ru catalyst (35 mg, 0.026 mmol Ru) and butan-1-ol (0.5 ml) were combined with 1 (90 mg, 0.65 mmol, 25 equiv.) in a glass insert and placed in a 10-ml high-pressure autoclave. After purging the autoclave with H_2_, the reaction mixture was stirred at 80 °C in an aluminium heating block under 15 bar H_2._ Once the reaction had finished, the reactor was cooled in an ice bath and carefully vented. After filtration, a sample of the reaction mixture was taken and analysed by GC-FID using tetradecane as internal standard.

### Hydrogenation of furfural acetone (1) with CO_2_/H_2_

The Ru catalyst (35 mg, 0.026 mmol Ru) and butan-1-ol (0.5 ml) were combined with 1 (90 mg, 0.65 mmol, 25 equiv.) in a glass insert and placed in a 10-ml high-pressure autoclave. After purging with CO_2_ and stirring for 2 min, the autoclave was further pressurized first with 15 bar CO_2_ and then with enough H_2_ to raise the total pressure to 30 bar (CO_2_/H_2_ ratio ~1:1). The reaction mixture was stirred at 80 °C in an aluminium heating block under the desired pressure of H_2_ and CO_2_. Once the reaction had finished, the reactor was cooled in an ice bath and carefully vented. After filtration, a sample of the reaction mixture was taken and analysed by GC-FID using tetradecane as internal standard.

### Hydrogenation of furan derivatives 6–10 with H_2_ or CO_2_/H_2_

Ru@PGS (35 mg, 0.026 mmol Ru) and butan-1-ol (0.5 ml) were combined with the substrate (0.65 mmol, 25 equiv.) in a glass insert and placed in a 10-ml high-pressure autoclave. After purging, the autoclave was pressurized with H_2_ (and CO_2_, if applicable) to raise the total pressure to the desired value. The reaction mixture was stirred at the desired temperature in an aluminium heating block. Once the reaction had finished, the reactor was cooled in an ice bath and carefully vented. After filtration, a sample of the reaction mixture was taken and analysed by GC-FID using tetradecane as internal standard.

### Switchability experiments under batch conditions: hydrogenation of furfural acetone (1)

The Ru catalyst (35 mg, 0.026 mmol Ru) and butan-1-ol (0.5 ml) were combined with 1 (90 mg, 0.65 mmol, 25 equiv.) in a glass insert and placed in a 10-ml high-pressure autoclave. After purging the autoclave with the respective gases, the reaction mixture was stirred at 80 °C in an aluminium heating block under the desired pressure of the gases used. Once the reaction had finished, the reactor was cooled in an ice bath and carefully vented. The mixture was centrifuged and a sample of the solution was taken and analysed by GC-FID using tetradecane as internal standard. For switchability, the reaction mixture was centrifuged, the supernatant removed and the residue washed with butan-1-ol (3 × 1 ml). The catalyst was then dried at 100 °C for 1 h. For the next cycle, a fresh portion of the substrate (90 mg, 0.65 mmol, 25 equiv.) and butan-1-ol (0.5 ml) were added and the reaction was performed again. This procedure was repeated for each catalytic cycle by alternately pressurizing the autoclave either with only H_2_ or with CO_2_ and H_2_.

### Hydrogenation of furfural acetone (1) in the presence of various additives

The Ru catalyst (35 mg, 0.026 mmol Ru), butan-1-ol (0.5 ml) and the additive (0.026 mmol, 1 equiv.) were combined with 1 (90 mg, 0.65 mmol, 25 equiv.) in a glass insert and placed in a 10-ml high-pressure autoclave. After purging the autoclave with H_2_, the reaction mixture was stirred at 80 °C in an aluminium heating block under 15 bar H_2_. Once the reaction had finished, the reactor was cooled in an ice bath and carefully vented. After filtration, a sample of the reaction mixture was taken and analysed by GC-FID using tetradecane as internal standard.

### Switchability experiments in continuous-flow conditions: hydrogenation of furfural acetone (1)

The reactor was loaded with the Ru@PGS catalyst (2.0 g, 1.486 mmol Ru, 3.2-ml reactor volume) and installed in the continuous-flow set-up. The system was pressurized with H_2_, heated to the desired temperature and the pump was loaded with the substrate solution. The substrate and gas flows were mixed in a volume flow mixer (alternately H_2_ and H_2_/CO_2_), the feed flow was passed through the heated reactor and samples were collected at the output. The compositions of the samples were determined by GC-FID using tetradecane as internal standard. To switch to H_2_/CO_2_, the CO_2_ flow was started (35 ml min^−1^), the total pressure at the back-pressure regulator was set to 40 bar and the catalyst was treated for 15 min. The substrate solution flow was then turned on and the first samples were collected after 15 min on stream. To switch to only H_2_, the H_2_ and CO_2_ flows were turned off, as well as the substrate solution flow. The catalyst was streamed with synthetic air (3 bar) for 1 h at 100 °C, until no solvent was observed at the output. The substrate and H_2_ flows were then started again, with the total pressure at the back-pressure regulator set to 20 bar. This procedure was repeated for each switch of atmosphere.

## Online content

Any methods, additional references, Nature Research reporting summaries, source data, extended data, supplementary information, acknowledgements, peer review information; details of author contributions and competing interests; and statements of data and code availability are available at 10.1038/s41557-021-00735-w.

### Supplementary information

Supplementary Information Detailed experimental section, Supplementary Figs. 1–9, Tables 1–9, additional characterization data (Supplementary Figs. 10–116, including GC chromatograms, NMR spectra).

Supplementary Data Source data for the graph in Supplementary Fig. 6.

## Data Availability

All of the data that support the findings of this study, including material characterizations and catalytic measurements, are available within the paper and its Supplementary Information files (Supplementary Figs. 1–117 and Supplementary Tables 1–9). Further requests about the data can be directed to the corresponding author. Source data are provided with this paper.

## 4-(2-furyl)but-3-en-2-one

PubChemID: 441325612
InChIKey: GBKGJMYPQZODMI-SNAWJCMRSA-N
MDL Molfile
Chemdraw file

## 4-(2-furyl)-butan-2-one

PubChemID: 441325613
InChIKey: GGJUJWSDTDBTLX-UHFFFAOYSA-N
MDL Molfile
Chemdraw file

## 4-(2-tetrahydrofuryl)-butan-2-one

PubChemID: 441325614
InChIKey: PMQRDJLOVZHSOC-UHFFFAOYSA-N
MDL Molfile
Chemdraw file

## 4-(2-furyl)-2-butanol

PubChemID: 441325615
InChIKey: WJKRHTCMSSXHNV-UHFFFAOYSA-N
MDL Molfile
Chemdraw file

## 4-(2-tetrahydrofuryl)-butan-2-ol

PubChemID: 441325616
InChIKey: JBLNBMHKAAHKDC-UHFFFAOYSA-N
MDL Molfile
Chemdraw file

## 1-(furan-2-yl)propan-2-one

PubChemID: 441325617
InChIKey: IQOJTGSBENZIOL-UHFFFAOYSA-N
MDL Molfile
Chemdraw file

## 1-(tetrahydrofuran-2-yl)propan-2-ol

PubChemID: 441325618
InChIKey: CAHGFMDHALWJAS-UHFFFAOYSA-N
MDL Molfile
Chemdraw file

## 1-(tetrahydrofuran-2-yl)propan-2-one

PubChemID: 441325619
InChIKey: LHYVBWMBMGJLLI-UHFFFAOYSA-N
MDL Molfile
Chemdraw file

## 1-(furan-2-yl)butan-2-one

PubChemID: 441325620
InChIKey: GLDGYLKTISAERO-UHFFFAOYSA-N
MDL Molfile
Chemdraw file

## 1-(tetrahydrofuran-2-yl)butan-2-ol

PubChemID: 441325621
InChIKey: RXKYGHVJRBTGFC-UHFFFAOYSA-N
MDL Molfile
Chemdraw file

## 1-(tetrahydrofuran-2-yl)butan-2-one

PubChemID: 441325622
InChIKey: MTMPGCVZHHGSQV-UHFFFAOYSA-N
MDL Molfile
Chemdraw file

## 1-(furan-2-yl)pentan-2-one

PubChemID: 441325623
InChIKey: NIVNFVHGZUNULS-UHFFFAOYSA-N
MDL Molfile
Chemdraw file

## 1-(tetrahydrofuran-2-yl)pentan-2-ol

PubChemID: 441325624
InChIKey: AUFHQHXLHVLBNP-UHFFFAOYSA-N
MDL Molfile
Chemdraw file

## 1-(tetrahydrofuran-2-yl)pentan-2-one

PubChemID: 441325625
InChIKey: XQHKVARVKPXDSN-UHFFFAOYSA-N
MDL Molfile
Chemdraw file

## (*E*)-4-(5-methylfuran-2-yl)but-3-en-2-one

PubChemID: 441325626
InChIKey: FKNBELIGEMXLJS-HWKANZROSA-N
MDL Molfile
Chemdraw file

## 4-(5-methyltetrahydrofuran-2-yl)butan-2-ol

PubChemID: 441325627
InChIKey: CLUBJFBDEKELGS-UHFFFAOYSA-N
MDL Molfile
Chemdraw file

## 4-(5-methyltetrahydrofuran-2-yl)butan-2-one

PubChemID: 441325628
InChIKey: BDEGNXINPSPUBJ-UHFFFAOYSA-N
MDL Molfile
Chemdraw file

## (*E*)-4-(5-(hydroxymethyl)furan-2-yl)but-3-en-2-one

PubChemID: 441325629
InChIKey: FCGIQKXMUQPTPR-NSCUHMNNSA-N
MDL Molfile
Chemdraw file

## 4-(5-(hydroxymethyl)tetrahydrofuran-2-yl)butan-2-ol

PubChemID: 441325630
InChIKey: CEXRRWLXQRCHAW-UHFFFAOYSA-N
MDL Molfile
Chemdraw file

## 4-(5-(hydroxymethyl)tetrahydrofuran-2-yl)butan-2-one

PubChemID: 441325631
InChIKey: JKVQHYRDFYEIGX-UHFFFAOYSA-N
MDL Molfile
Chemdraw file

## Source data

Source Data Fig. 3 Source data for the graphs in Fig. 3.
Source Data Fig. 5 Source data for the graph in Fig. 5.
