# Supplementary material for: Selectivity control in hydrogenation through adaptive catalysis using ruthenium nanoparticles on a CO2-responsive support
Source: Nat Chem. 2021 Jul 5;13(9):916–22. doi: 10.1038/s41557-021-00735-w (PMC8440215; doi:10.1038/s41557-021-00735-w)
Supplement: Supplementary file 1 — Detailed experimental section, Supplementary Figs. 1–9, Tables 1–9, additional characterization data (Supplementary Figs. 10–116, including GC chromatograms, NMR spectra). [file 41557_2021_735_MOESM41_ESM.pdf]

nature portfolio

https://doi.org/10.1038/s41557-021-00735-w

Supplementary information

Selectivity control in hydrogenation through adaptive catalysis using ruthenium nanoparticles on a CO_2-responsive support

In the format provided by the authors and unedited

Selectivity control in hydrogenation through adaptive catalysis using ruthenium nanoparticles on a CO_2-responsive support

Alexis Bordet,[1] Sami El Sayed,[2] Matthew Sanger,[3] Kyle J. Boniface,[3] Deepti Kalsi,[1] Kylie L. Luska,[2] Philip G. Jessop,[3] and Walter Leitner[1,2]

[1] Max Planck Institute for Chemical Energy Conversion, Stiftstrasse 34-36, 45470 Mülheim an der Ruhr, Germany

[2] Institut für Technische und Makromolekulare Chemie, RWTH Aachen University, Worringerweg 2, 52074 Aachen, Germany.

[3] Department of Chemistry, Queen's University, 90 Bader Lane, Kingston, ON, Canada

These authors contributed equally: Alexis Bordet, Sami El Sayed. Correspondence and requests for materials should be addressed to Walter Leitner. (email: walter.leitner@cec.mpg.de)

Experimental **3**
Safety warning 3
General 3
Analysis 3
Synthesis and characterization of Ru@PGS 4
Synthesis of the polymer-grafted silica (PGS)[1] 4
Synthesis of Ru@PGS 6
Synthesis of Ru@SiO_2 6
Supplementary tables and figures **7**
Characterization 7
Catalytic study under batch conditions 11
Mechanistic investigations 16
Switchability test in batch conditions 20
Continuous flow catalysis 20
Additional characterization data **25**
Polymer grafted silica (PGS) 25
Ru@SiO_2 29
GC Chromatograms 30
NMR Spectra 74
References **88**

Experimental

Safety warning

High-pressure experiments with compressed H_2 must be carried out only with appropriate equipment and under rigorous safety precautions.

General

If not otherwise stated, the immobilization of nanoparticles on the PGS-material (Ru@PGS) was performed out under an inert atmosphere using standard Schlenk techniques or in a glovebox as previously reported. Furfuralacetone (IUPAC name: 4-(2-Furyl)but-3-en-2-one) was purified by sublimation prior to use (white crystals). [Ru(2-methylallyl)$_2$(cod)] was obtained from Umicore. Synthetic air (20.5 Vol-% O_2, rest N_2, no hydrocarbon) was purchased from Westfalen AG. Catalyst solutions and substrate were prepared under air, but were flushed with H_2 and/or CO_2 prior to catalysis. All other chemicals and solvents were purchased from commercial sources and used without purification.

Analysis

^{1}H and ^{13}C NMR spectra were obtained using a Bruker Avance 400™ spectrometer at 400 MHz (^{1}H) and 100 MHz (^{13}C) at 25 °C. Solid-state ^{29}Si & ^{13}C CP-MAS NMR spectra were obtained using a Bruker AVIII-500 spectrometer. The GPC analysis was performed using THF as the eluent. Samples were prepared at 5.0 mg/mL and passed through a 0.2 µm PTFE filter prior to injection. The samples were analysed on a Waters 2695 separation module using a refractive index detector (Waters 410 differential refractometer) at 32 °C and 1 mL/min flow rate. The GPC was calibrated using polystyrene standards. Brunauer-Emmett-Teller (BET) measurements were performed on a Quadrasord SI automated Surface Area and Pore Size Analyser from Quantachrome Instruments and the data analysis using QuadraWin 5-04. Inductively coupled plasma (ICP) was performed at Mikroanalytisches Laboratorium Kolbe on a

Perkin Elmer Analyst 200 Atomic Absorption Spectrometer. High-pressure experiments were performed using in-house engineered 10 and 20 mL stainless steel finger autoclaves. Catalytic reactions were performed in glass inlets using a magnetic stirrer (800 rpm) and an aluminium heating block. Gas chromatography (GC) was performed on a Thermo Scientific Chromatograph Trace GC Ultra equipped with a CP-WAX column. The composition of the reaction mixture was identified by injecting the pure products in the GC. Electron microscopy images (TEM, STEM-HAADF-EDX) were collected using a Hitachi aberration-corrected scanning transmission electron microscope (HF-2000) operated at 200 kV. Thermogravimetric analysis was performed on a Netzsch STA 409 with a temperature program (1000°C, 5°C/min) under an argon flow of 100 mL/min.

Synthesis and characterization of Ru@PGS

Synthesis of the polymer-grafted silica (PGS)[1]

Amine functionalization of silica. 15 g of silica particles (SiO_2 #5, SiliaFlash P60, 40-63 μm, 285 $m^2.g^{-1}$, 60 Å pore size) were purified and hydroxylated in a 1 M HCl solution at 50 °C for 4 h and then calcined at 200 °C for 18 h. The hydroxyl-rich silica was transferred directly to a reaction vessel, evacuated and then replaced with argon atmosphere. Under inert conditions, 500 mL of anhydrous toluene was cannulated to the reaction flask and heated to 70 °C. Under vigorous stirring, 5 mL of the amine "APTES" ((3-aminopropyl)triethoxysilane) was added to the mixture via syringe and left to react at 70 °C for 18 h. The amine-functionalized silica was collected by vacuum filtration and washed several times with anhydrous ethanol and stored under argon until further use.

Synthesis of SI-ATRP initiator functionalized silica. The amine-functionalized silica particles were transferred to a dried round bottom flask equipped with an overhead stirrer, evacuated and then replaced with argon. Under inert conditions, 200 mL of anhydrous tetrahydrofuran (THF) was added along with diisopropylamine (1eq., 4.0 mmol) to the flask via cannula. The reaction mixture was cooled down in an ice bath for ca. 30 min followed by adding 2-bromo-2-methylpropionyl bromide (BIBB, 1 eq., 4.04 mmol) dropwise under vigorous stirring in the ice bath for an additional 30 min. After removing the ice bath, the mixture was left to react for 16 h at room temperature. The BIBB-functionalized silica was then collected by vacuum filtration and washed multiple times with THF and stored under argon until further use.

SI-AGET-ATRP of DiPAEMA. The BIBB-grafted silica particles were transferred to a round bottom flask equipped with an overhead stirrer followed by adding 2-(diisopropylamino)ethyl methacrylate (DiPAEMA, 235 eq., 401.0 mmol) passed over an inhibitor removal column [Sigma-Aldrich, removing hydroquinone and hydroquinone monomethyl ether (MEHQ, 4-methoxyphenol), or 4-tert-butylcatechol (TBC)]. Anhydrous anisole (100 mL) was added via cannula to the mixture followed by L-ascorbic acid (10 eq., 16.7 mmol) Cu(II)Br_2 (0.66 eq., 1.1 mmol), N,N,N′,N″,N″-pentamethyldiethylenetriamine (PMDETA, 3.29 eq., 5.6 mmol) and ethyl 2-bromo-2-methylpropionate (EBIB, 1 eq., 1.7 mmol). The mixture was slowly heated to 40 °C and left to react for 20 h. The polymerization solution changed colour from blue (oxidized copper) to colourless and then to yellow/orange over the course of 20 h. The polymer-grafted silica (PGS) was collected in a vacuum funnel. The PGS were washed multiple times with THF followed by sonication 3 times in THF. The particles were stirred in a 1 M EDTA solution (pH~10) for 1 h to remove any residual copper,

followed by drying under reduced atmosphere at 60 °C for 18 h and stored under argon until further use.

Synthesis of Ru@PGS

[Ru(2-methylallyl)$_2$(cod)] (128 mg, 0.401 mmol) was dissolved in DCM (10 mL) and added to a suspension of PGS (500 mg) in DCM (10 mL). The reaction mixture was stirred at room temperature for 1 h. After solvent removal at room temperature and *in vacuo* drying of the impregnated PGS, the powder was loaded into a 20 mL high-pressure autoclave and subjected to an atmosphere of H_2 (25 bar) at 100 °C for 18 h. Under this reducing environment, the impregnated PGS transformed from a light orange to a black colour indicating the immobilization of the Ru NPs onto the PGS.

Synthesis of Ru@SiO$_2$

[Ru(2-methylallyl)2(cod)] (128 mg, 0.401 mmol) was dissolved in DCM (10 mL) and added to a suspension of at 500°C dehydroxylated SiO_2 (500 mg) in DCM (10 mL). The reaction mixture was stirred at room temperature for 1 h. After solvent removal at room temperature and *in vacuo* drying of the impregnated SiO_2, the powder was loaded into a 20 mL high-pressure autoclave and subjected to an atmosphere of H_2 (25 bar) at 100 °C for 18 h. Under this reducing environment, the impregnated SiO_2 transformed from a white to a black colour indicating the immobilization of the Ru NPs onto the SiO_2.

Supplementary tables and figures

Characterization

Supplementary Table 1: BET and ICP data for SiO_2, PGS and Ru@PGS

Material	Surface Area ($m^2.g^{-1}$)	Pore Radius (Å)	ICP ($mmol.g^{-1}$)		SEM-EDX ($mmol.g^{-1}$)
			N	Ru	Ru
SiO_2	285	88	-	-	-
PGS	55	67	1.86	-	-
Ru@PGS	84	61	1.84	0.53	0.82

Solid-State ^{29}Si & ^{13}C CP-MAS NMR of PGS vs. Ru@PGS

The ^{29}Si & ^{13}C solid-state NMR spectra were obtained using a Bruker AVIII 500 spectrometer operating at 99.36 MHz and 125.76 MHz, respectively. The spectra were measured in a 4 mm standard zirconium oxide rotor spinning at 5 kHz employing cross polarization with SPINAL ^{1}H-decoupling during acquisition. The contact time was 6 ms (2 ms for ^{13}C NMR) and the recycle delay was set to 8 s. All spectra were calibrated to TMS according to the unified scale method recommended by IUPAC (10.1006/snmr.2002.0063) using adamantane as external reference. The star-marked signals (*) in the ^{13}C NMR spectra (at 105, 140, 220, 260 ppm) correspond to spinning sidebands.

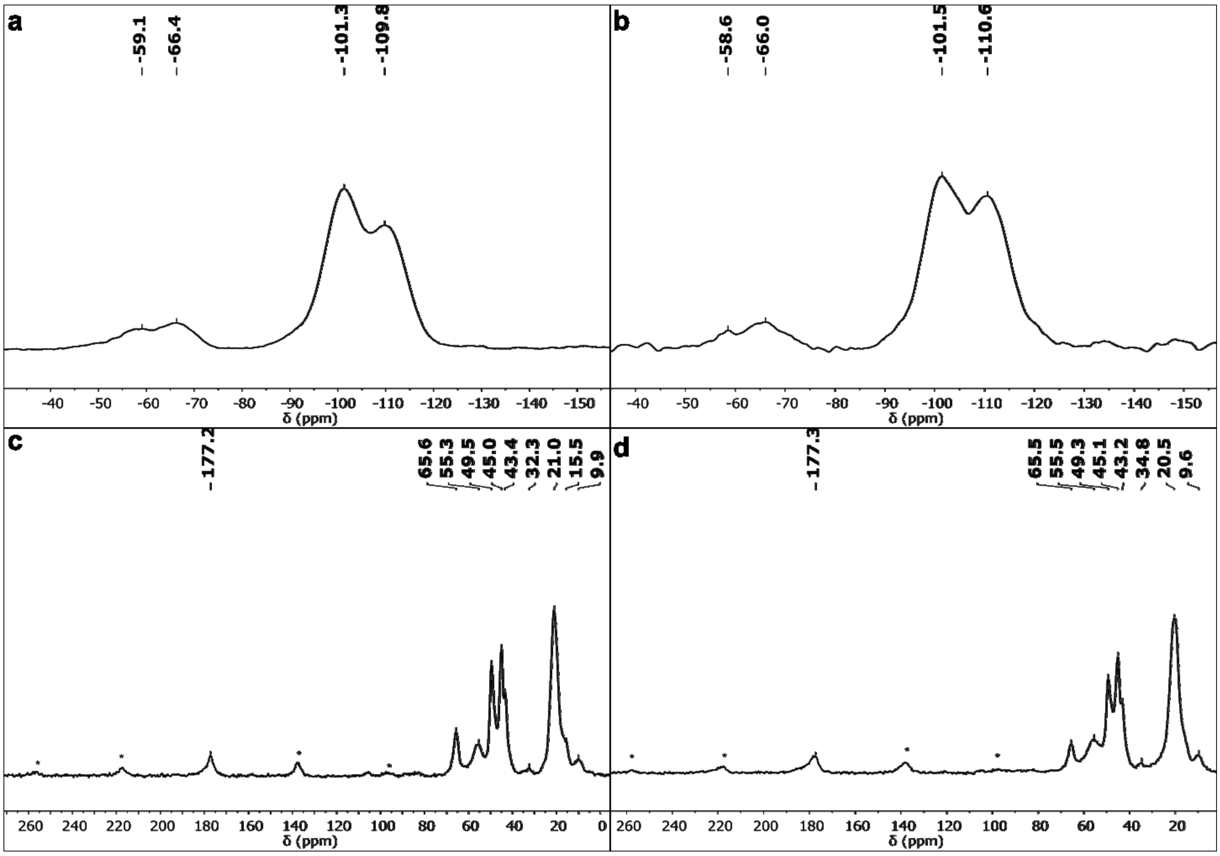

Supplementary Figure 1: Solid-state CP-MAS ^{29}Si NMR spectra (99.3 MHz) of **a**, PGS and **b**, Ru@PGS; and solid-state CP-MAS ^{13}C NMR spectra (125.7 MHz) of **c**, PGS and **d**, Ru@PGS. *spinning sidebands.

Supplementary Table 2: Hydrogenation of *N*-Methylacetamide and ethyl acetate using [Ru(2-methylallyl)$_2$(cod)] and Ru@PGS

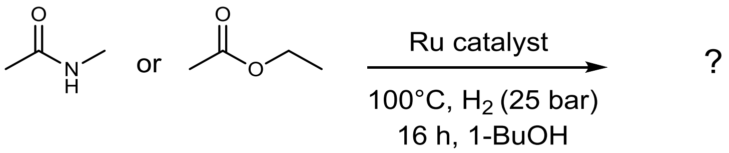

Catalyst	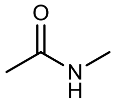 Conversion (%)	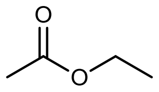 Conversion (%)
[Ru(2-methylallyl)$_2$(cod)]	0	0
Ru@PGS	0	0

Reactions conditions: Ru-Catalysts (35 mg, 0.026 mmol), substrate (0.65 mmol, 25 eq.), 1-butanol (0.5 mL), H_2 (25 bar), 100 °C, 16 h. Conversions determined by ^{1}H and ^{13}C NMR using mesitylene as internal standard.

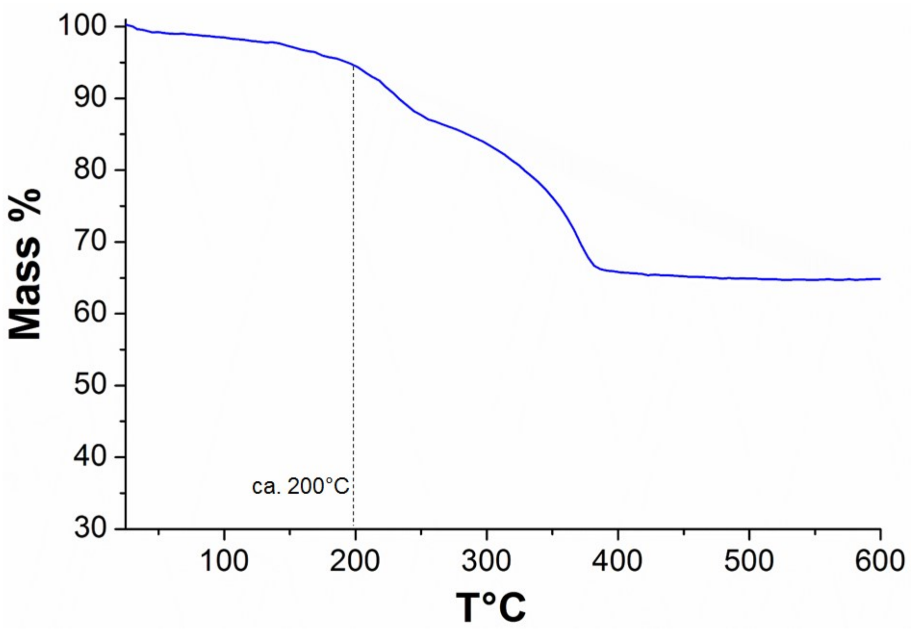

Supplementary Figure 2: Thermal stability of Ru@PGS investigated by thermogravimetric analysis under Ar. Conditions: 5°C/min under an argon flow of 100 mL/min.

Titration method

The procedure for the titration method was previously reported by Boniface et al.[1, 3] All vessels were plastic and they were rinsed thoroughly with Millipore water to have pH value of > 6. Stock solutions of sodium hydroxide (NaOH) 9.006 mM, and hydrochloric acid (HCl) 10.5 mM were prepared. In a typical experiment, ca. 10 mg of PGS or Ru@PGS was added to 20 mL of Millipore water in a 50 mL centrifuge vial. 0.1 mL aliquots of the 10.5 mM HCl were sequentially added to the centrifuge vial which was then tightly closed and vigorously shaken for 30 seconds. The pH was recorded before the next addition. The titration was stopped at ca. pH 4. The acidified sample was then centrifuged for 45 min at 2000 rpm. 15 mL of the acidified supernatant was transferred into a freshly washed centrifuge vial and was back-titrated with NaOH until ca. pH 7. This procedure was repeated twice (PGS1 & 2 and Ru@PGS1 & 2) providing close values for the accessible amine content (1.26 and

1.30 mmol. g^{-1}). A simple calculation for the titration of accessible tertiary amines (-NR_2) on PGS is as follows:

Moles of HCl added – Moles HCl left in supernatant = Moles of Accessible Amines

$$\frac{\text{Mole of Accessible amines}}{mg\ of\ sample\ used} * \frac{1000\ mmol}{1\ mol} * \frac{1000\ mg}{1\ g} = \frac{mmol}{g}$$

$\mathbf{C}_{supernatant} = \mathbf{n}_{NaOH} / \mathbf{V}_{supernatant}$

$\mathbf{V}_{supernatant} = \mathbf{V}_{HCl} + \mathbf{V}_{H2O}$

$\mathbf{n}_{supernatant} = \mathbf{C}_{supernatant} \bullet \mathbf{V}_{supernatant}$

$\mathbf{n}_{accessible\ amines} = \mathbf{n}_{HCl} - \mathbf{n}_{supernatant}$

Accessible amines = $\mathbf{n}_{accessible\ amines} / \mathbf{m}_{PGS}$

**PGS1**	**PGS2**
**m** = 10.1 mg	**m** = 10.2 mg
$\mathbf{V}_{H2O}$ = 20 mL	
$\mathbf{V}_{HCl}$ = 2.0 mL	$\mathbf{V}_{HCl}$ = 1.9 mL
$\mathbf{n}_{HCl}$ = 2.10E-5 mol	$\mathbf{n}_{HCl}$ = 1.99E-5 mol
$\mathbf{V}_{NaOH}$ = 0.6 mL	$\mathbf{V}_{NaOH}$ = 0.5 mL
$\mathbf{n}_{NaOH}$ = 5.403E-6 mol	$\mathbf{n}_{NaOH}$ = 4.503E-6 mol
$\mathbf{V}_{supernatant\ extract}$ = 15 mL	
$\mathbf{C}_{supernatant}$ = 3.602E-4 mol/L	$\mathbf{C}_{supernatant}$ = 3.002E-4 mol/L
$\mathbf{V}_{supernatant}$ = 22.0 mL	$\mathbf{V}_{supernatant}$ = 21.9 mL
$\mathbf{n}_{supernatant}$ = 7.925E-6 mol	$\mathbf{n}_{supernatant}$ = 6.574E-6 mol
$\mathbf{n}_{accessible\ amines}$ = 1.307E-5 mol	$n_{accessible\ amines}$ = 1.338E-5 mol
Accessible amines (-NR_2) = 1.26 mmol.g^{-1}	Accessible amines (-NR_2) = 1.30 mmol.g^{-1}

**Ru@PGS1**	**Ru@PGS2**
**m** = 11.0 mg	**m** = 10.5 mg
$\mathbf{V}_{H2O}$ = 20 mL	
$\mathbf{V}_{HCl}$ = 2.1 mL	$\mathbf{V}_{HCl}$ = 2.0 mL
$\mathbf{n}_{HCl}$ = 2.205E-5 mol	$\mathbf{n}_{HCl}$ = 2.10E-5 mol
$\mathbf{V}_{NaOH}$ = 0.7 mL	$\mathbf{V}_{NaOH}$ = 0.7 mL
$\mathbf{n}_{NaOH}$ = 6.304E-6 mol	$\mathbf{n}_{NaOH}$ = 6.304E-6 mol
$\mathbf{V}_{supernatant\ extract}$ = 15 mL	
$\mathbf{C}_{supernatant}$ = 4.203E-4 mol/L	$\mathbf{C}_{supernatant}$ = 4.203E-4 mol/L
$\mathbf{V}_{supernatant}$ = 22.1 mL	$\mathbf{V}_{supernatant}$ = 22.0 mL

**n**$_{supernatant}$ = 9.289E-6 mol	**n**$_{supernatant}$ = 9.246E-6 mol
**n**$_{accessible\ amines}$ = 1.276E-5 mol	n$_{accessible\ amines}$ = 1.175E-5 mol
Accessible amines (-NR_2) = 1.16 mmol.g^{-1}	Accessible amines (-NR_2) = 1.12 mmol.g^{-1}

Catalytic study under batch conditions

Hydrogenation of furfuralacetone (1) without CO_2

In a typical experiment, Ru-catalyst (35 mg, 0.026 mmol), 1-butanol (0.5 mL) were combined with **1** (90 mg, 0.65 mmol, 25 eq.) in a glass insert and placed in a high-pressure autoclave. After purging the autoclave with H_2, the reaction mixture was stirred at 80°C in an aluminium heating block under 15 bar of H_2. Once the reaction was finished, the reactor was cooled in an ice bath and carefully vented. After filtration, a sample of the reaction mixture was taken and analysed *via* GC-FID using tetradecane as an internal standard.

Hydrogenation of 1 with CO_2/H_2

In a typical experiment, Ru-catalyst (35 mg, 0.026 mmol), 1-butanol (0.5 mL) were combined with **1** (90 mg, 0.65 mmol, 25 eq.) in a glass insert and placed in a high-pressure autoclave. After purging with CO_2 and left to stir for a couple of minutes, the autoclave was further pressurized first with15 bar CO_2 and then with enough H2 to raise the total pressure to 30 bar (CO_2/H_2 ratio ~ 1:1). The reaction mixture was stirred 80°C in an aluminium heating block under desired pressure of H_2 and CO_2. Once the reaction was finished, the reactor was cooled in an ice bath and carefully vented. After filtration, a sample of the reaction mixture was taken and analysed *via* GC-FID using tetradecane as an internal standard.

Hydrogenation of other furan derivatives with H_2 or CO_2/H_2 .

In a typical experiment, Ru@PGS (35 mg, 0.026 mmol) and 1-butanol (0.5 mL) were combined with the substrate (0.65 mmol, 25 eq.) in a glass insert and placed in a high-pressure autoclave. After purging, the autoclave was pressurized with H_2 (and CO_2 with 1:1 ratio, if applicable) to raise the total pressure to the desired value. The reaction mixture was stirred at the desired temperature in an aluminium heating

block. Once the reaction was finished, the reactor was cooled in an ice bath and carefully vented. After filtration, a sample of the reaction mixture was taken and analysed *via* GC using tetradecane as an internal standard.

Switchability experiments in batch: Hydrogenation of 1

In a typical experiment, Ru-catalyst (35 mg, 0.026 mmol), 1-butanol (0.5 mL) were combined with **1** (90 mg, 0.65 mmol, 25 eq.) in a glass insert and placed in a high-pressure autoclave. After purging the autoclave with the respective gas, the reaction mixture was stirred at 80°C in an aluminium heating block under either H2 or a mixture of H_2 and CO_2. Once the reaction was finished, the reactor was cooled in an ice bath and carefully vented. The mixture was centrifuged and a sample of the solution was taken and analysed *via* GC-FID using tetradecane as an internal standard. For switchability, the reaction mixture was centrifuged, the supernatant removed and the residue was washed with 1-butanol (3 x 1 mL). The catalyst was dried at 100°C for 1 h. For the next cycle, fresh portions of the substrate (90 mg, 0.65 mmol, 25 eq.) with 1-butanol (0.5 mL) were added and the reaction mixture was performed again. This procedure was repeated for each catalyst cycle by alternatively pressurizing the autoclave either with only H_2 or with CO_2 and H_2.

Hydrogenation of 1 with various additives

In a typical experiment, Ru-catalyst (35 mg, 0.026 mmol), 1-butanol (0.5 mL) and the additive were combined with **1** (90 mg, 0.65 mmol, 25 eq.) in a glass insert and placed in a high-pressure autoclave. After purging the autoclave with H_2, the reaction mixture was stirred at 80°C in an aluminium heating block under 15 bar of H_2. Once the reaction was finished, the reactor was cooled in an ice bath and carefully vented. After filtration, a sample of the reaction mixture was taken and analysed via GC using tetradecane as an internal standard.

Supplementary Table 3: Parameter screening in the hydrogenation of furfuralacetone using Ru@PGS

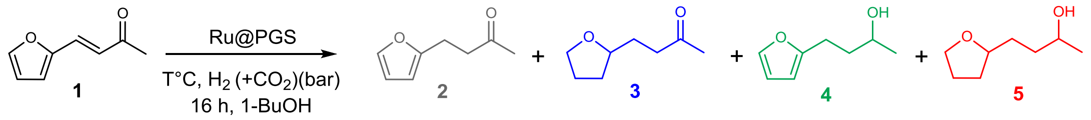

Entry	Ru Loading (wt%)	T (°C)	Pressure (bar)		Ratio	Product yield (%)[a]			
			H_2	CO_2	H_2:CO_2	2	3	4	5
1	0.3	80	60	–	–	80	17	3	–
2	0.3	100	60	–	–	74	22	4	–
3	0.3	100	100	–	–	60	36	2	2
4	1.2	80	60	–	–	75	20	4	1
5	2.5	80	60	–	–	19	75	1	5
6	5.0	80	60	–	–	–	77	1	22
7	7.5	80	60	–	–	–	13	–	87
8	7.5	80	50	10	5:1	–	65	–	35
9	7.5	80	40	20	4:2	–	77	0	23
10	7.5	80	30	30	1:1	–	89	–	11
11	7.5	80	20	20	1:1	–	86	–	14
12	7.5	80	15	15	1:1	–	**87**	–	13
13	7.5	80	15	–	–	–	15	–	**85**
14[b]	7.5	80	15	15	1:1	1	79	1	19
15[b]	7.5	80	15	–	–	–	8	–	92
16[c]	7.5	80	15	15	1:1	9	78	3	10
17[c]	7.5	80	15	–	–	–	11	–	89
18	7.5	80	10	10	1:1	13	75	3	9
19	7.5	80	5	5	1:1	42	49	6	3

Reactions conditions: Ru@PGS, substrate (25 eq.), 1-butanol (0.5 mL), 16 h. [a] Conversion > 99%, product yields were determined by GC-FID using tetradecane as an internal standard. [b] Heptane as solvent. [c] Methyl tert-butyl ether as solvent.

Supplementary Table 4: Hydrogenation of furfuralacetone (**1**, 100 eq.) using Ru@SiO_2 with H_2 and H_2/CO_2.

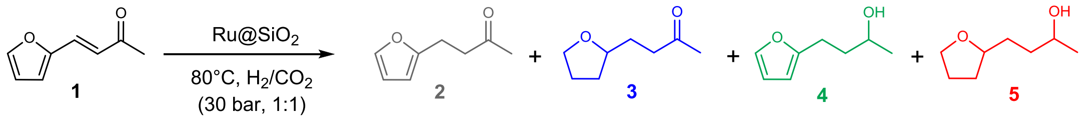

Entry	*p* [bar]		Product yield[a] [%]			
	H_2	CO_2	**2**	**3**	**4**	**5**
1	15	-	73	22	4	1
2	15	15	76	20	3	1

Reactions conditions: Ru@SiO_2 (35 mg, 0.026 mmol), substrate (2.6 mmol, 100 eq.), 1-butanol (0.5 mL), H_2 (15 bar), CO_2 (15 bar), 80 °C, 1 h. [a] Conversion > 99%, composition of the reaction mixture was determined by GC using tetradecane as an internal standard.

Supplementary Table 5: Hydrogenation of furfuralacetone (**1**) using Ru@SiO_2 and Ru@PGS under various reducing atmospheres.

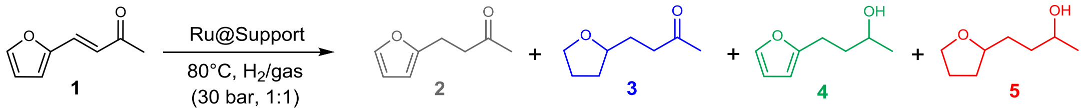

Entry	Catalyst	*p* [bar]			Product yield[a] [%]			
		H_2	Ar	CO_2	**2**	**3**	**4**	**5**
1	Ru@SiO_2	15	-	-	0	0	0	>99
2	Ru@SiO_2	15	15	-	0	0	0	>99
3	Ru@SiO_2	15	-	15	0	0	0	>99
4	Ru@PGS	15	-	-	0	61	0	39
5	Ru@PGS	15	15	-	0	60	0	40
6	Ru@PGS	15	-	15	52	44	3	1

Reactions conditions: Ru@Support (35 mg, 0.026 mmol), substrate (0.65 mmol, 25 eq.), 1-butanol (0.5 mL), H_2 (15 bar), Ar or CO_2 (15 bar), 80 °C, 2 h. [a] Conversion > 99%, composition of the reaction mixture was determined by GC using tetradecane as an internal standard.

Supplementary Table 6: Reaction time-profile for the hydrogenation of furfuralacetone under H_2 and H_2/CO_2 using Ru@PGS (data of Figure 3b-c).

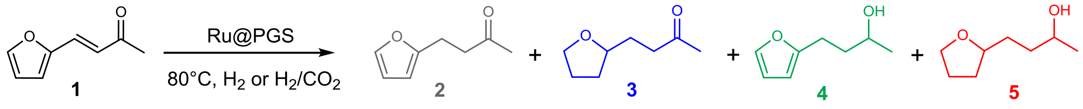

Entry	Reducing atmosphere	Time (h)	Product yield[a] [%] 2	3	4	5
1	H_2	0	0	0	0	0
2		1	8 ± 4	80 ± 0	4	8 ± 4
3		2	0	62 ± 2	0	38 ± 2
4		4	0	32 ± 3	0	68 ± 3
5		8	0	13 ± 2	0	87 ± 2
6		12	0	10 ± 0	0	90 ± 0
7		16	0	8 ± 1	0	92 ± 1
8		32	0	1 ± 0	0	99 ± 0
9	H_2/CO_2	0	0	0	0	0
10		1	64 ± 4	33 ± 3	2	1 ± 0
11		2	53 ± 3	43 ± 2	3	1 ± 0
12		4	12 ± 0	82 ± 3	1	5 ± 1
13		8	6 ± 0	84 ± 1	2	8 ± 1
14		12	2 ± 0	87 ± 0	1	10 ± 0
15		16	0	87 ± 1	0	13 ± 2
16		32	0	84 ± 0	0	16 ± 0

Reactions conditions: Ru@Support (35 mg, 0.026 mmol), substrate (0.65 mmol, 25 eq.), 1-butanol (0.5 mL), H_2 (15 bar), CO_2 (15 bar), 80 °C. [a] Conversion > 99%, composition of the reaction mixture was determined by GC using tetradecane as an internal standard.

Mechanistic investigations

^{1}H and ^{13}C NMR of the catalyst suspension

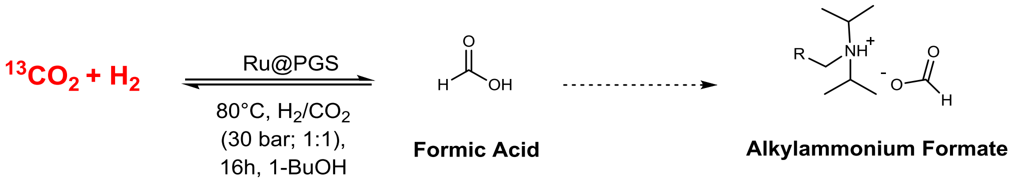

Ru@PGS (35 mg, 0.026 mmol) was combined with deuterated methanol (0.5 mL) however without furfuralacetone in a glass insert and placed in a high-pressure autoclave. After purging with an argon atmosphere, 2 bar labelled CO_2 were pressurized and left to stir for a couple of minutes. The autoclave was further pressurized with CO_2 up to 15 bar and then with enough H_2 to raise the total pressure to 30 bar. The reaction mixture was stirred at 80°C in an aluminium heating block under H_2 and CO_2. Once the reaction was finished, the reactor was cooled in an ice bath and carefully vented. The mixture of catalyst and solution was removed with a syringe, transferred into an NMR tube and analysed by ^{1}H, ^{13}C and 2D NMR spectroscopy. A heteronuclear single-quantum correlation spectroscopy (HSQC) was measured by 2D NMR spectroscopy.

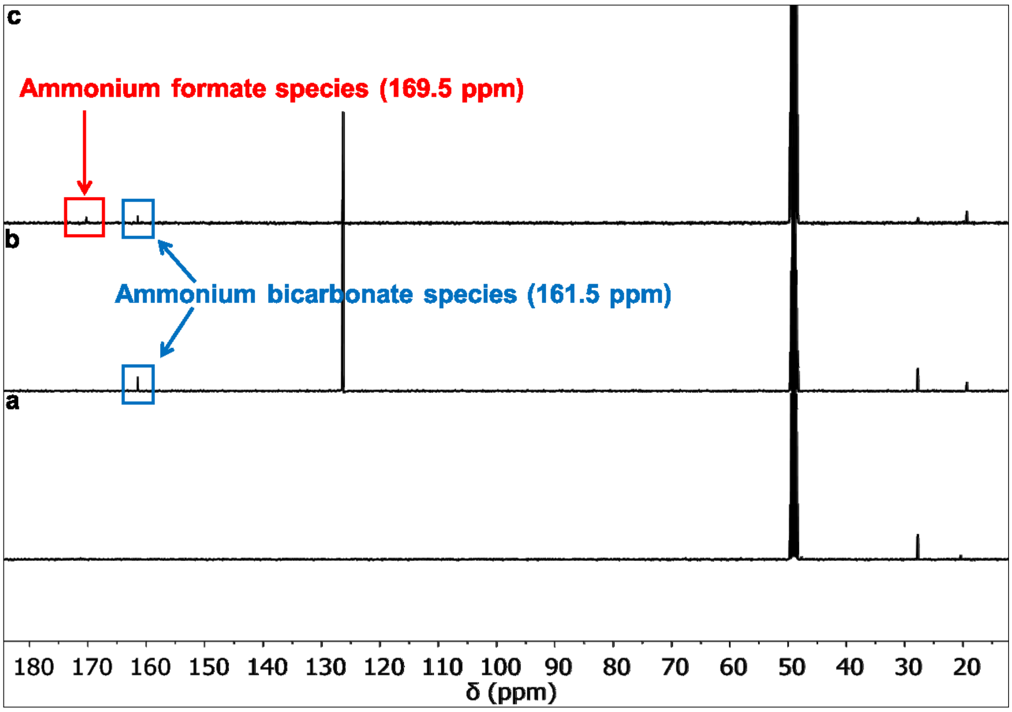

Supplementary Figure 3: ^{13}C NMR spectra (400 MHz, MeOD) of the reaction mixture of **a**, catalyst suspension (Catalyst + MeOD). **b**, catalyst suspension before catalysis (catalyst + MeOD + CO_2 + H_2). **c**, catalyst suspension after catalysis (catalyst + MeOD + CO_2 + H_2).

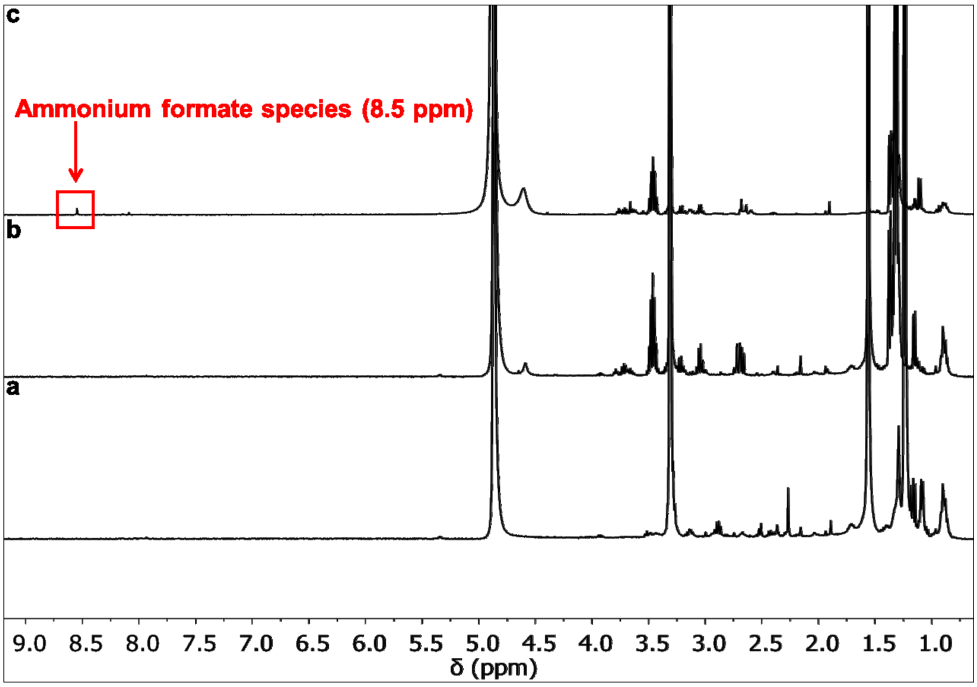

Supplementary Figure 4: ^{1}H NMR spectra (400 MHz, MeOD) of the reaction mixture of **a**, catalyst suspension (Catalyst + MeOD). **b**, catalyst suspension before catalysis (catalyst + MeOD + CO_2 + H_2). **c**, catalyst suspension after catalysis (catalyst + MeOD + CO_2 + H_2).

D_2-experiment: ^{13}C NMR of the catalyst suspension

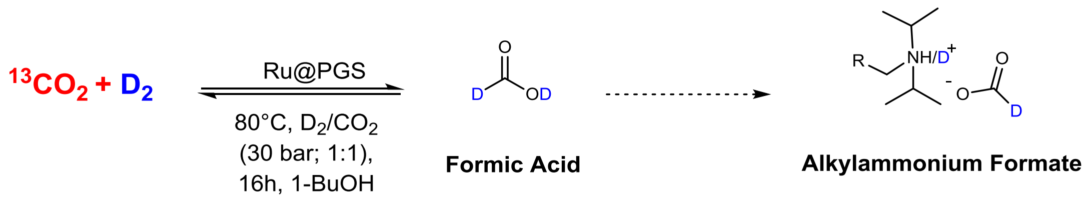

Ru@PGS (35 mg, 0.026 mmol) was combined with deuterated methanol (0.5 mL) without furfuralacetone in a glass insert and placed in a high-pressure autoclave. After purging with an argon atmosphere, 2 bar labelled CO_2 were pressurized and left to stir for a couple of minutes. The autoclave was further pressurized then with CO_2 up to 15 bar and then with enough deuterium (D_2) to raise the total pressure to 30 bar. The reaction mixture was stirred at 80°C in an aluminium heating block under H_2 and CO_2. Once the reaction was finished, the reactor was cooled in an ice bath and carefully vented. The mixture of catalyst and solution was removed with a syringe, transferred into an NMR tube and analysed by ^{13}C NMR spectra.

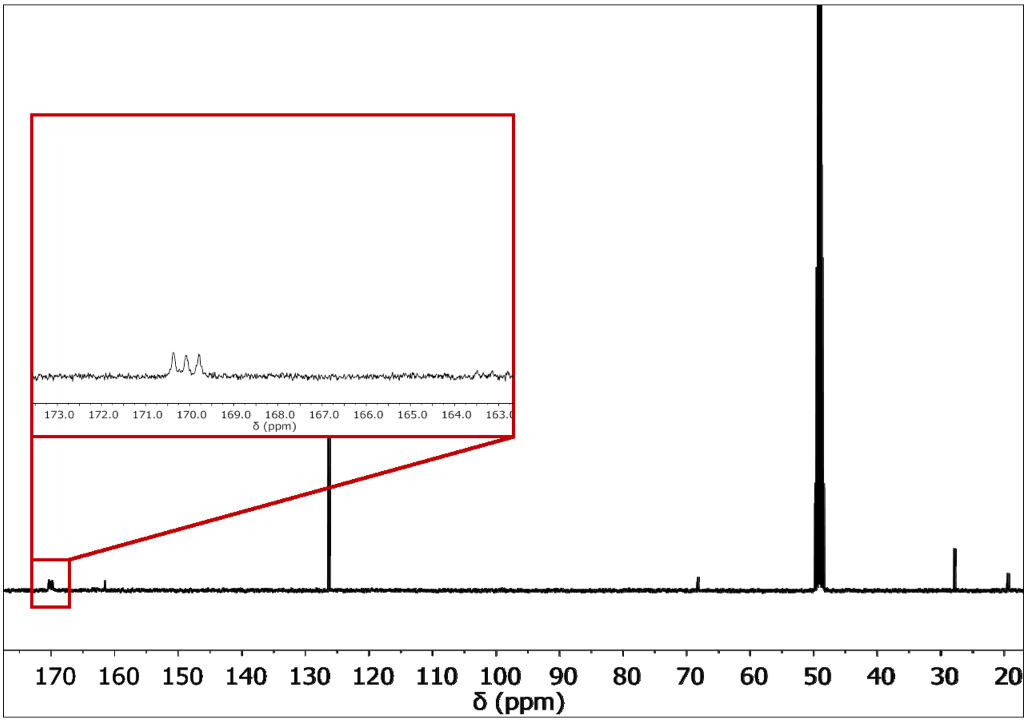

Supplementary Figure 5: ^{13}C NMR spectrum (400 MHz, MeOD) of the reaction mixture after catalysis using deuterium gas instead of H_2.

Solid-state ^{13}C CP-MAS NMR of the Ru@PGS-catalyst

Ru@PGS (35 mg, 0.026 mmol) was combined with deuterated methanol (0.5 mL) without furfuralacetone in a glass insert and placed in a high-pressure autoclave. After purging with an argon atmosphere, 2 bar labelled CO_2 were pressurized and left to stir for a couple of minutes. The autoclave was further pressurized then with CO_2 up to 15 bar and then with enough H_2 to raise the total pressure to 30 bar. The reaction mixture was stirred at 80°C in an aluminium heating block under H_2 and CO_2. Once the reaction was finished, the reactor was cooled in an ice bath and carefully vented. The reaction mixture was left to dry under air at room temperature for 48 h. The dried catalyst was then analysed by solid-state NMR spectroscopy. The parameters of the measurements are the same as stated above for solid-state NMR spectroscopy.

Supplementary Table 7: Hydrogenation of furfuralacetone (**1**) using Ru@Supports with various additives

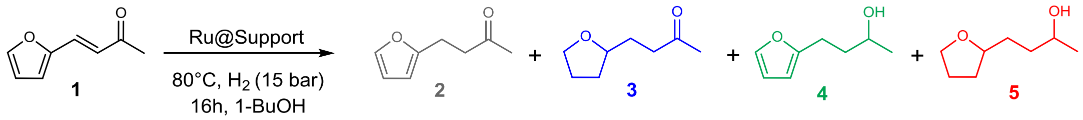

Entry	Catalysts	Additives	Product Yield (%)[a]			
			2	3	4	5
1	Ru@SiO$_2$	CO_2	0	4	0	96
2		DIPEF	4	71	4	9
3	Ru@PGS	CO_2	1	87	1	11
4		DIPEF	20	70	3	3

Reactions conditions: Ru-Catalysts (35 mg, 0.026 mmol), substrate (0.65 mmol, 25 eq.), additives: CO_2 (15 bar) or DIPEF (0.026 mmol, 1 eq. to Ru). 1-butanol (0.5 mL), H_2 (15 bar), 80 °C, 16 h. [a] Conversion > 99%, composition of the reaction mixture was determined by GC using tetradecane as an internal standard.

Switchability test in batch conditions

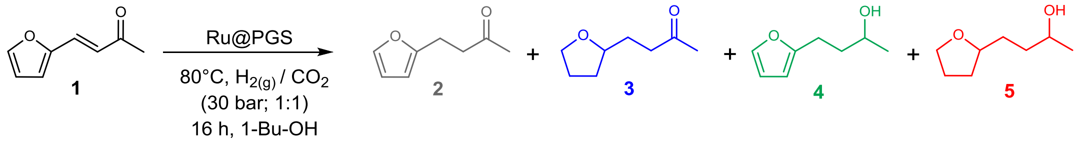

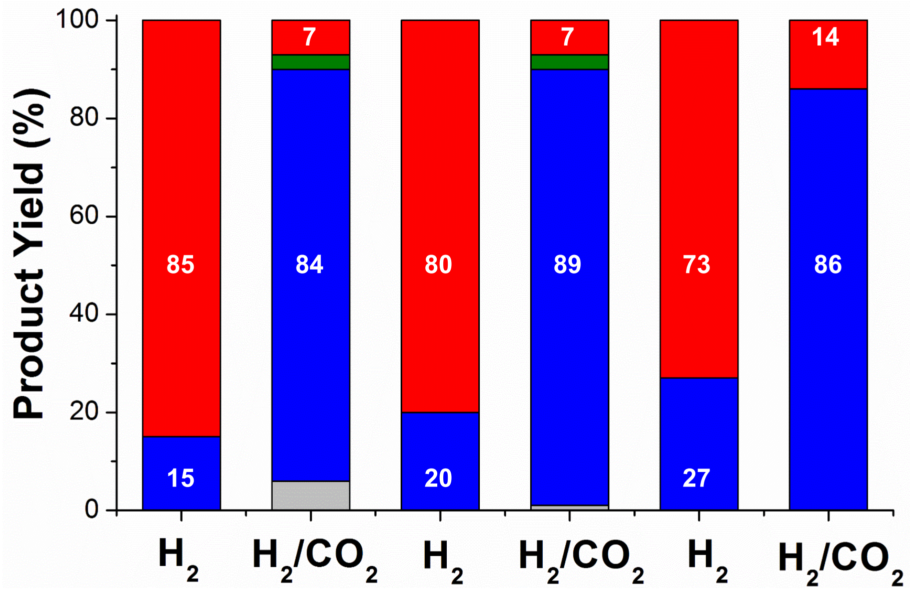

Supplementary Figure 6: Switchability tests in batch conditions: hydrogenation of furfuralacetone (**1**) using Ru@PGS with/without CO_2. Reactions conditions: Ru@PGS (35 mg, 0.026 mmol), substrate (0.65 mmol, 25 eq.), 1-butanol (0.5 mL), H_2 (15 bar), CO_2 (15 bar), 80 °C, 16 h. Composition of the reaction mixture was determined by GC-FID using tetradecane as an internal standard.

Continuous flow catalysis

Switchability experiments in continuous flow conditions: Hydrogenation of 1

The reactor was loaded with the Ru@PGS-catalyst (2.0 g) (reactor volume = 3.2 mL) and installed into the continuous flow setup. The system was pressurized with H_2, heated to the desired temperature and the pump was loaded with the substrate solution. Substrate and gas flows were mixed in a volume flow mixer (alternately H_2 and H_2/CO_2) and the feed flow was passed through the heated reactor and samples were collected at the output. The composition of the reaction mixture was determined by GC-FID using tetradecane as an internal standard. For switching to H_2 and CO_2,

the CO_2 flow rate was started (35 mL.min^{-1}) and the total pressure at BPR (back pressure regulator) was set up to 40 bar. The first samples were collected after 15 min on stream. For the switch to only H_2, the H_2 and CO_2 flow rates were turned off, as well as the substrate solution flow. The catalyst was streamed with synthetic air (3 bar) for 1 h at 100°C, until no solvent was observed at the output. The substrate and H_2 flow rate were started again, with a total pressure at BPR set back to 20 bar. This procedure was repeated for each atmosphere switch.

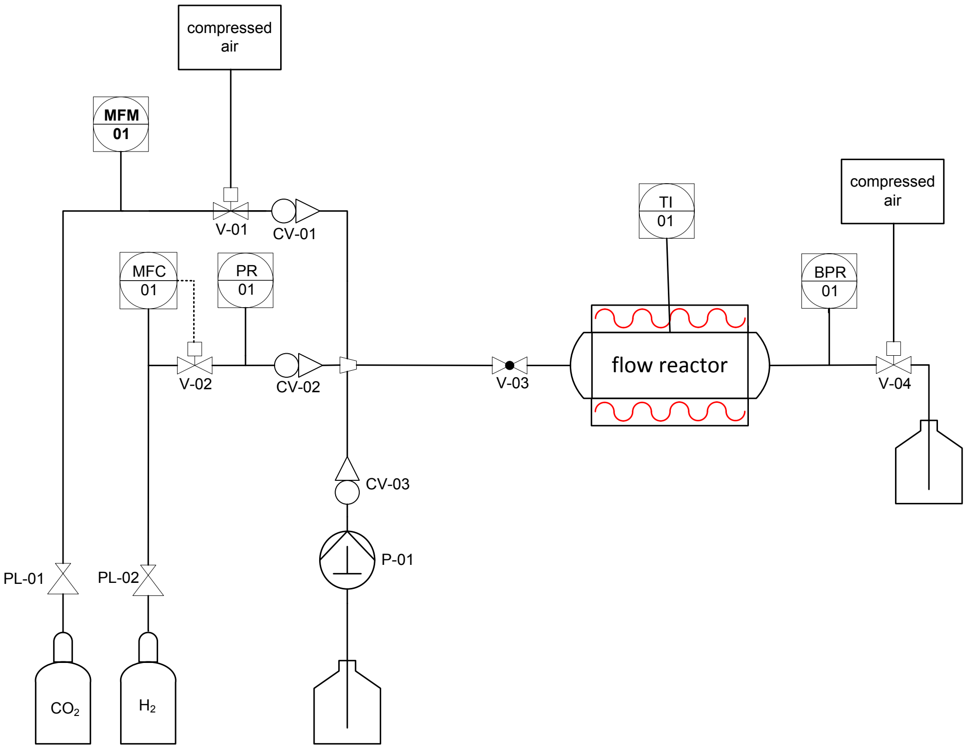

Supplementary Figure 7: Scheme of the In-house built continuous flow setup. PL = pressure inducer; MFM = mass flow meter; MFC = mass flow controller; P = HPLC-pump; V = valve; CV = Check valve; PR = pressure sensor; TI = temperature inducer; BPR = Back pressure regulator.

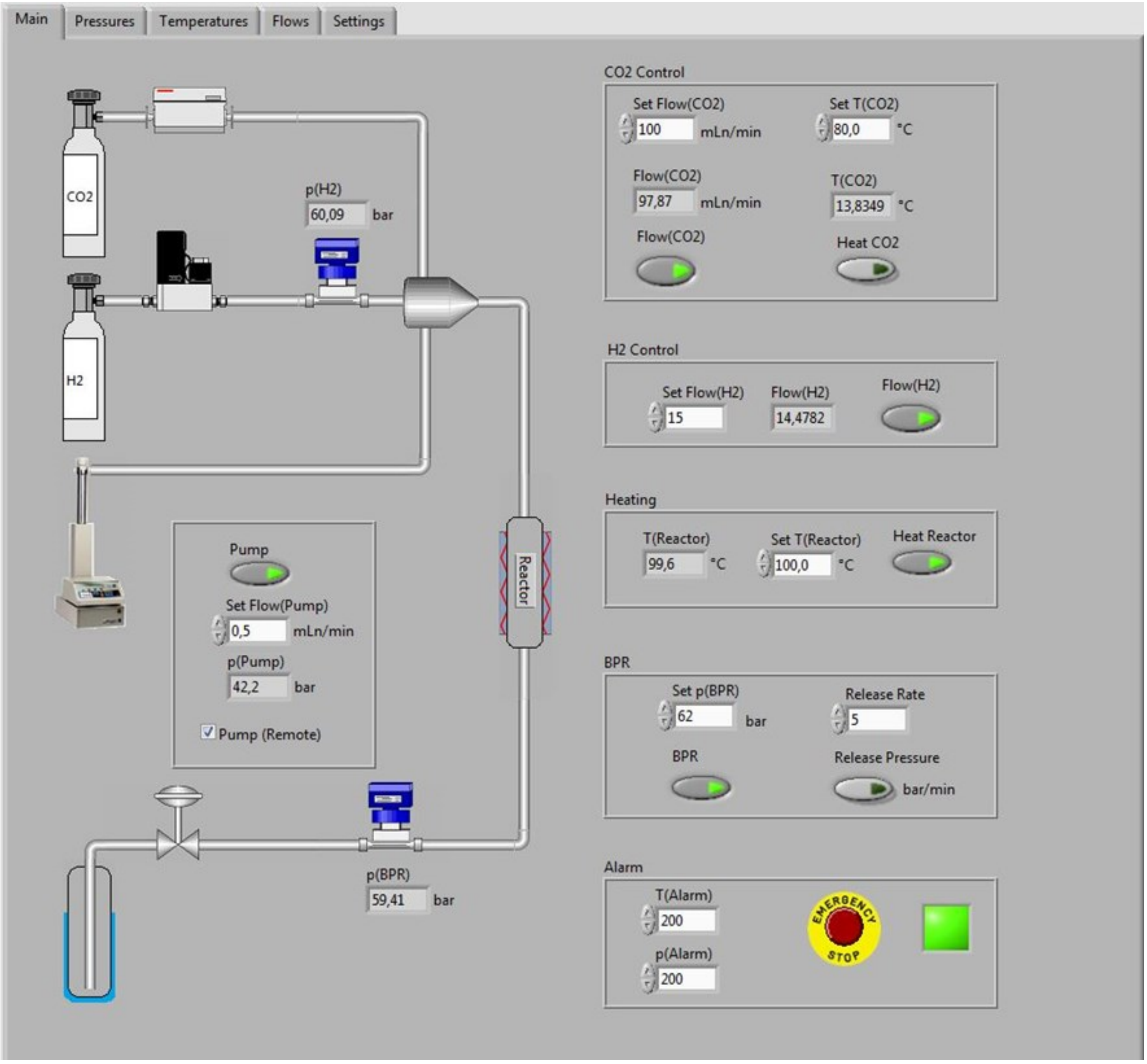

Supplementary Figure 8: Graphical surface interface of the program used to control the continuous flow setup.

Supplementary Table 8: Parameter screening for the hydrogenation of furfuralacetone (**1**) using Ru@PGS in continuous flow[a]

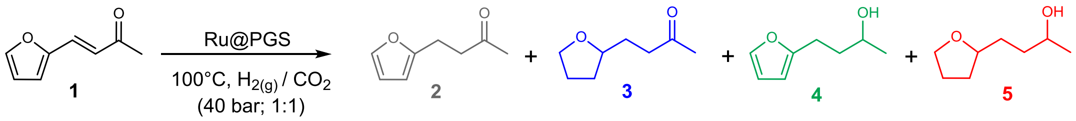

T [°C]	*p* [bar]		Substrate flow [mL.min^{-1}]	Substrate conc.	Catalyst [g]	Product distribution[a] [%]			
	H_2	CO_2				2	3	4	5
80	10	10	0.5	0.05 M	0.5	41	59	0	0
80	15	15	0.5	0.05 M	0.5	40	60	0	0
80	20	20	0.5	0.05 M	0.5	29	71	0	0
80[b]	10	10	1.0	0.05 M	0.5	61	35	0	0
80[b]	15	15	1.0	0.05 M	0.5	51	40	0	0
80	20	20	1.0	0.05 M	0.5	35	65	0	0
100	10	10	0.5	0.05 M	0.5	19	78	0	3
100[b]	10	10	1.0	0.05 M	0.5	37	59	0	0
100	20	20	1.0	0.05 M	0.5	10	85	0	5
100	20	20	0.5	0.05 M	0.5	0	99	0	1
100	20	-	0.5	0.05 M	0.5	0	65	0	35
100	20	20	0.5	0.025 M	0.5	0	80	0	20
100[c]	20	-	0.5	0.025 M	0.5	0	60	0	40
100	20	-	0.5	0.025 M	2.0	0	7	0	93
100	20	20	0.5	0.025 M	2.0	0	73	0	27
100	7	33	0.5	0.025 M	2.0	0	81	0	19
100	12	28	0.5	0.025 M	2.0	0	80	0	20
100	16	24	0.5	0.025 M	2.0	0	78	0	22
100[c]	20	-	0.5	0.025 M	2.0	0	25	0	75
100[d]	20	20	0.5	0.025 M	2.0	0	90	0	10
100[e]	20	-	0.5	0.025 M	2.0	0	13	0	87

[a]Reactions conditions: Ru@PGS, substrate (in 1-butanol), residence time = 6.4 min for substrate flow rate = 0.5 mL.min^{-1}, residence time = 3.2 min for 1 mL.min^{-1}), gas flow rate = 35 NmL.min^{-1}. Composition of the reaction mixture was determined by GC using tetradecane as an internal standard. Conversion > 99%. [b] Conversion < 99%. [c] Catalyst was washed with argon for 1 h before switching to H_2 pressure. [d] Catalyst treated with H_2/CO_2 for 15 min. [e] Catalyst washed under air for 1 h before applying the H_2 pressure.

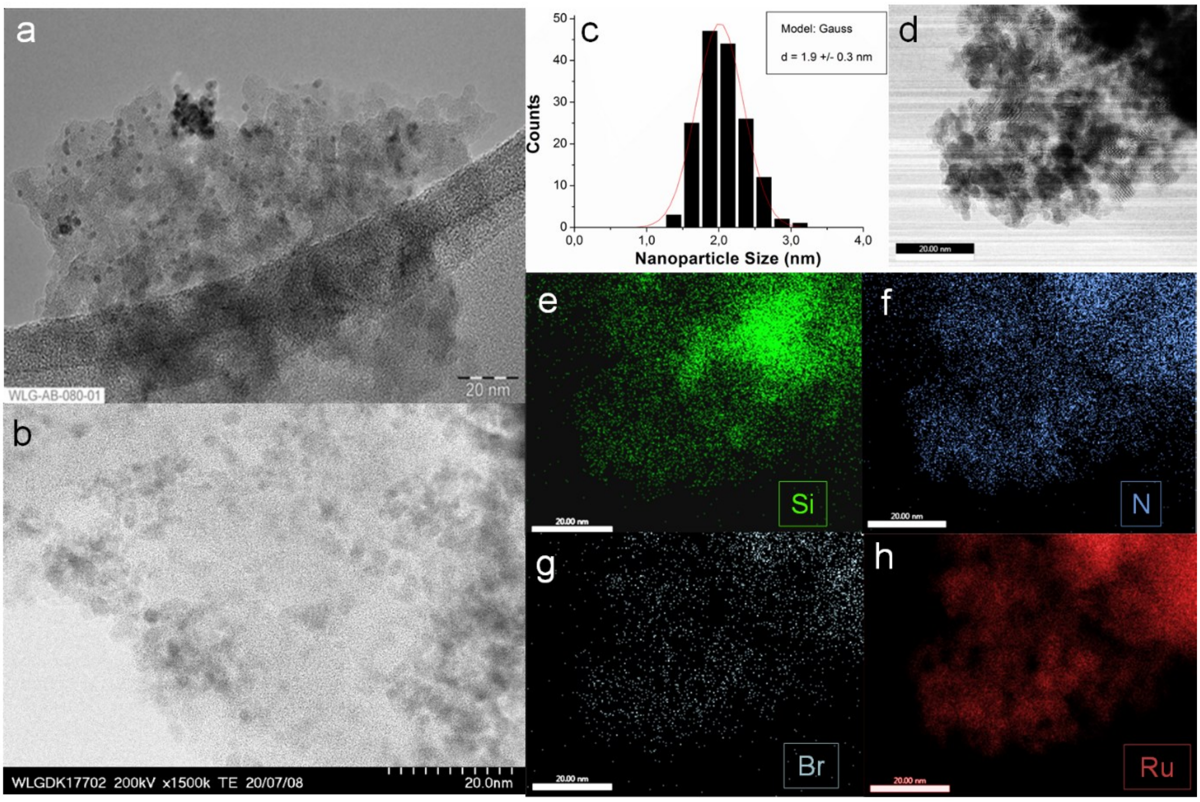

Supplementary Figure 9: Characterization of the Ru@PGS catalyst by electron microscopy after continuous flow catalysis. **a** and **b**, Transmission electron micrographs. **c**, histogram. **d**, Scanning transmission electron micrograph. **e-h**, Scanning transmission electron micrographs in high angle annular dark field with elemental mapping (**e**: Si, **f**: N, **g**: Br, **h**: Ru) using energy dispersive X-ray spectroscopy.

Supplementary Table 9: BET and ICP data for Ru@PGS after continuous flow catalysis

Sample	Surface Area ($m^2.g^{-1}$)	Pore Radius (Å)	ICP ($mmol.g^{-1}$)		SEM-EDX ($mmol.g^{-1}$)
			N	Ru	Ru
Before Catalysis	84	61	1.84	0.53	0.82
After Catalysis	177	57	1.28	0.54	0.78

Additional characterization data

Polymer grafted silica (PGS)

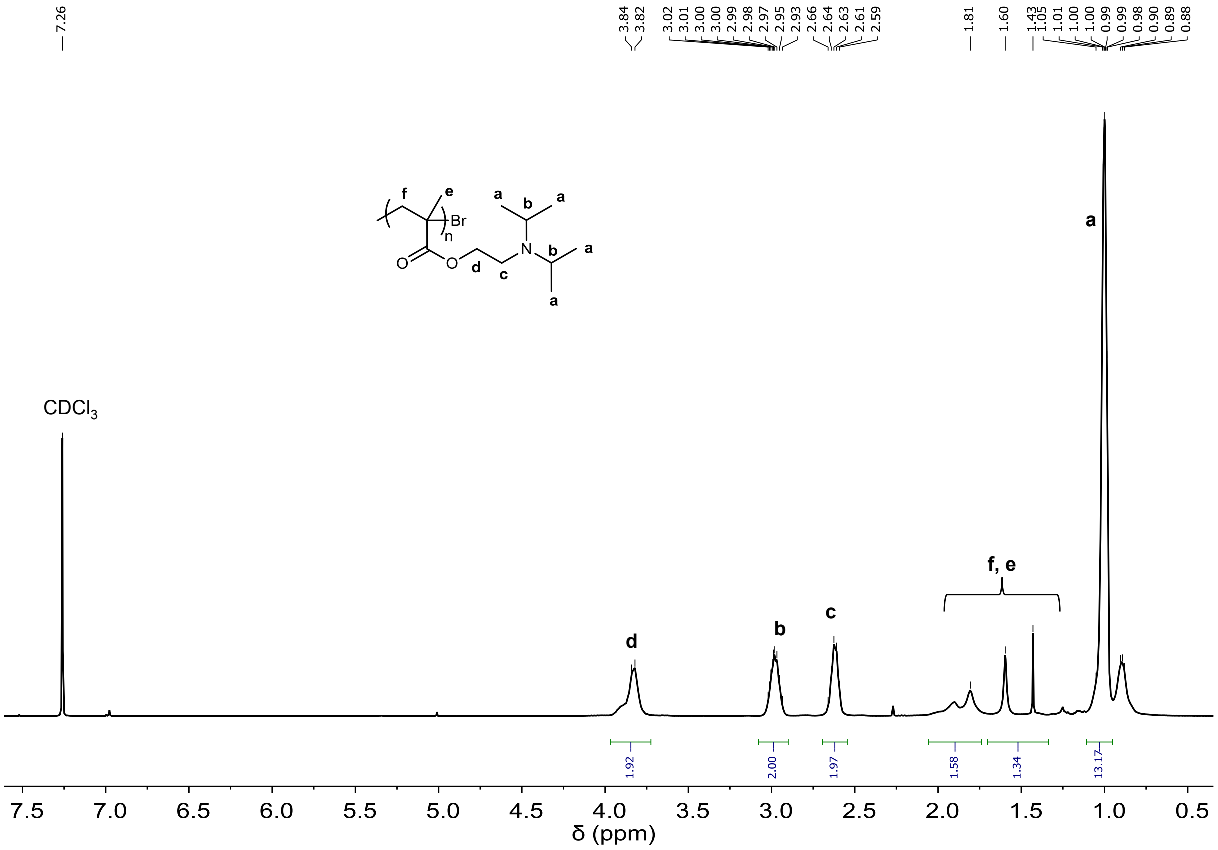

Supplementary Figure 10: ^{1}H NMR spectrum ($CDCl_3$, 400 MHz) of the free polymer (pDiPAEMA).

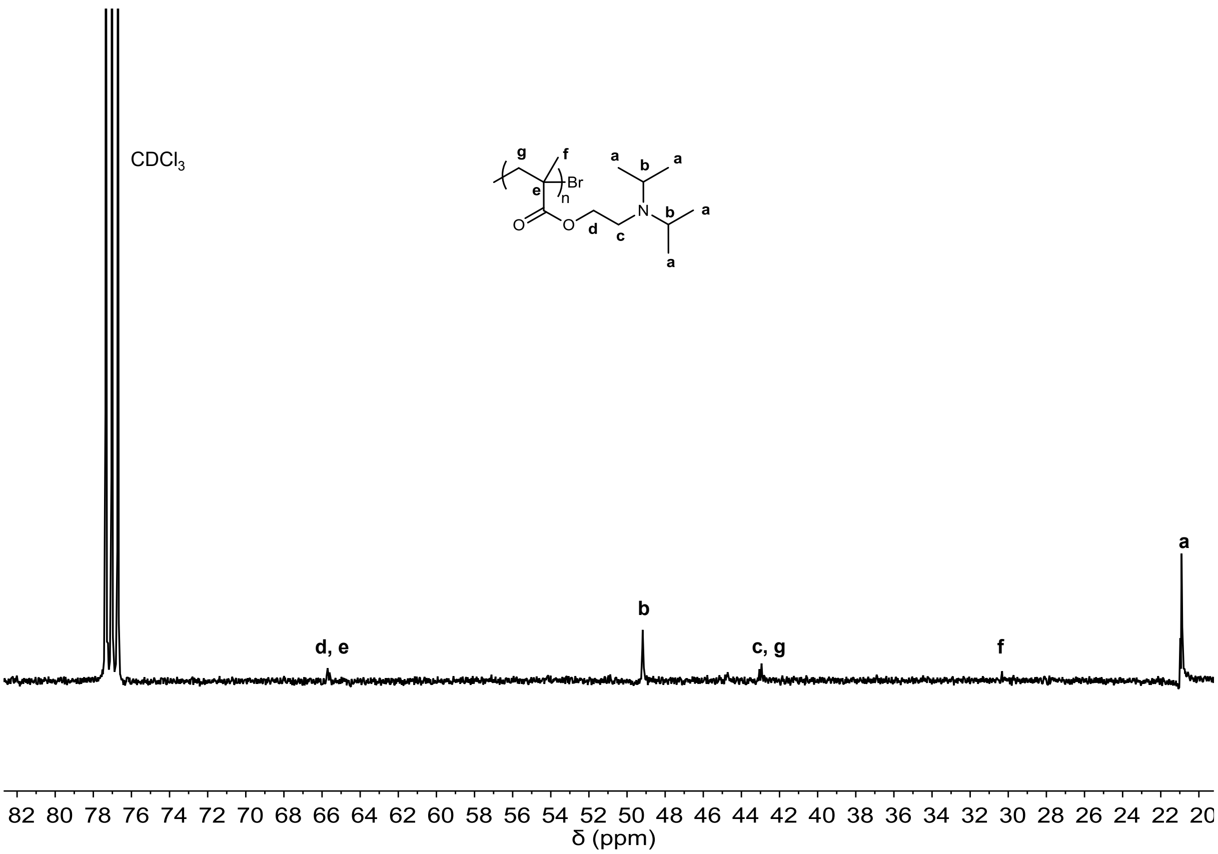

Supplementary Figure 11: ^{13}C NMR spectrum ($CDCl_3$, 100 MHz) of the free polymer (pDiPAEMA).

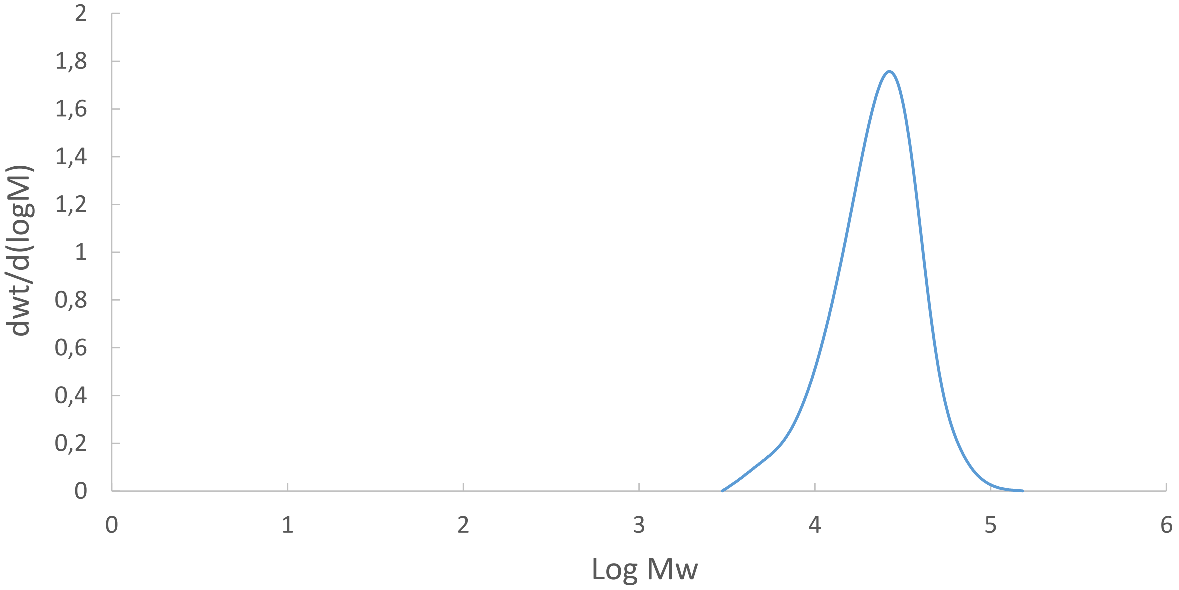

Supplementary Figure 12: Gel permeation chromatography (GPC) trace for free pDiPAEMA (same polymer as on the PGS) grown from sacrificial initiator.

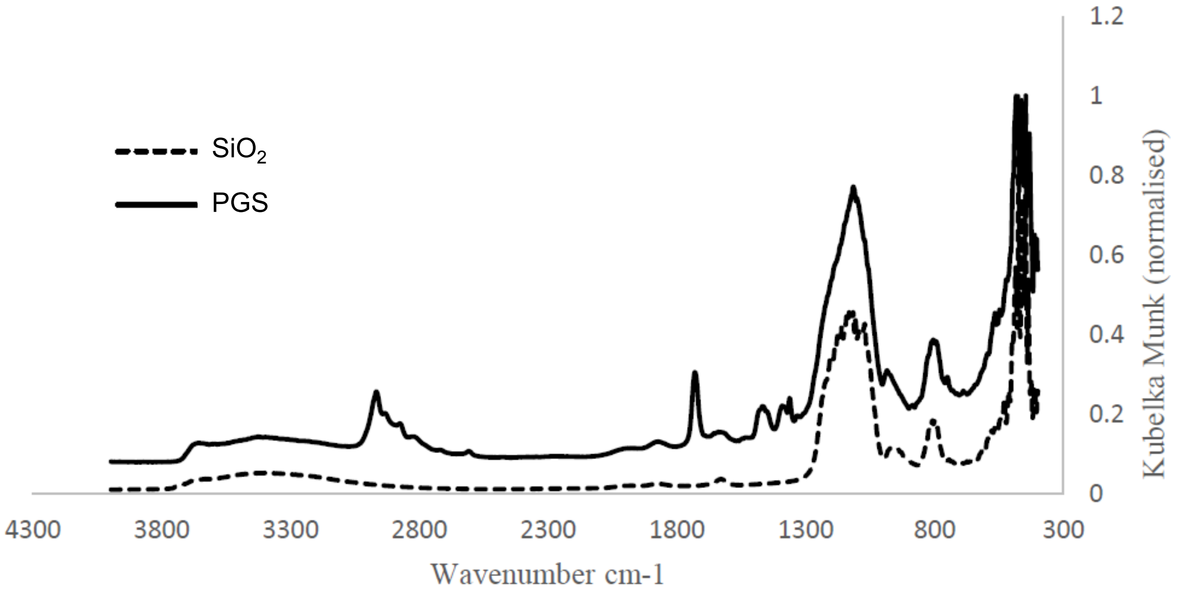

Supplementary Figure 13: DRIFT-IR spectra of silica particles before polymer attachment (S_IO_2) and after polymer attachment (PGS).

Supplementary Figure 13 illustrates a typical spectrum that is recorded for a polymer-grafted silica sample, juxtaposed to a control spectrum. PGS consists of silica particles that have been grafted with PDiPAEMA. The control sample is the same silica particles used for PGS but without chemical modification of any means. Comparison of these two spectra allows for the identification of target regions in which one looks to determine whether a given sample of silica contains polymer. The regions between 1300 cm^{-1} to 2000 cm^{-1} and 2800 cm^{-1} to 3000 cm^{-1} are the target regions in which the presence of peaks are indicative of functionalization with PDiPAEMA.

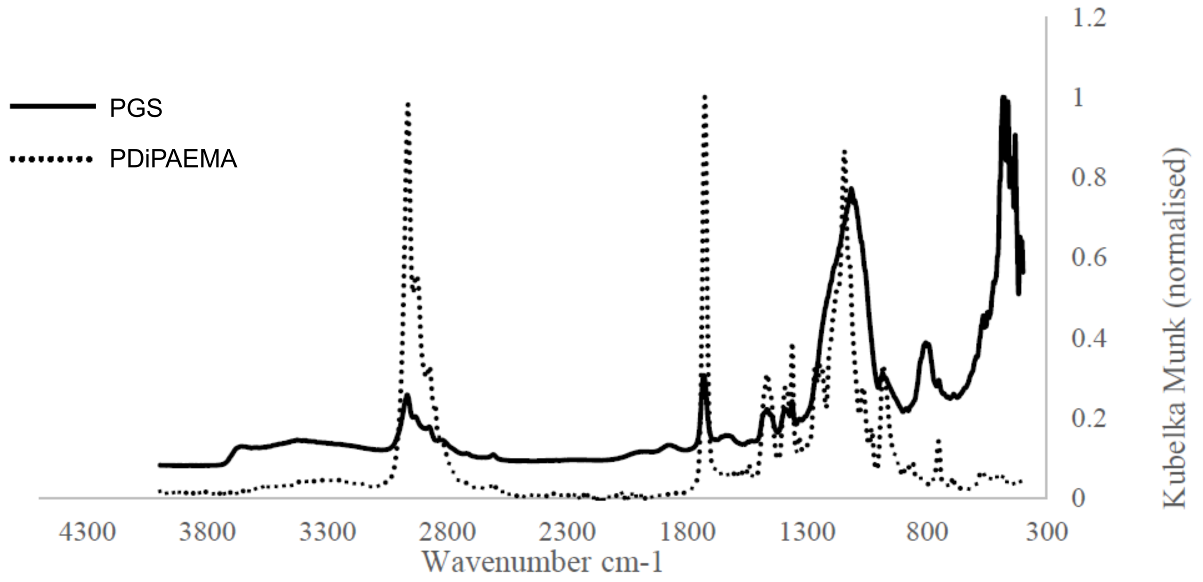

Supplementary Figure 14: DRIFT-IR spectra of the free polymer and of PGS.

Supplementary Figure 14 illustrates the same PGS sample that was shown in Supplementary Figure 13 juxtaposed against a spectrum collected from a sample of PDiPAEMA homopolymer. The homopolymer was precipitated twice; the ^{1}H NMR spectrum of the twice precipitated polymer did not show signs of residual monomer. Supplementary Figure 14 clearly indicates that the target regions identified from analysis of Supplementary Figure 13 (1300 cm^{-1} to 2000 cm^{-1} and 2800 cm^{-1} to 3000 cm^{-1}) are resultant from PDiPAEMA and not from an impurity. The best agreements between peaks that are characteristic of functionalization with PDiPAEMA are 1727 ±2 cm^{-1} and 2874 ±1 cm^{-1}.

Ru@SiO$_2$

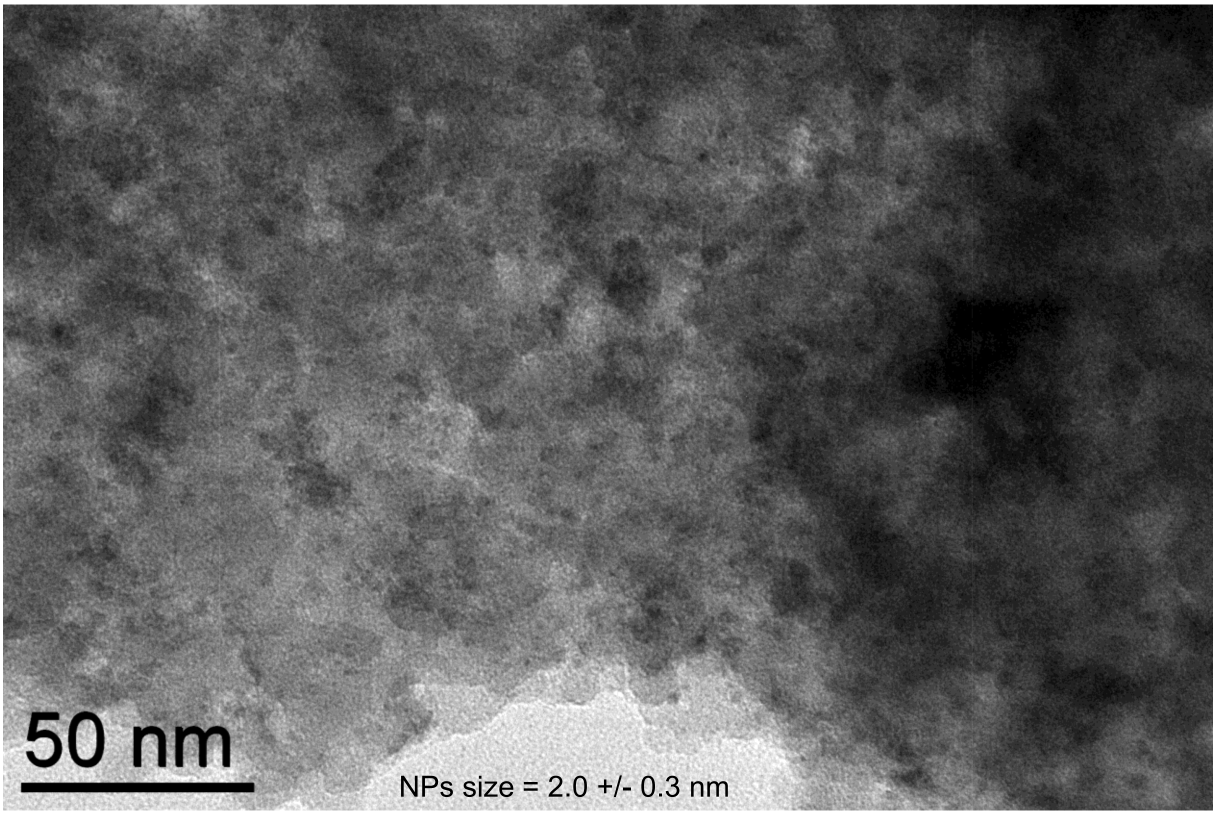

Supplementary Figure 15: Transmission electron microscopy image of Ru@SiO$_2$.

GC Chromatograms

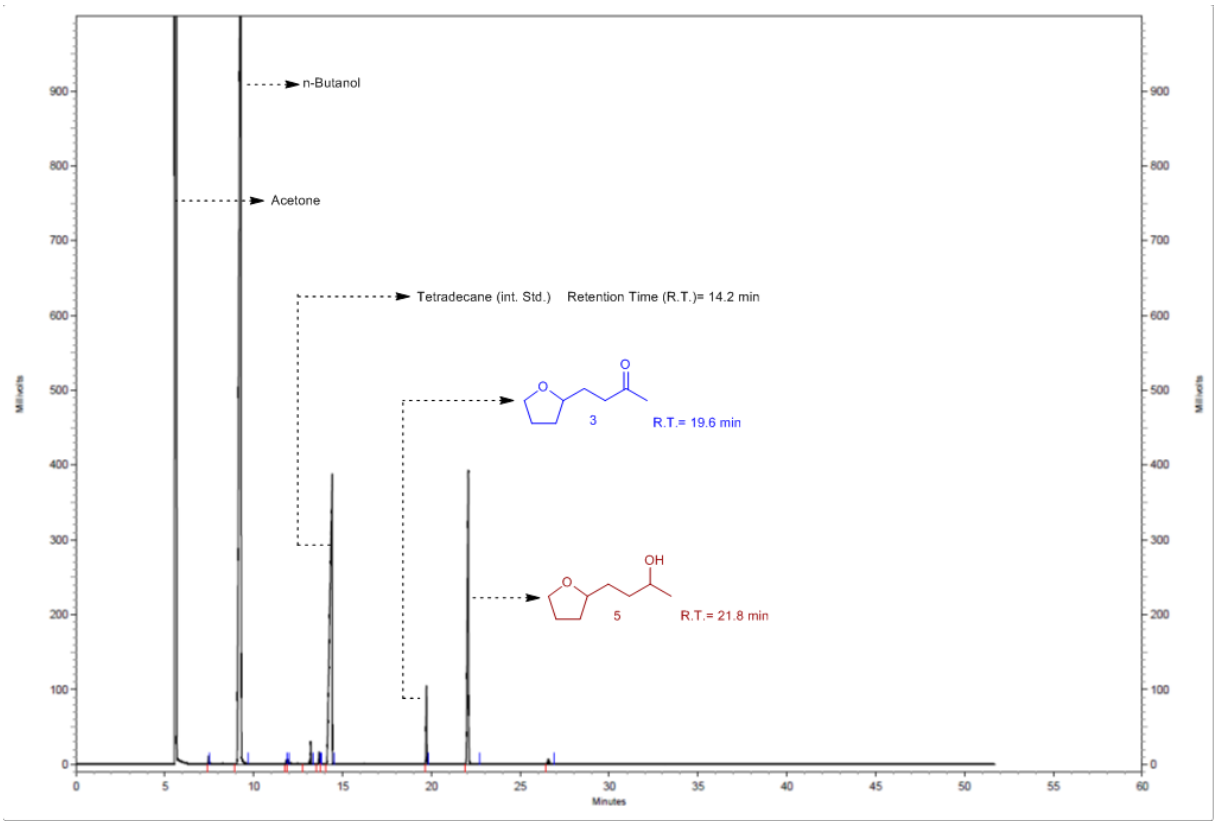

Supplementary Figure 16: Chromatogram corresponding to Figure 3b (Ru@PGS, H_2).

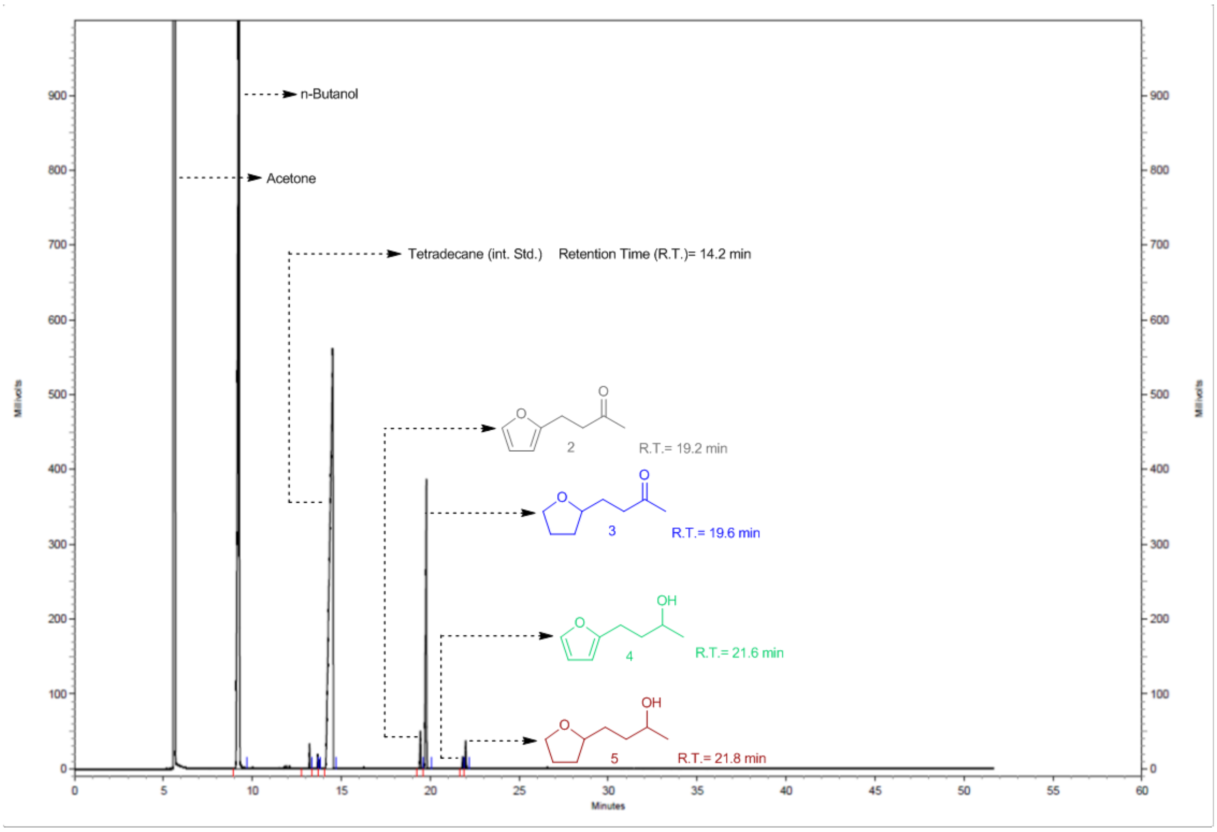

Supplementary Figure 17: Chromatogram corresponding to Figure 3b (Ru@PGS, H_2/CO_2).

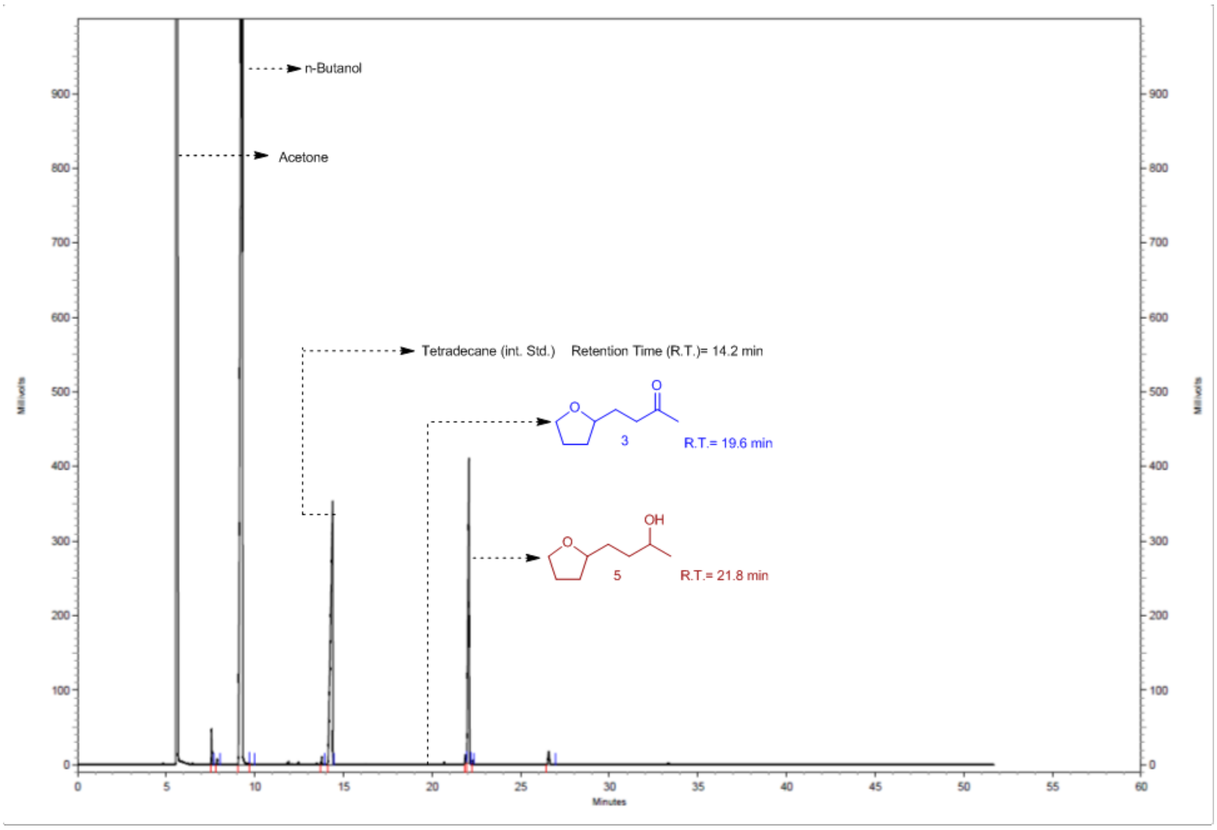

Supplementary Figure 18: Chromatogram corresponding to Figure 3b (Ru@SiO_2, H_2).

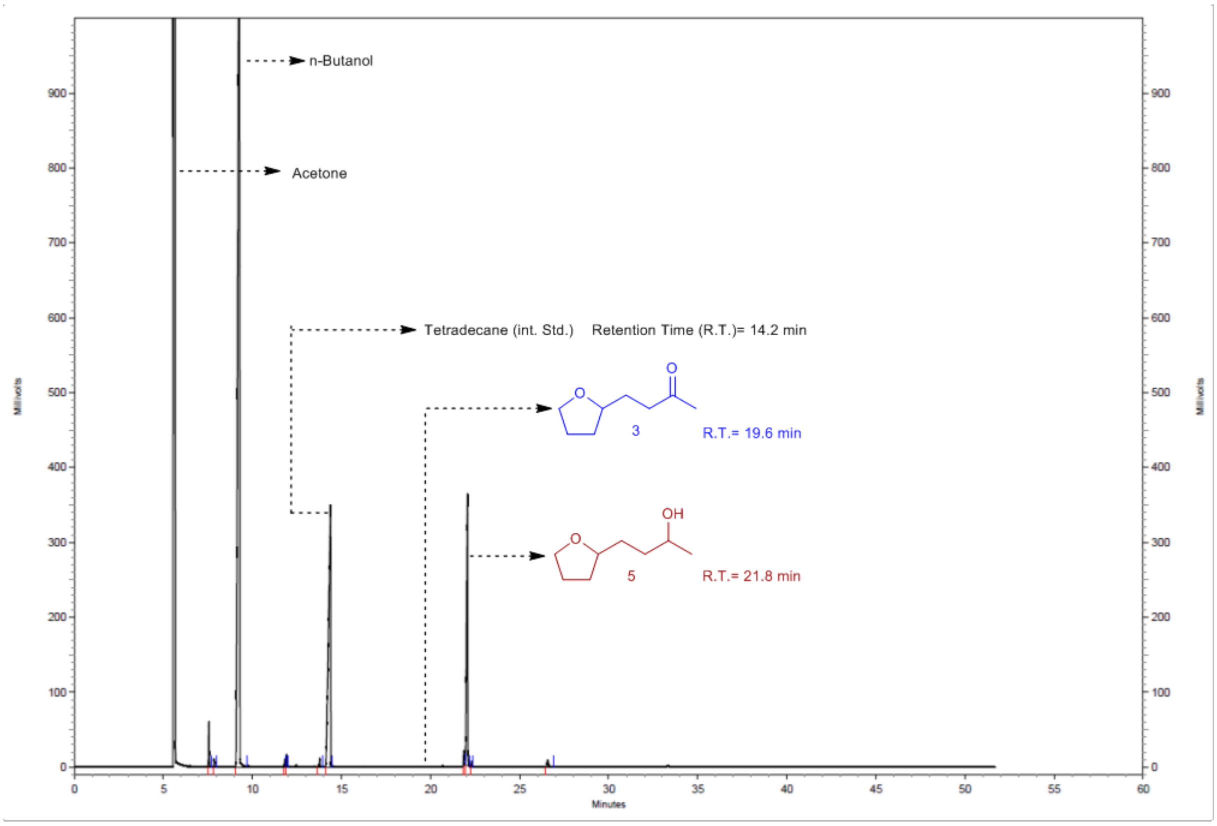

Supplementary Figure 19: Chromatogram corresponding to Figure 3b (Ru@SiO_2, H_2/CO_2).

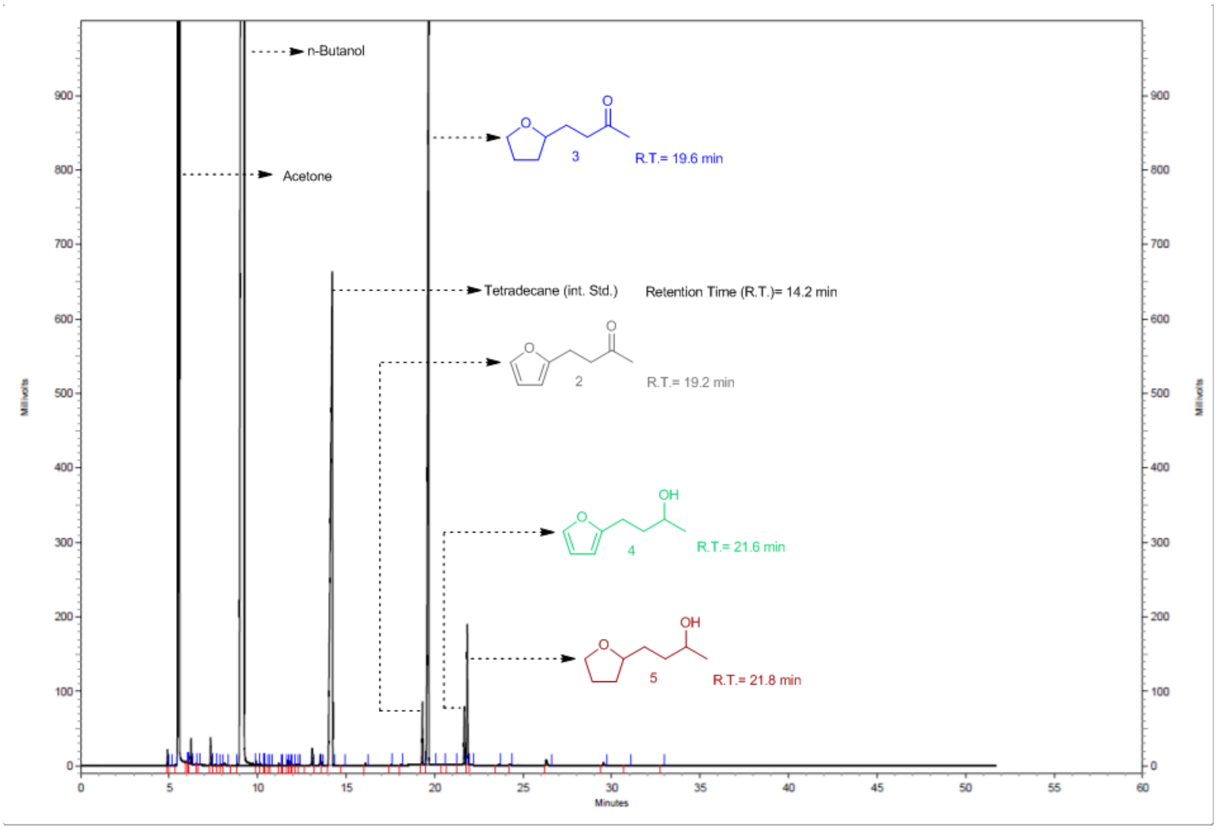

Supplementary Figure 20: Chromatogram corresponding to Figure 3c (Ru@PGS, H_2, 1 h).

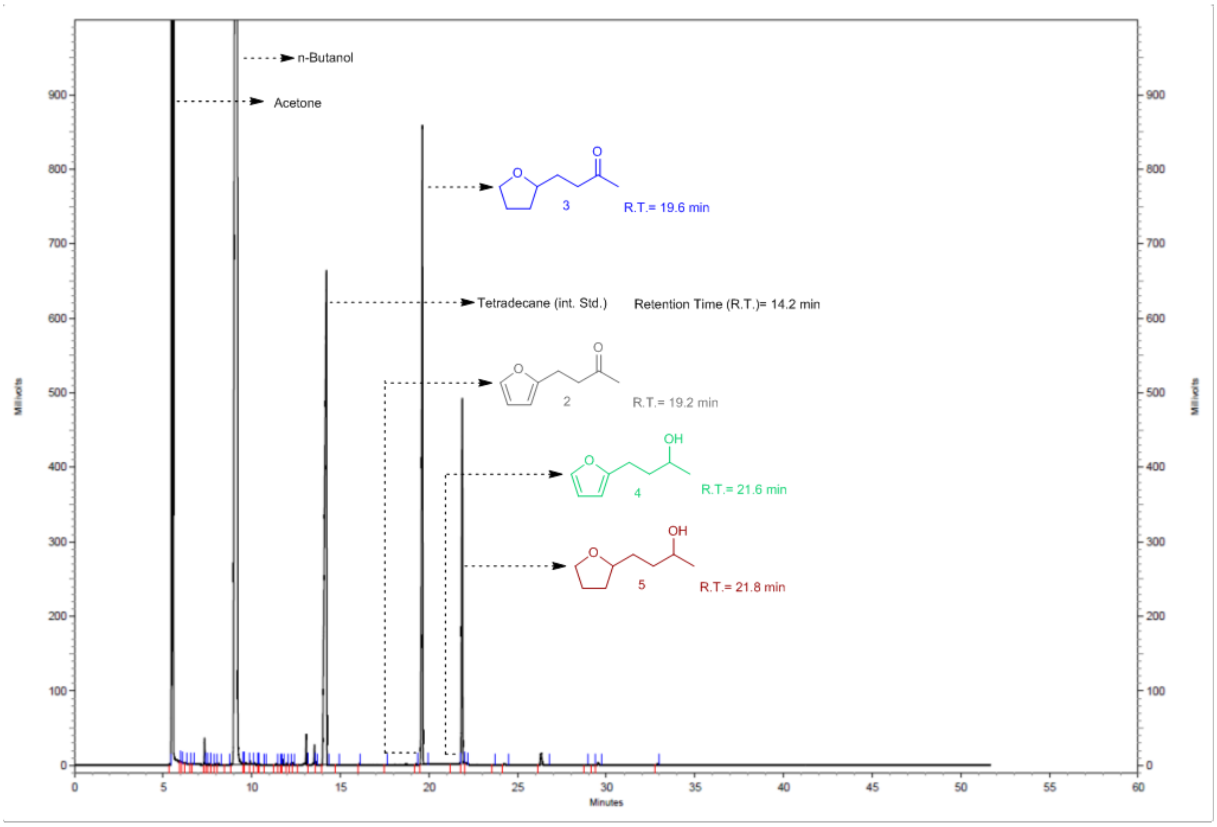

Supplementary Figure 21: Chromatogram corresponding to Figure 3c (Ru@PGS, H_2, 2 h).

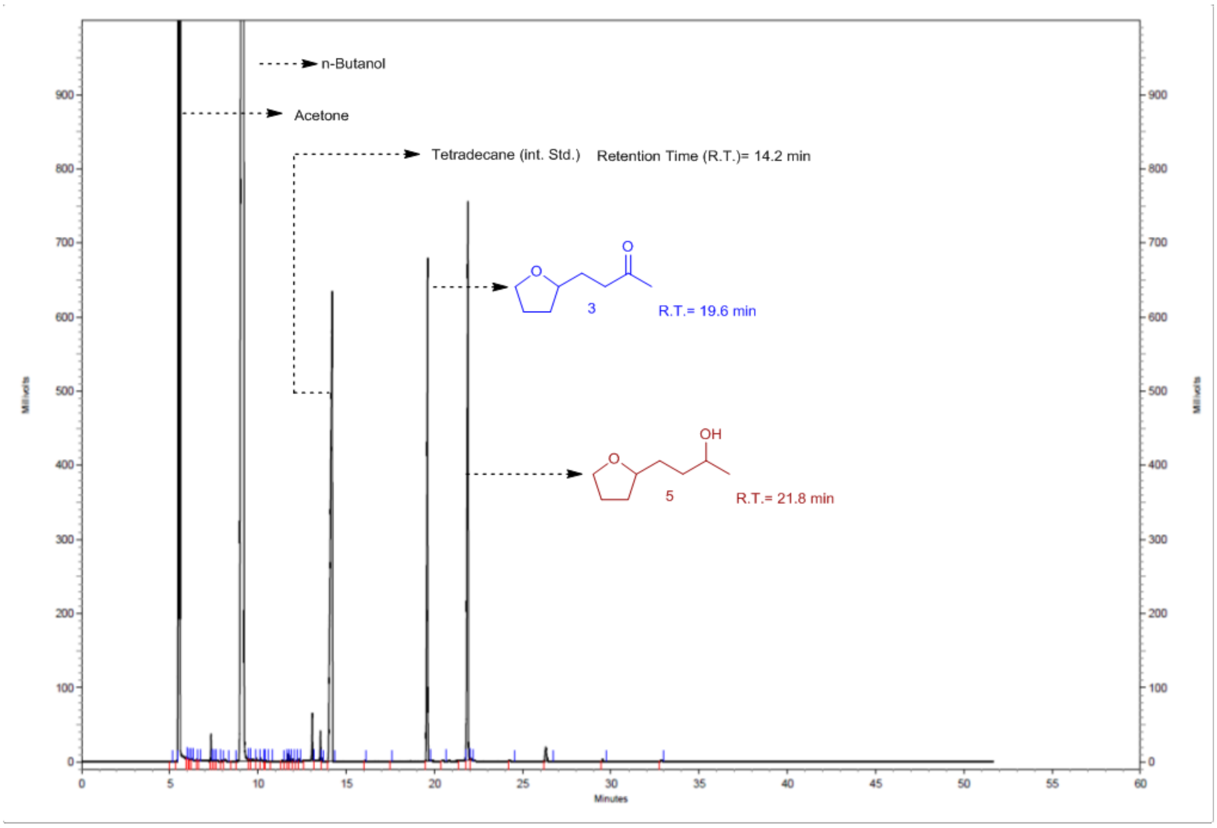

Supplementary Figure 22: Chromatogram corresponding to Figure 3c (Ru@PGS, H_2, 4 h).

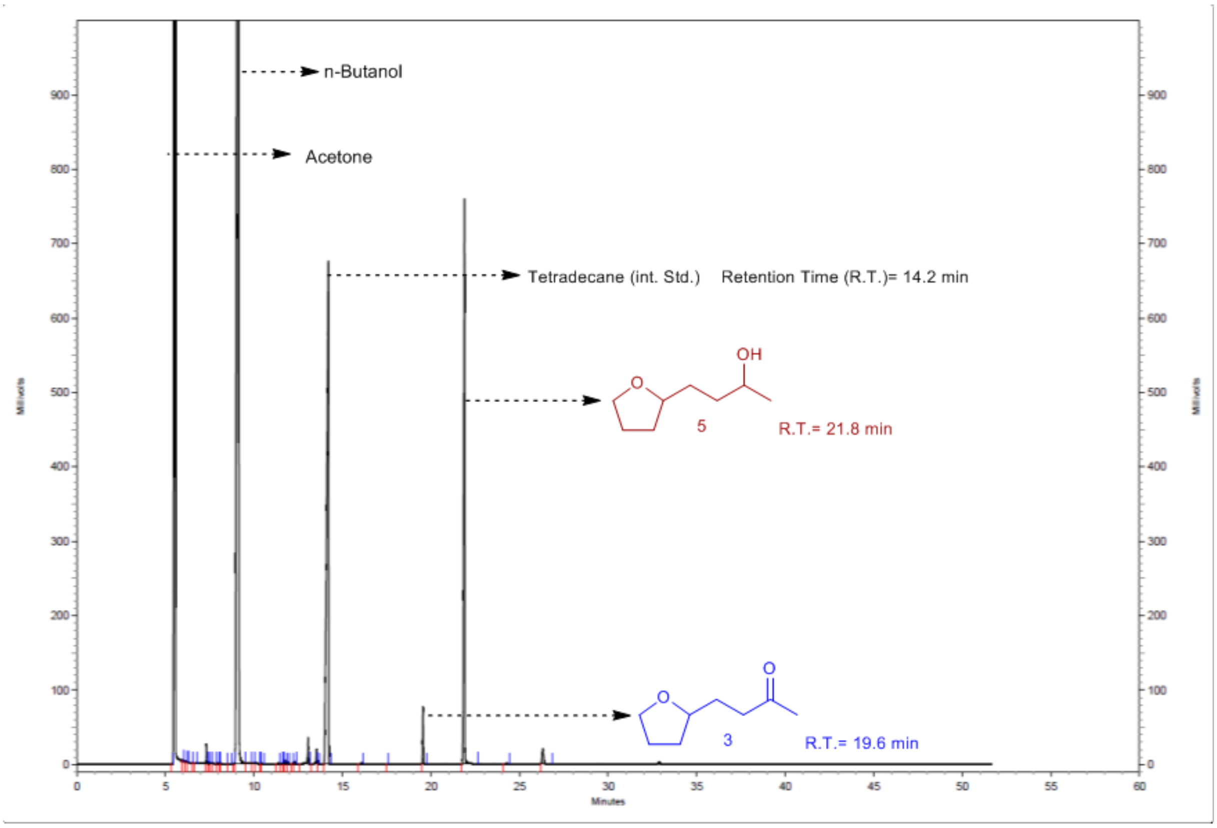

Supplementary Figure 23: Chromatogram corresponding to Figure 3c (Ru@PGS, H_2, 8 h).

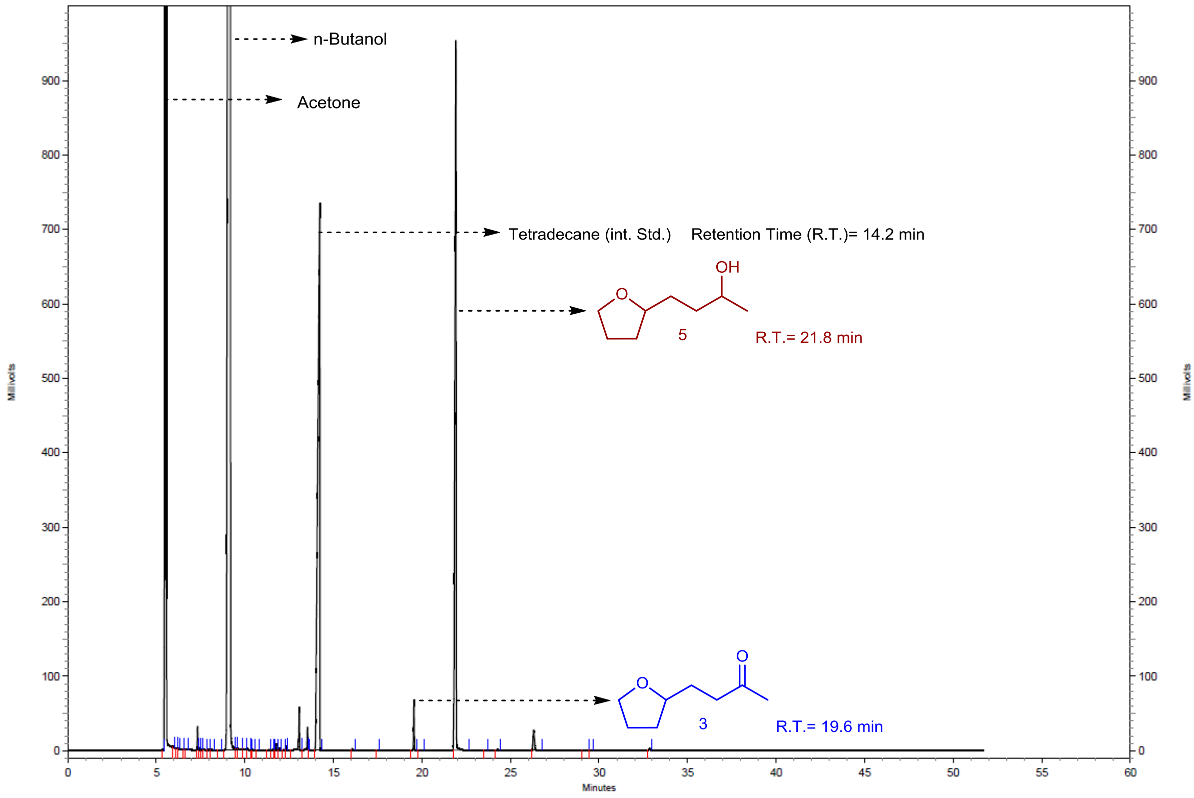

Supplementary Figure 24: Chromatogram corresponding to Figure 3c (Ru@PGS, H_2, 12 h).

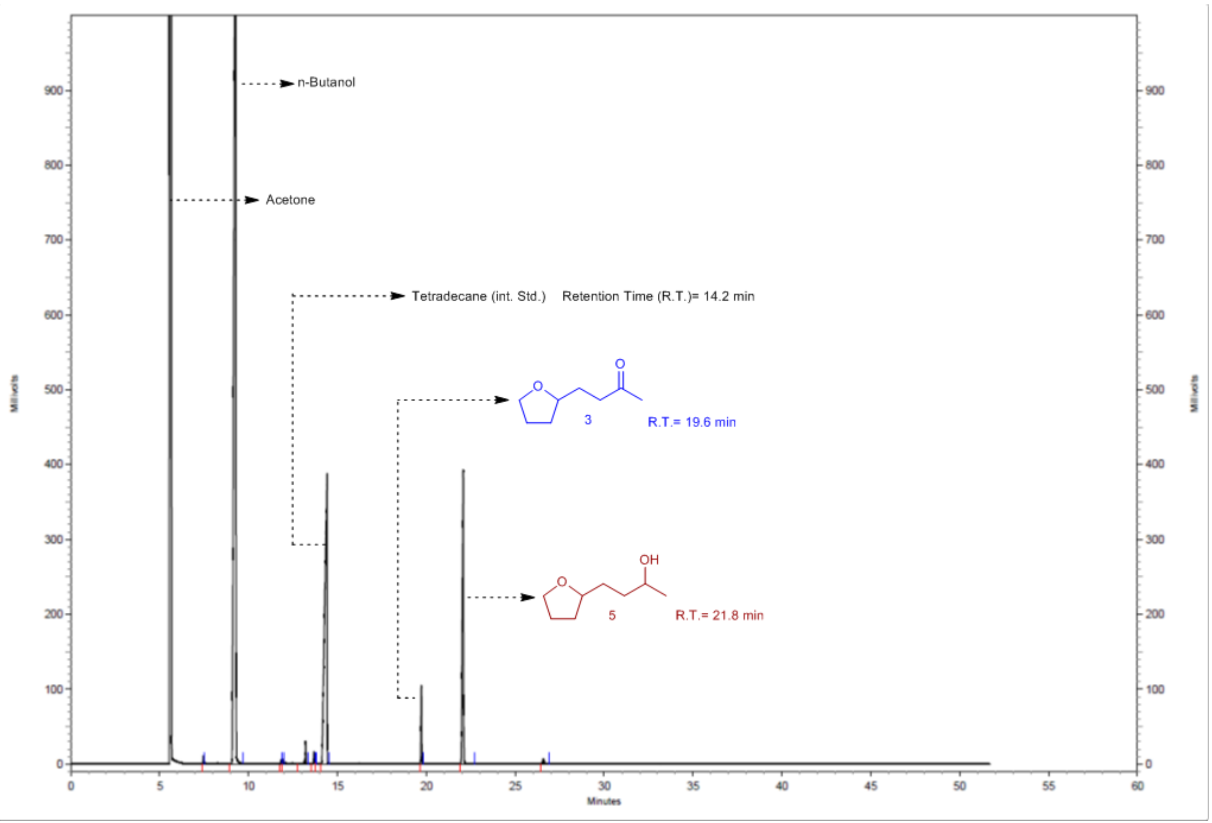

Supplementary Figure 25: Chromatogram corresponding to Figure 3c (Ru@PGS, H_2, 16 h).

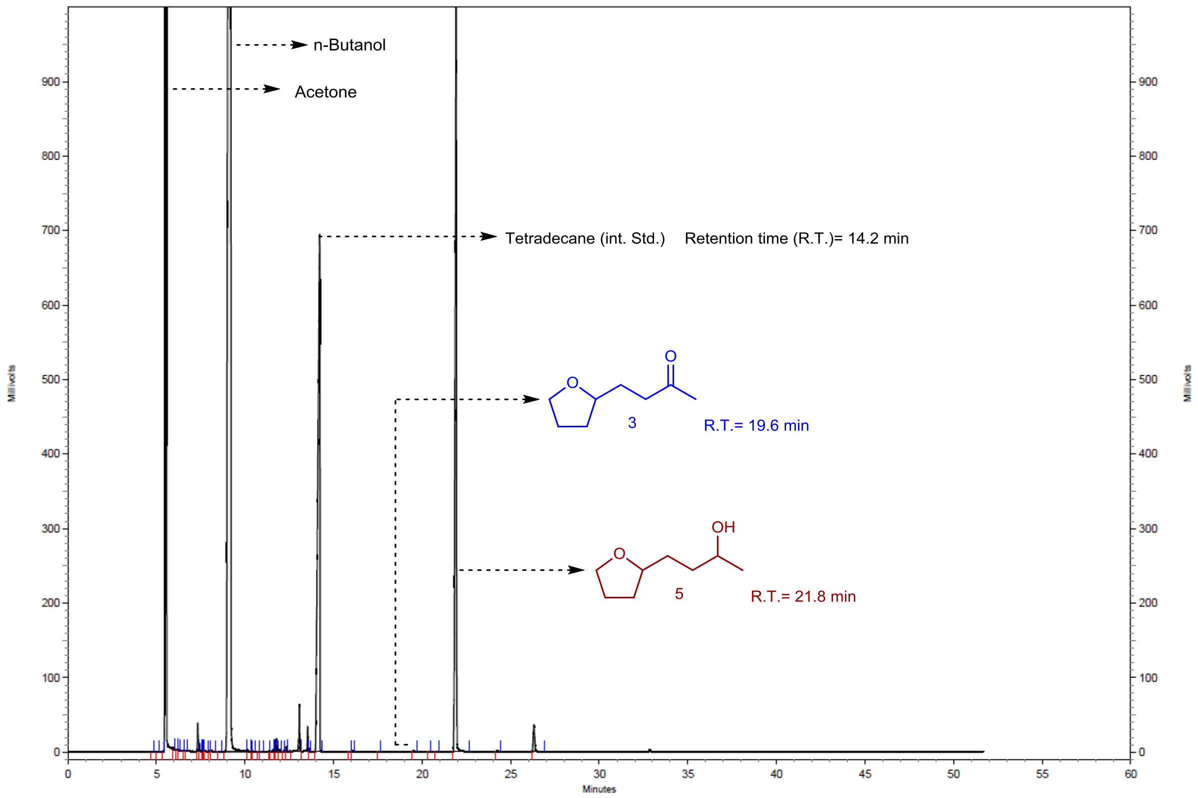

Supplementary Figure 26: Chromatogram corresponding to Figure 3c (Ru@PGS, H_2, 32 h).

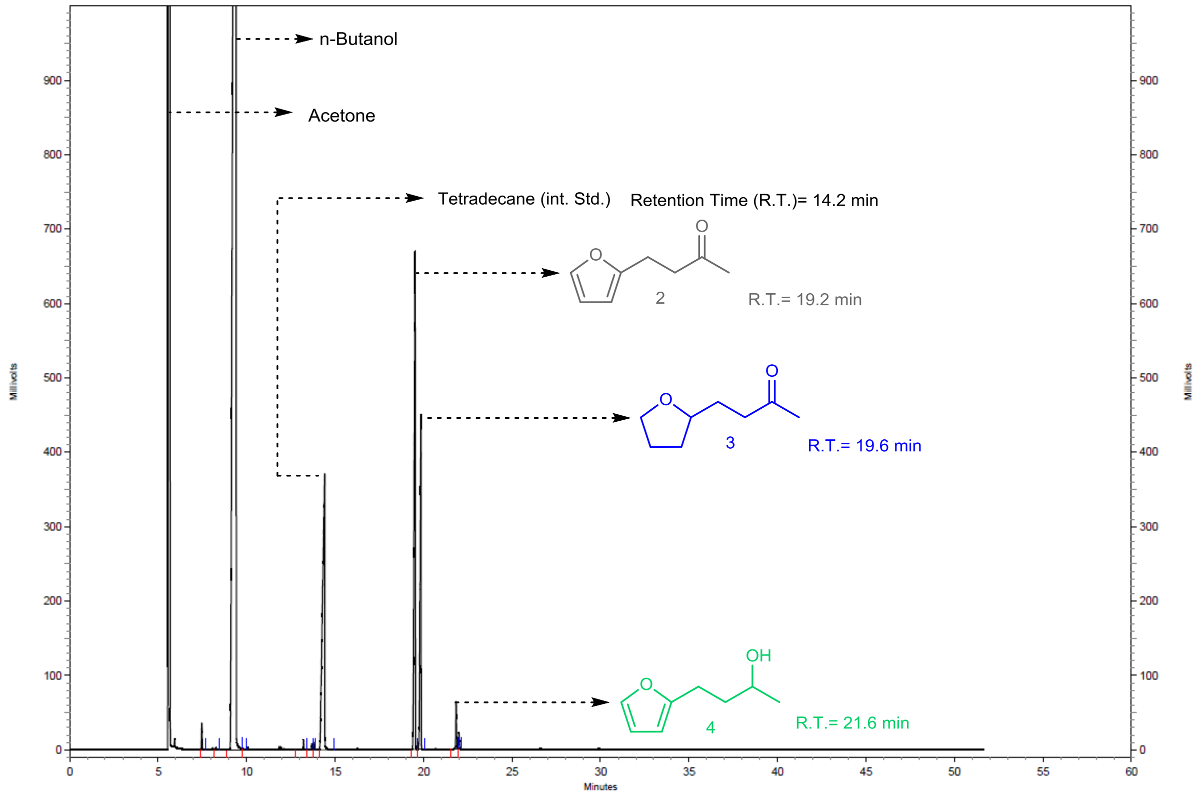

Supplementary Figure 27: Chromatogram corresponding to Figure 3d (Ru@PGS, H_2/CO_2, 1 h).

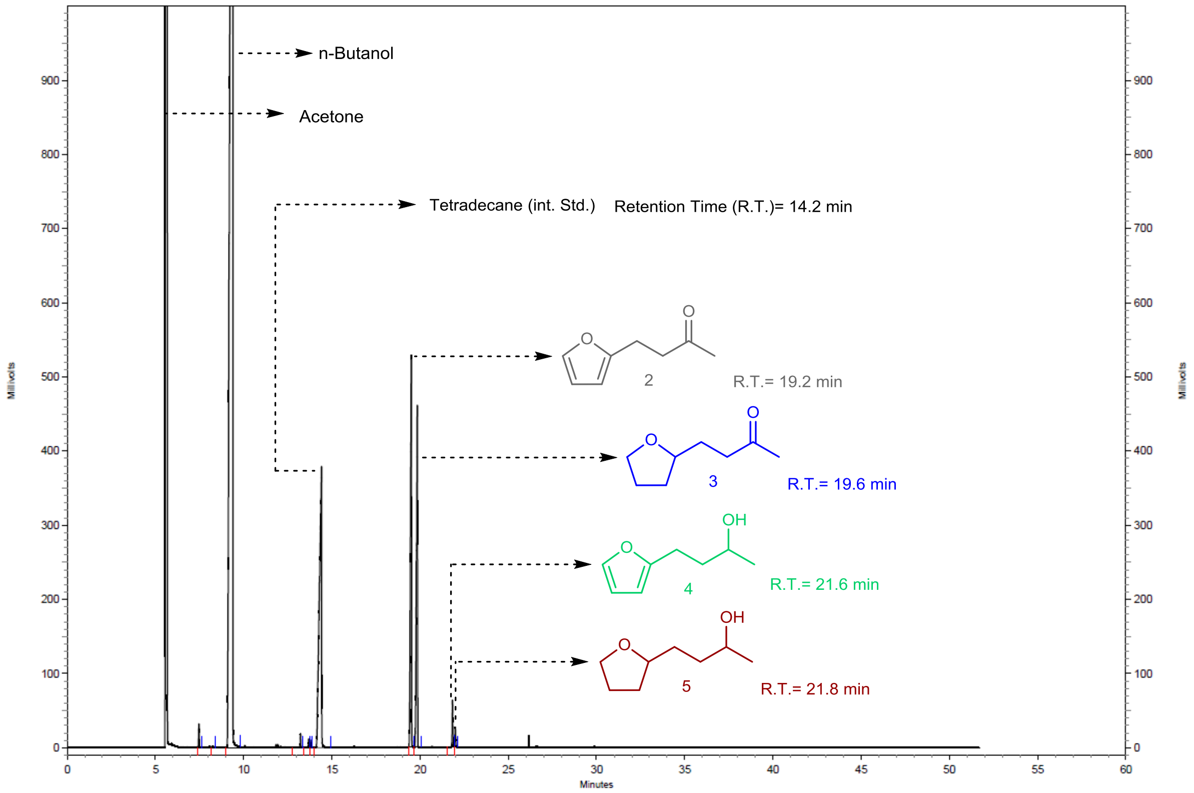

Supplementary Figure 28: Chromatogram corresponding to Figure 3d (Ru@PGS, H_2/CO_2, 2 h).

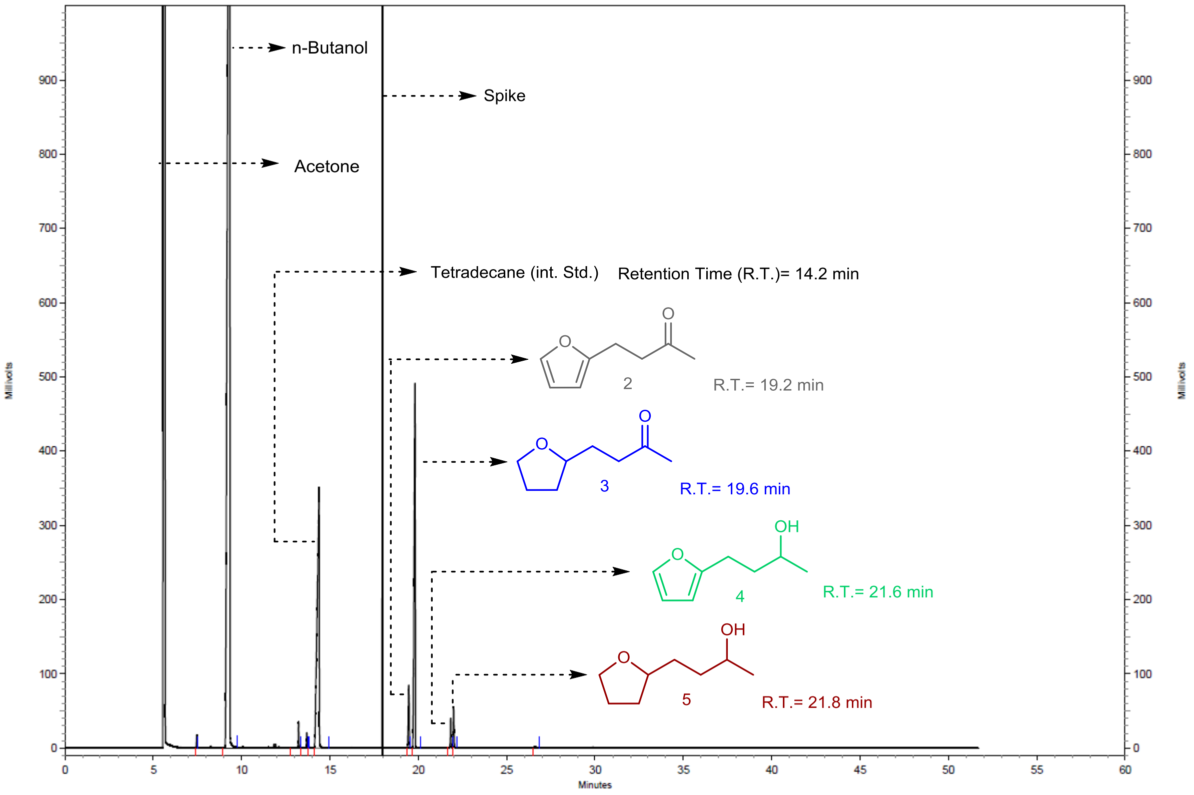

Supplementary Figure 29: Chromatogram corresponding to Figure 3d (Ru@PGS, H_2/CO_2, 4 h).

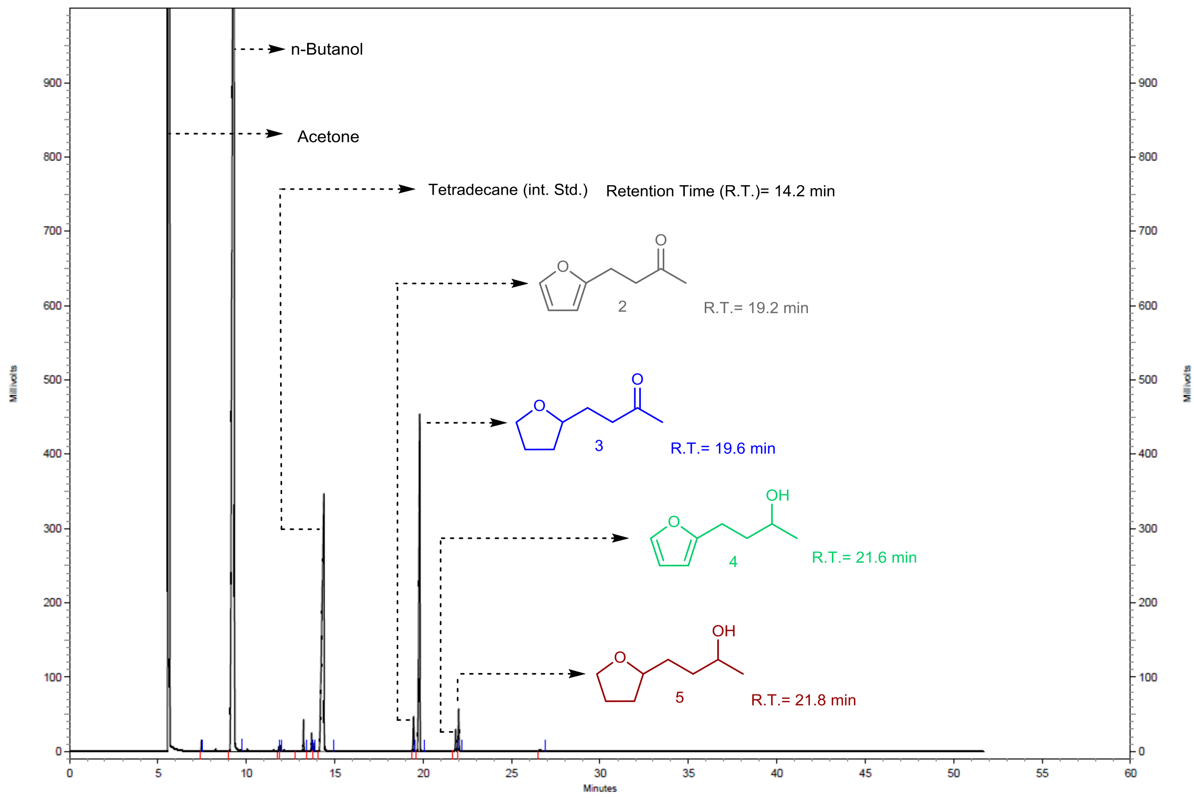

Supplementary Figure 30: Chromatogram corresponding to Figure 3d (Ru@PGS, H_2/CO_2, 8 h).

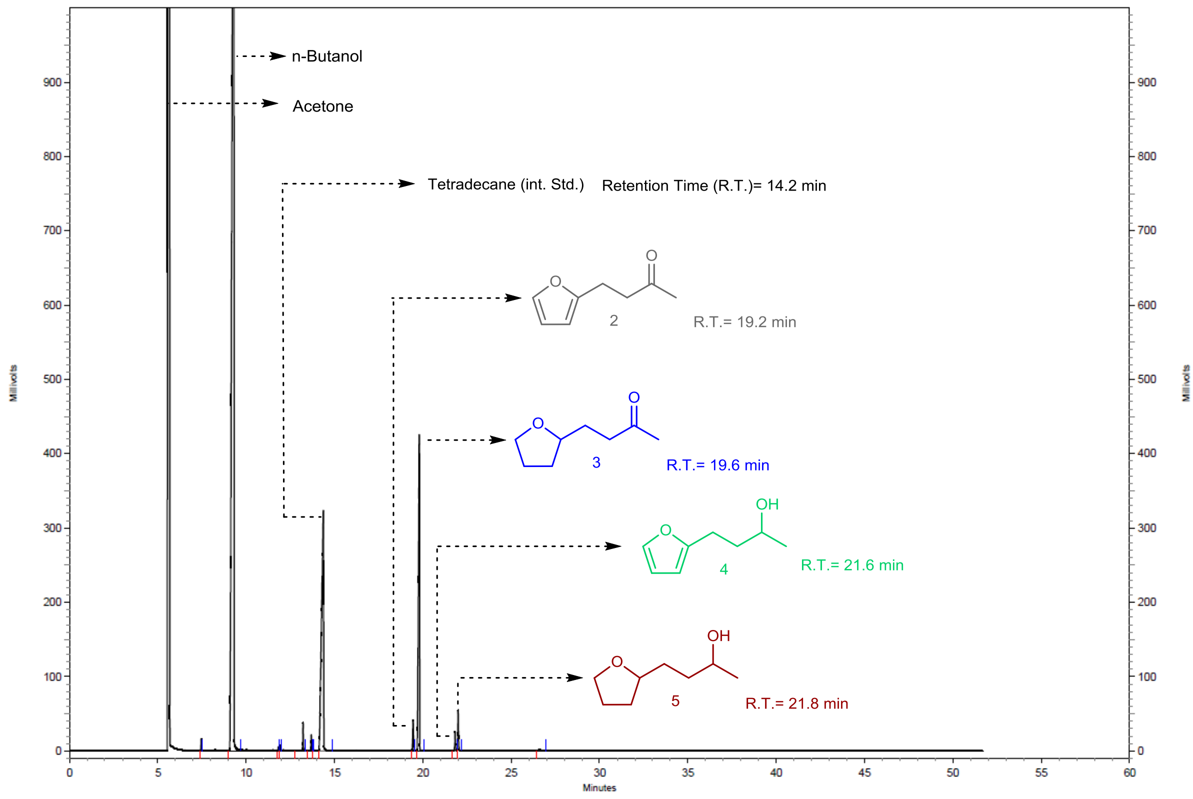

Supplementary Figure 31: Chromatogram corresponding to Figure 3d (Ru@PGS, H_2/CO_2, 12 h).

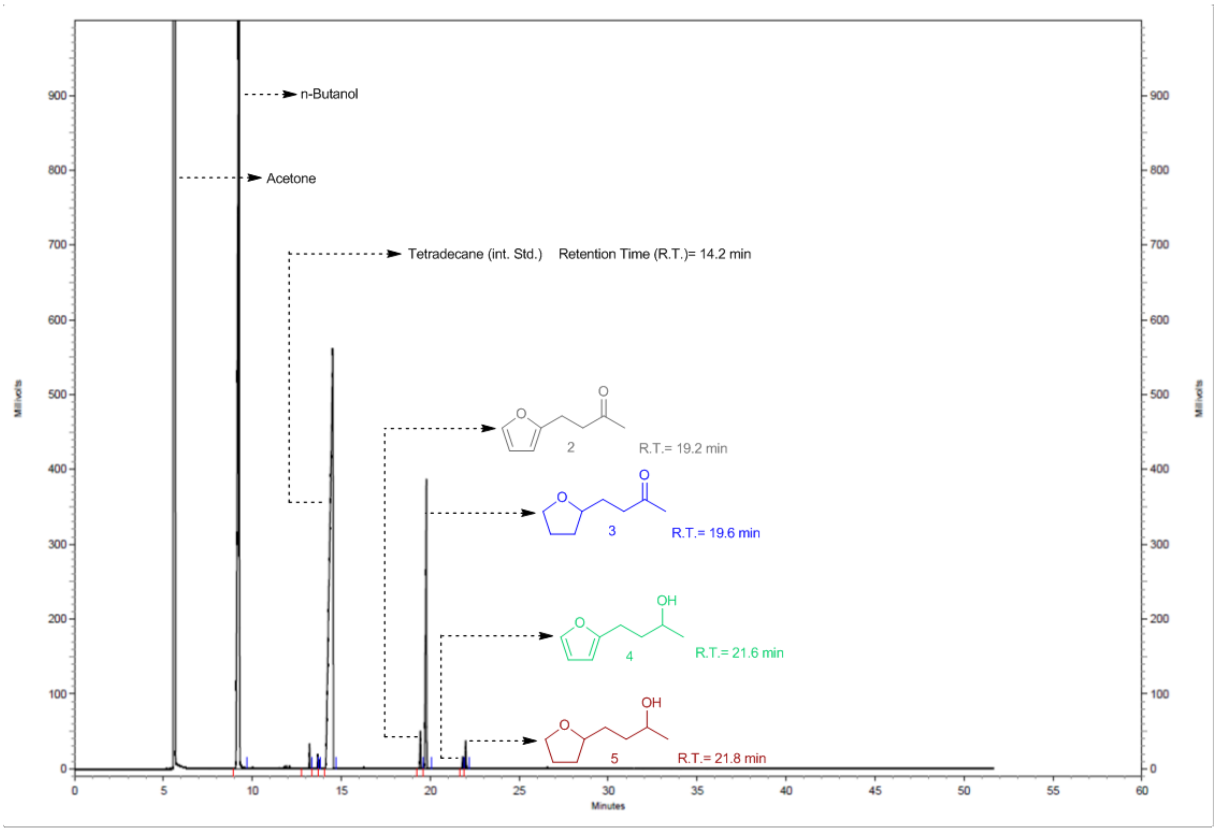

Supplementary Figure 32: Chromatogram corresponding to Figure 3d (Ru@PGS, H_2/CO_2, 16 h).

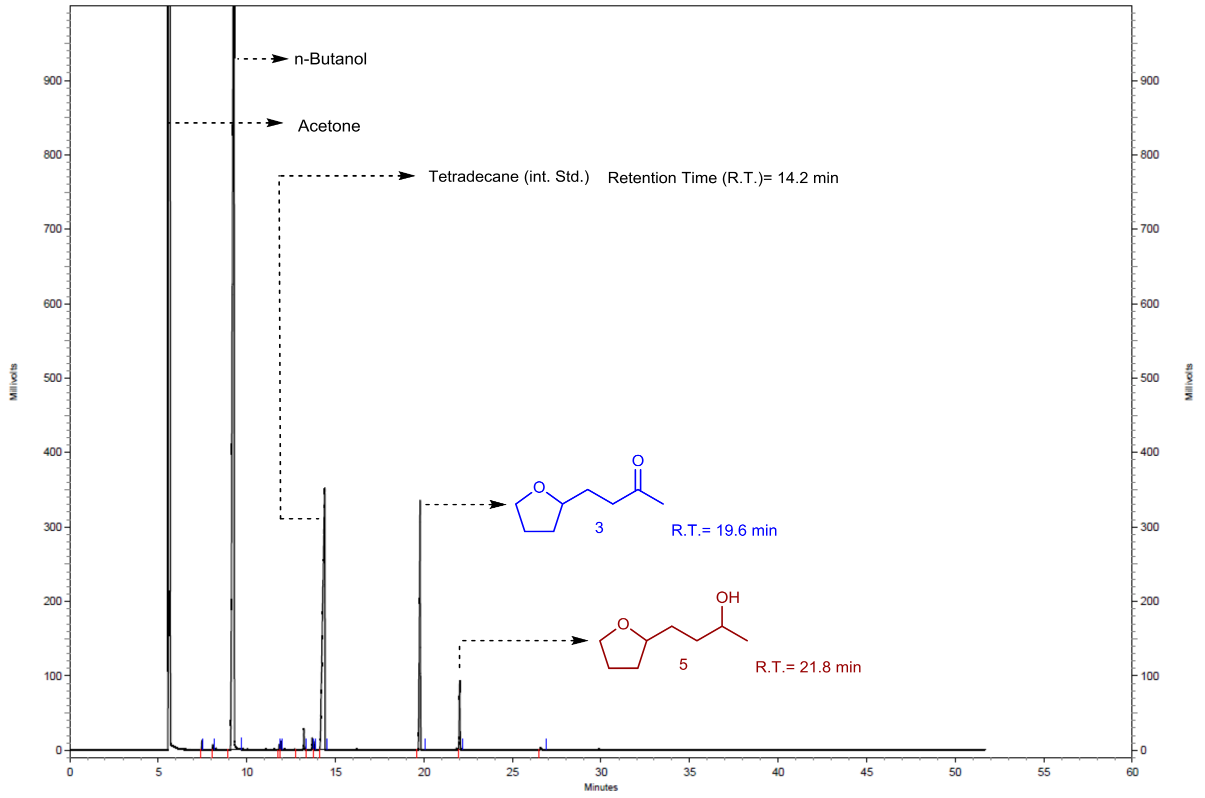

Supplementary Figure 33: Chromatogram corresponding to Figure 3d (Ru@PGS, H_2/CO_2, 32 h).

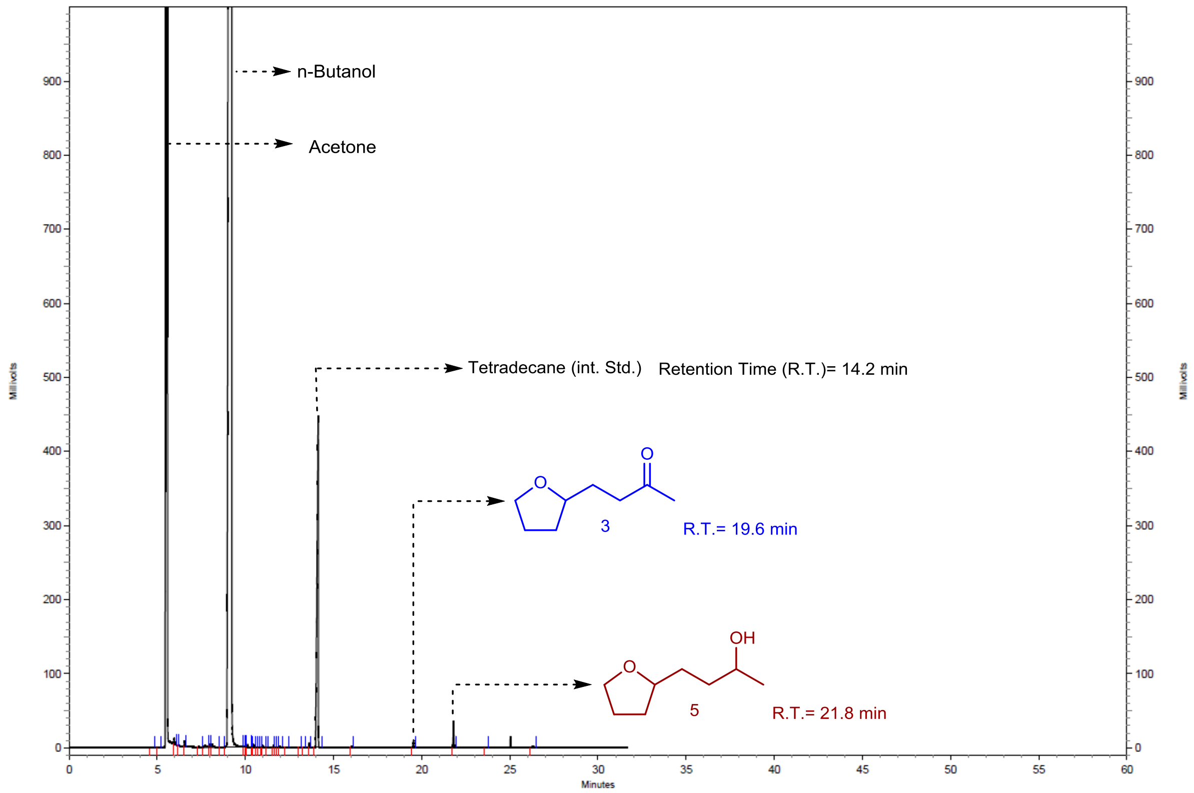

Supplementary Figure 34: Chromatogram corresponding to Figure 5b (switch 1, H_2).

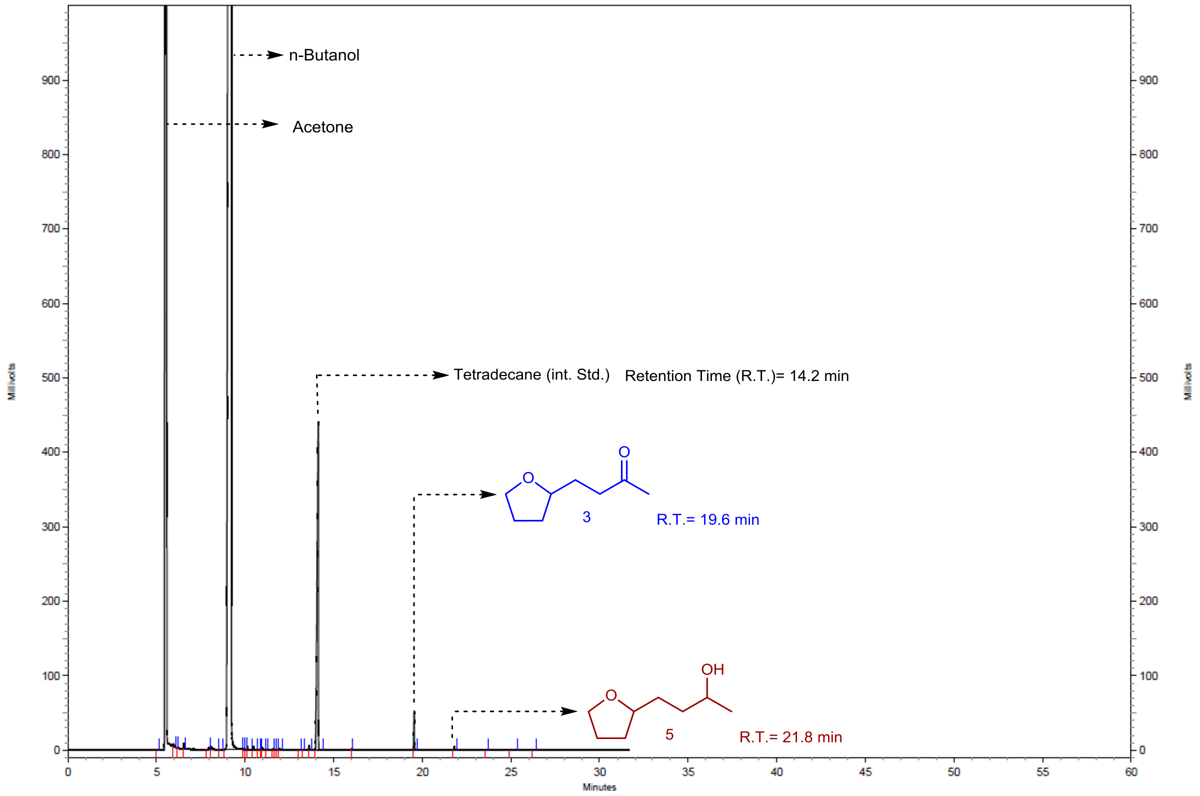

Supplementary Figure 35: Chromatogram corresponding to Figure 5b (switch 2, H_2/CO_2).

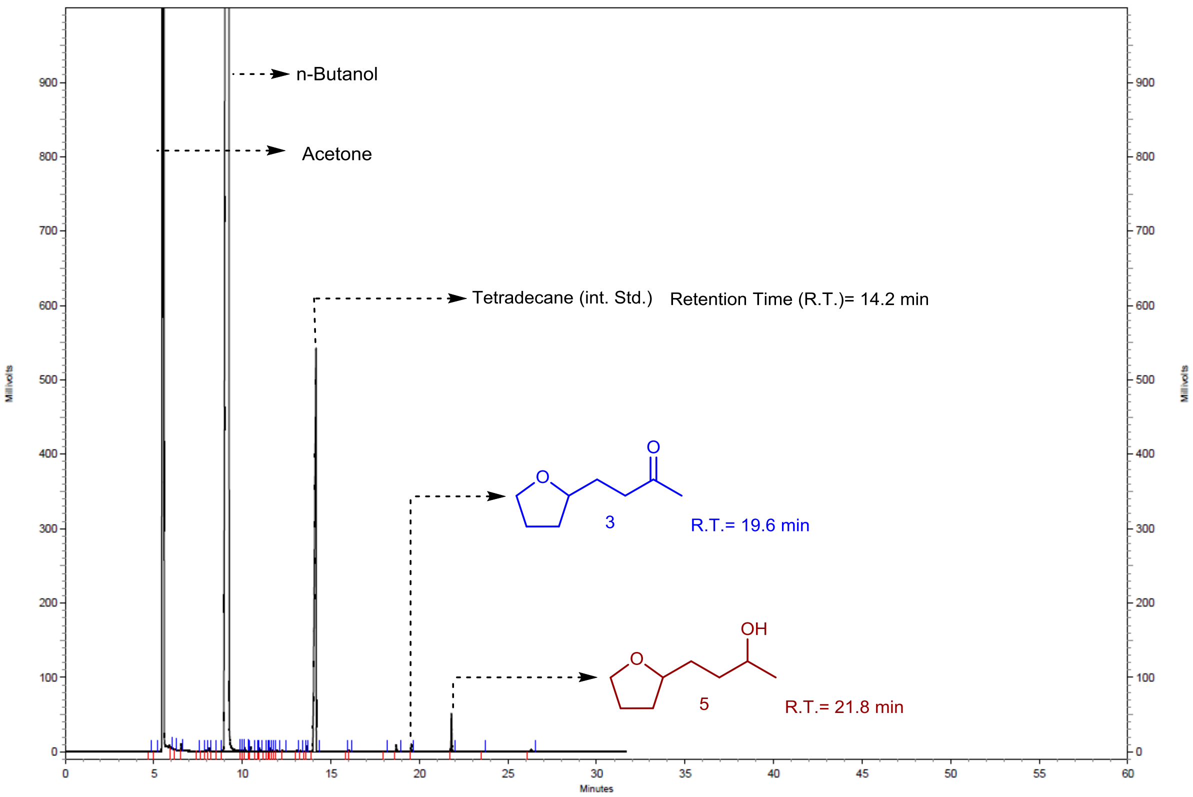

Supplementary Figure 36: Chromatogram corresponding to Figure 5b (switch 3, H_2).

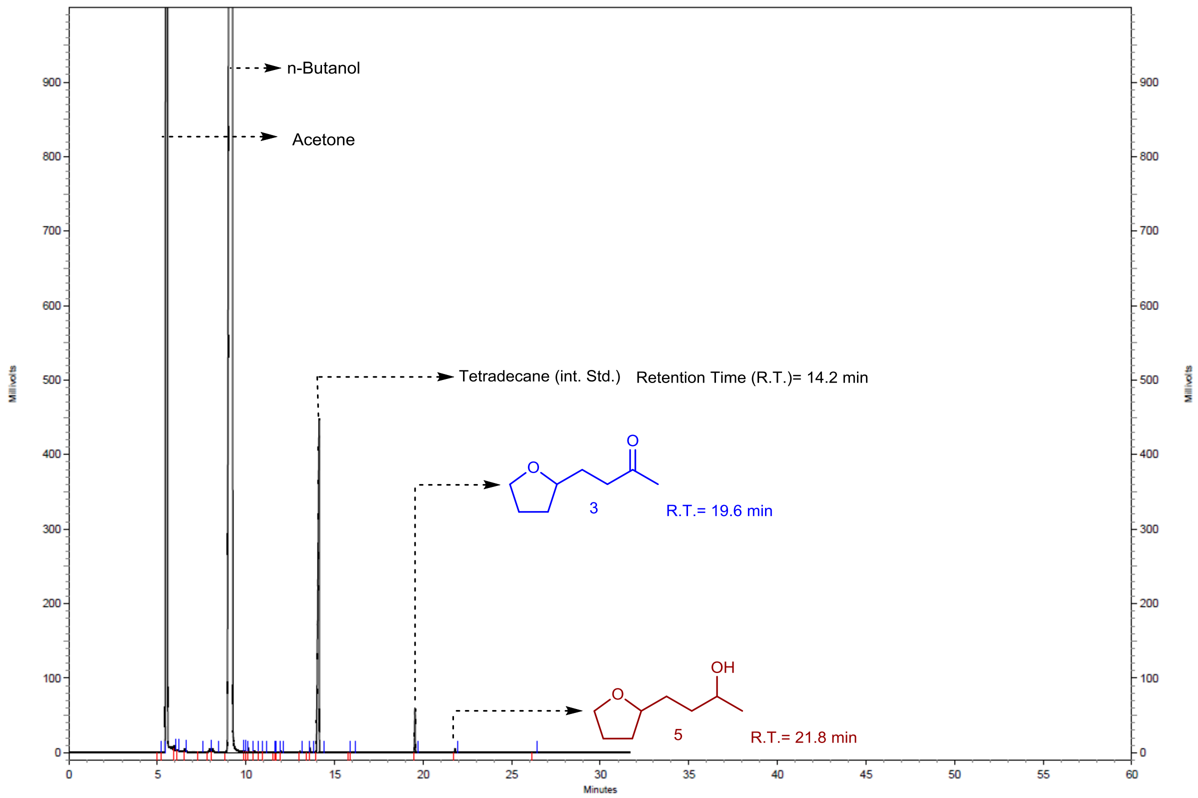

Supplementary Figure 37: Chromatogram corresponding to Figure 5b (switch 4, H_2/CO_2).

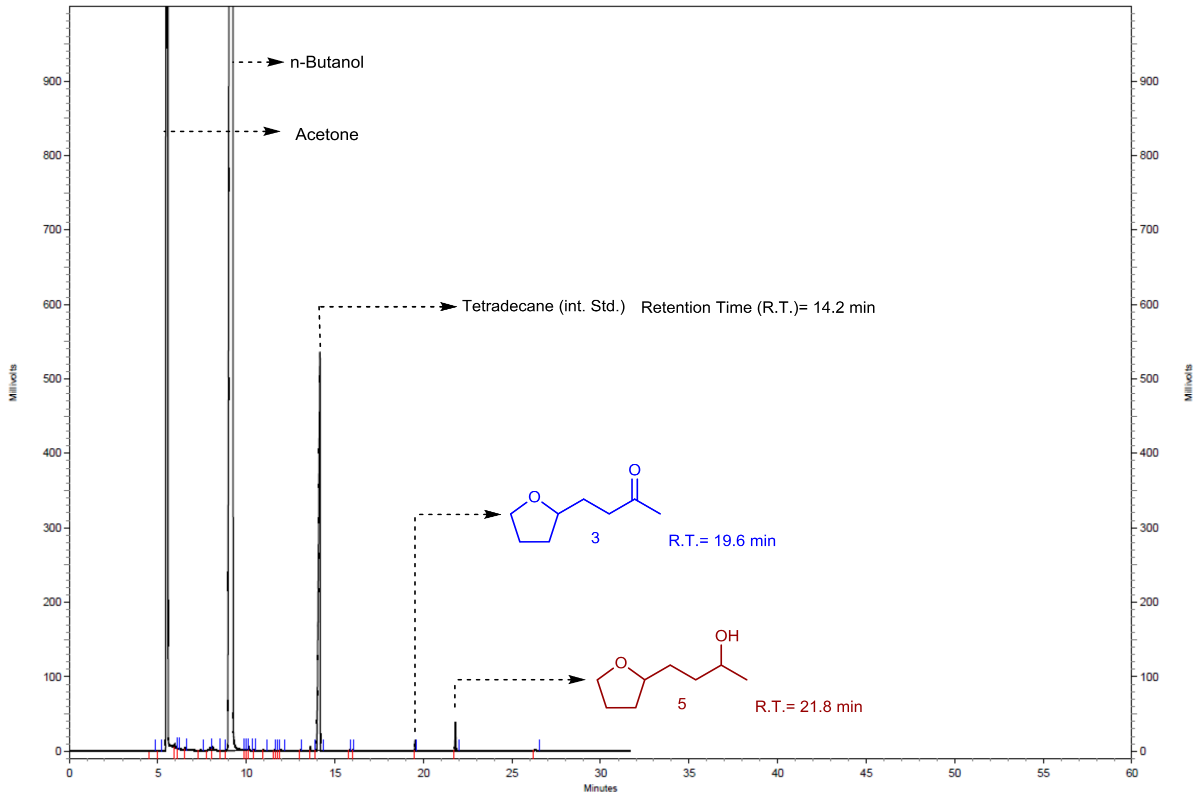

Supplementary Figure 38: Chromatogram corresponding to Figure 5b (switch 5, H_2).

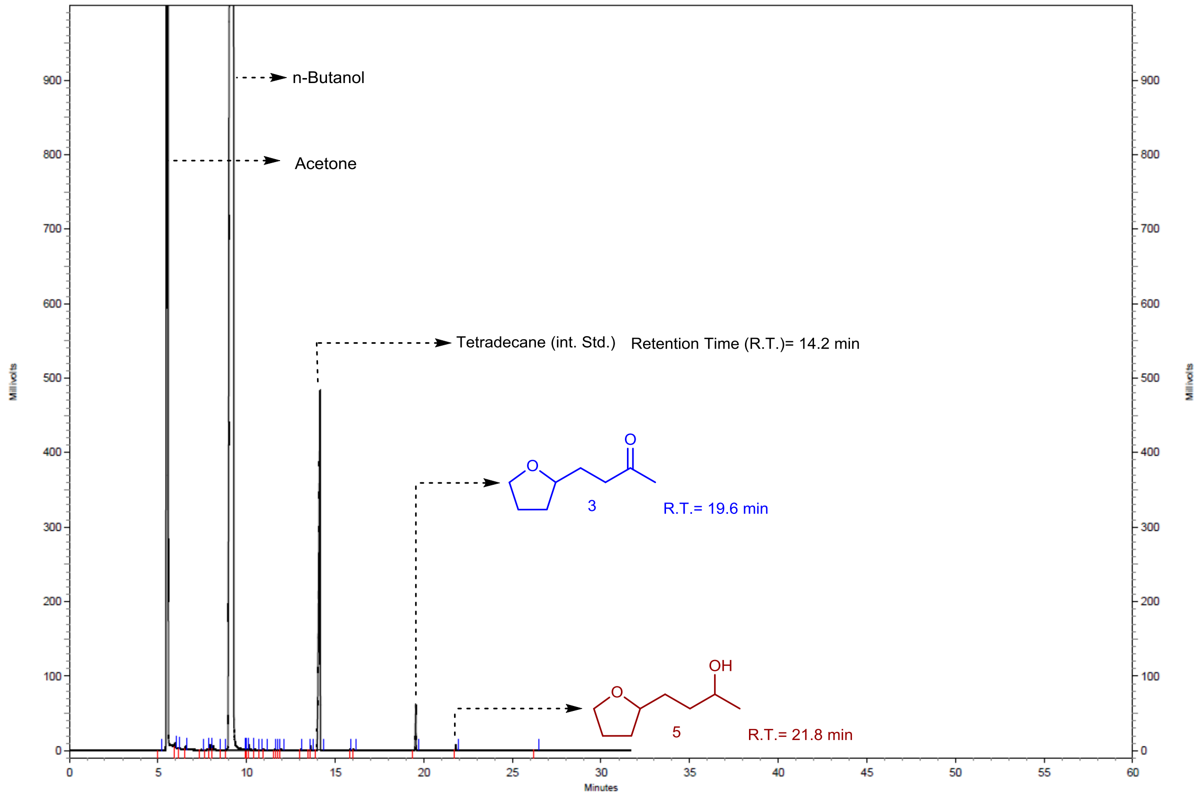

Supplementary Figure 39: Chromatogram corresponding to Figure 5b (switch 6, H_2/CO_2).

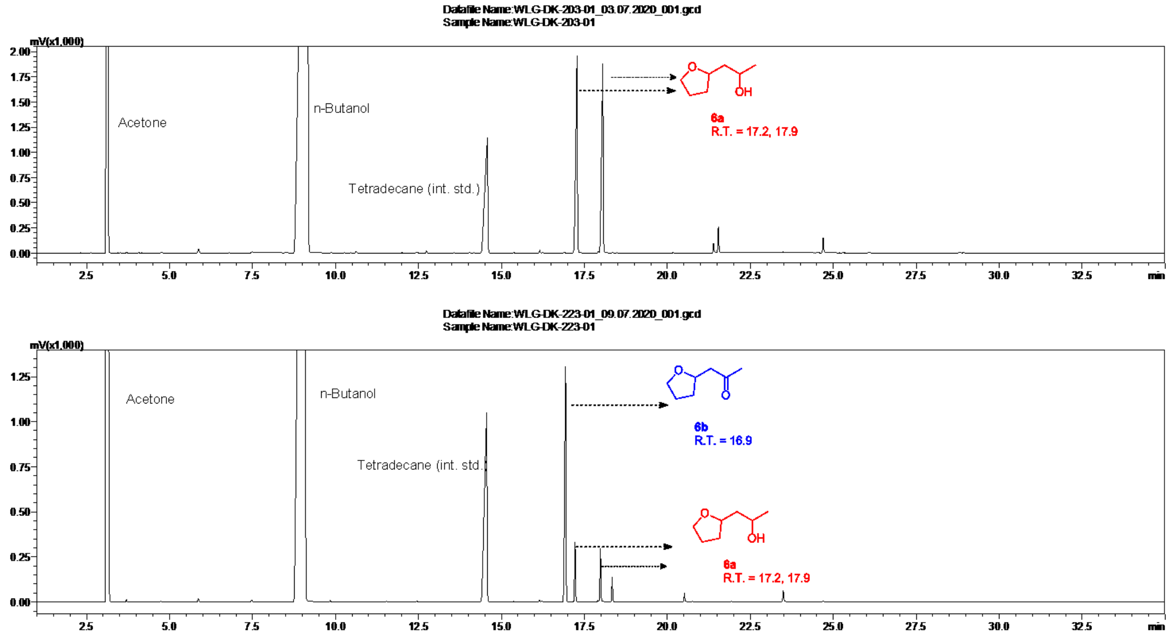

Supplementary Figure 40: Chromatograms corresponding to Table 1, Entry 1. Top: CO_2 switched OFF; bottom: CO_2 switched ON.

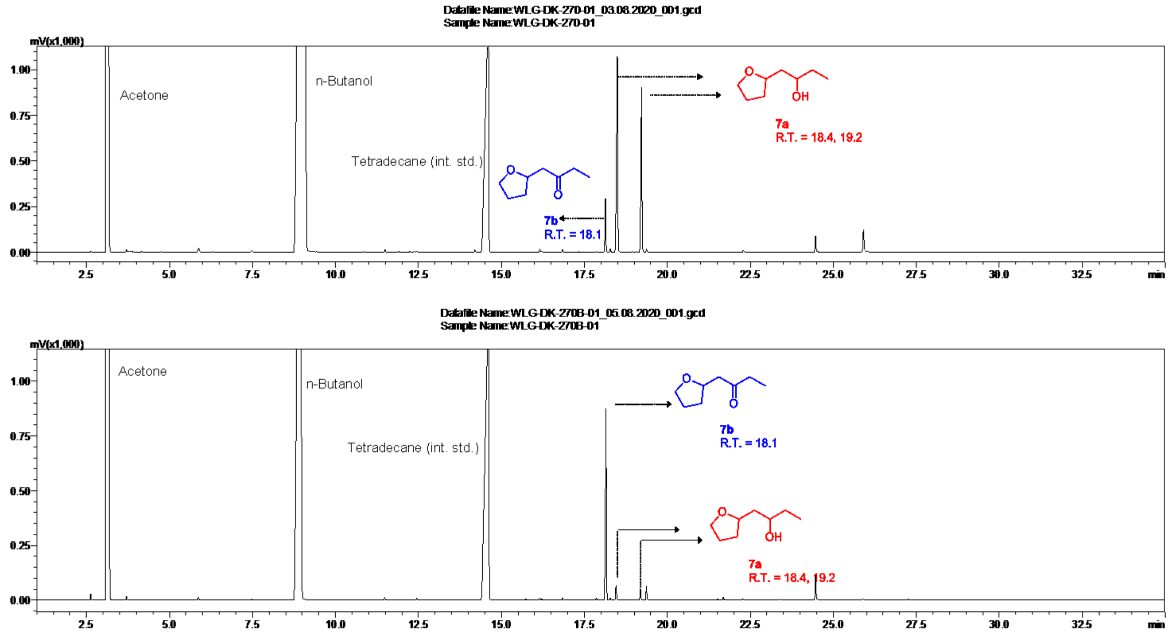

Supplementary Figure 41: Chromatograms corresponding to Table 1, Entry 2. Top: CO_2 switched OFF; bottom: CO_2 switched ON.

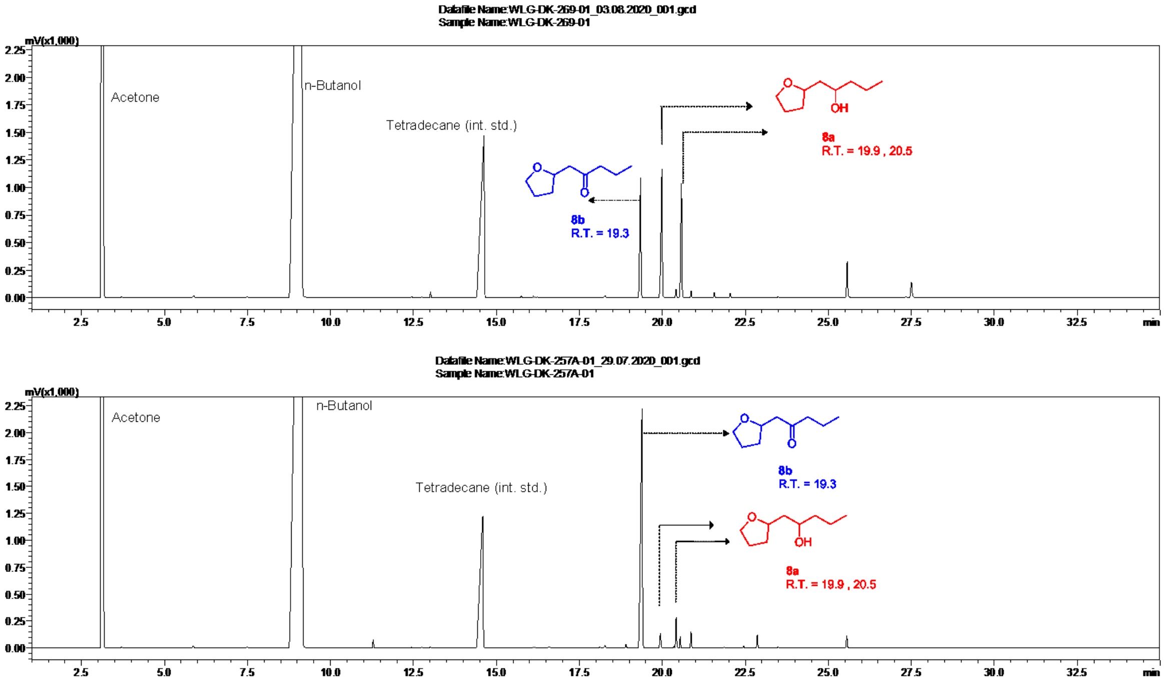

Supplementary Figure 42: Chromatograms corresponding to Table 1, Entry 3. Top: CO_2 switched OFF; bottom: CO_2 switched ON.

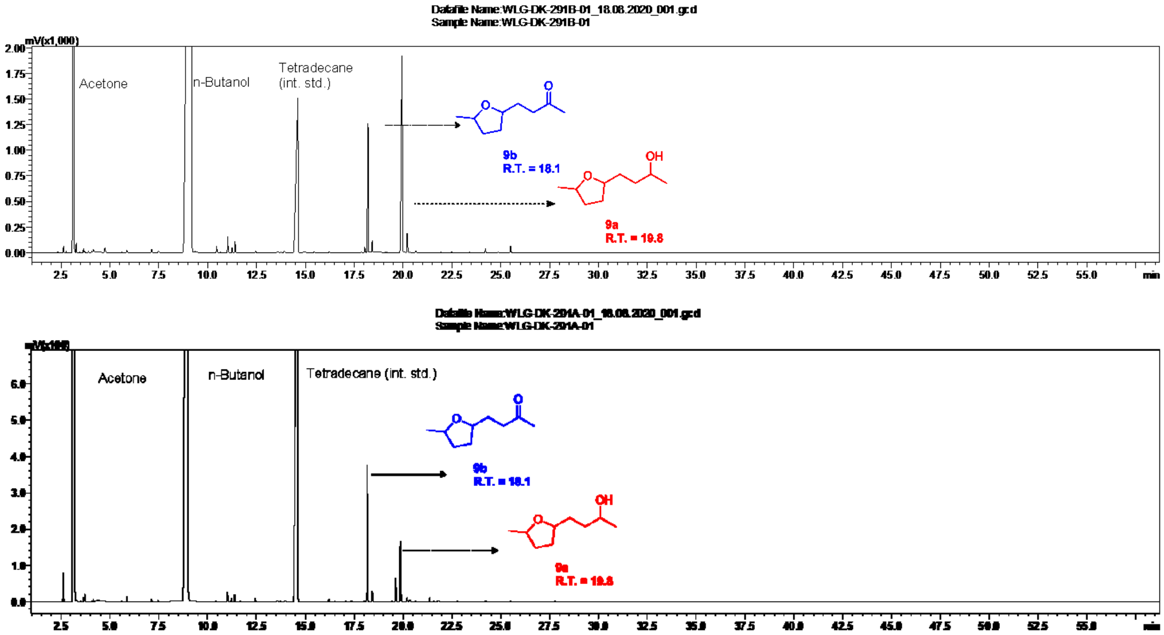

Supplementary Figure 43: Chromatograms corresponding to Table 1, Entry 4. Top: CO_2 switched OFF; bottom: CO_2 switched ON.

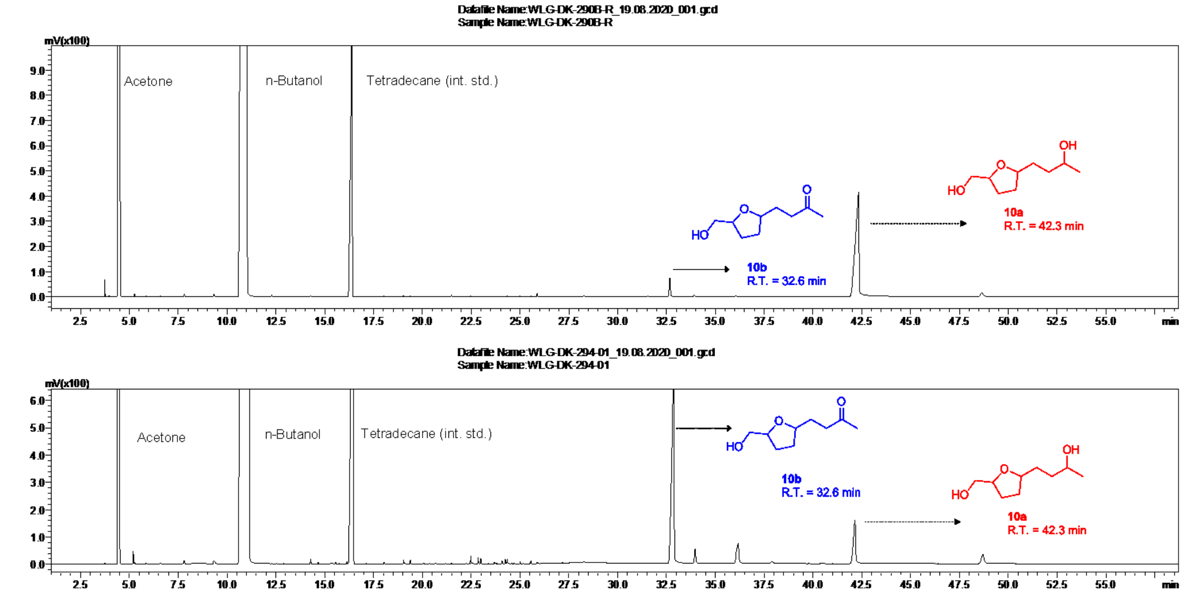

Supplementary Figure 44: Chromatograms corresponding to Table 1, Entry 5. Top: CO_2 switched OFF; bottom: CO_2 switched ON.

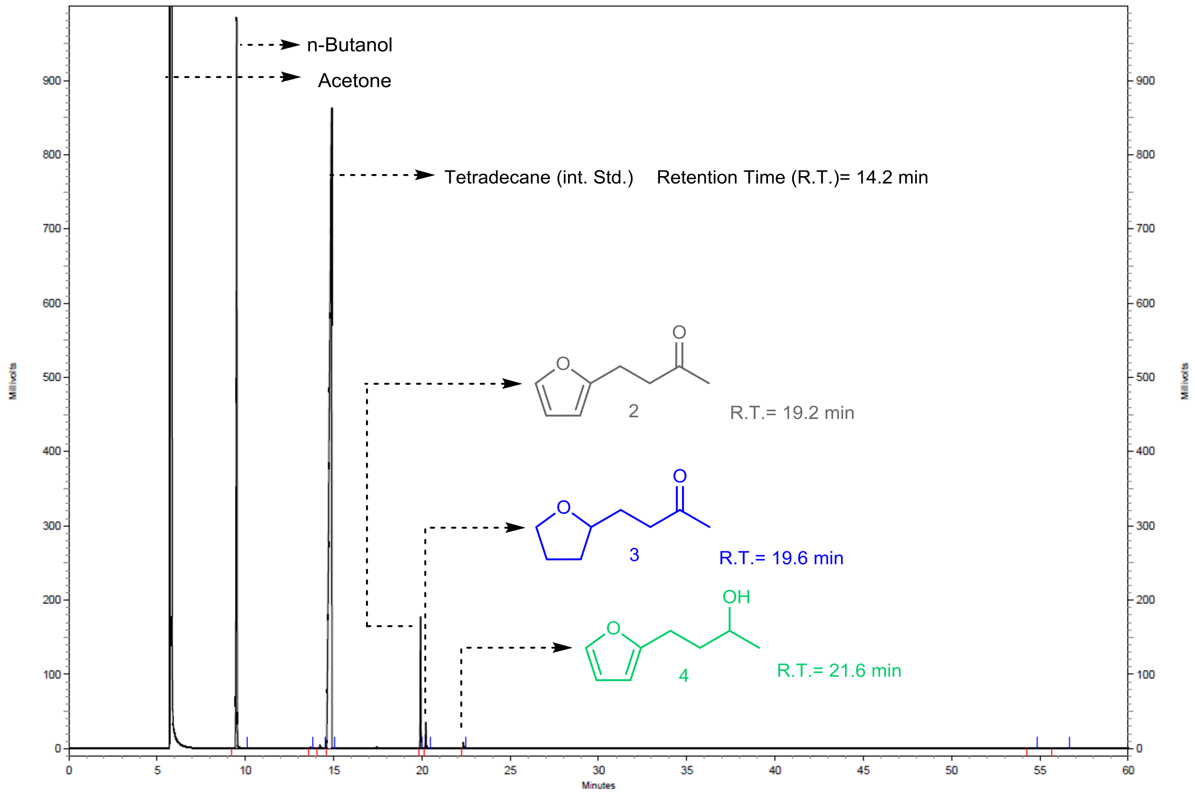

Supplementary Figure 45: Chromatogram corresponding to Supplementary Table 3, Entry 1.

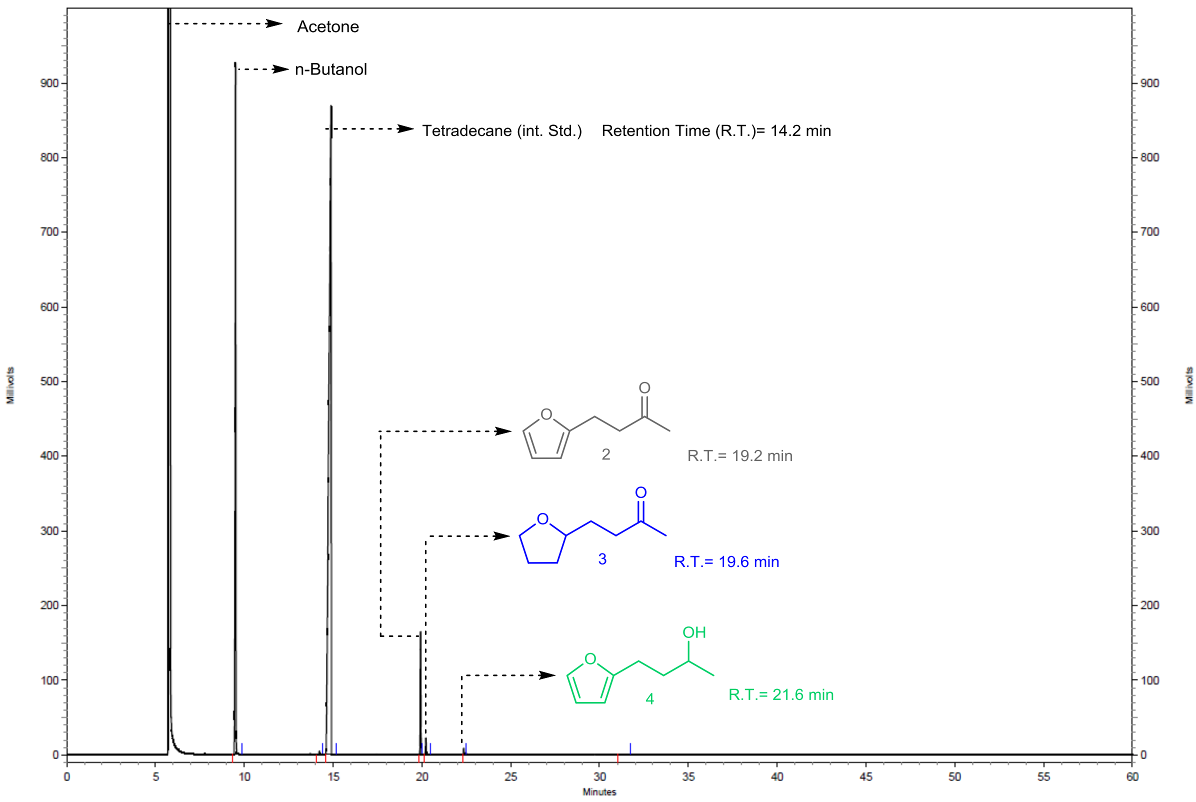

Supplementary Figure 46: Chromatogram corresponding to Supplementary Table 3, Entry 2.

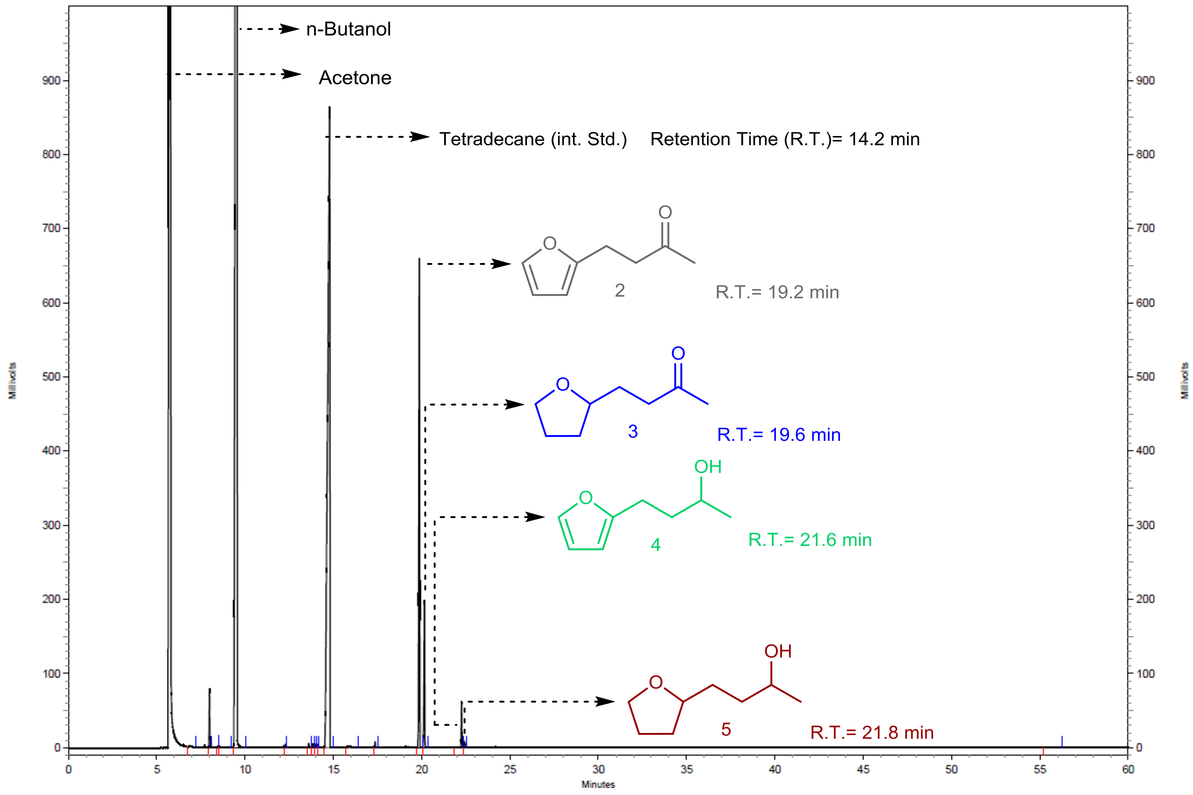

Supplementary Figure 47: Chromatogram corresponding to Supplementary Table 3, Entry 3.

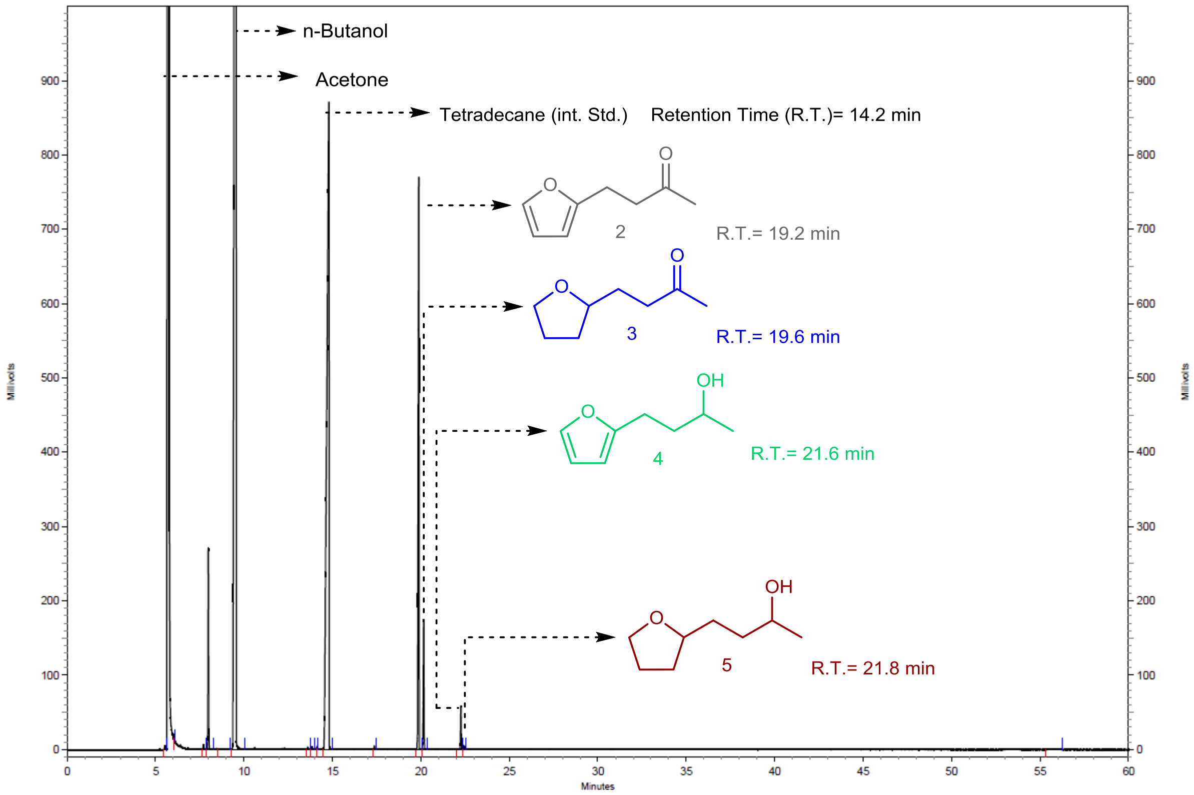

Supplementary Figure 48: Chromatogram corresponding to Supplementary Table 3, Entry 4.

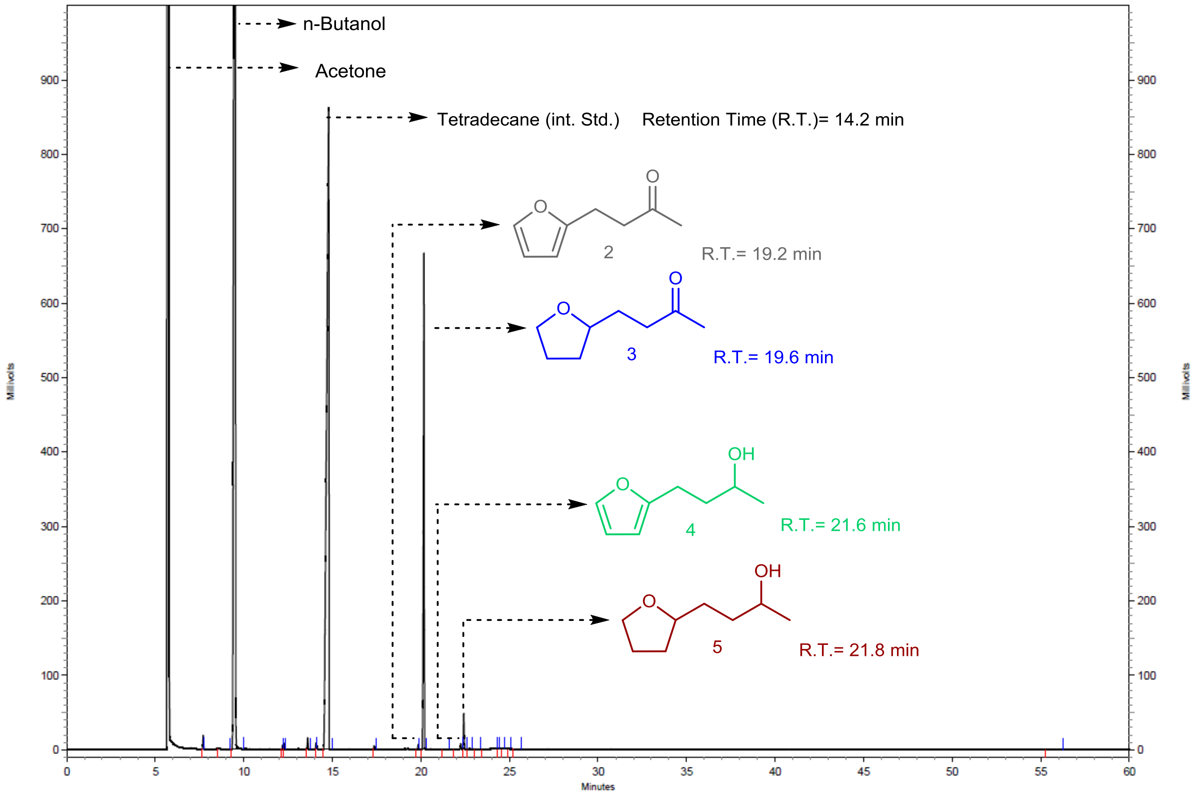

Supplementary Figure 49: Chromatogram corresponding to Supplementary Table 3, Entry 5.

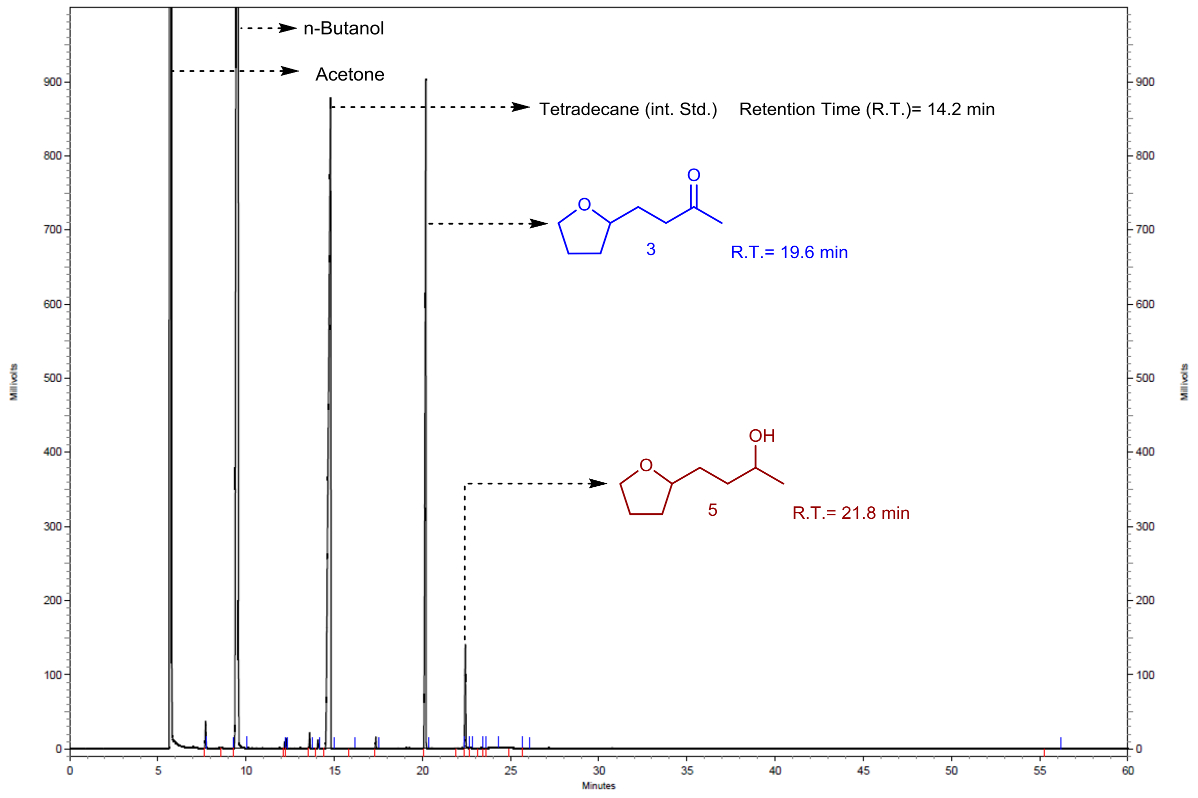

Supplementary Figure 50: Chromatogram corresponding to Supplementary Table 3, Entry 6.

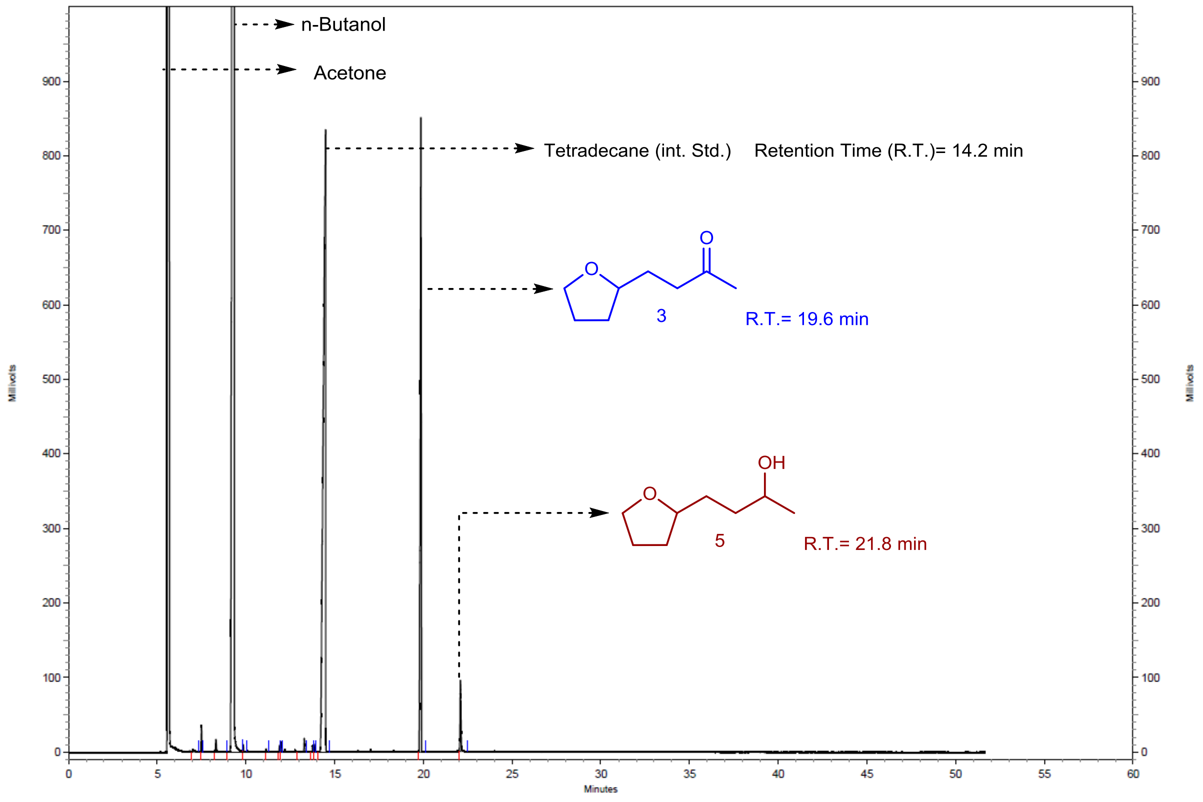

Supplementary Figure 51: Chromatogram corresponding to Supplementary Table 3, Entry 7.

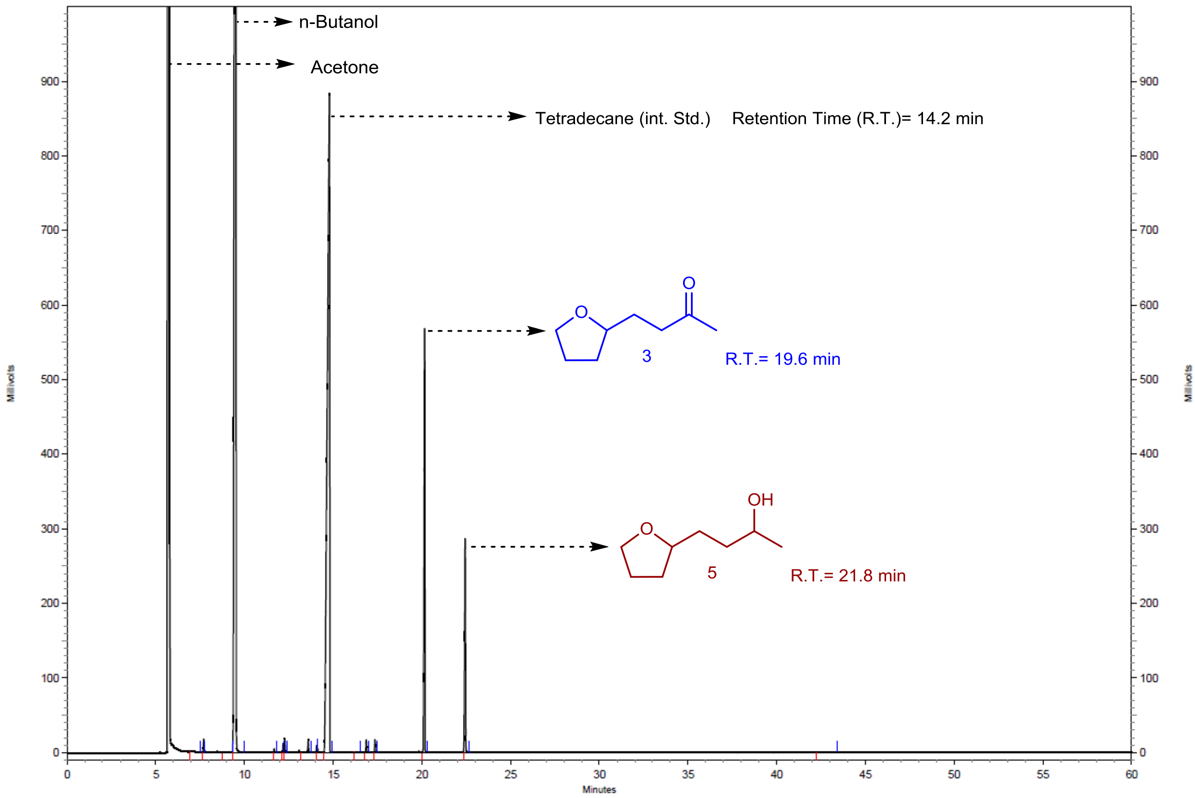

Supplementary Figure 52: Chromatogram corresponding to Supplementary Table 3, Entry 8.

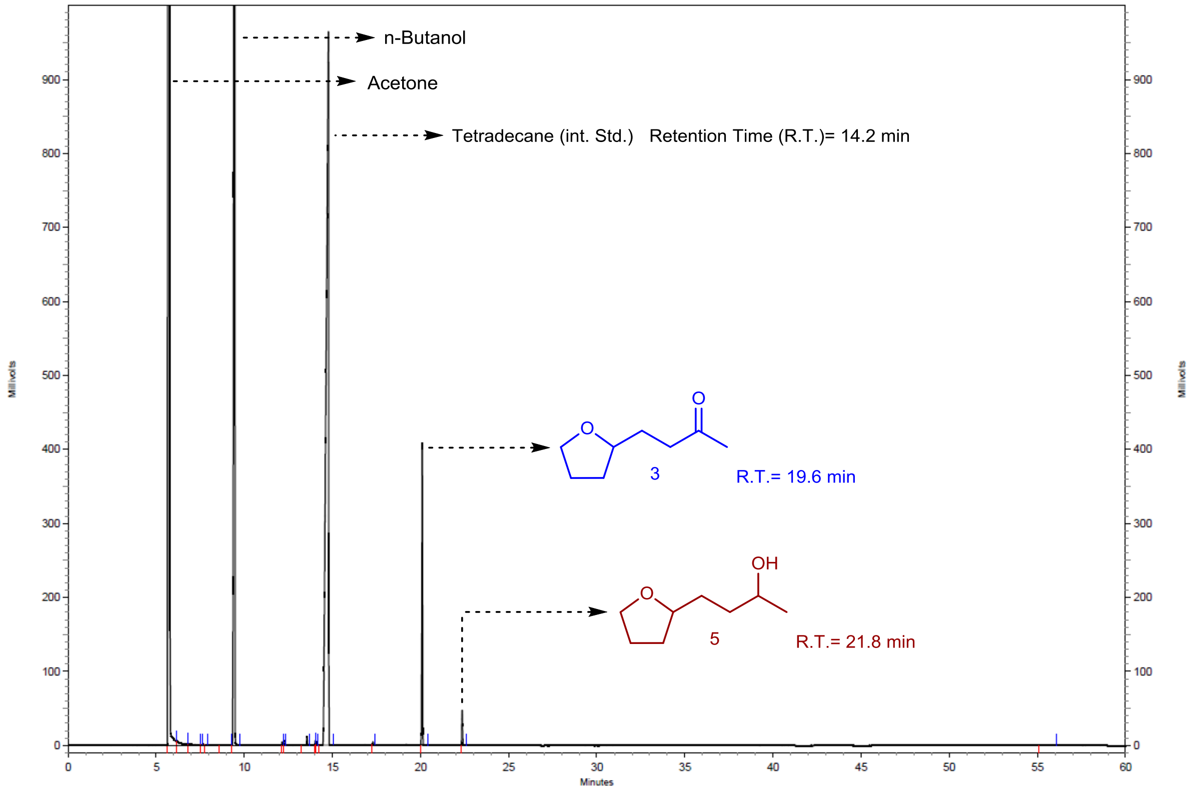

Supplementary Figure 53: Chromatogram corresponding to Supplementary Table 3, Entry 9.

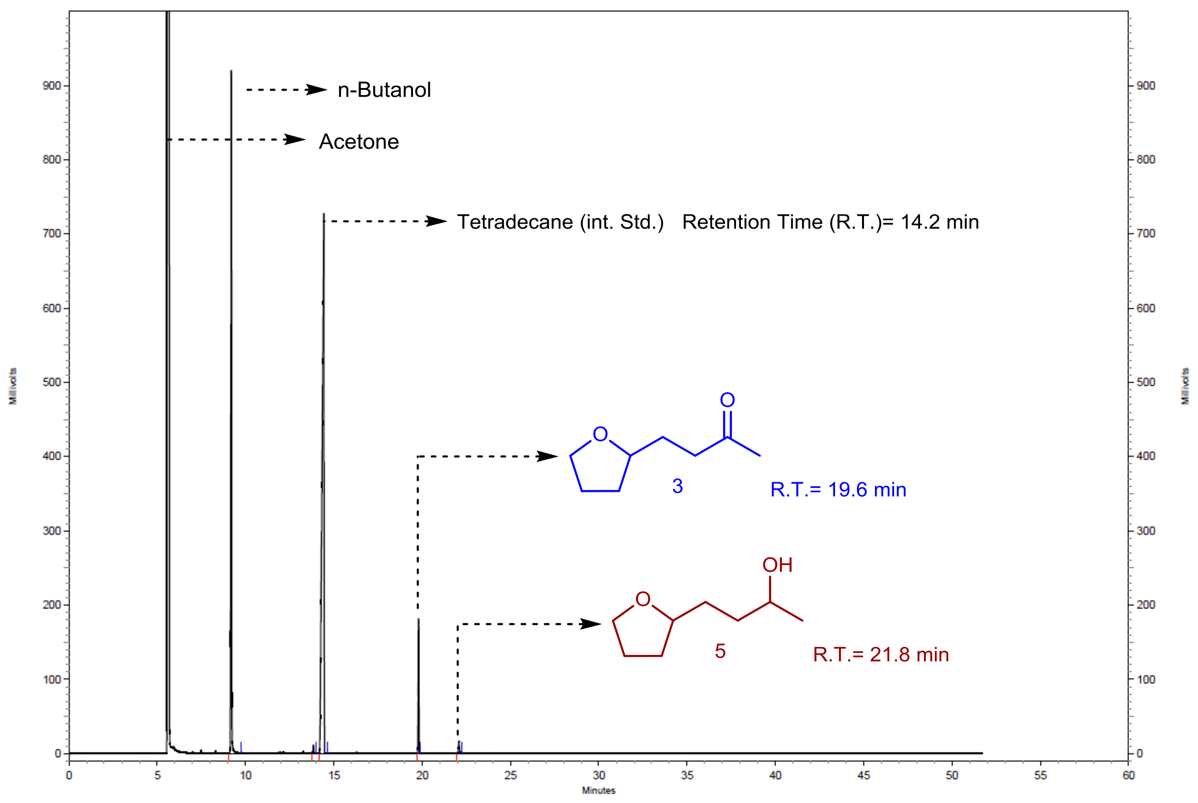

Supplementary Figure 54: Chromatogram corresponding to Supplementary Table 3, Entry 10.

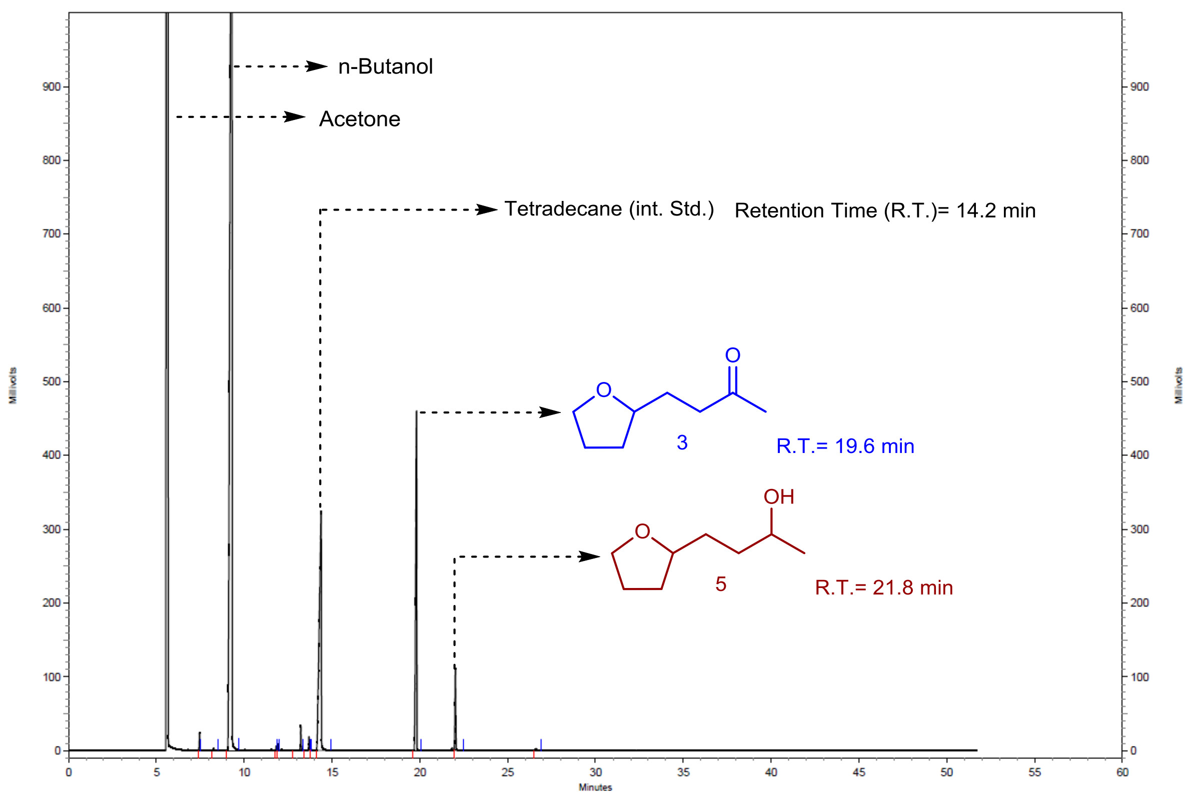

Supplementary Figure 55: Chromatogram corresponding to Supplementary Table 3, Entry 11.

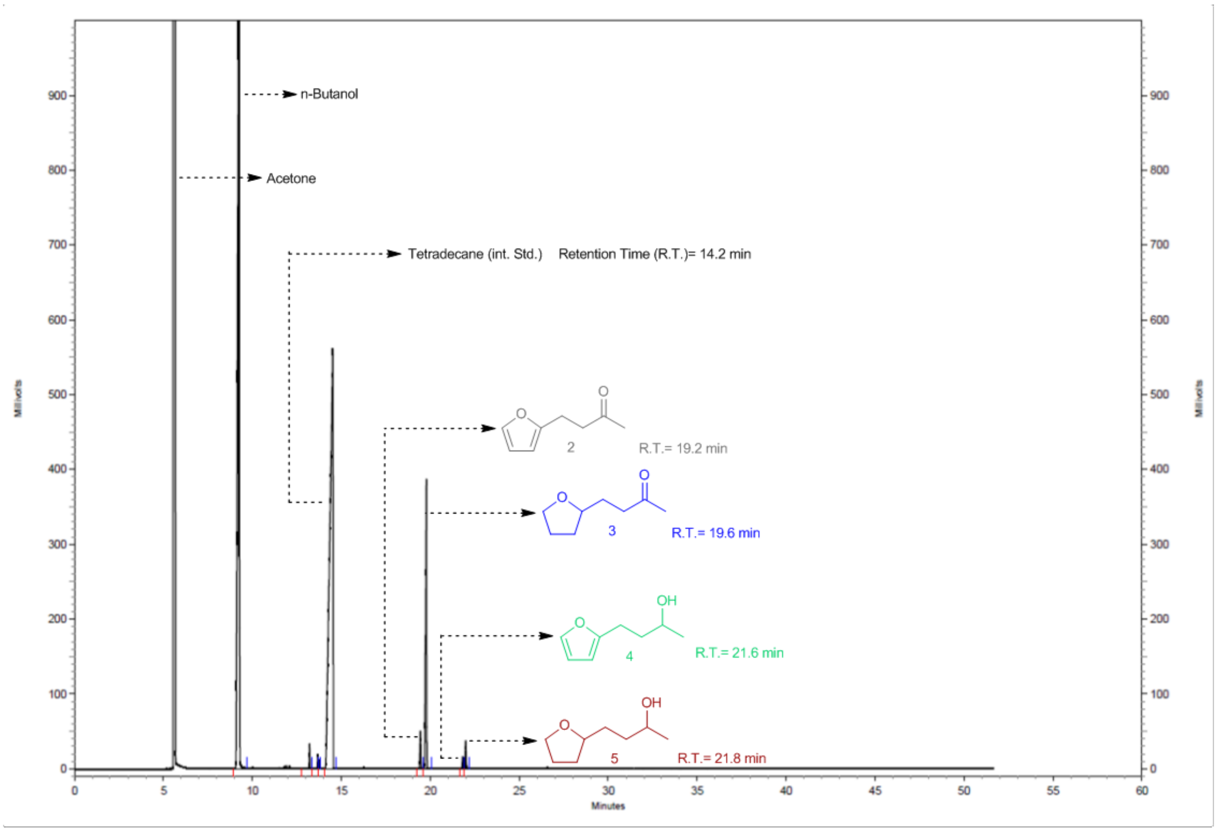

Supplementary Figure 56: Chromatogram corresponding to Supplementary Table 3, Entry 12.

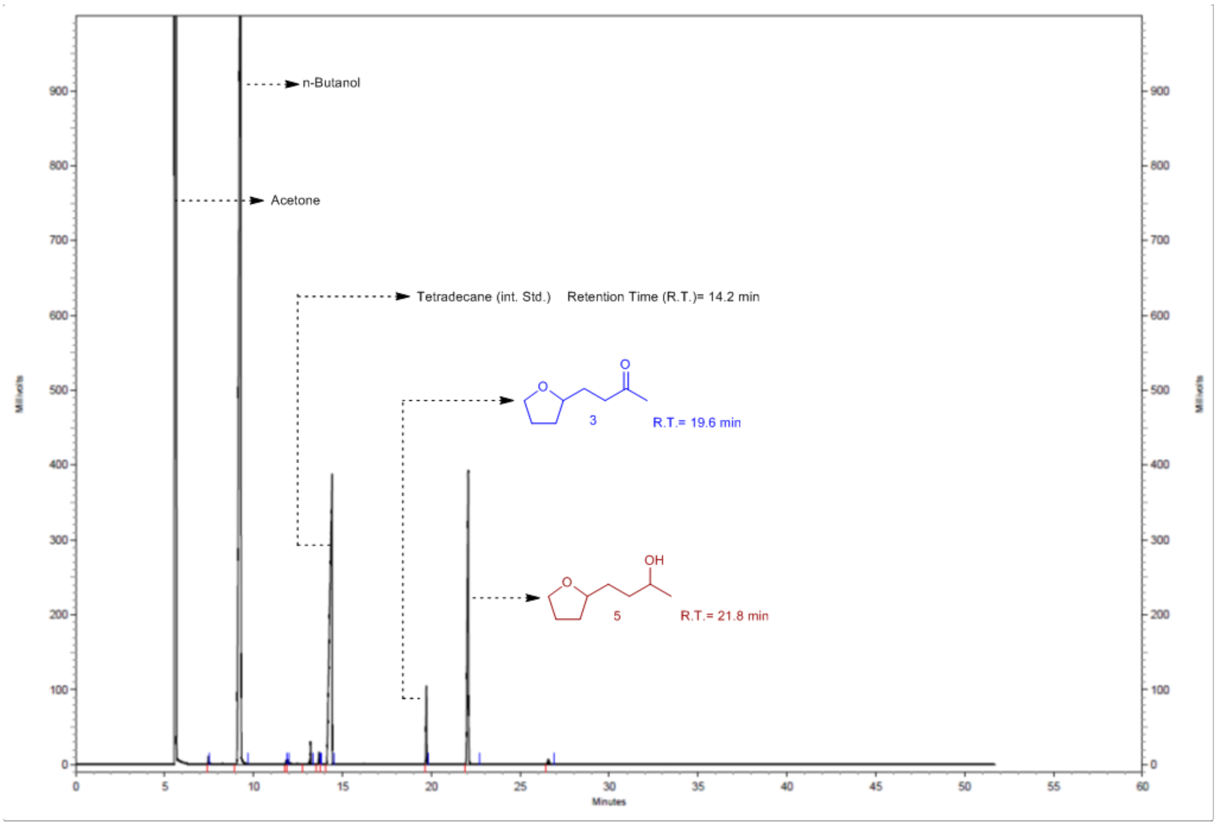

Supplementary Figure 57: Chromatogram corresponding to Supplementary Table 3, Entry 13.

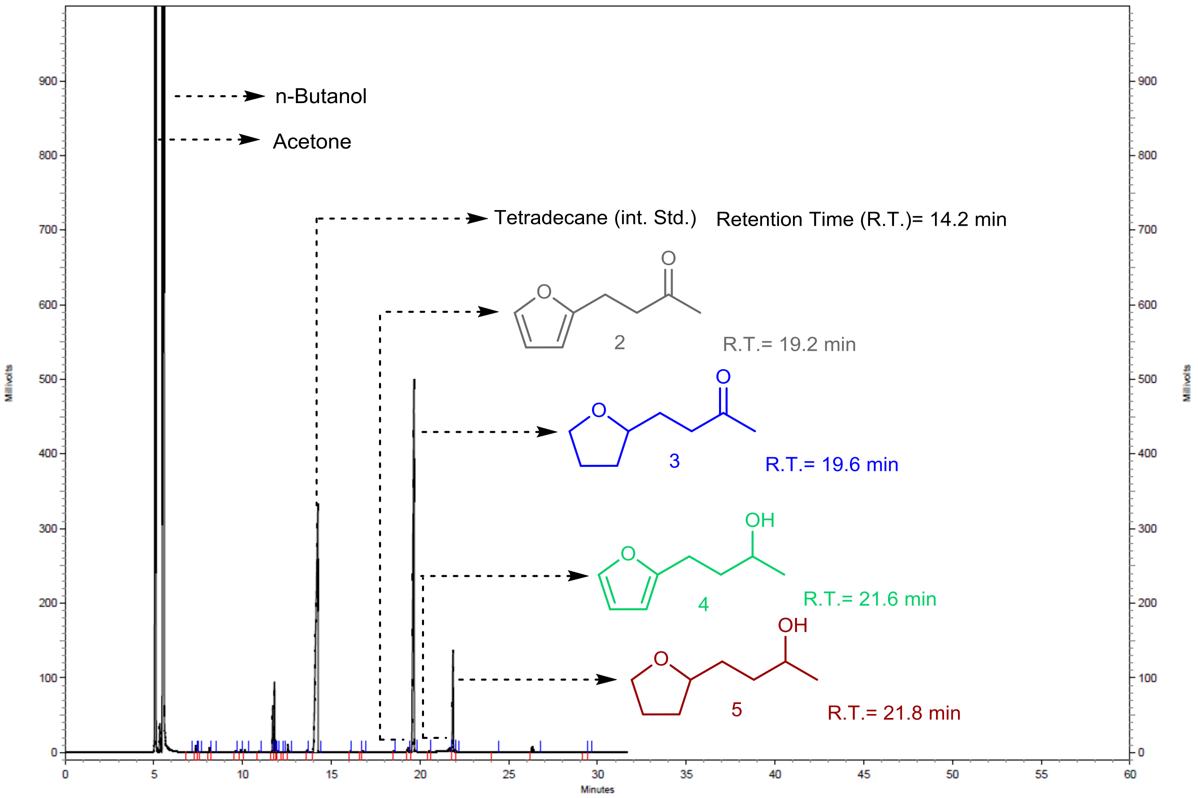

Supplementary Figure 58: Chromatogram corresponding to Supplementary Table 3, Entry 14.

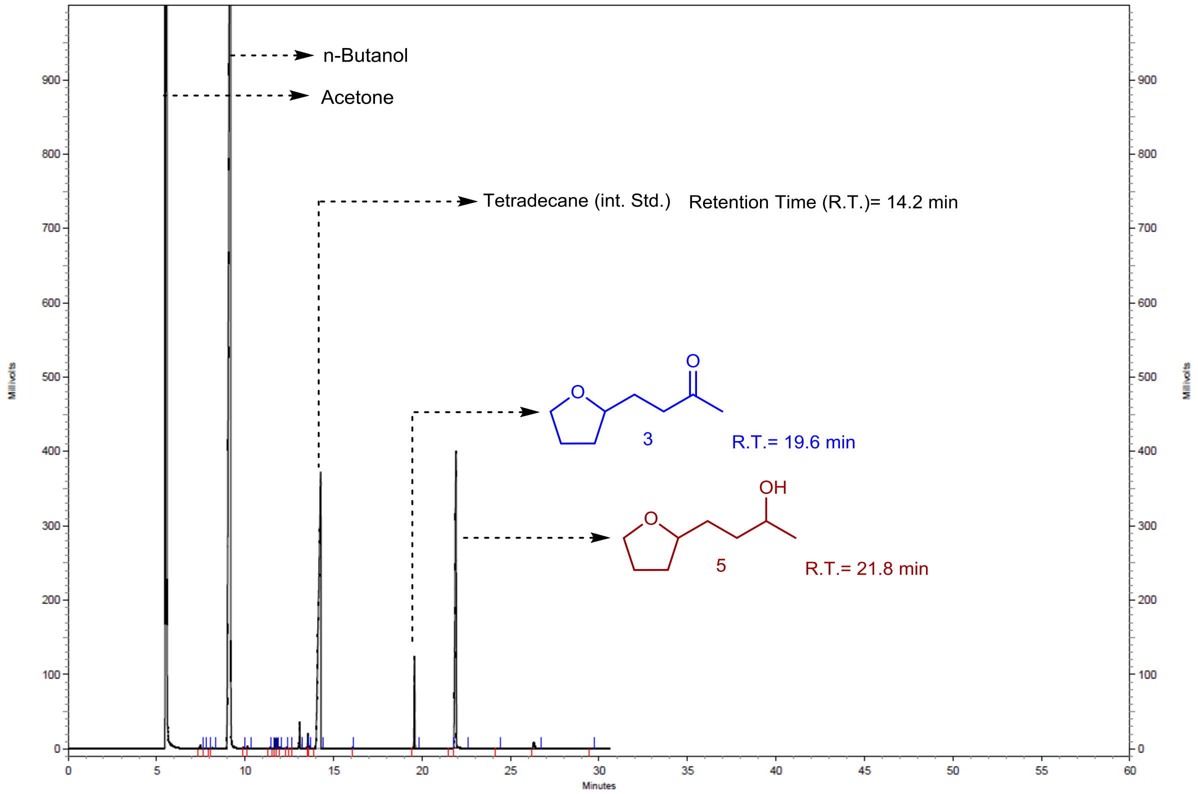

Supplementary Figure 59: Chromatogram corresponding to Supplementary Table 3, Entry 15.

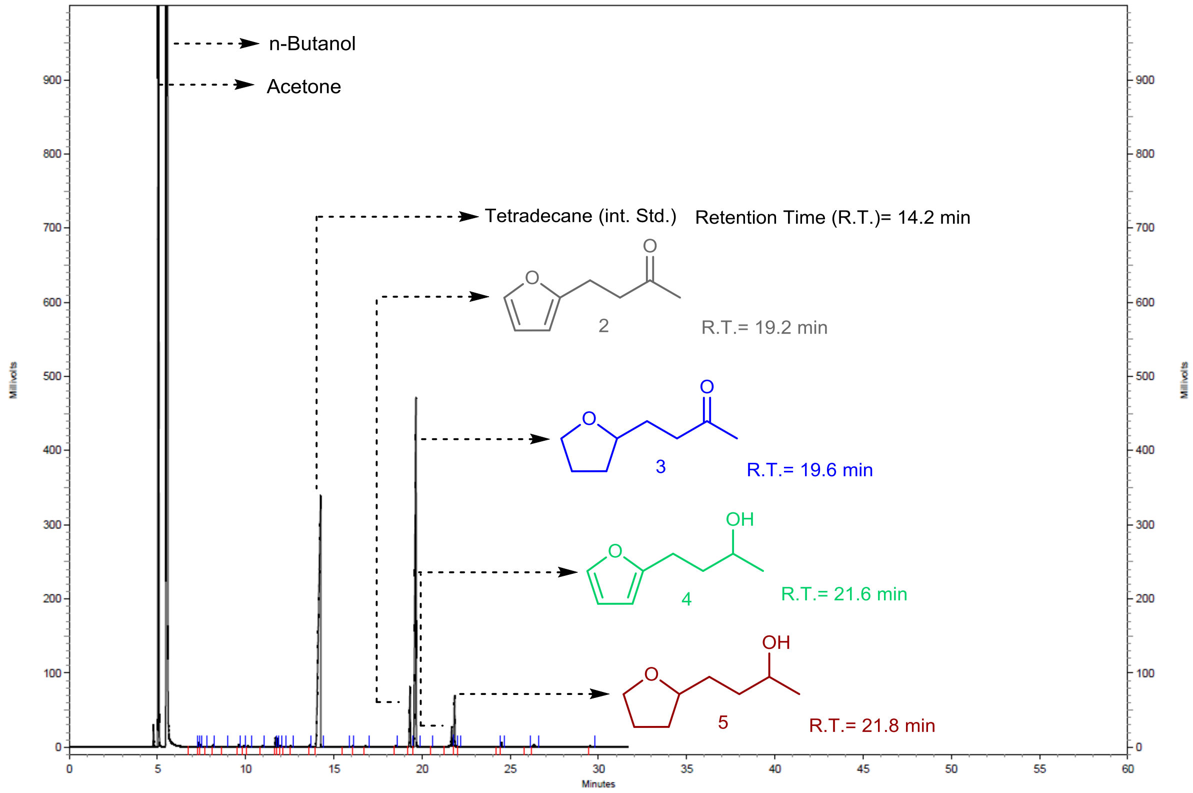

Supplementary Figure 60: Chromatogram corresponding to Supplementary Table 3, Entry 16.

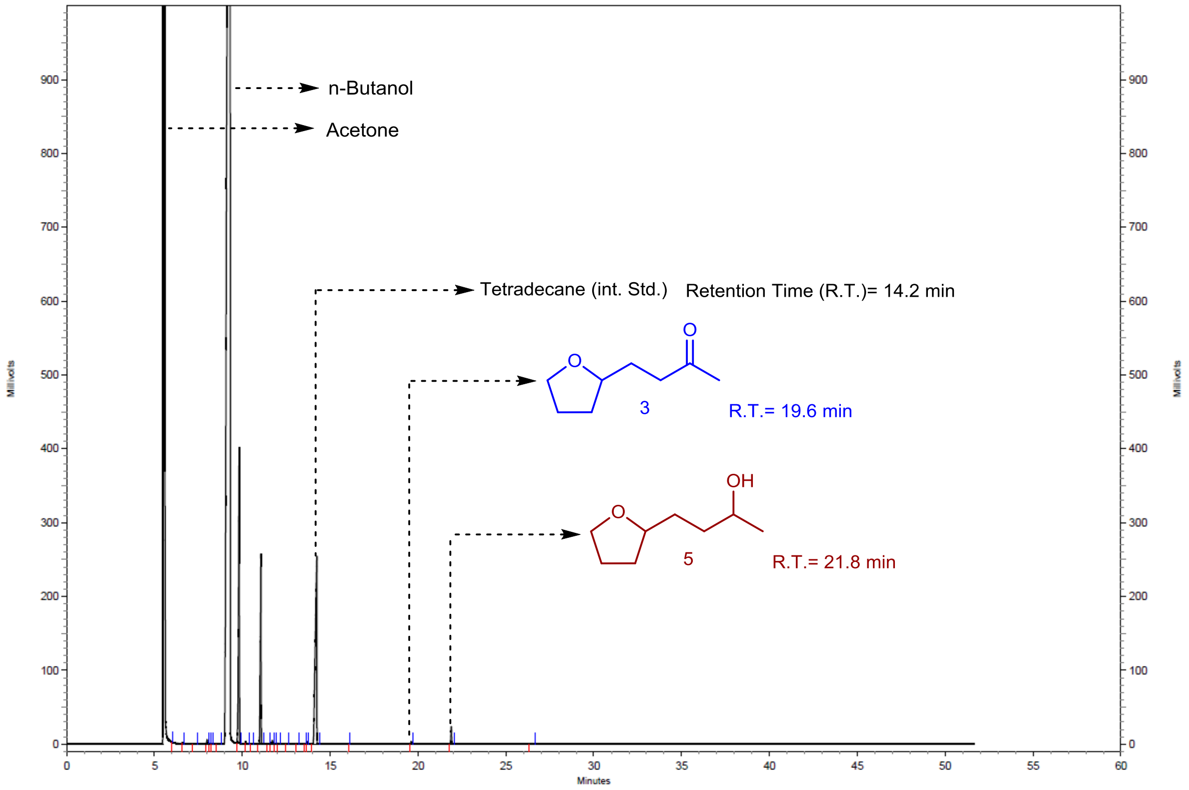

Supplementary Figure 61: Chromatogram corresponding to Supplementary Table 3, Entry 17.

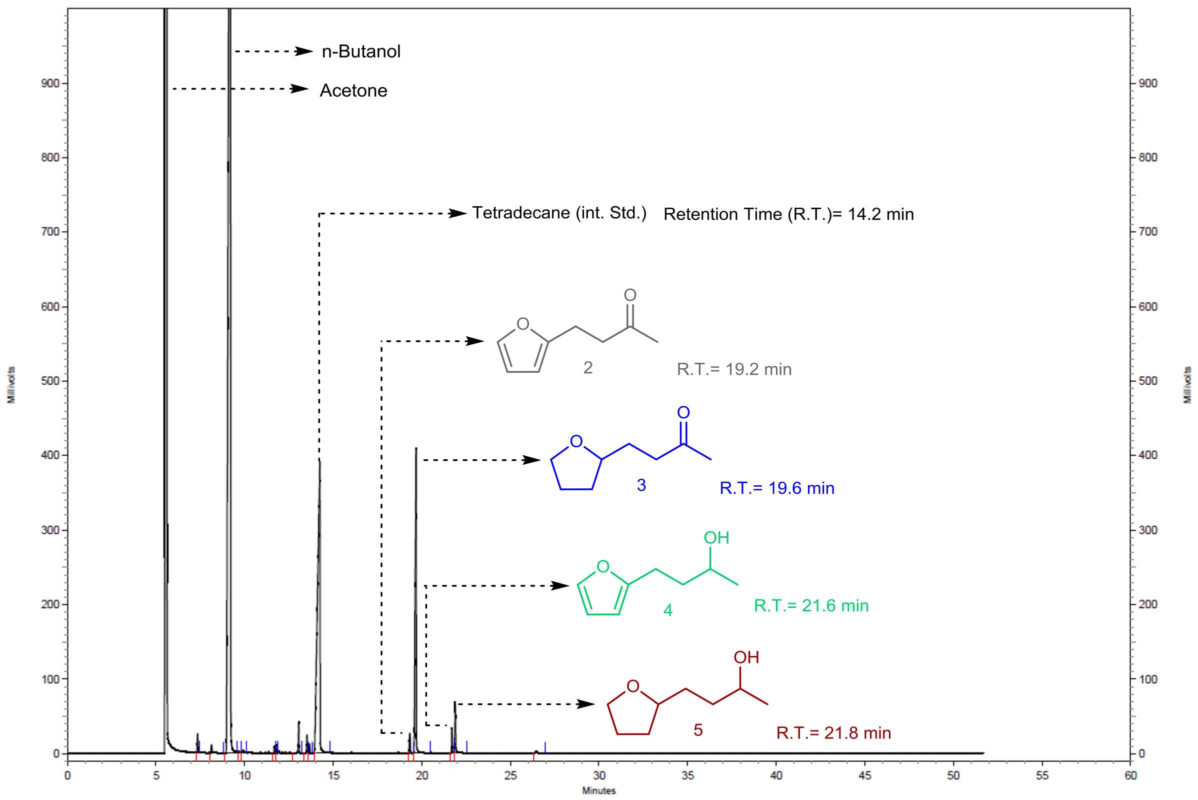

Supplementary Figure 62: Chromatogram corresponding to Supplementary Table 3, Entry 18.

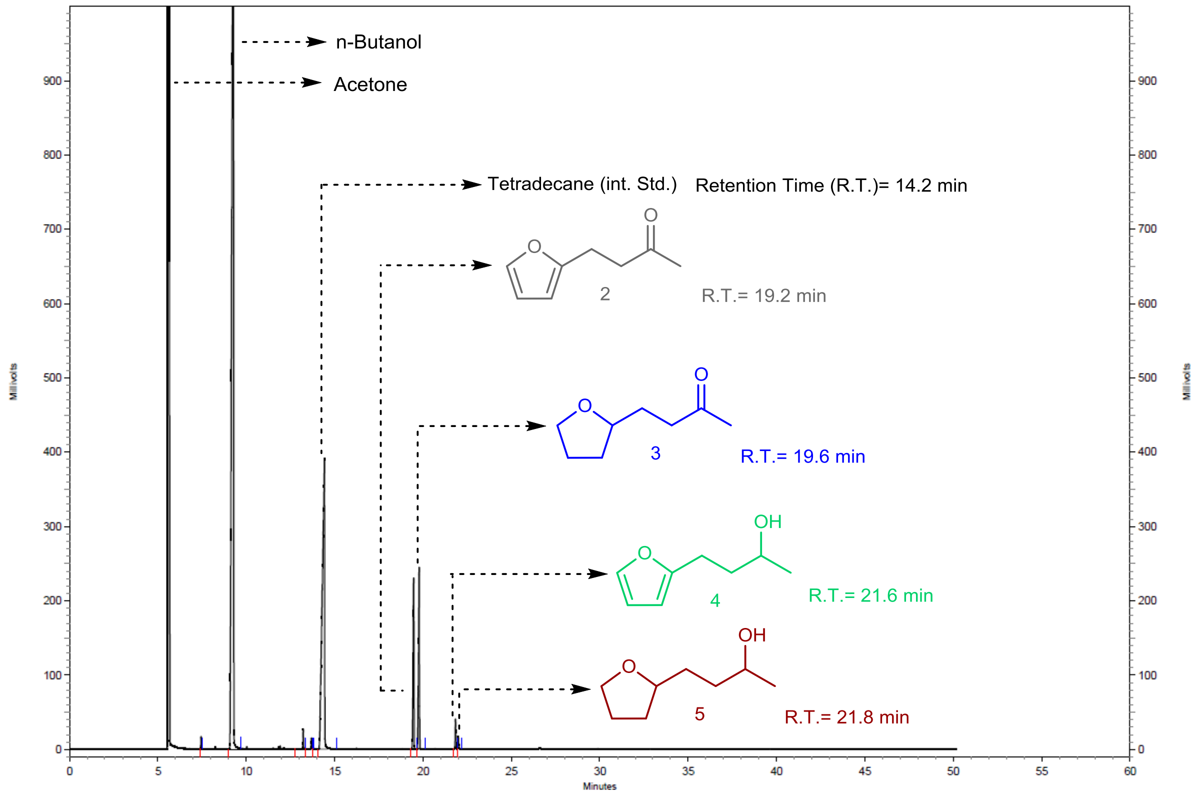

Supplementary Figure 63: Chromatogram corresponding to Supplementary Table 3, Entry 19.

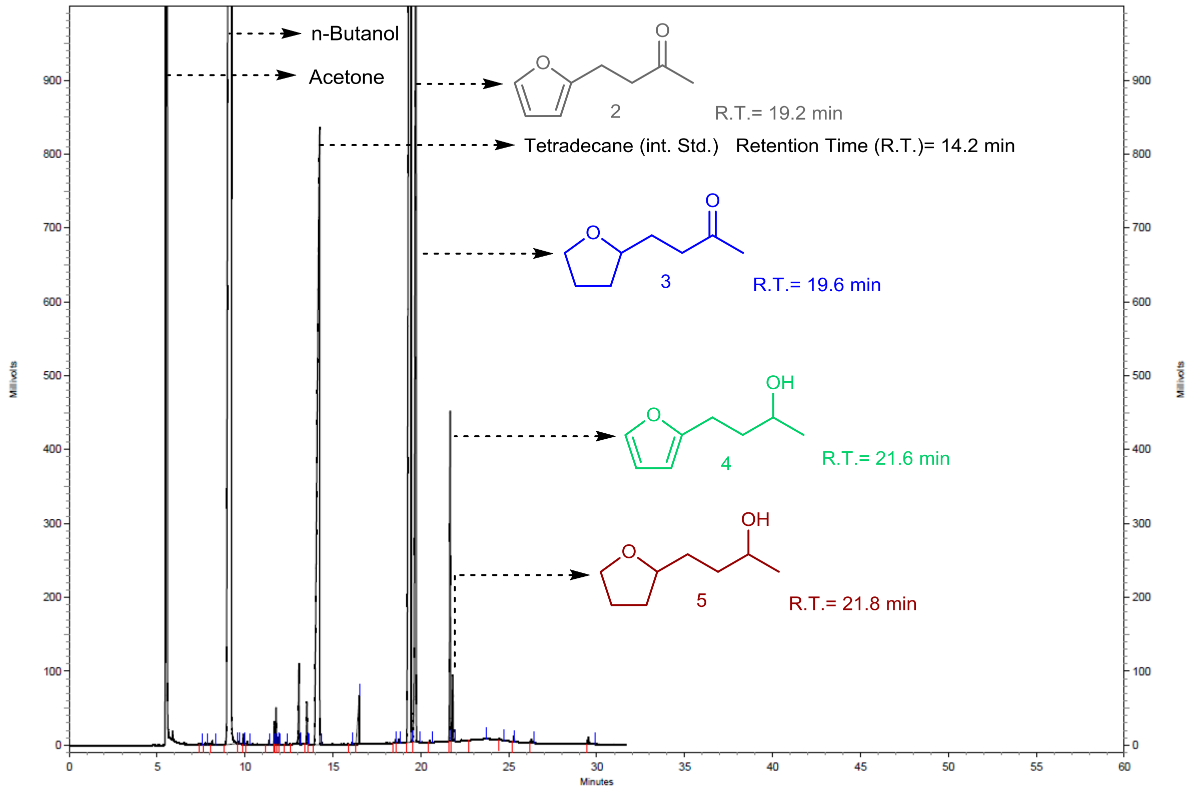

Supplementary Figure 64: Chromatogram corresponding to Supplementary Table 4, Entry 1.

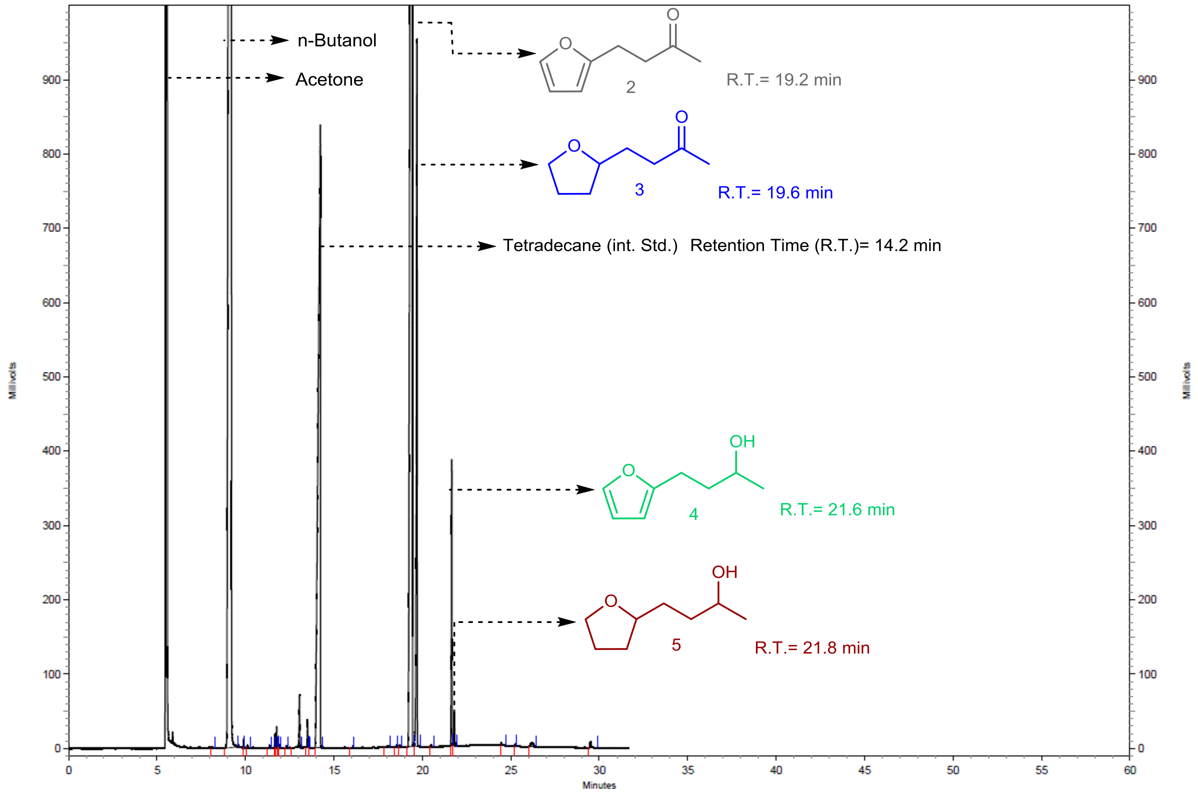

Supplementary Figure 65: Chromatogram corresponding to Supplementary Table 4, Entry 2.

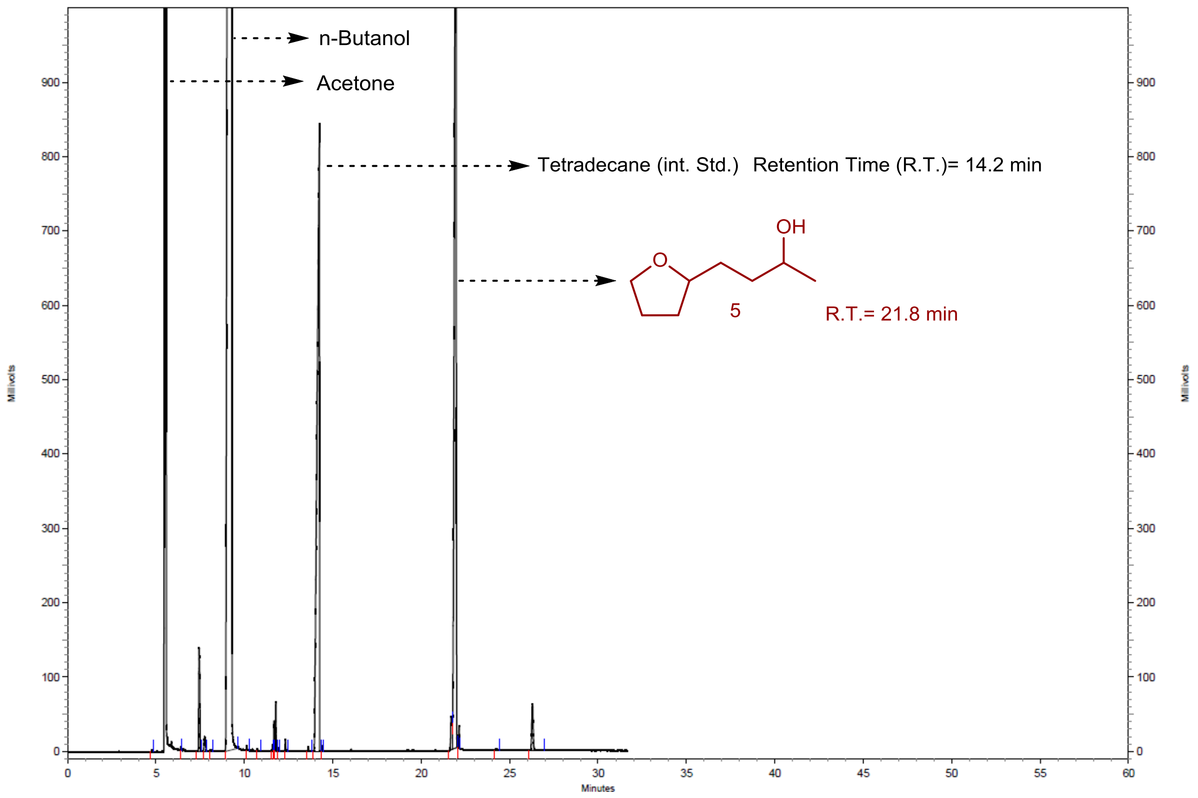

Supplementary Figure 66: Chromatogram corresponding to Supplementary Table 5, Entry 1.

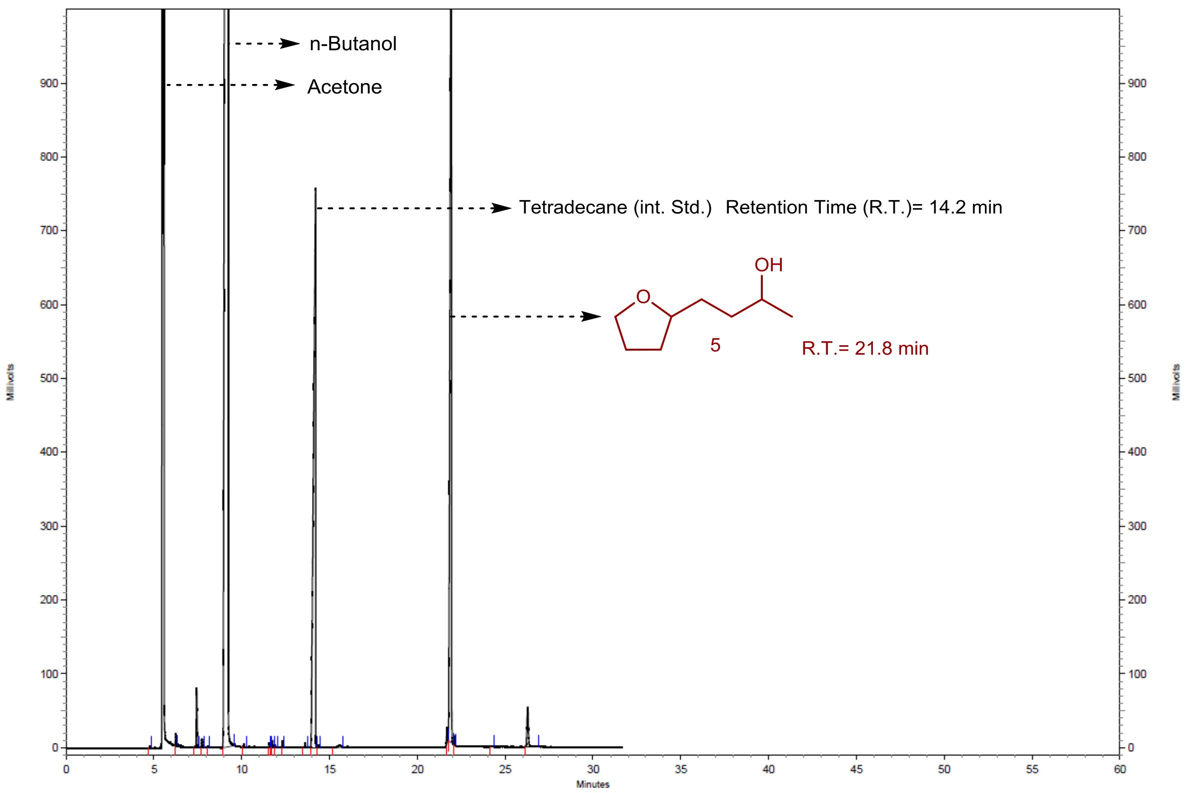

Supplementary Figure 67: Chromatogram corresponding to Supplementary Table 5, Entry 2.

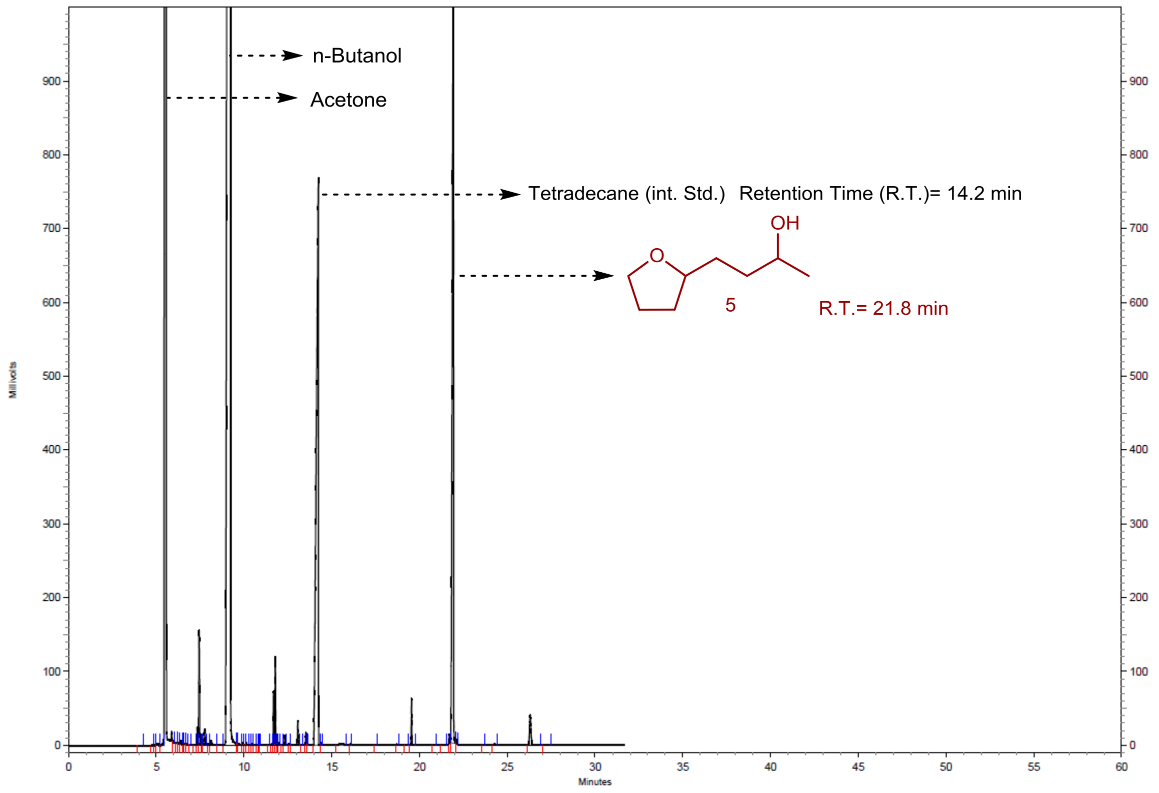

Supplementary Figure 68: Chromatogram corresponding to Supplementary Table 5, Entry 3.

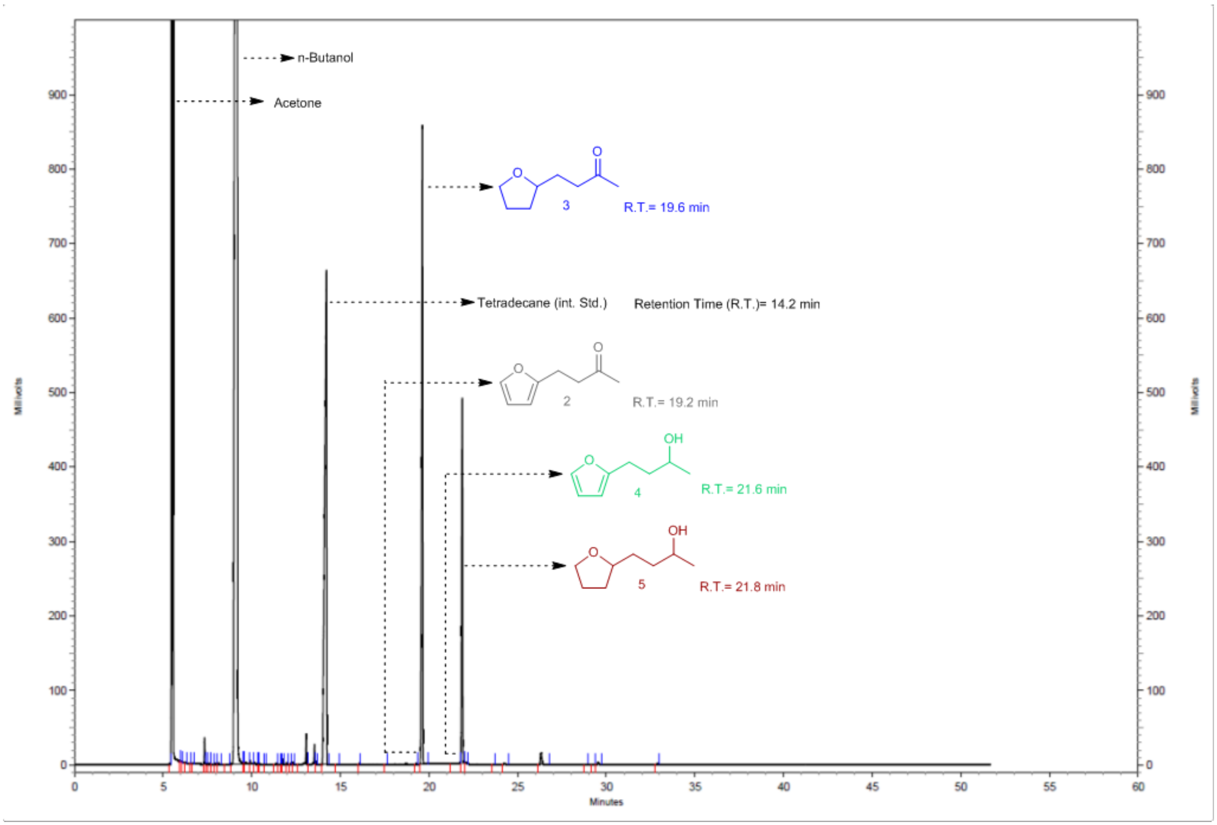

Supplementary Figure 69: Chromatogram corresponding to Supplementary Table 5, Entry 4.

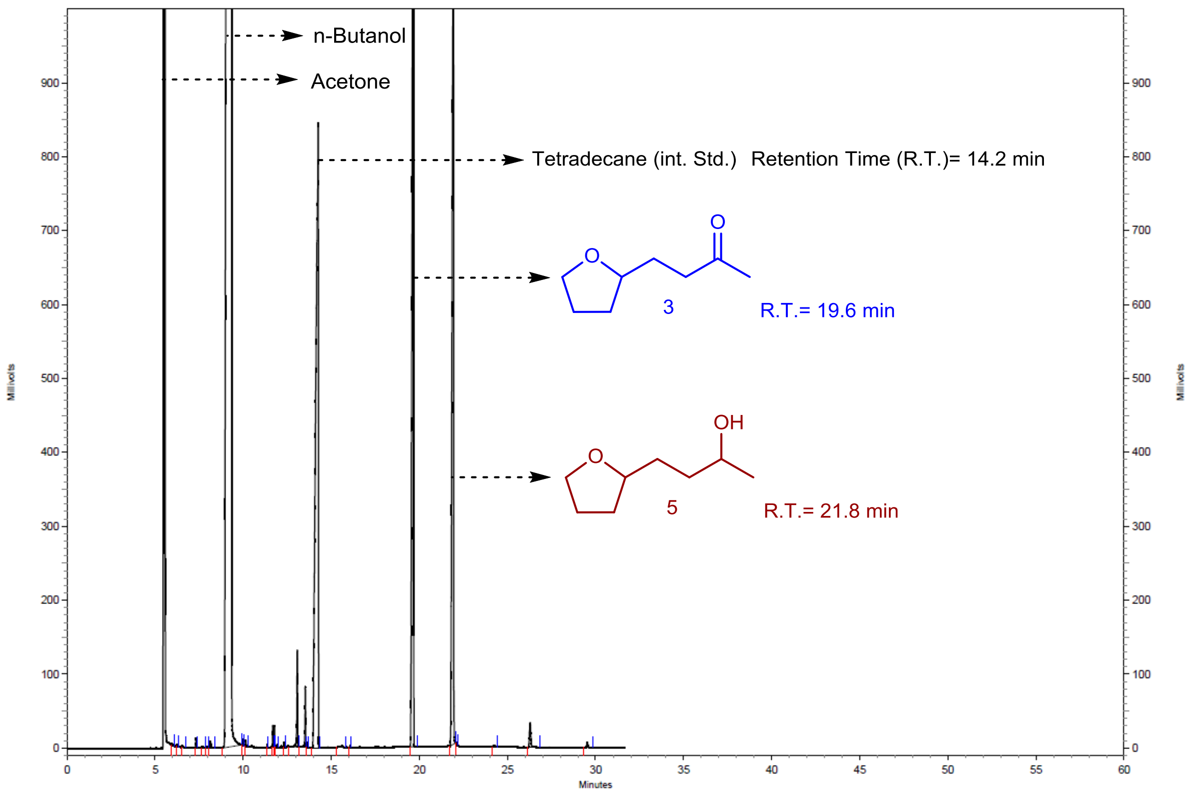

Supplementary Figure 70: Chromatogram corresponding to Supplementary Table 5, Entry 5.

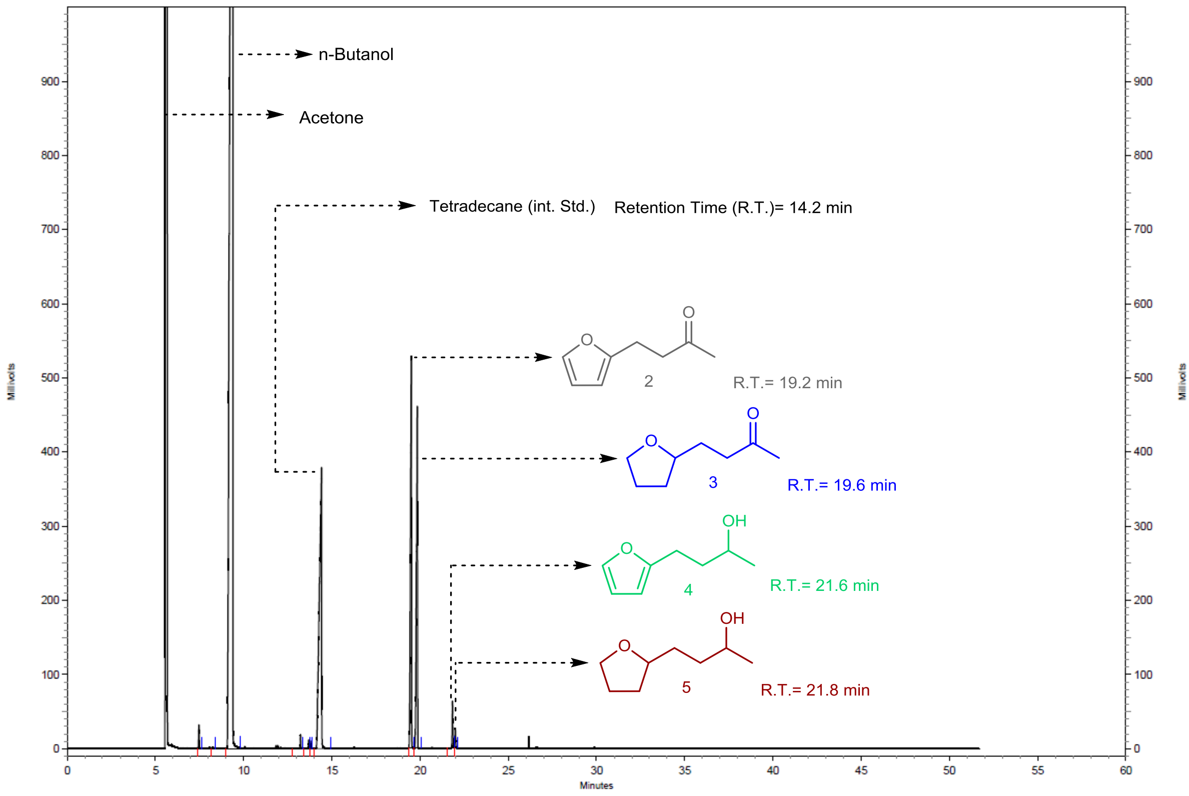

Supplementary Figure 71: Chromatogram corresponding to Supplementary Table 5, Entry 6.

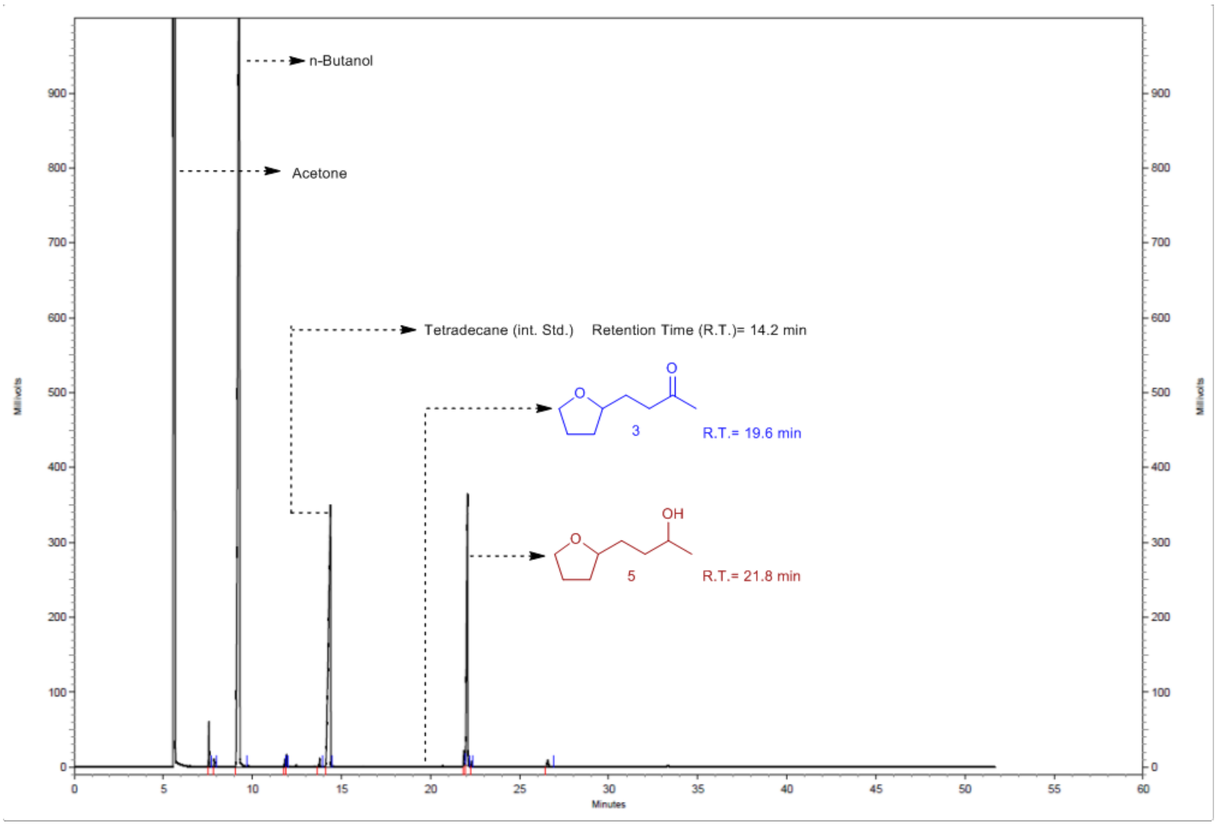

Supplementary Figure 72: Chromatogram corresponding to Supplementary Table 7, Entry 1.

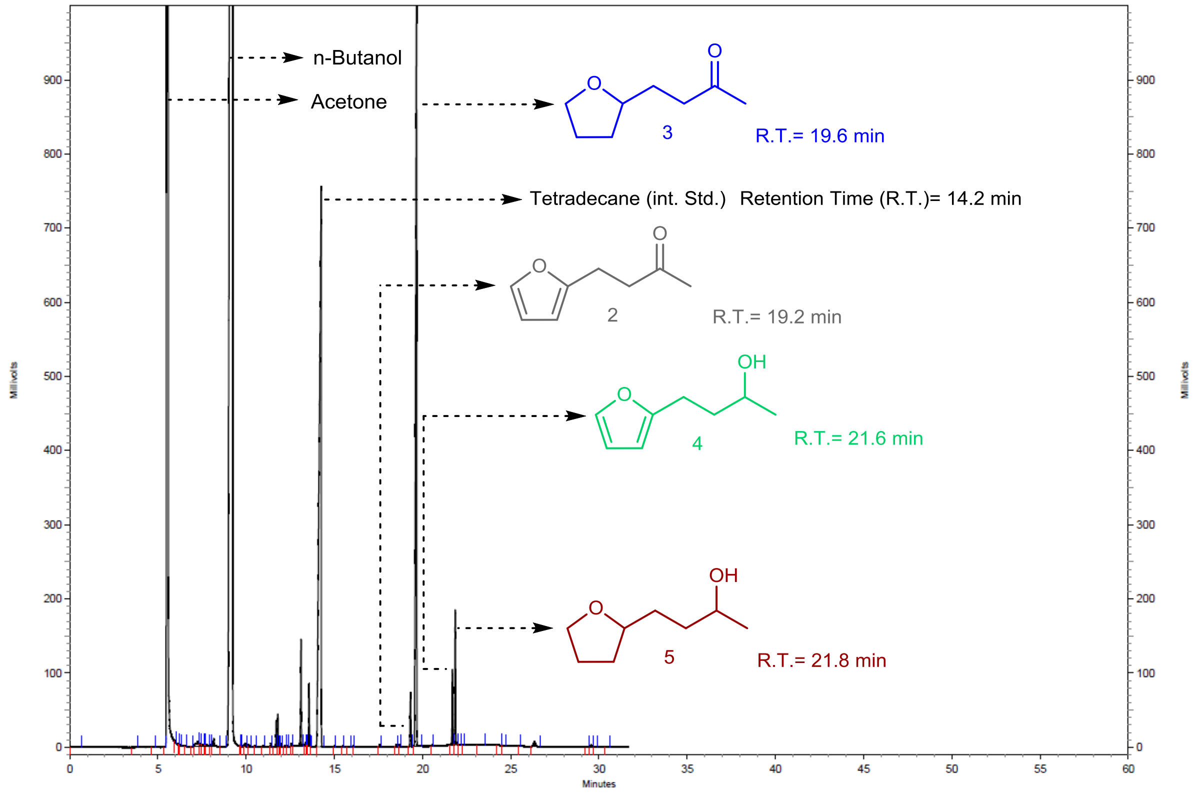

Supplementary Figure 73: Chromatogram corresponding to Supplementary Table 7, Entry 2.

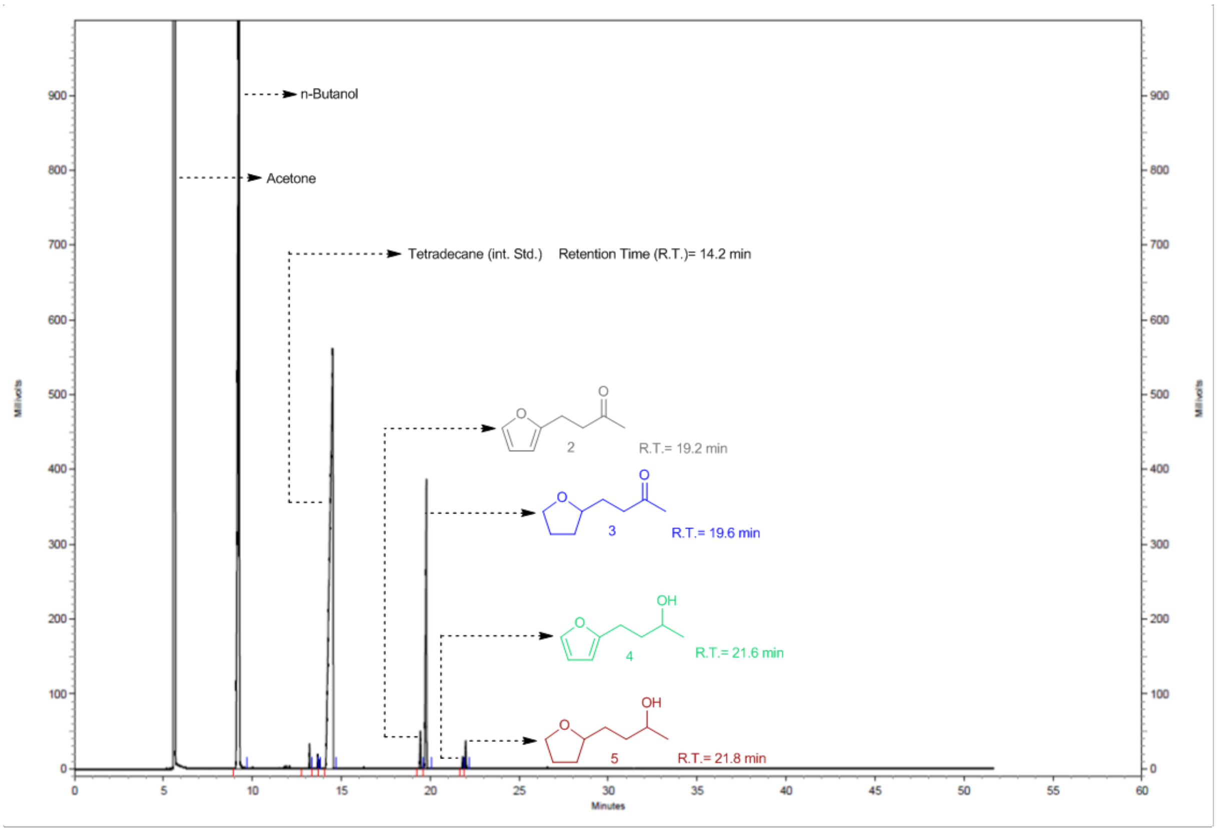

Supplementary Figure 74: Chromatogram corresponding to Supplementary Table 7, Entry 3.

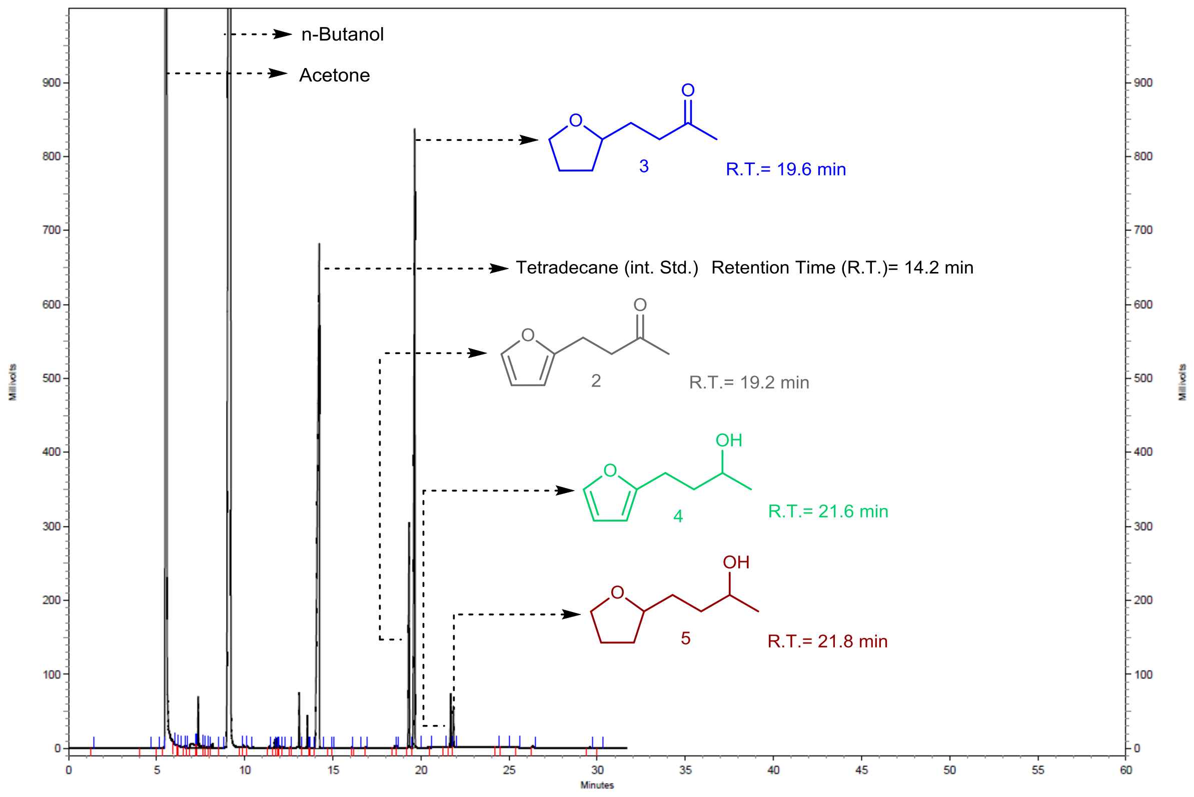

Supplementary Figure 75: Chromatogram corresponding to Supplementary Table 7, Entry 4.

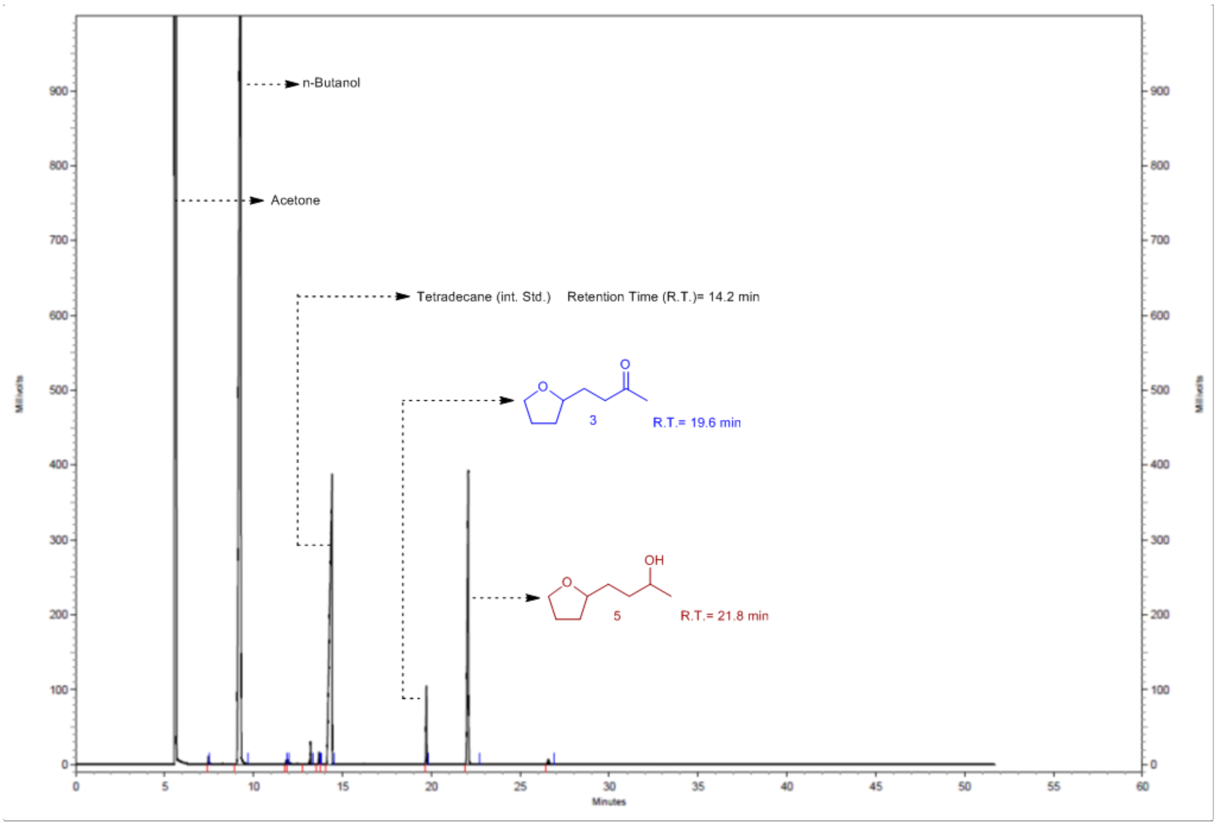

Supplementary Figure 76: Chromatogram corresponding to Supplementary Figure 6 (switch 1, H_2).

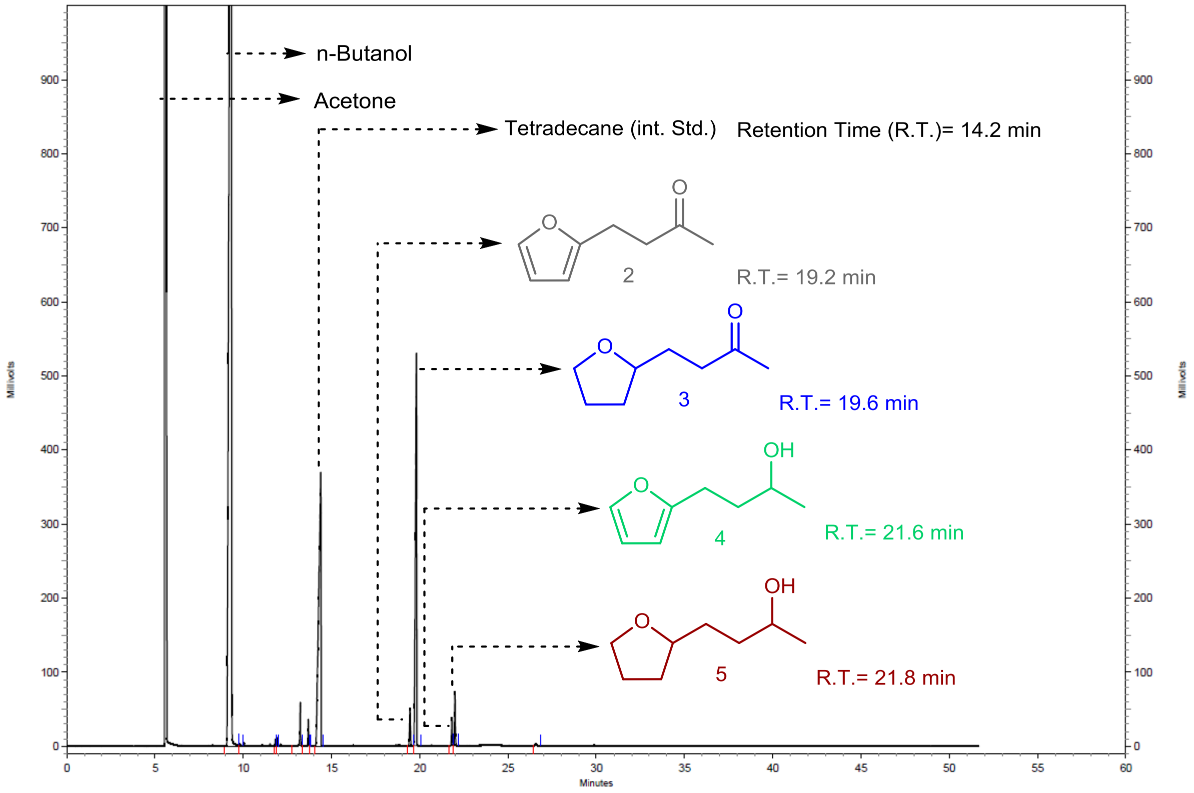

Supplementary Figure 77: Chromatogram corresponding to Supplementary Figure 6 (switch 2, H_2/CO_2).

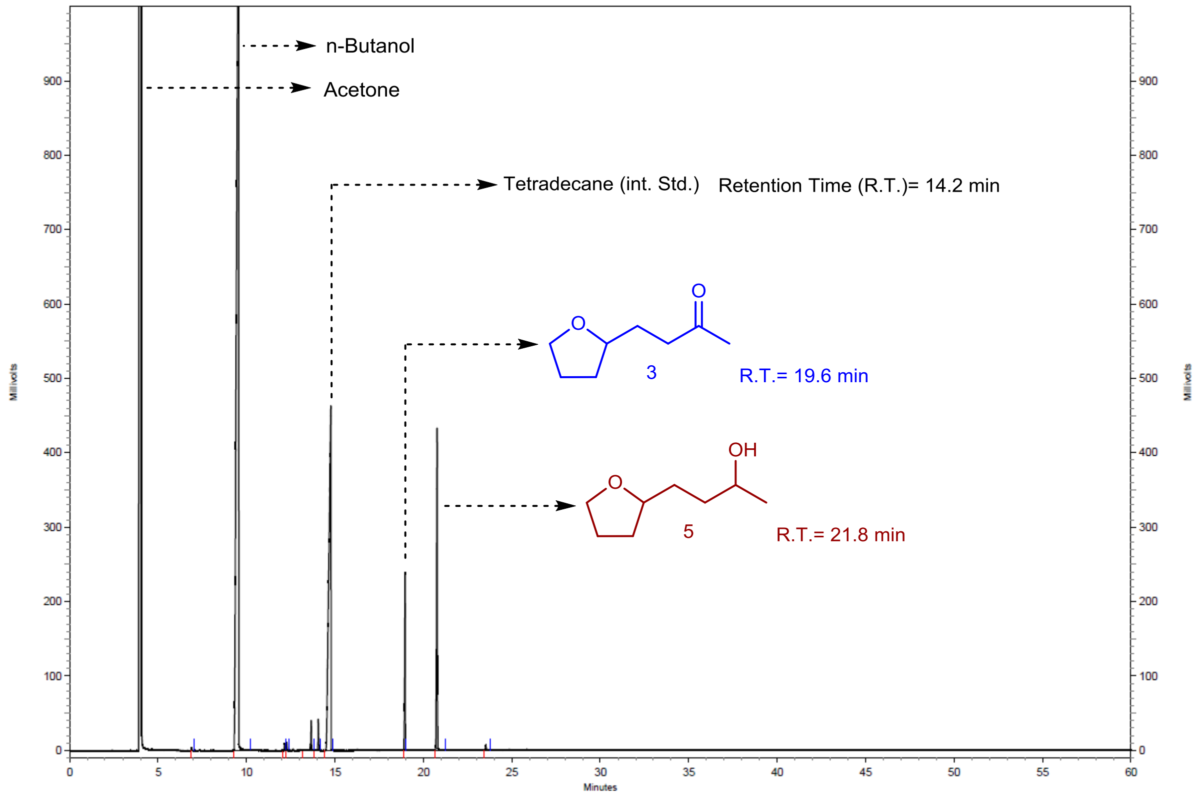

Supplementary Figure 78: Chromatogram corresponding to Supplementary Figure 6 (switch 3, H_2).

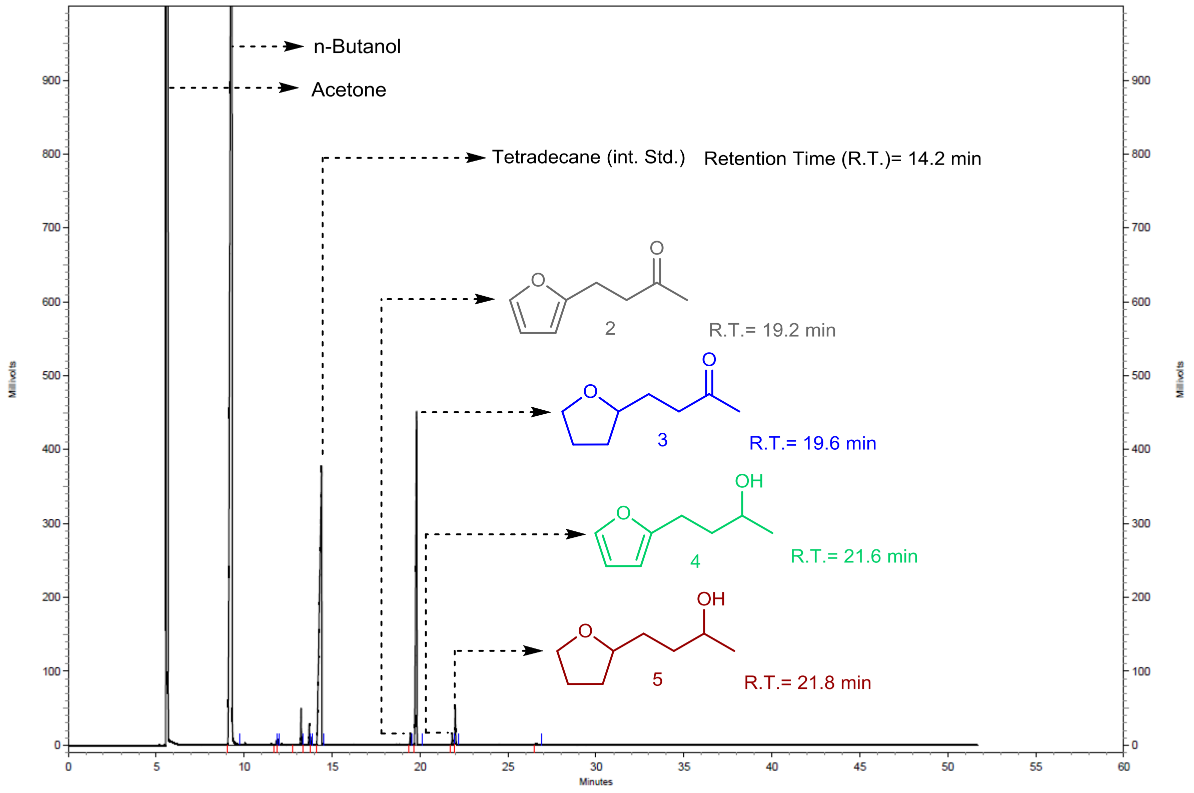

Supplementary Figure 79: Chromatogram corresponding to Supplementary Figure 6 (switch 4, H_2/CO_2).

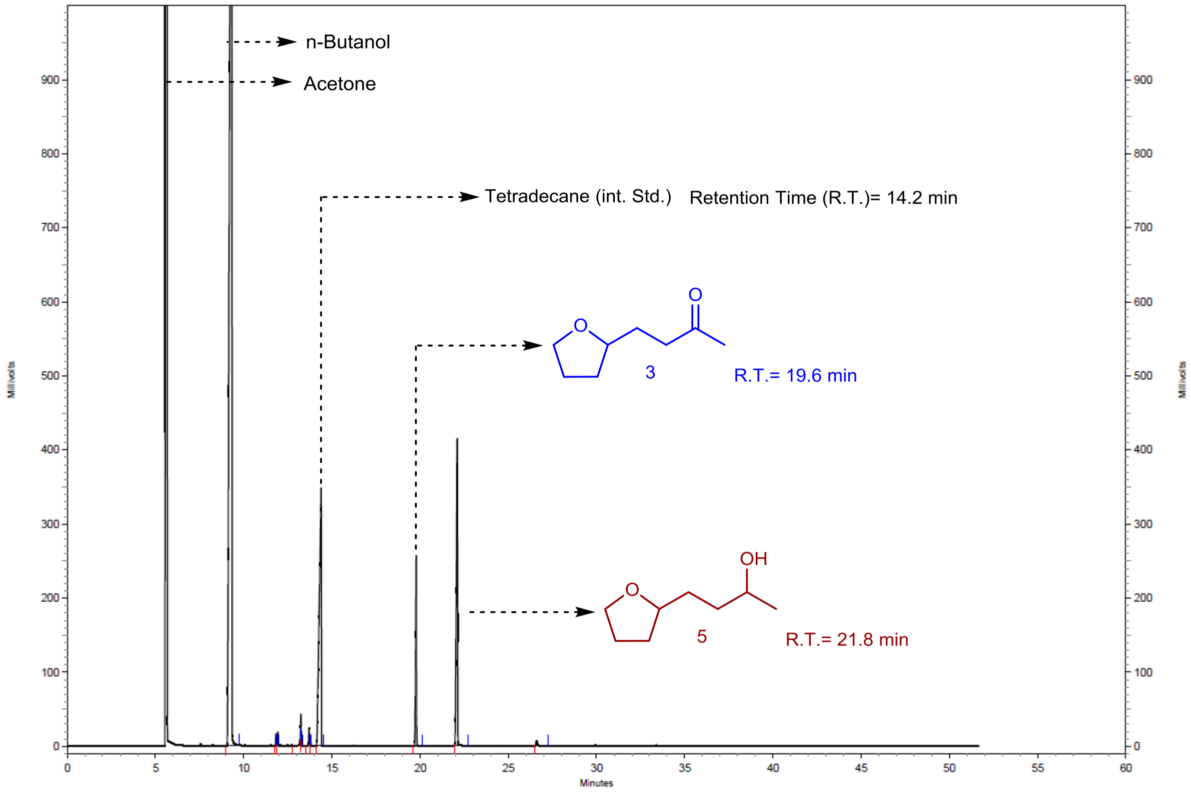

Supplementary Figure 80: Chromatogram corresponding to Supplementary Figure 6 (switch 5, H_2).

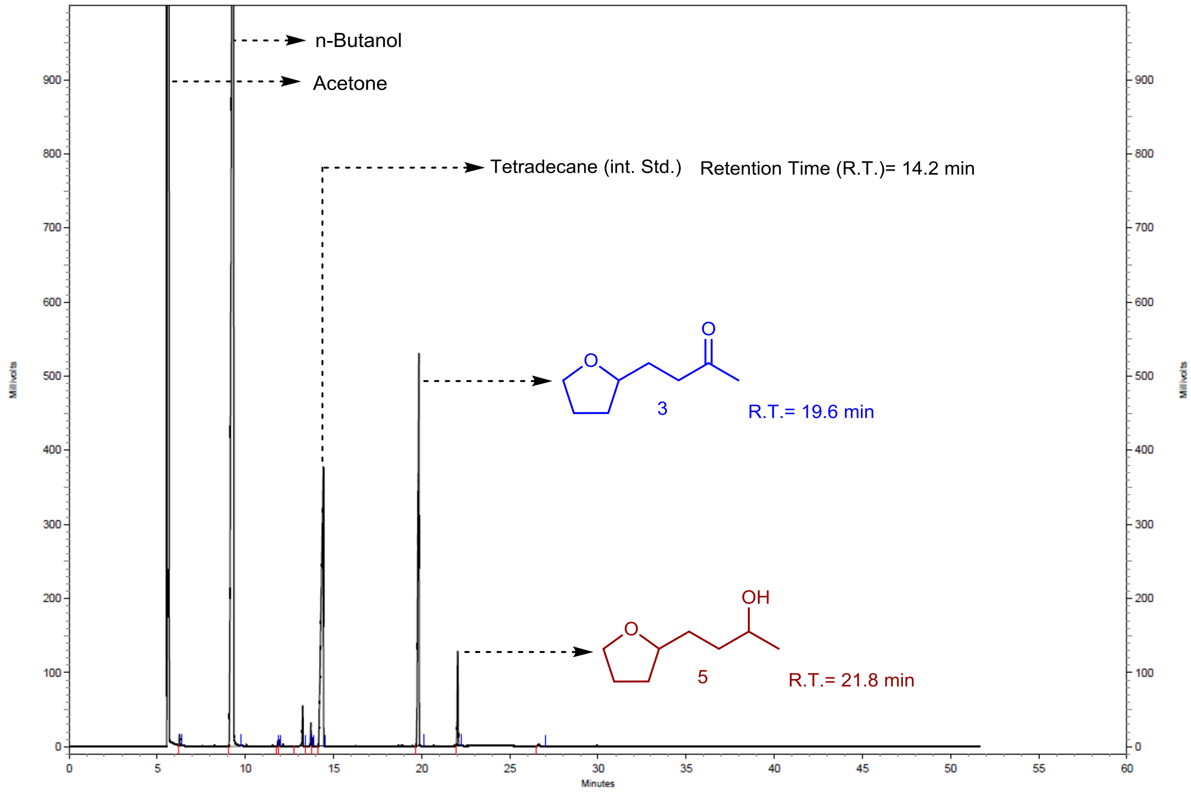

Supplementary Figure 81: Chromatogram corresponding to Supplementary Figure 6 (switch 6, H_2/CO_2).

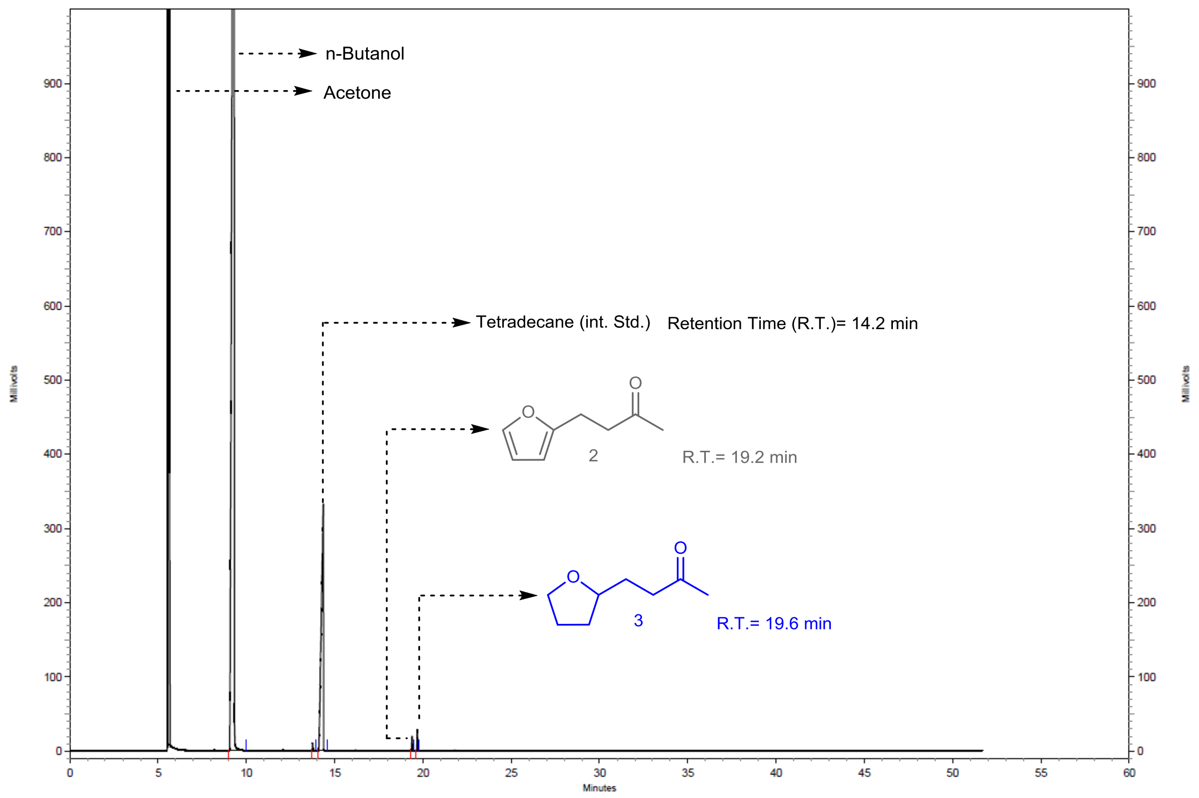

Supplementary Figure 82: Chromatogram corresponding to Supplementary Table 8, Entry 1.

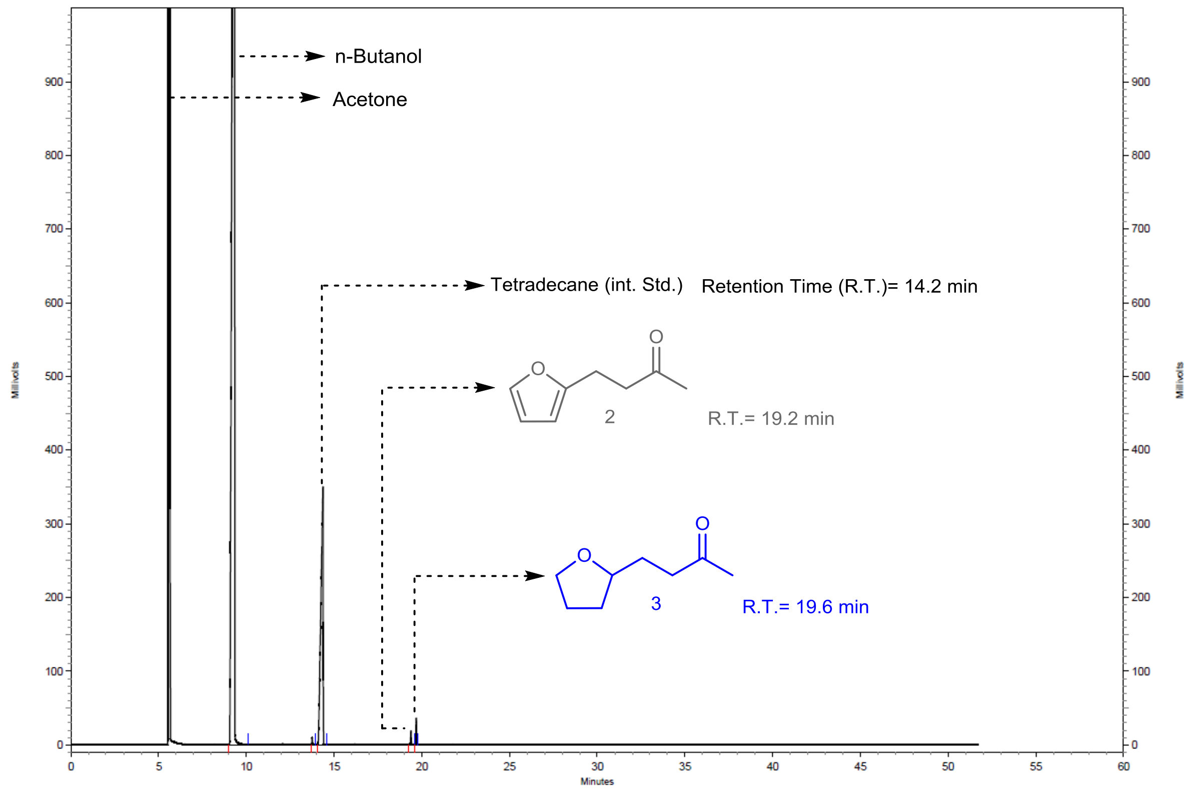

Supplementary Figure 83: Chromatogram corresponding to Supplementary Table 8, Entry 2.

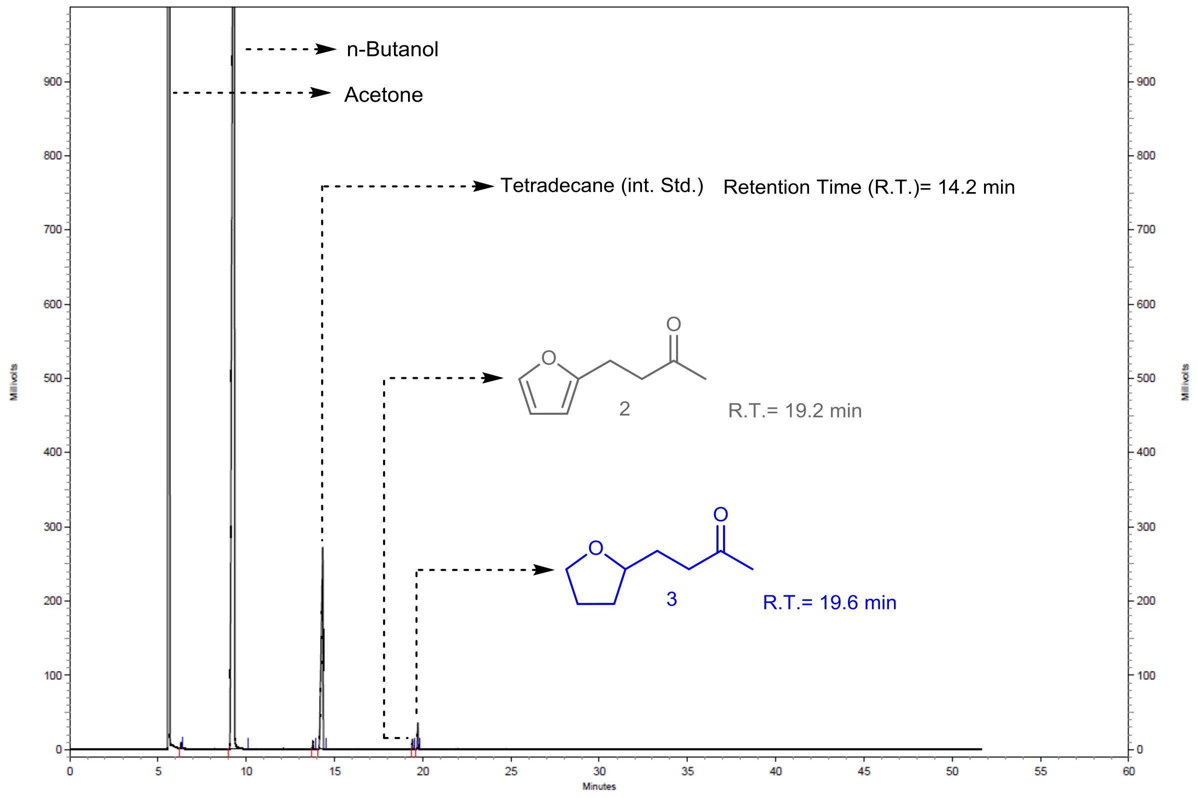

Supplementary Figure 84: Chromatogram corresponding to Supplementary Table 8, Entry 3.

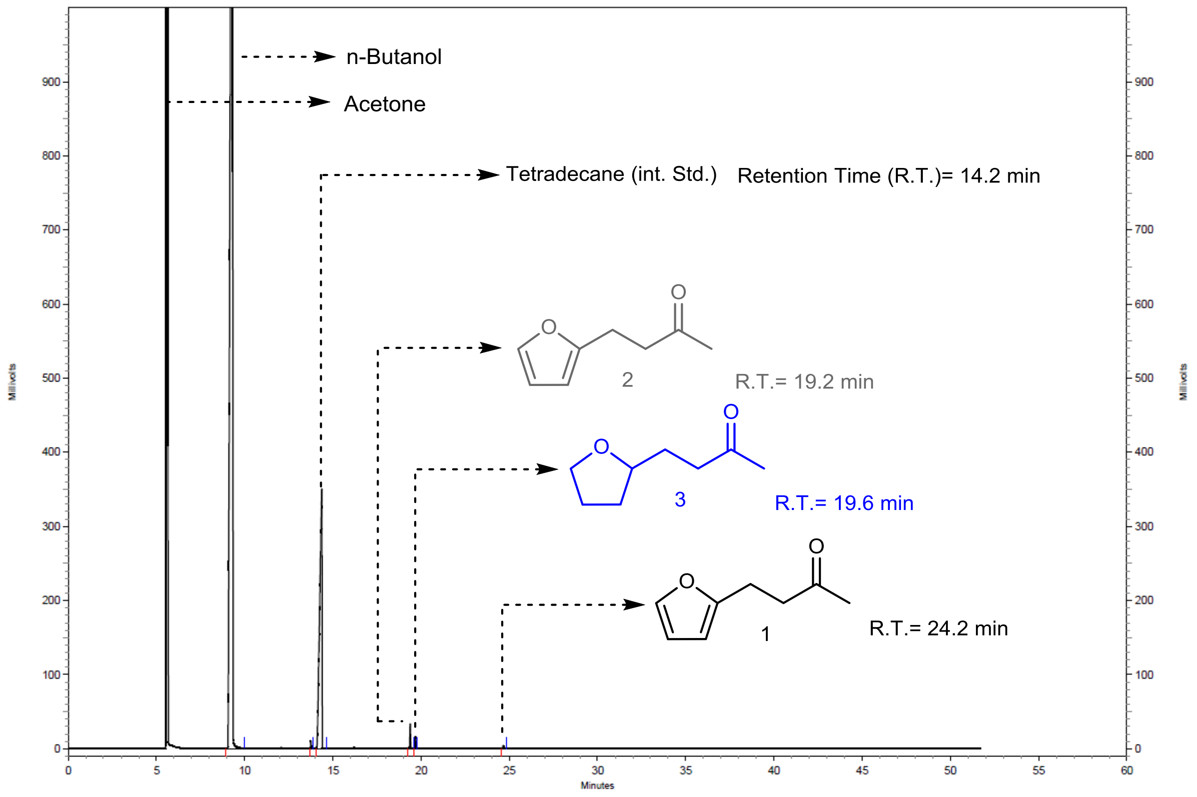

Supplementary Figure 85: Chromatogram corresponding to Supplementary Table 8, Entry 4.

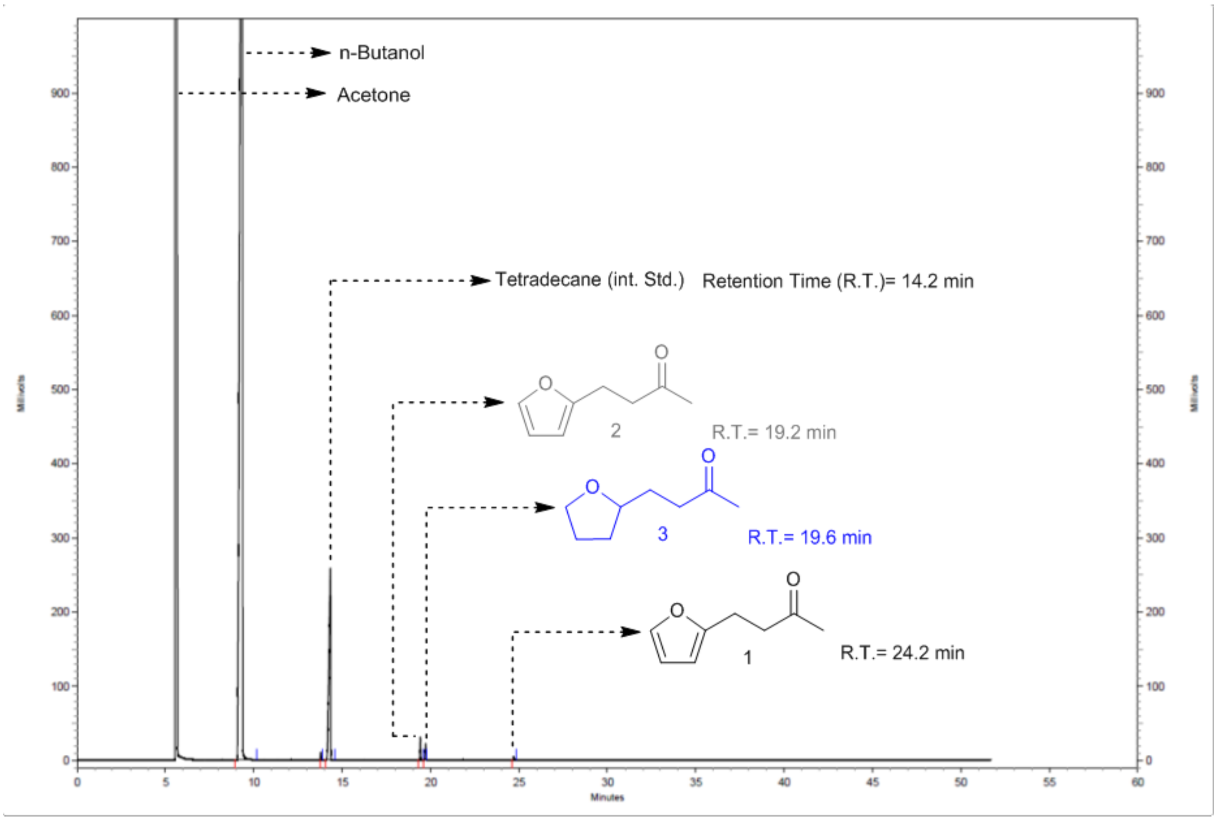

Supplementary Figure 86: Chromatogram corresponding to Supplementary Table 8, Entry 5.

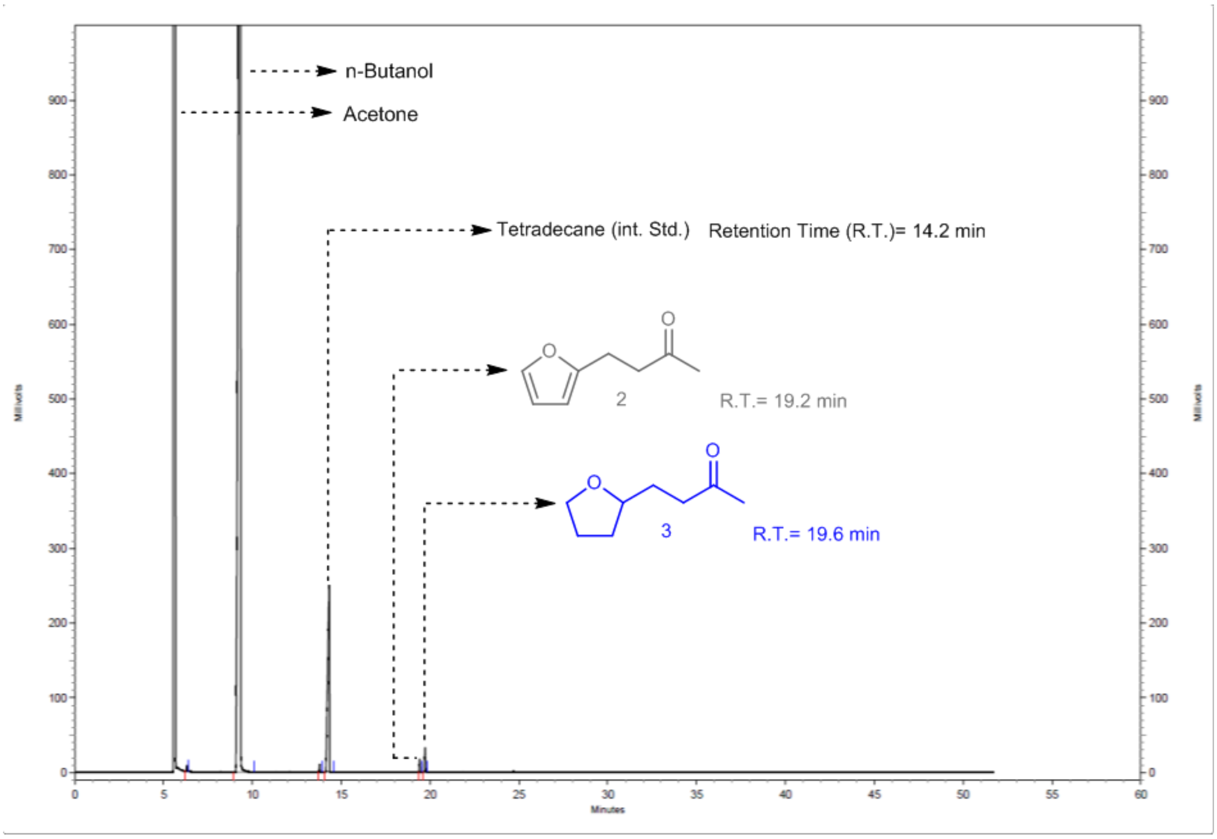

Supplementary Figure 87: Chromatogram corresponding to Supplementary Table 8, Entry 6.

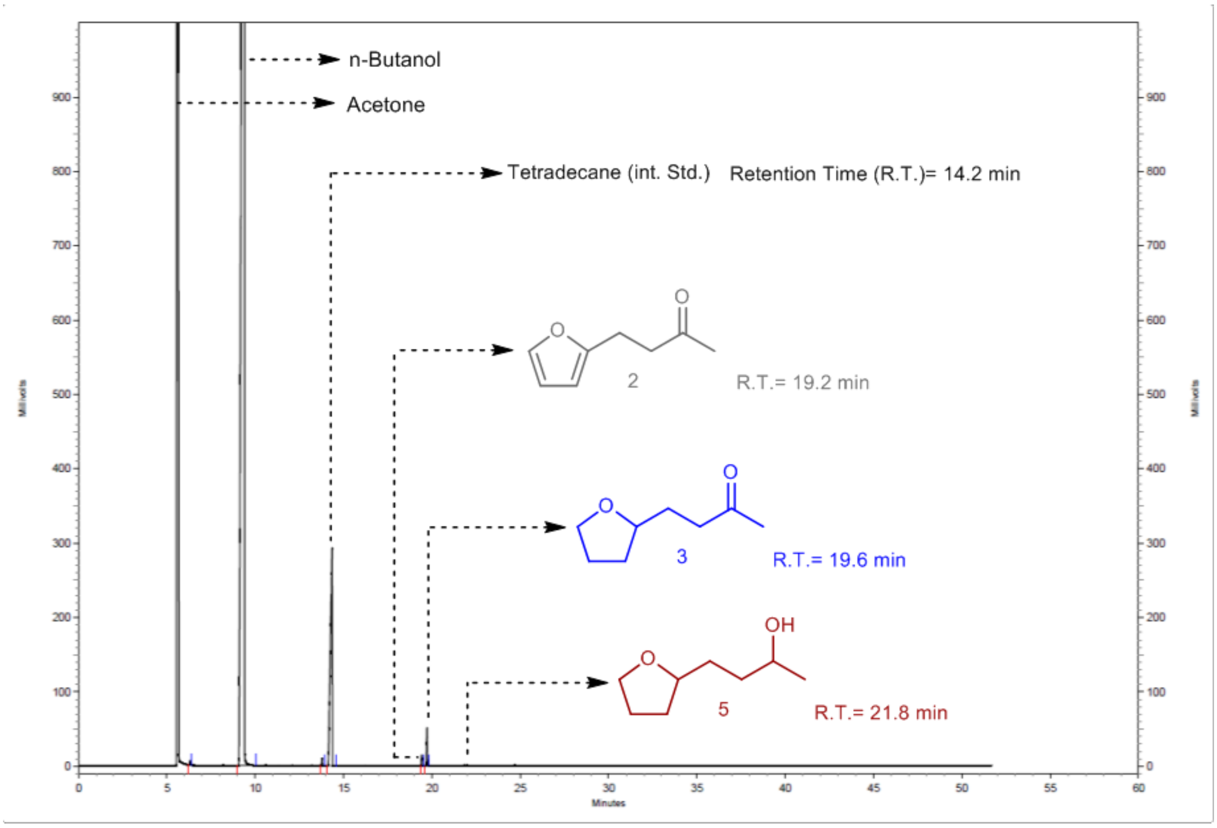

Supplementary Figure 88: Chromatogram corresponding to Supplementary Table 8, Entry 7.

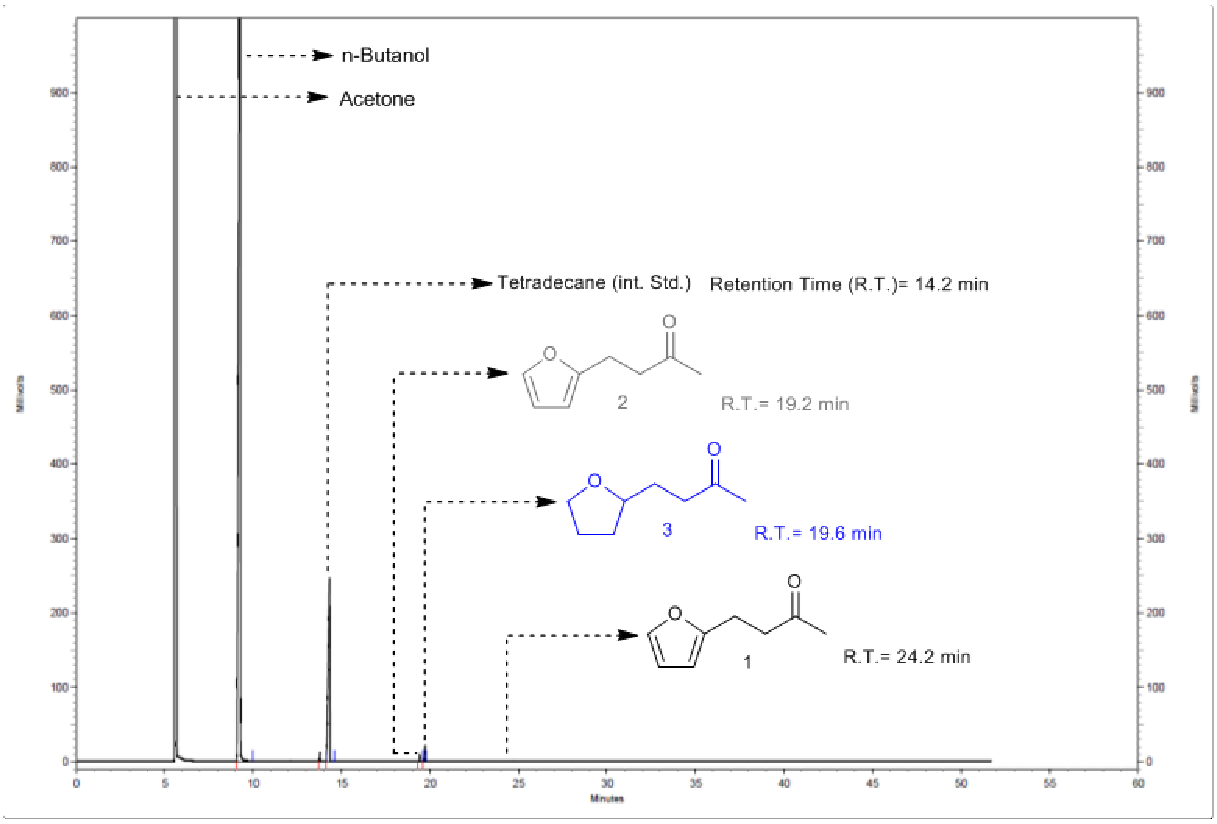

Supplementary Figure 89: Chromatogram corresponding to Supplementary Table 8, Entry 8.

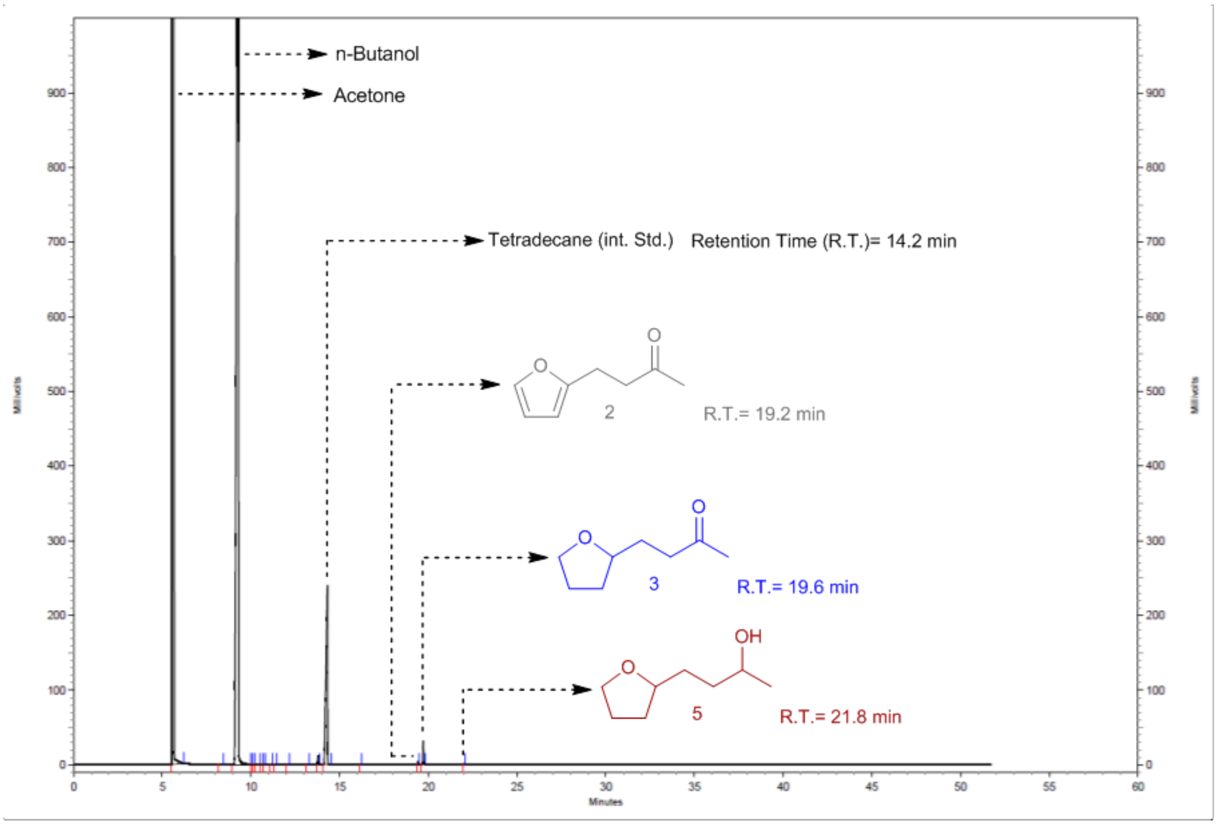

Supplementary Figure 90: Chromatogram corresponding to Supplementary Table 8, Entry 10.

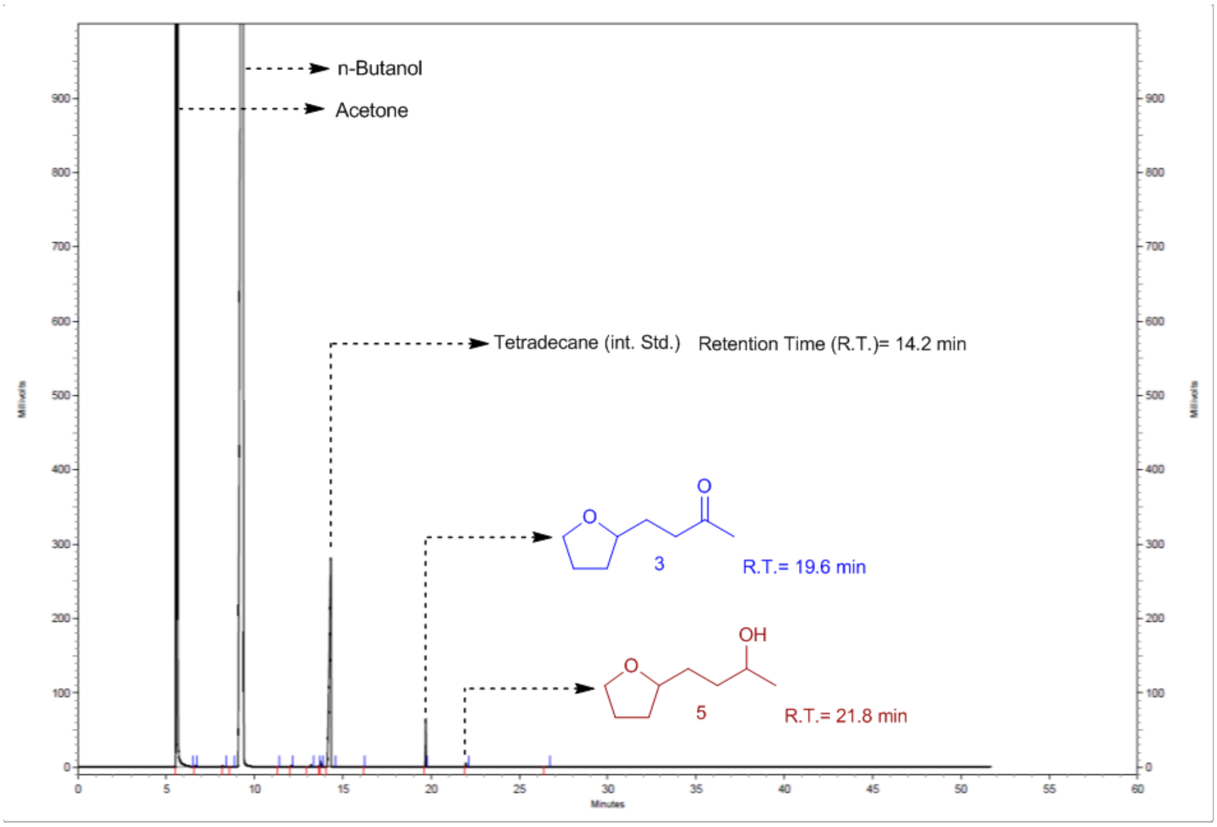

Supplementary Figure 91: Chromatogram corresponding to Supplementary Table 8, Entry 11.

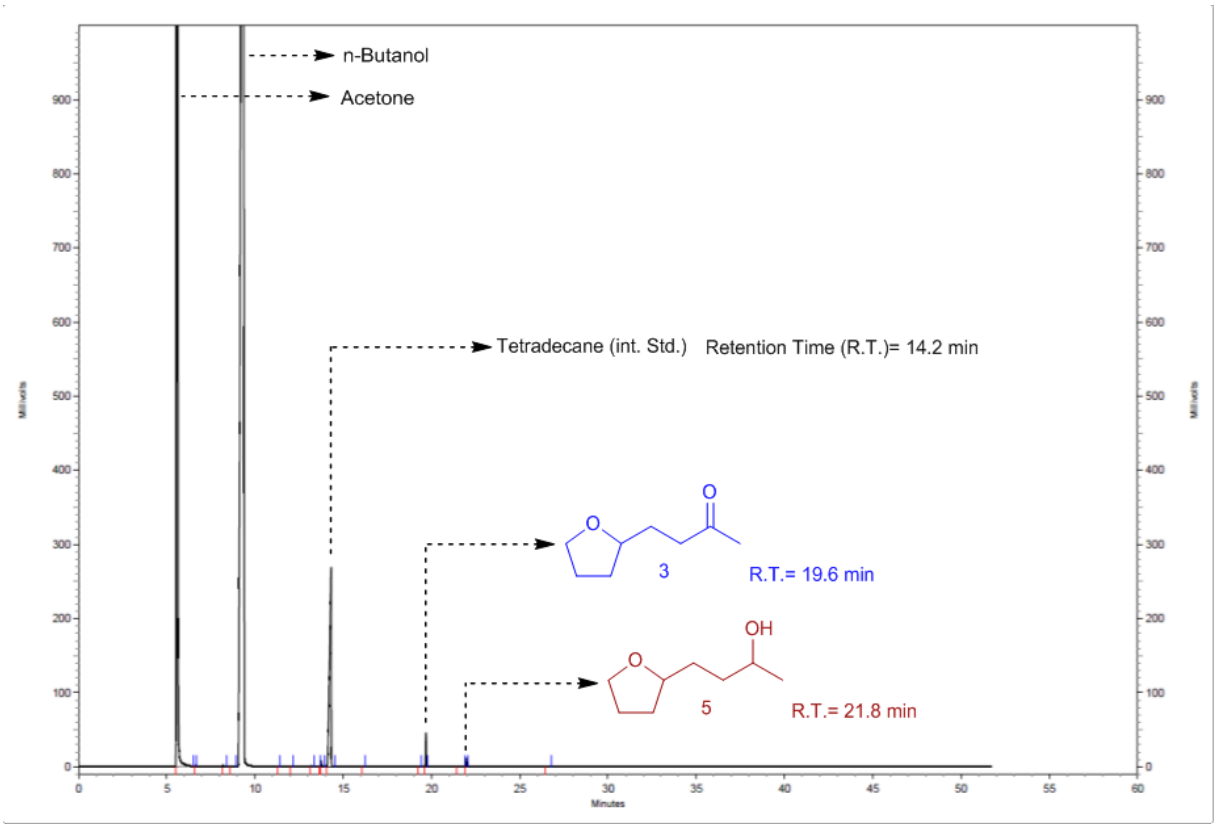

Supplementary Figure 92: Chromatogram corresponding to Supplementary Table 8, Entry 12.

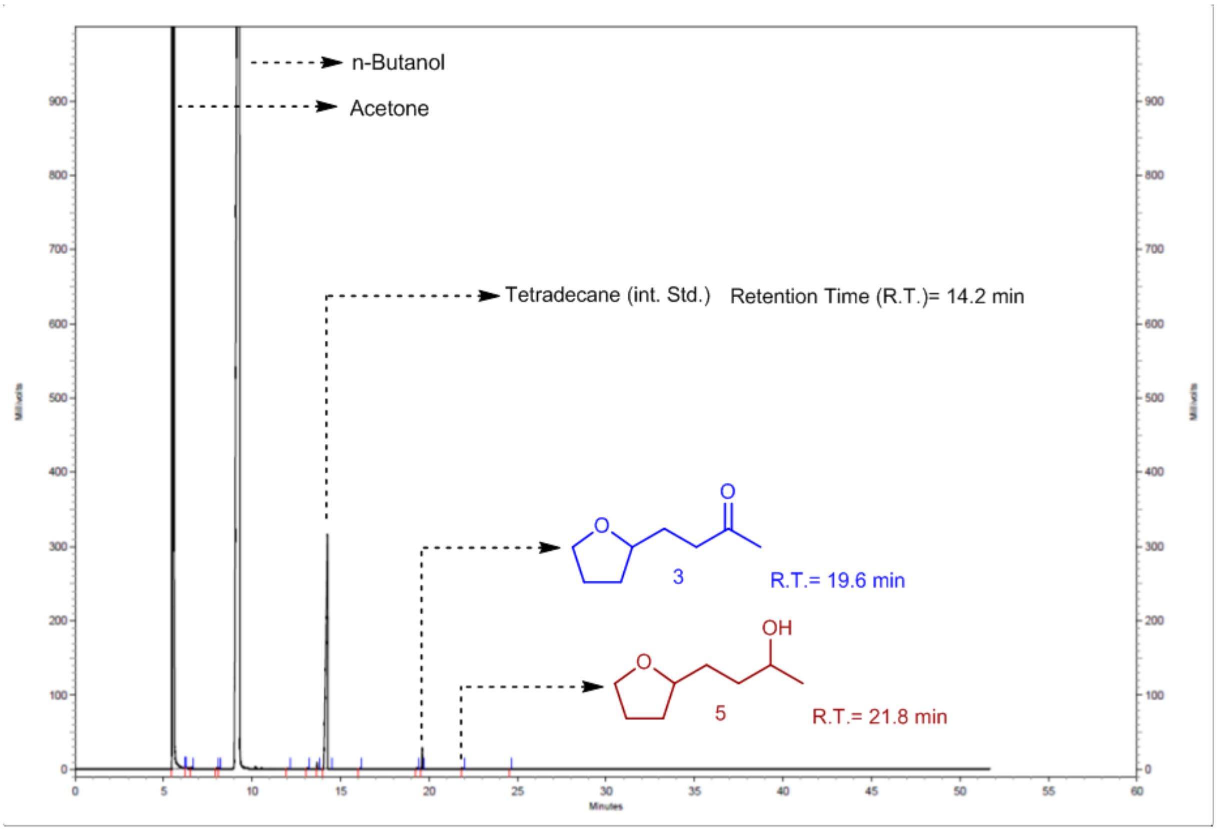

Supplementary Figure 93: Chromatogram corresponding to Supplementary Table 8, Entry 13.

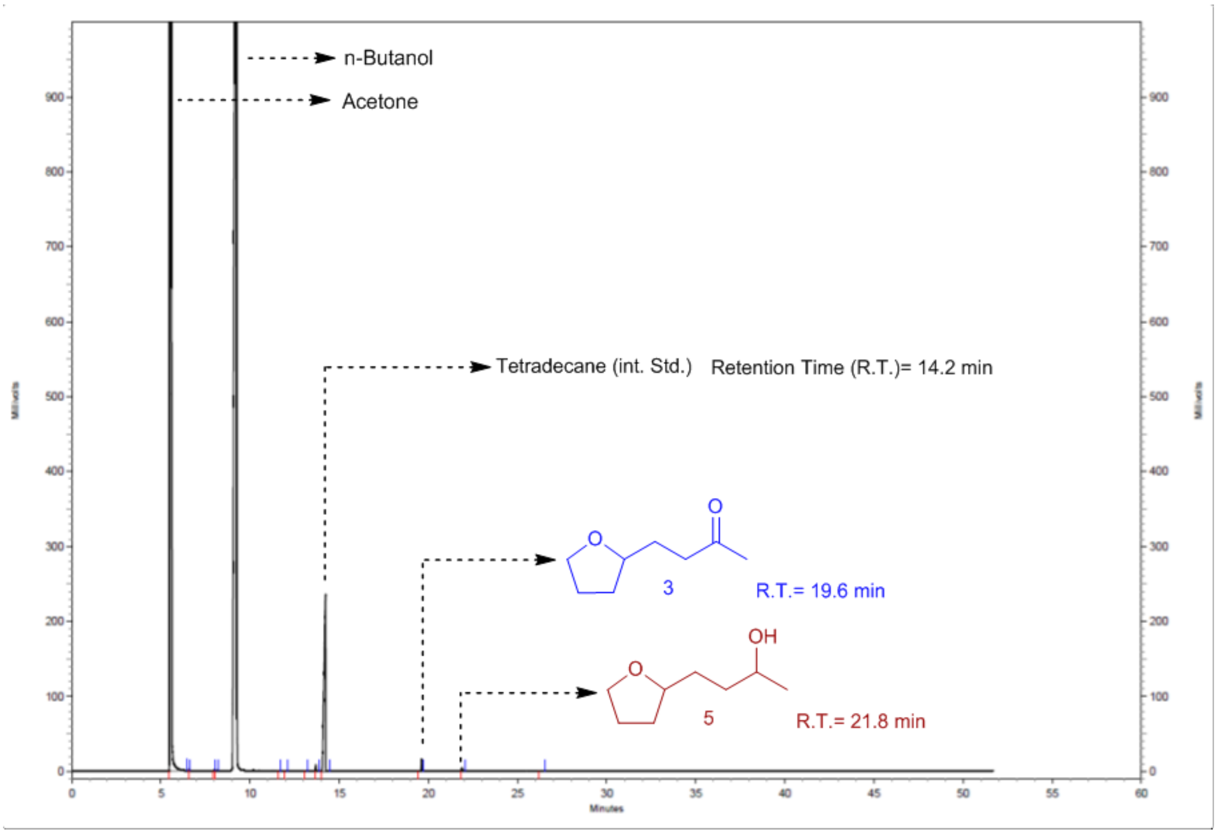

Supplementary Figure 94: Chromatogram corresponding to Supplementary Table 8, Entry 14.

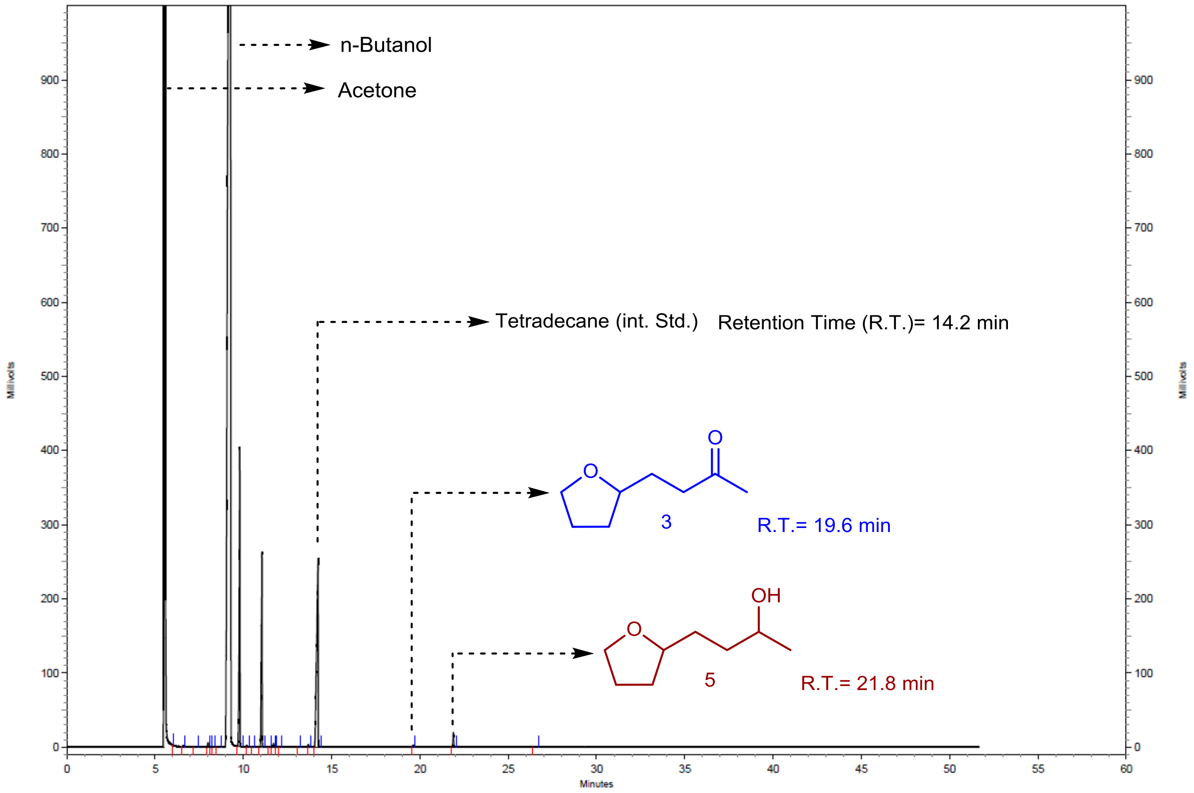

Supplementary Figure 95: Chromatogram corresponding to Supplementary Table 8, Entry 15.

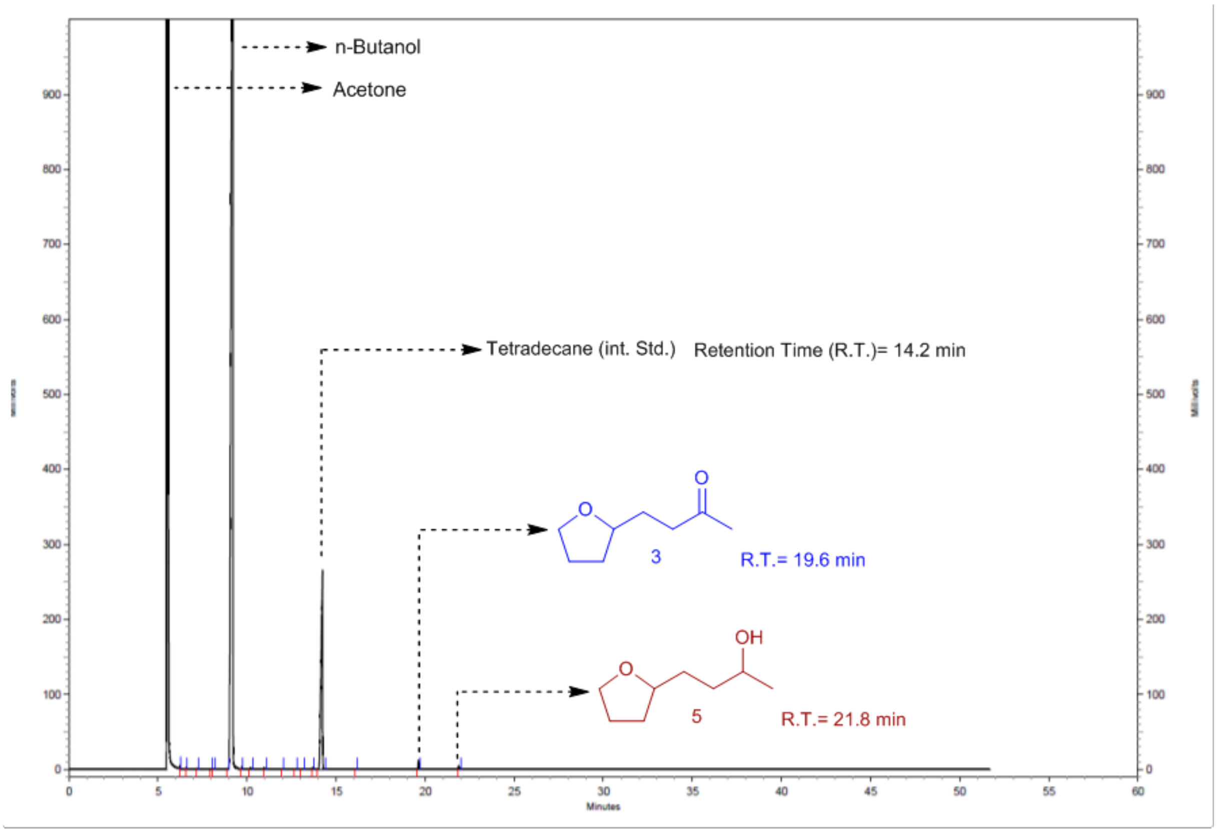

Supplementary Figure 96: Chromatogram corresponding to Supplementary Table 8, Entry 16.

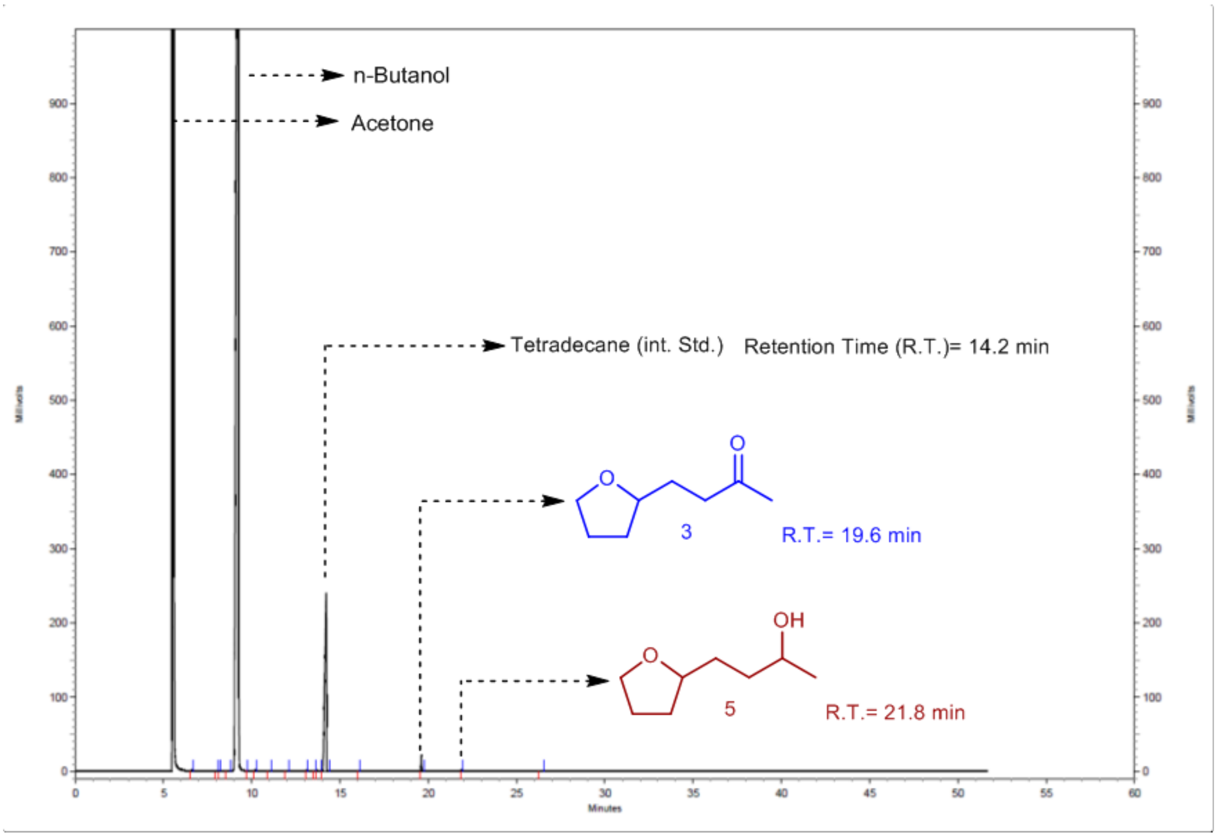

Supplementary Figure 97: Chromatogram corresponding to Supplementary Table 8, Entry 17.

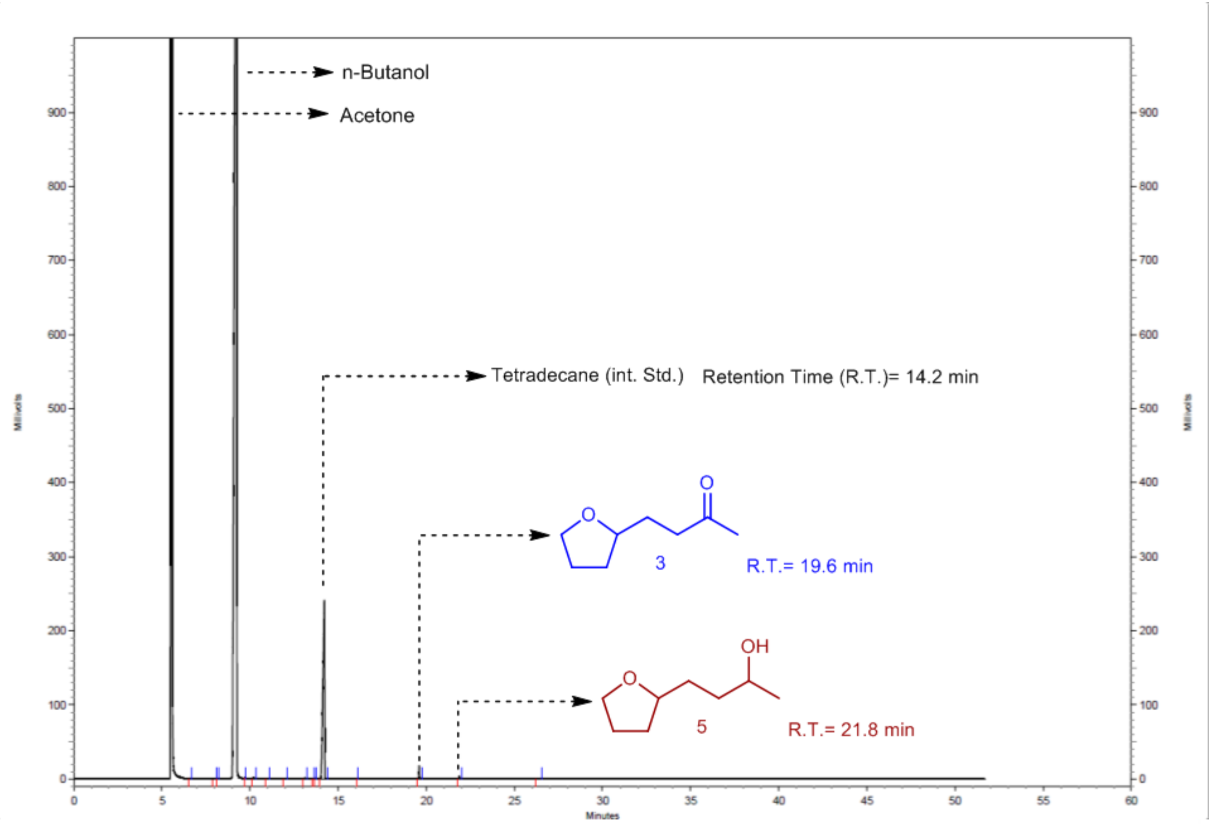

Supplementary Figure 98: Chromatogram corresponding to Supplementary Table 8, Entry 18.

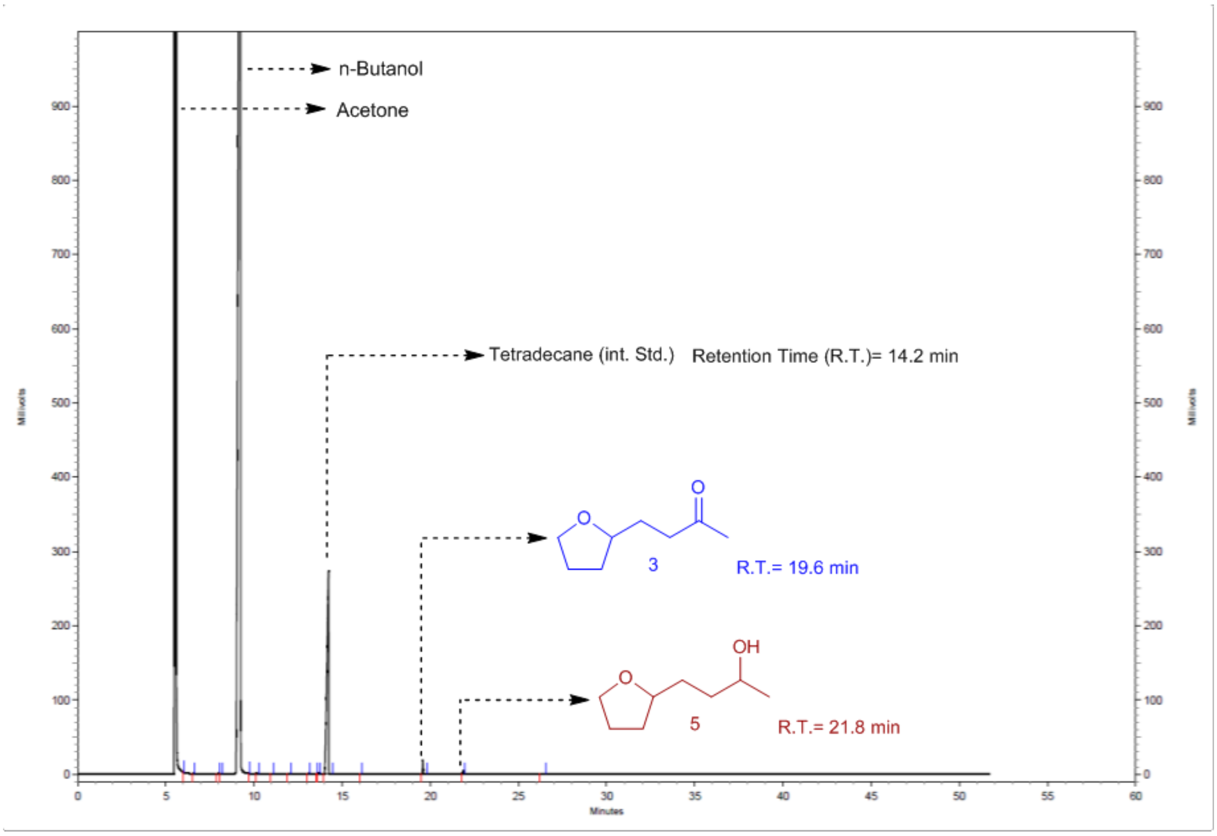

Supplementary Figure 99: Chromatogram corresponding to Supplementary Table 8, Entry 19.

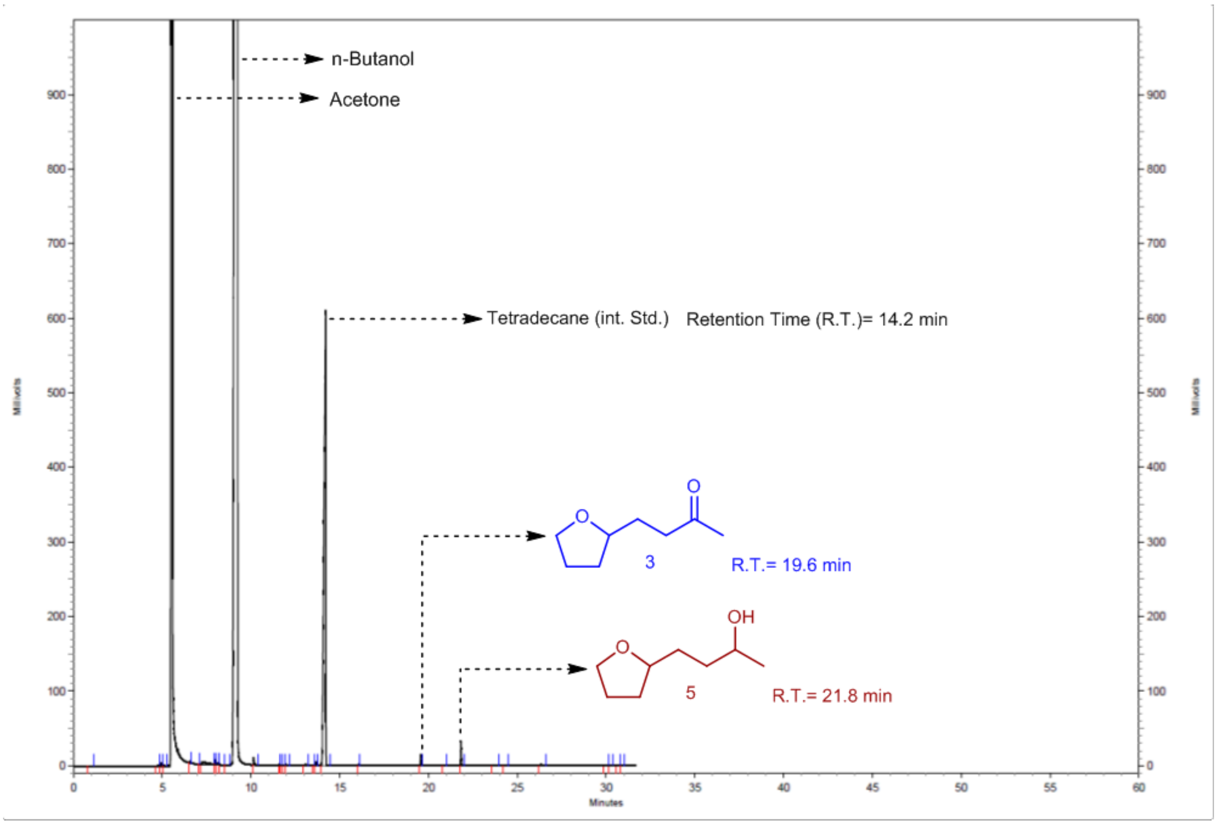

Supplementary Figure 100: Chromatogram corresponding to Supplementary Table 8, Entry 20.

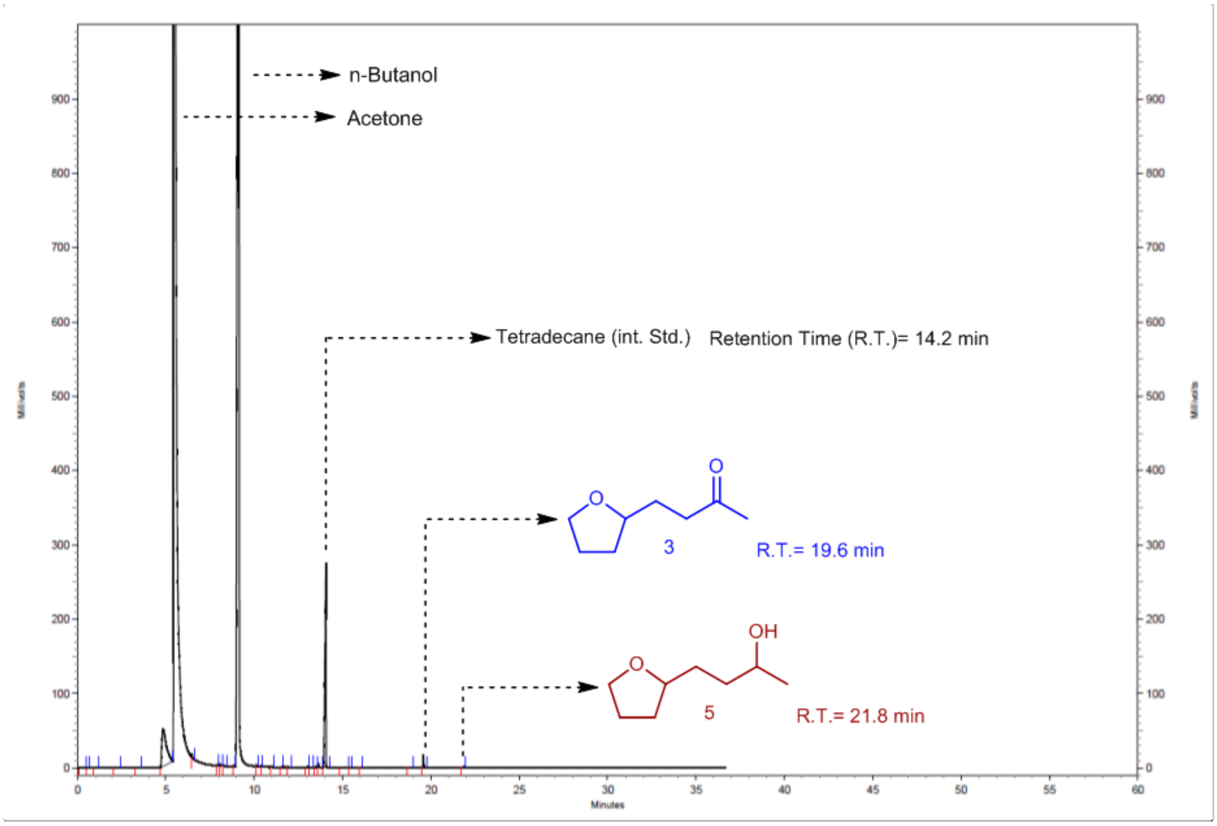

Supplementary Figure 101: Chromatogram corresponding to Supplementary Table 8, Entry 21.

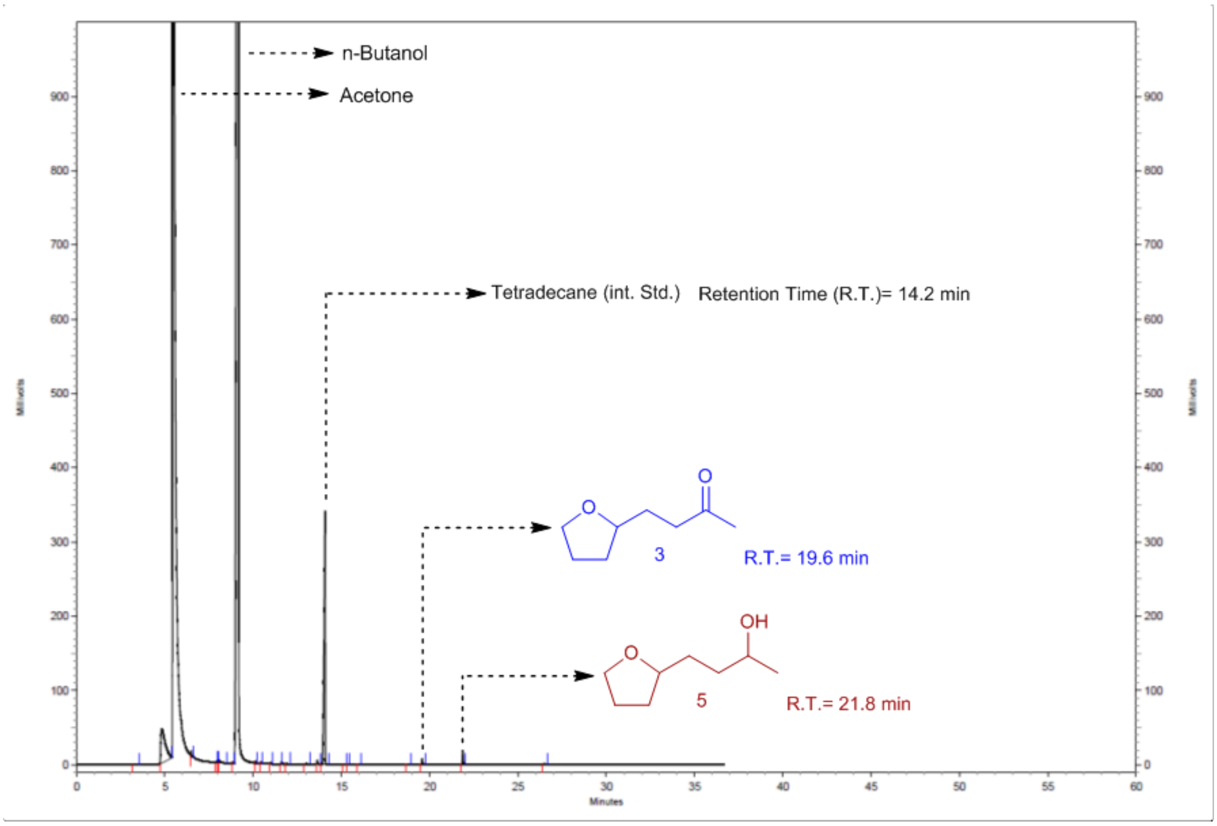

Supplementary Figure 102: Chromatogram corresponding to Supplementary Table 8, Entry 22.

NMR Spectra

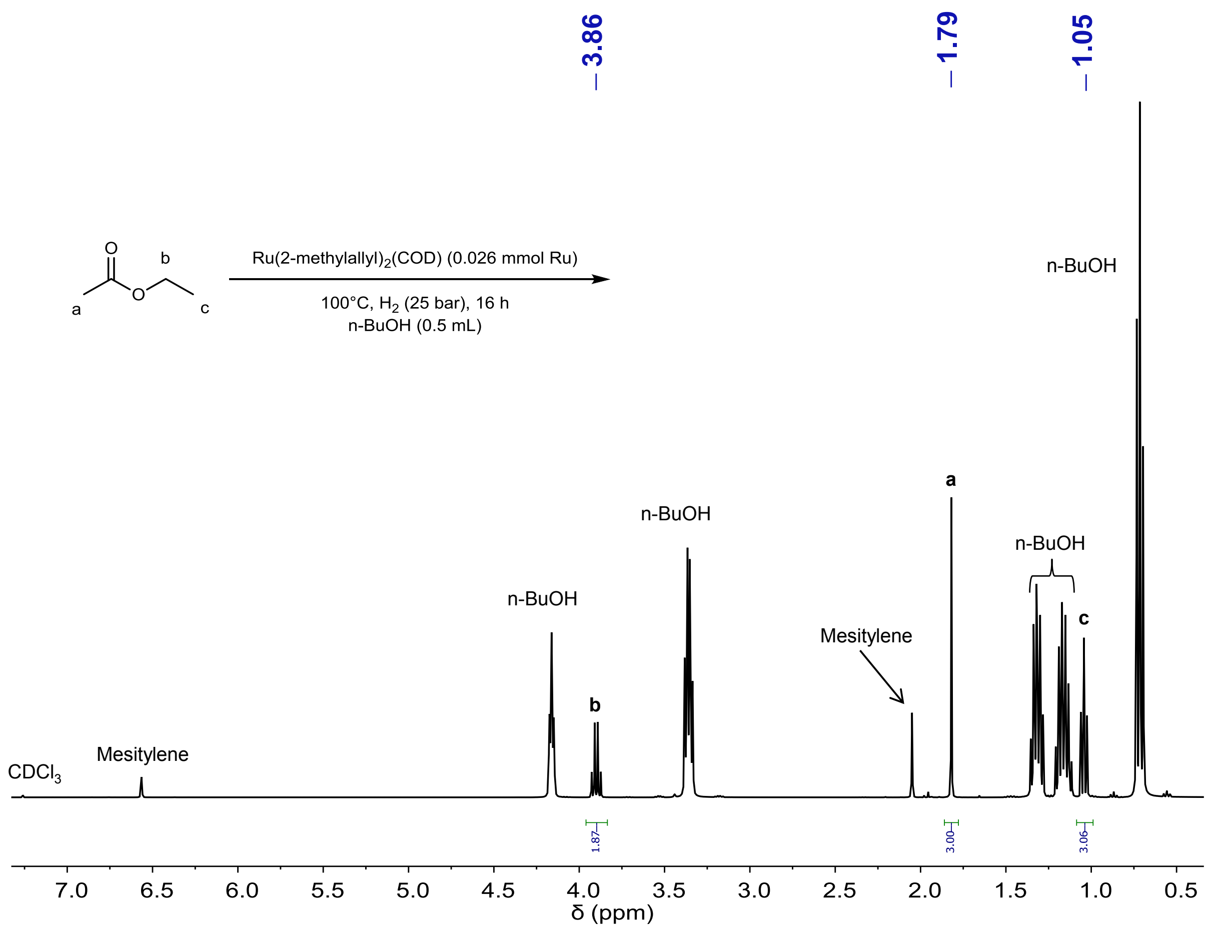

Supplementary Figure 103: ^{1}H NMR spectrum ($CDCl_3$, 400 MHz) corresponding to Supplementary Table 2, Entry 1 *right*.

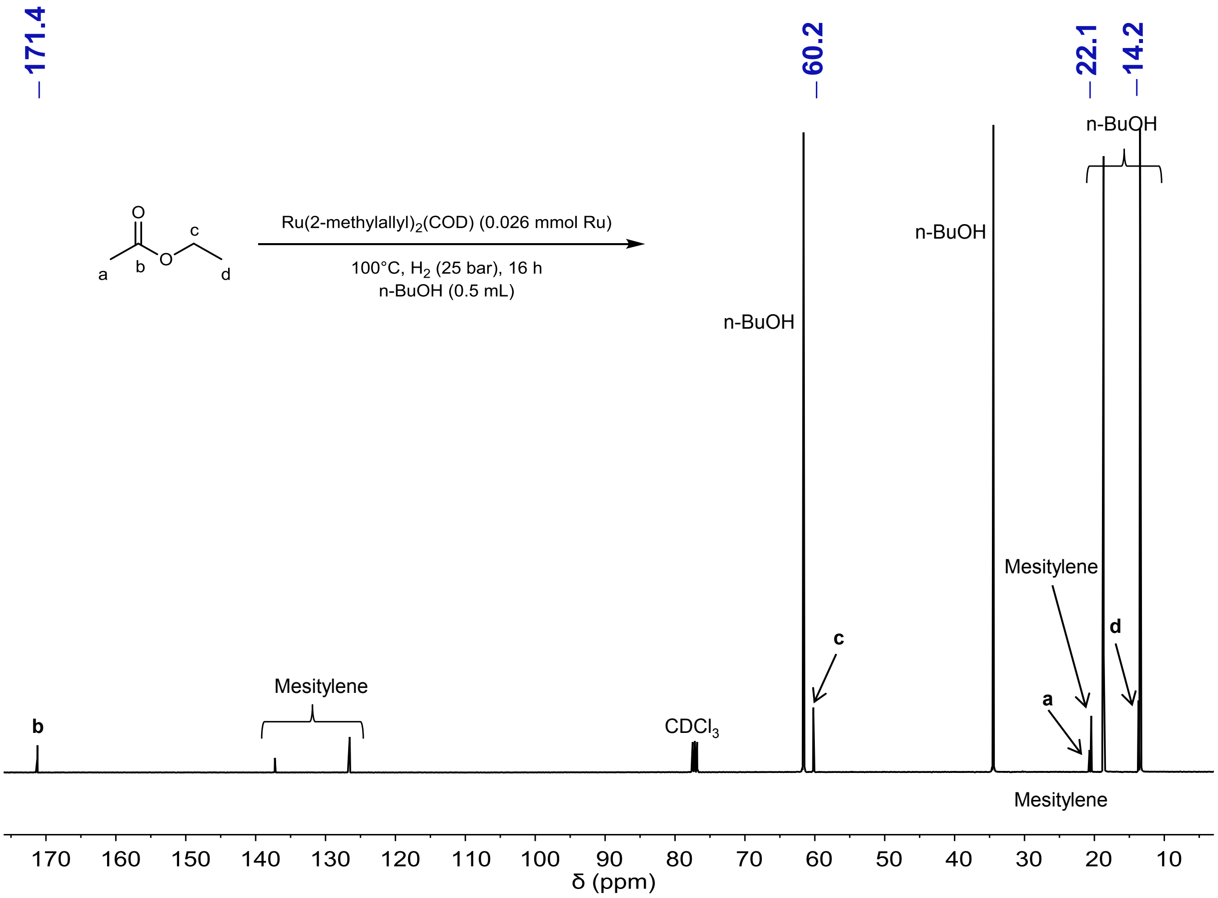

Supplementary Figure 104: ^{13}C NMR ($CDCl_3$, 100 MHz) spectrum corresponding to Supplementary Table 2, Entry 1 *right*.

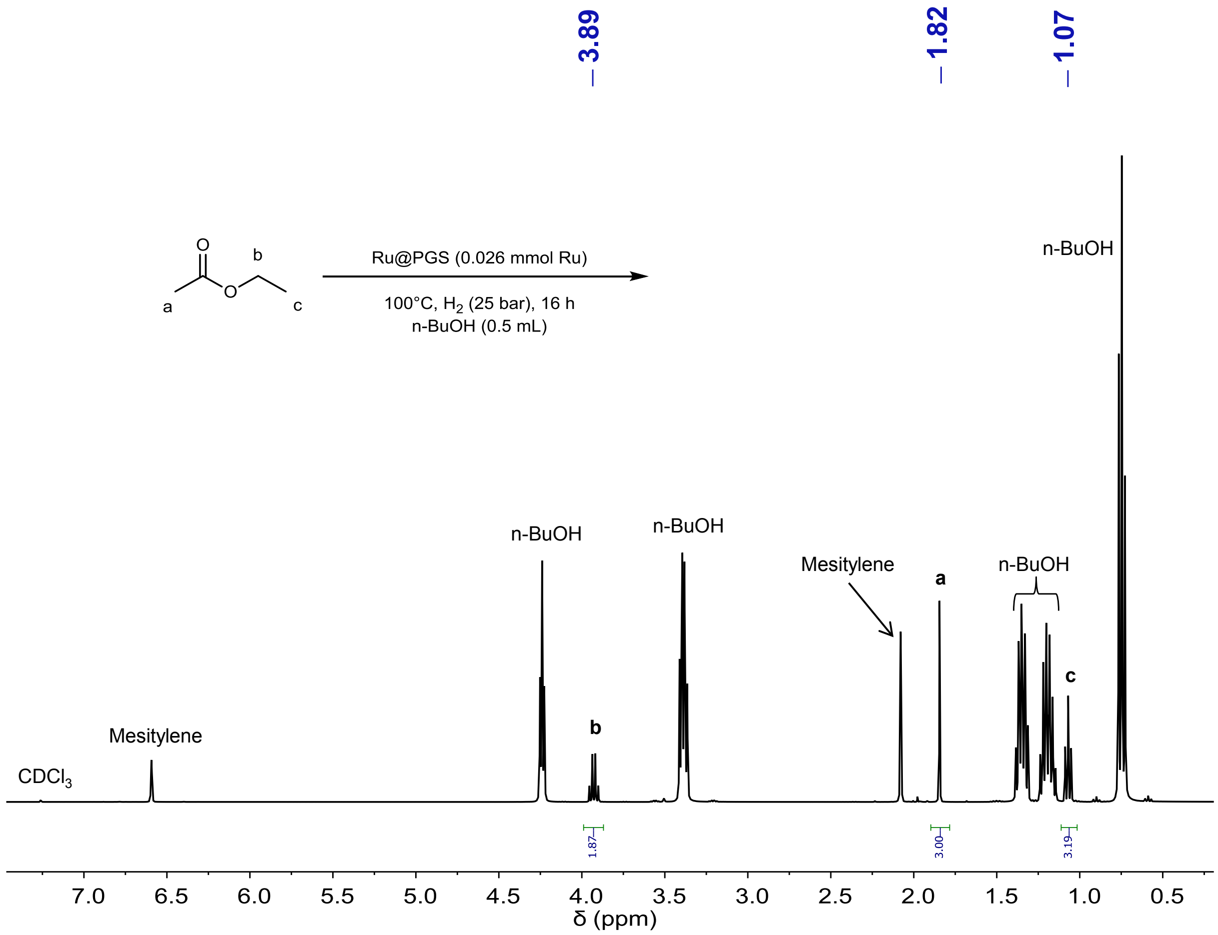

Supplementary Figure 105: ^{1}H NMR ($CDCl_3$, 400 MHz) spectrum corresponding to Supplementary Table 2, Entry 2 *right*.

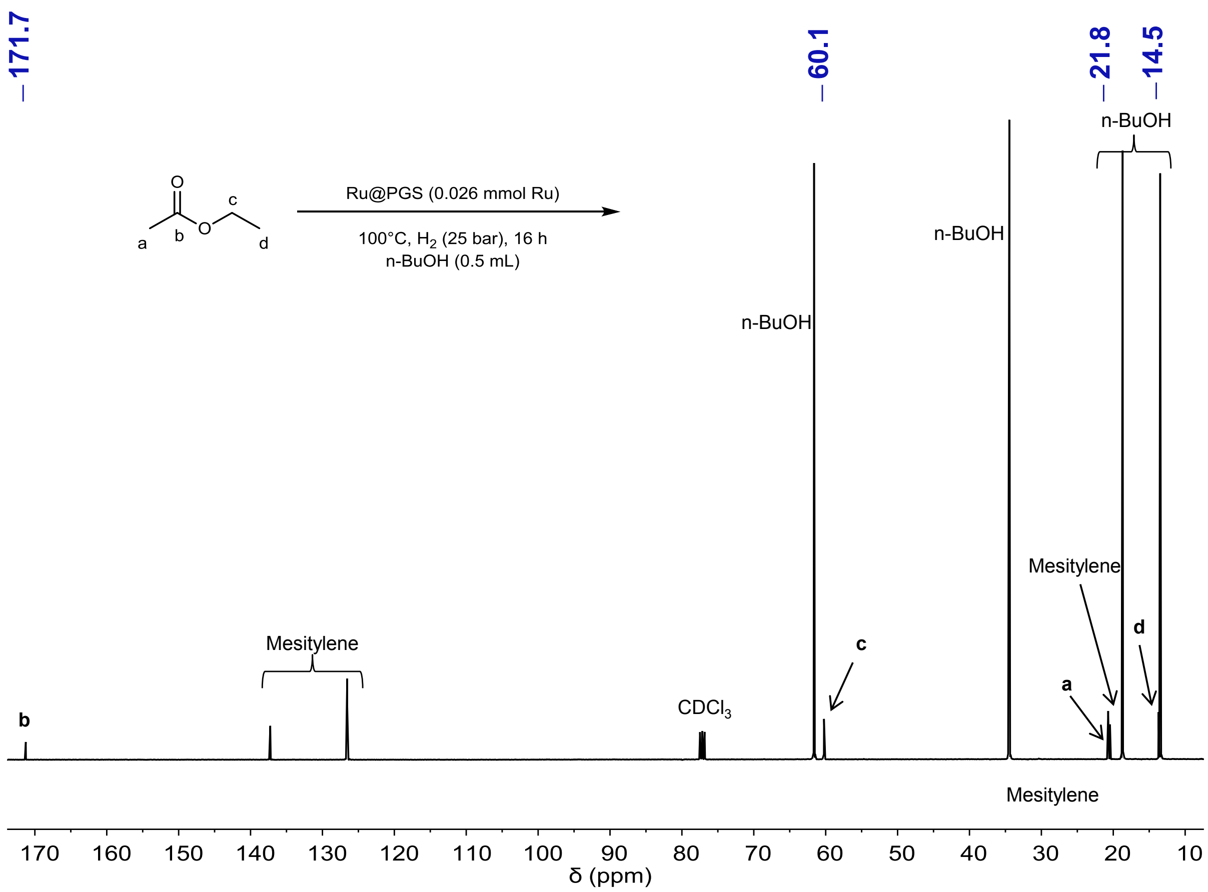

Supplementary Figure 106: ^{13}C NMR spectrum ($CDCl_3$, 100 MHz) corresponding to Supplementary Table 2, Entry 2 *right*.

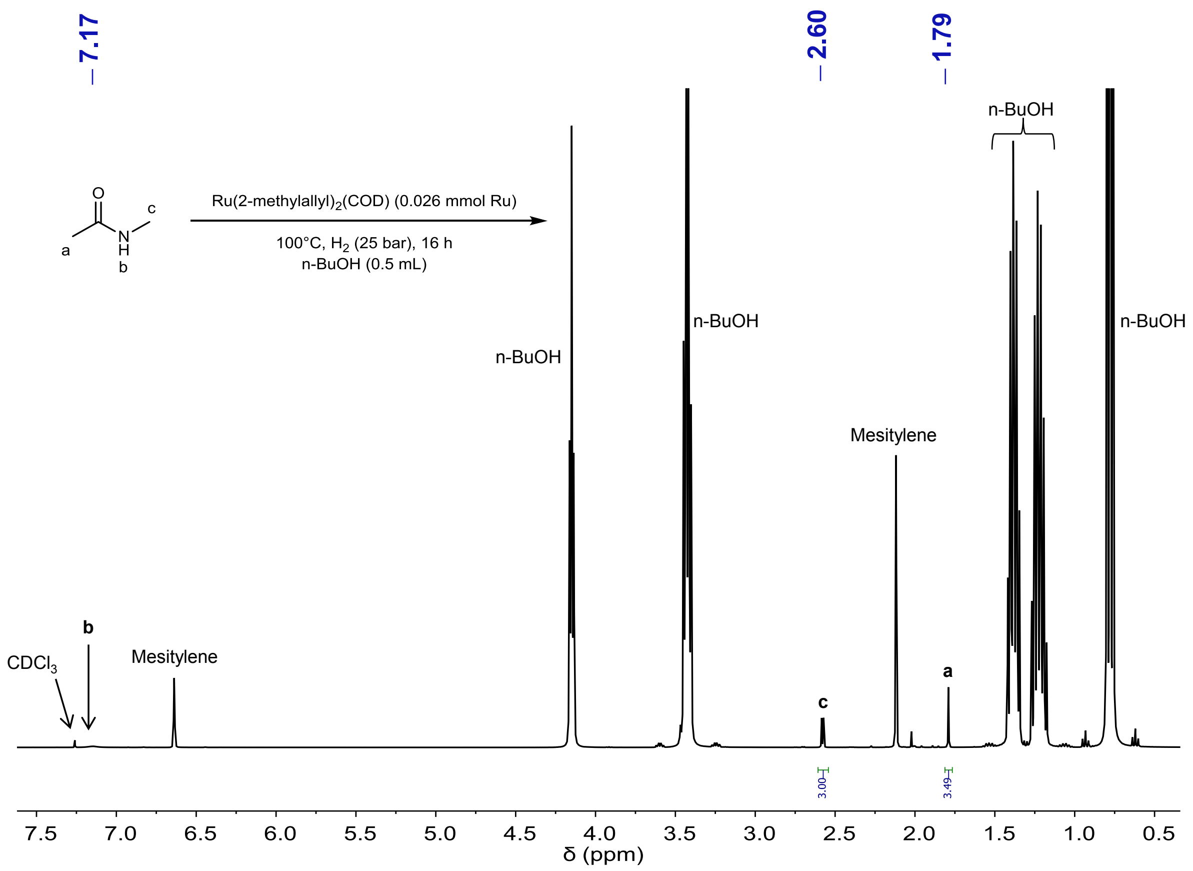

Supplementary Figure 107: ^{1}H NMR spectrum ($CDCl_3$, 400 MHz) corresponding to Supplementary Table 2, Entry 1 *left*.

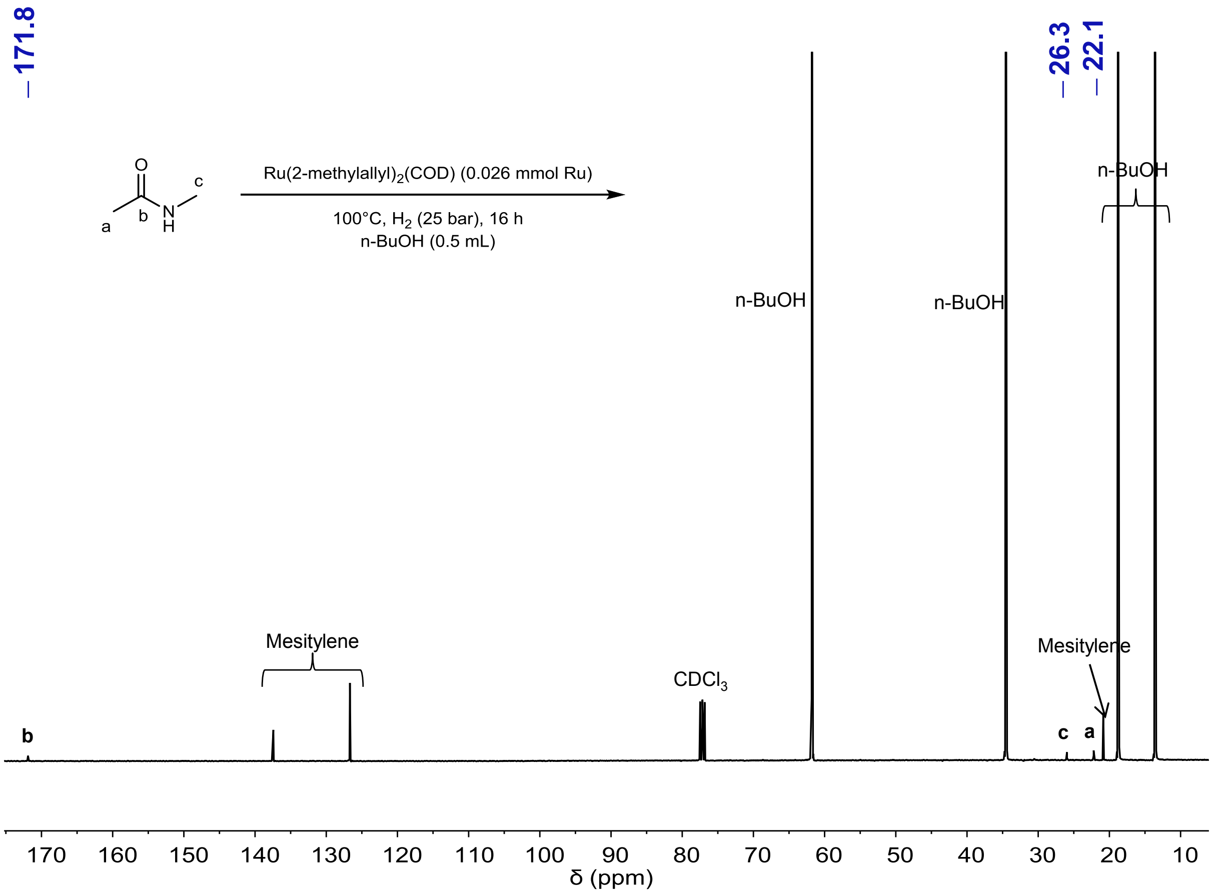

Supplementary Figure 108: ^{13}C NMR spectrum (CDCl$_3$, 100 MHz) corresponding to Supplementary Table 2, Entry 1 *left*.

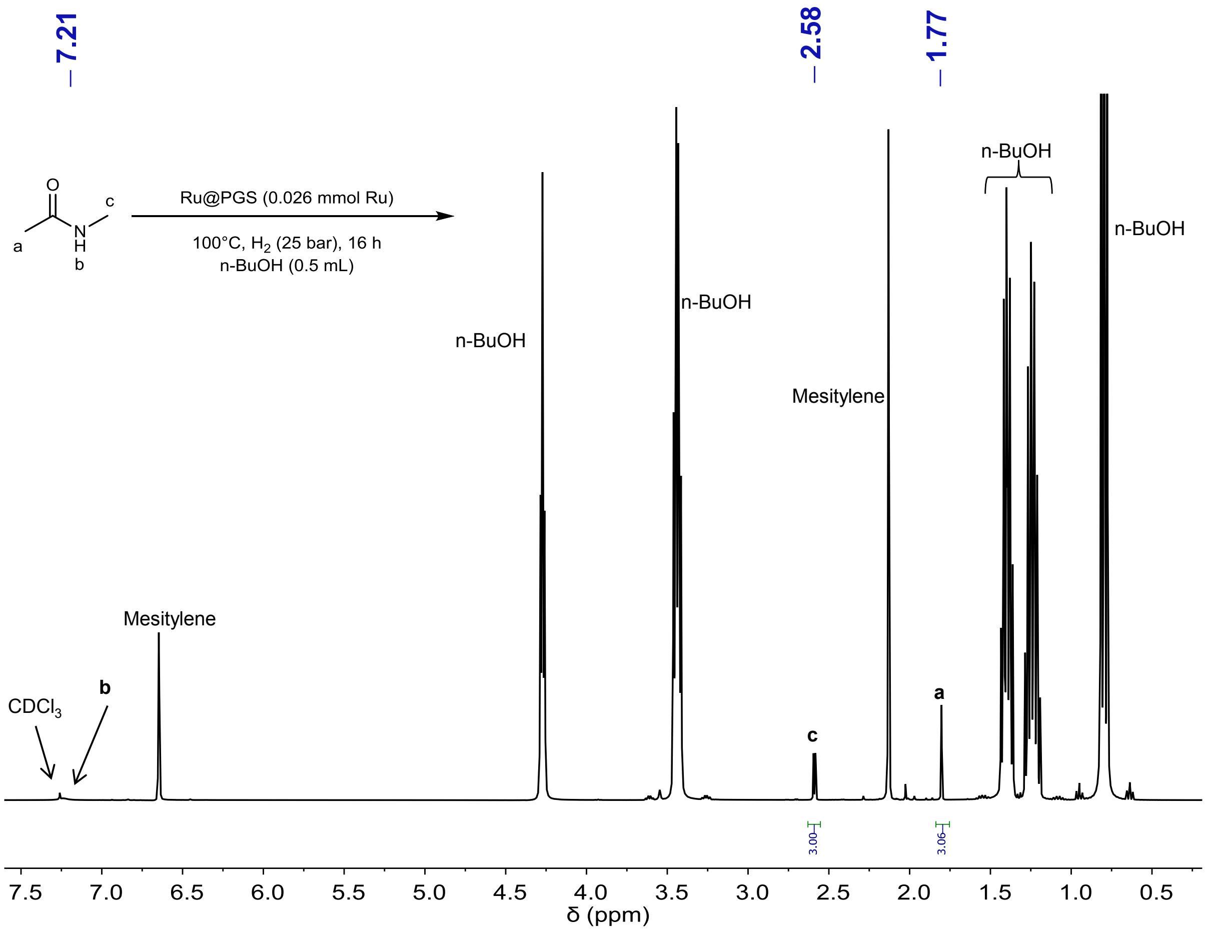

Supplementary Figure 109: ^{1}H NMR spectrum ($CDCl_3$, 400 MHz) corresponding to Supplementary Table 2, Entry 2 *left*.

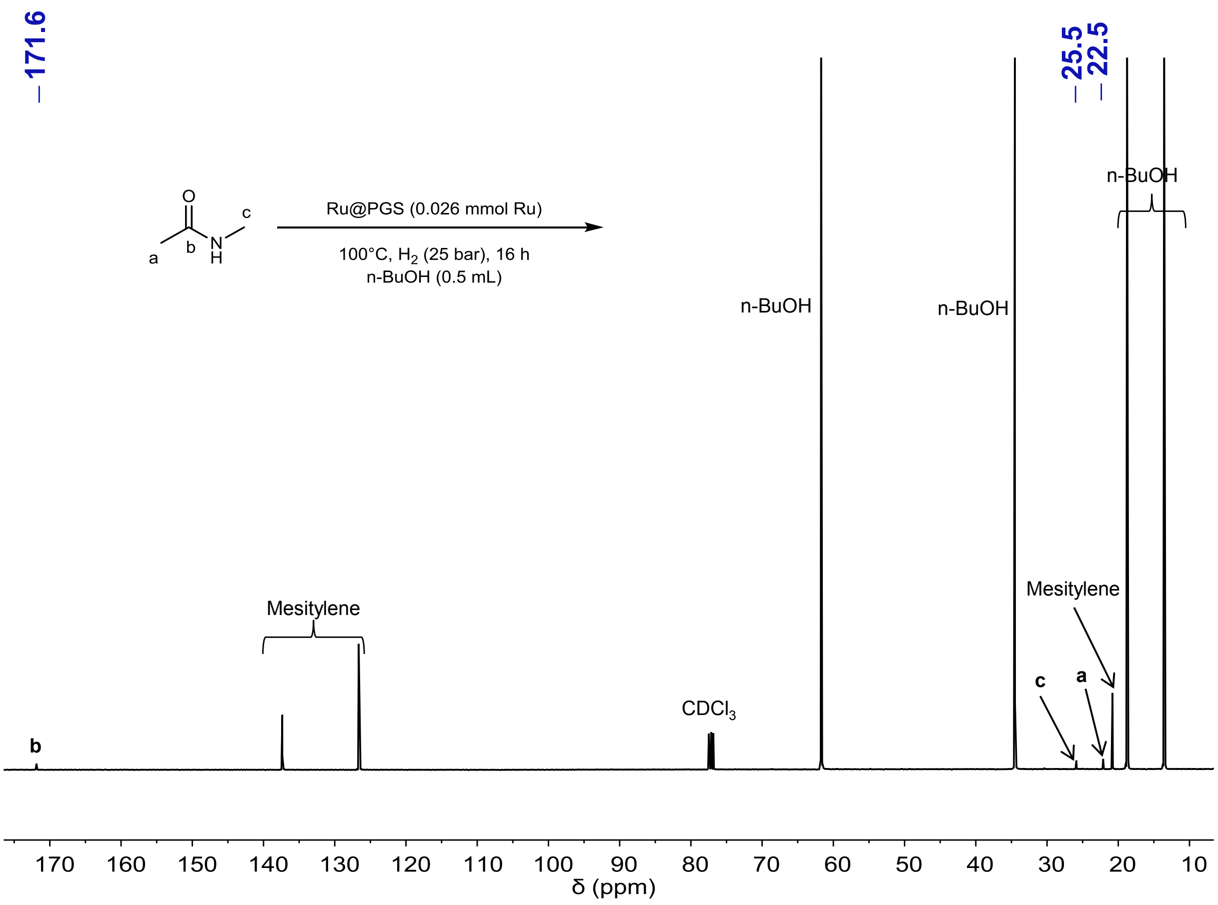

Supplementary Figure 110: ^{13}C NMR spectrum ($CDCl_3$, 100 MHz) corresponding to Supplementary Table 2, Entry 2 *left*.

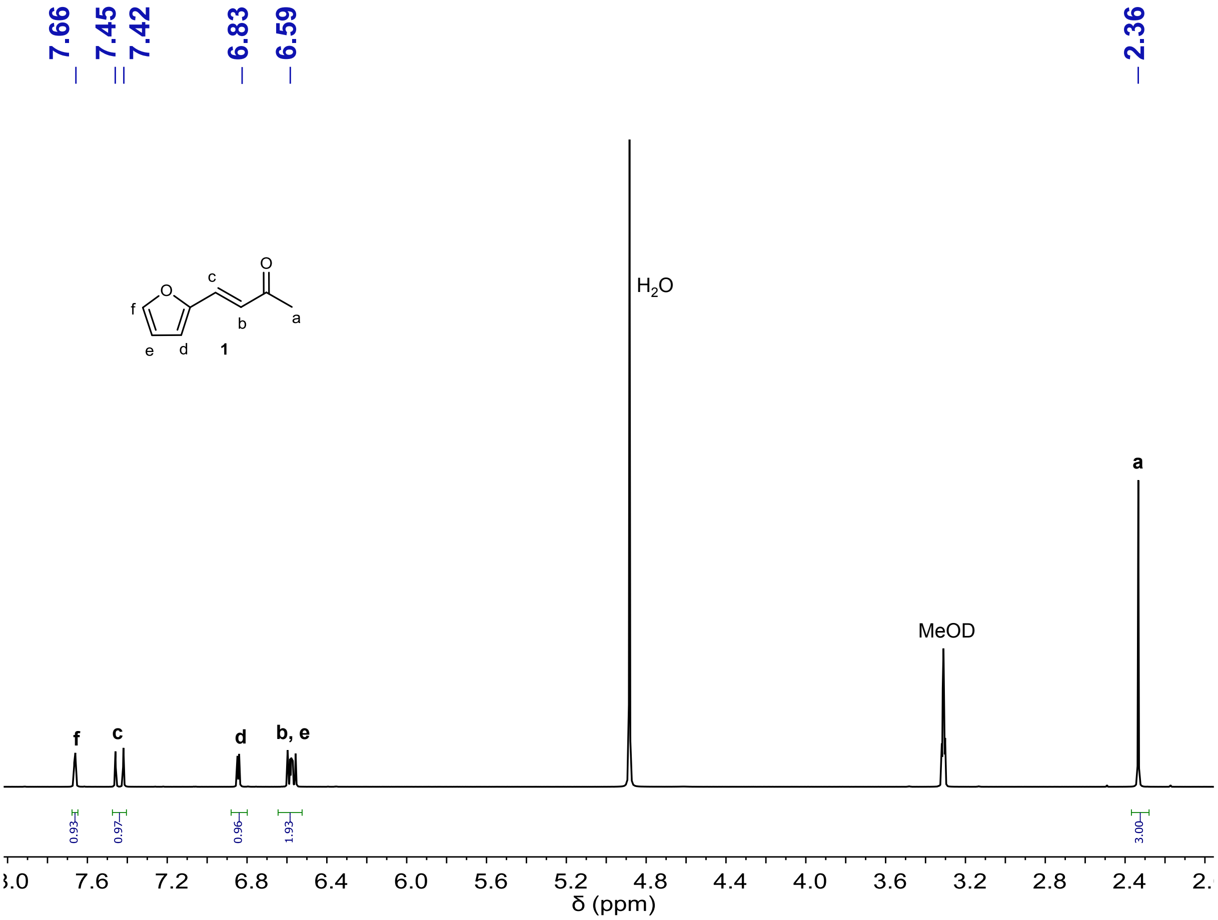

Supplementary Figure 111: ^{1}H NMR (MeOD, 400 MHz) spectrum of furfuralacetone (**1**).

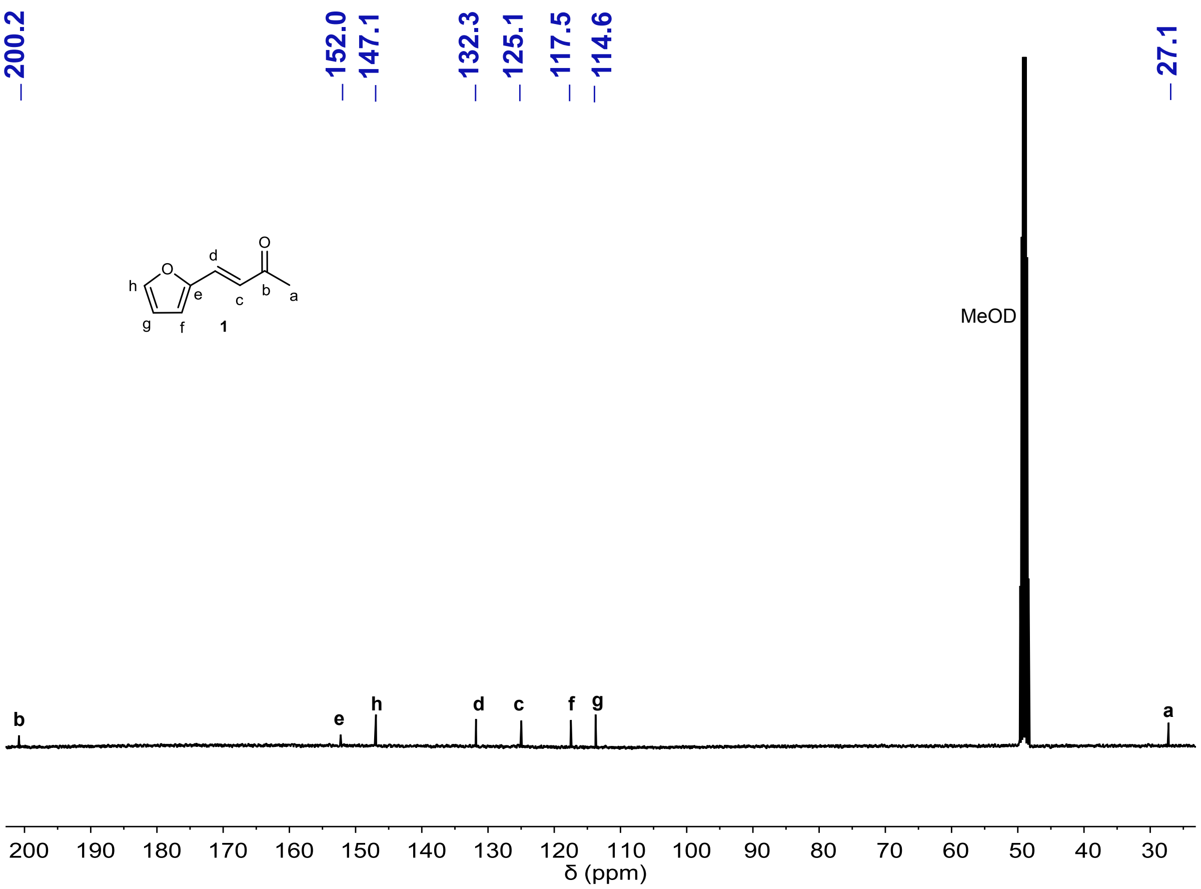

Supplementary Figure 112: ^{13}C NMR (MeOD, 100 MHz) spectrum of furfuralacetone (**1**).

Summary furfuralacetone (**1**):

^{1}H NMR (400 MHz, MeOD): δ 7.66 (s, 1H), 7.45-7.42 (d, J = 12 Hz, 1H), 6.83 (d, J = 5 Hz, 1H), 6.49 (m, 2H), 2.36 (s, 3H); ^{13}C NMR (100 MHz, MeOD): δ 200.2, 152.0, 147.1, 132.3, 125.1, 117.5, 114.6, 27.1.

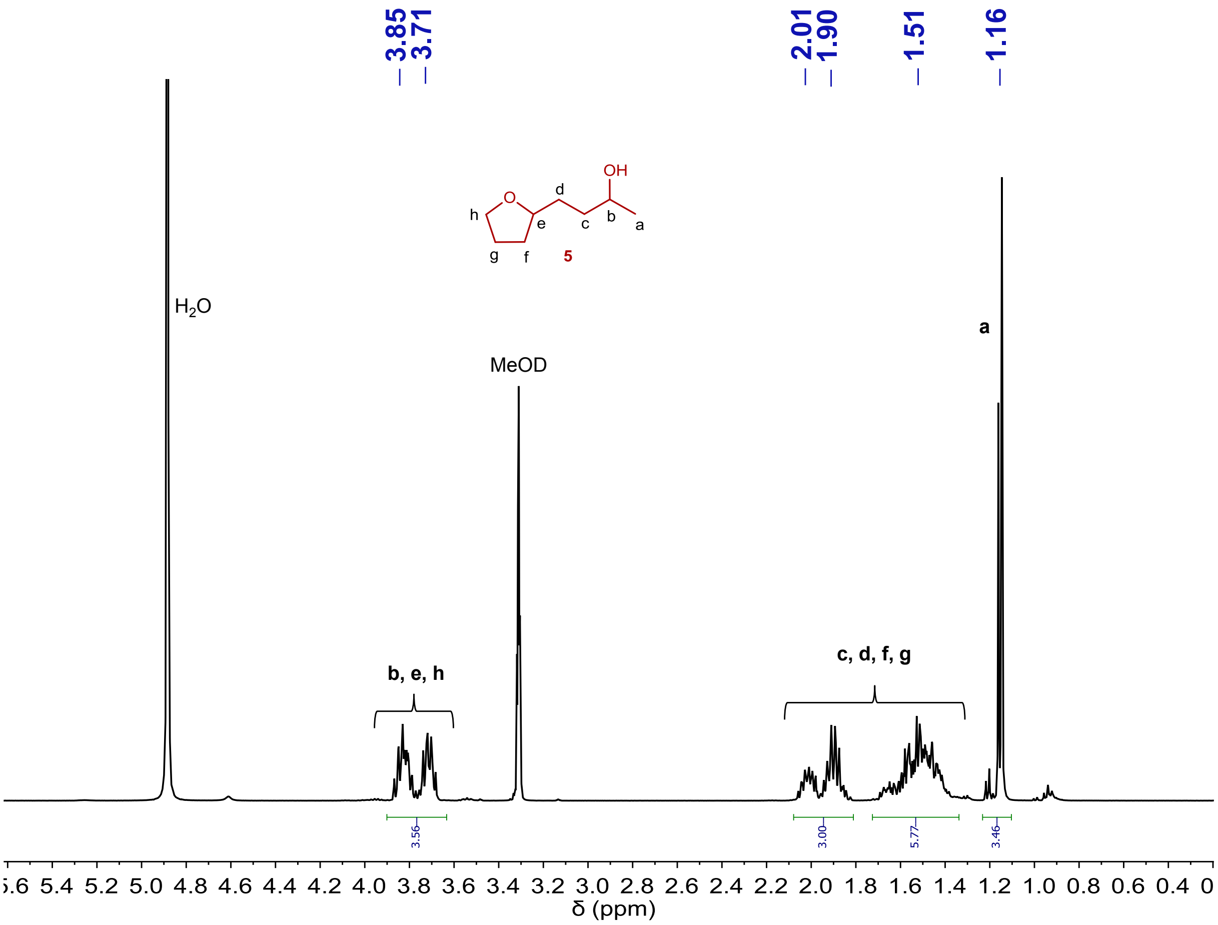

Supplementary Figure 113: Representative ^{1}H NMR (MeOD, 400 MHz) spectrum of 4-(2-tetrahydrofuryl)-butan-2-ol (**5**) obtained after hydrogenation of **1** using Ru@PGS under H_2 (crude mixture).

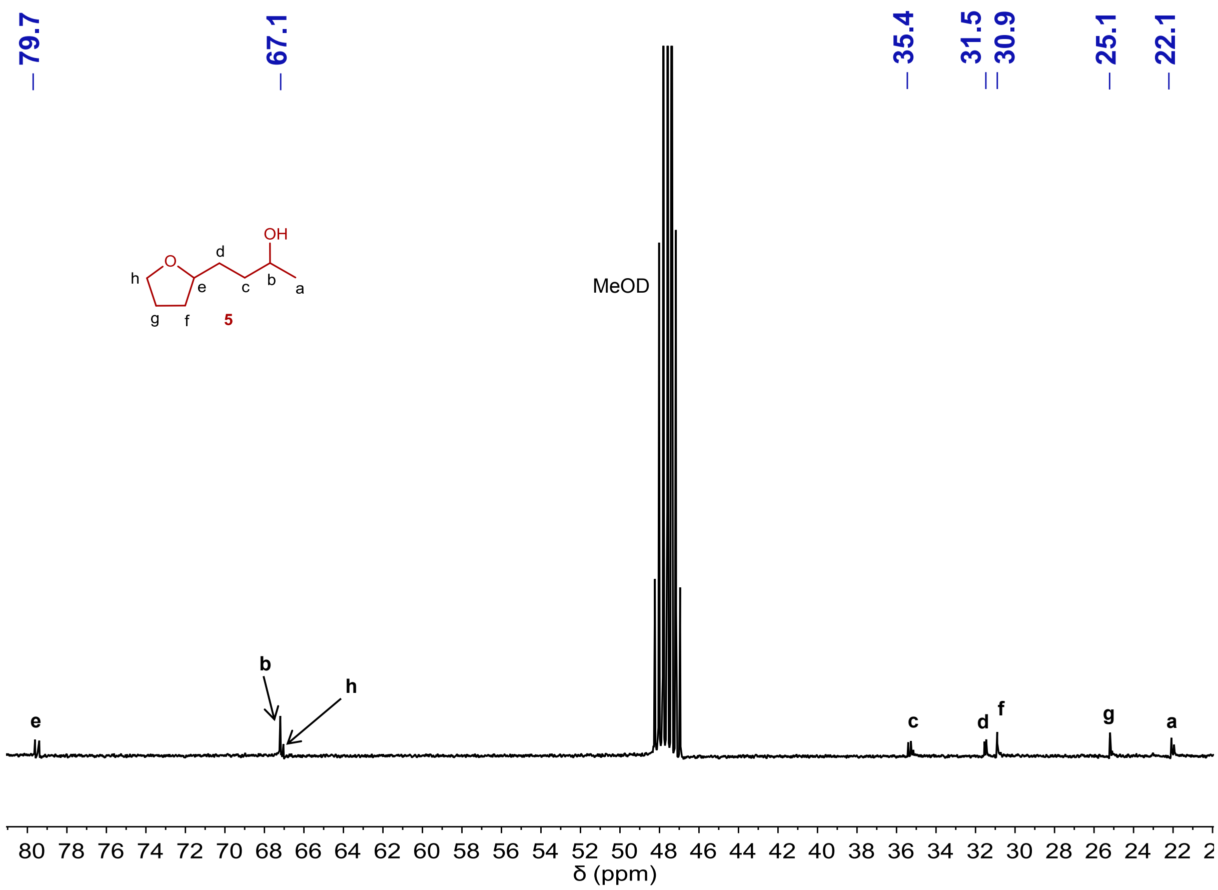

Supplementary Figure 114: Representative ^{13}C NMR (MeOD, 100 MHz) spectrum of 4-(2-tetrahydrofuryl)-butan-2-ol (**5**) obtained after hydrogenation of **1** using Ru@PGS under H_2 (crude mixture).

Summary 4-(2-tetrahydrofuryl)-butan-2-ol (**5**):

^{1}H NMR (400 MHz, MeOD): δ 3.85-3.71 (m, 4H), 2.01-1.51 (m, 8H), 1.16 (d, J = 6.8 Hz, 3H); ^{13}C NMR (100 MHz, MeOD): δ 79.7, 67.1, 66.9, 35.4, 31.5, 30.9, 25.1, 22.1.

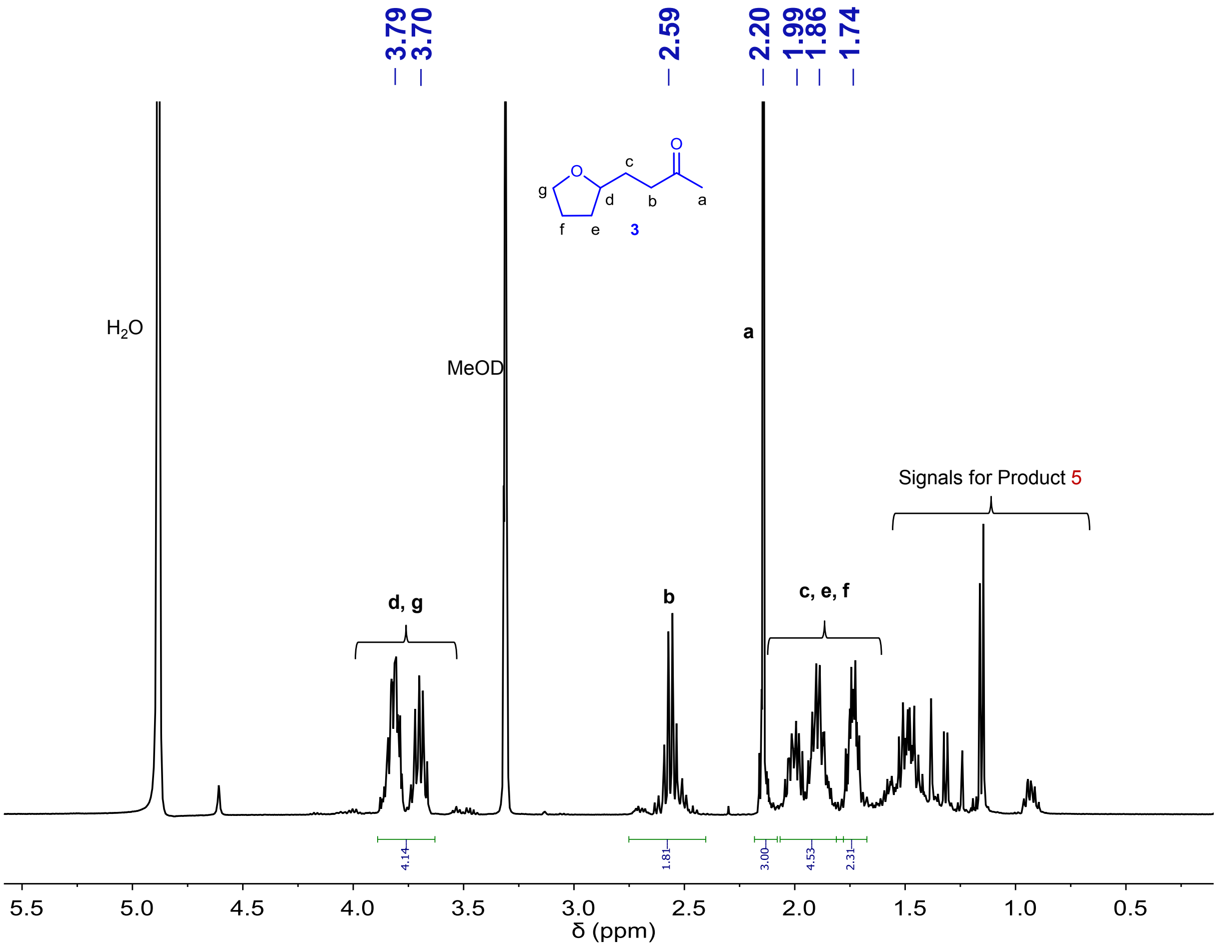

Supplementary Figure 115: Representative ^1H NMR (MeOD, 400 MHz) spectrum of 4-(2-tetrahydrofuryl)-butan-2-one (**3**) obtained after hydrogenation of **1** using Ru@PGS under H_2/CO_2 (crude mixture).

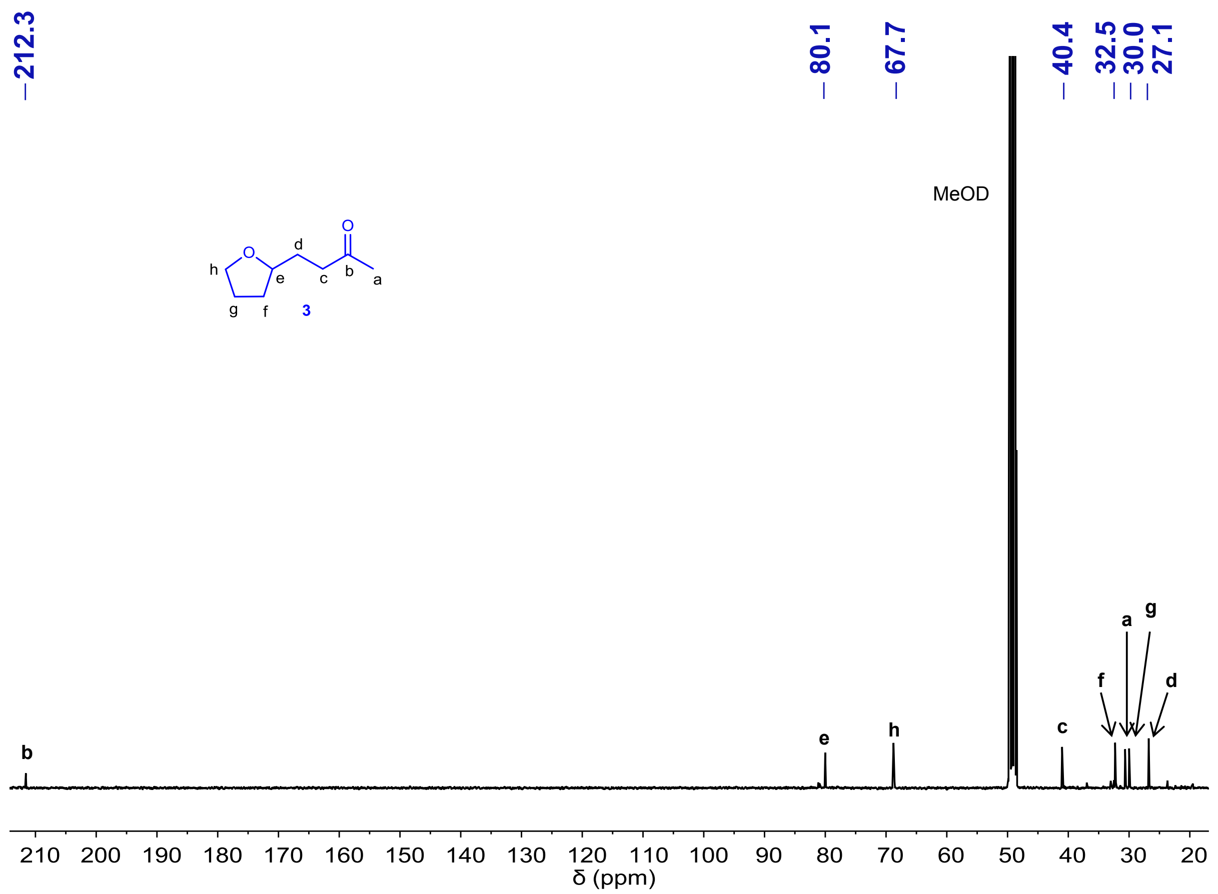

Supplementary Figure 116: Representative ^{13}C NMR (MeOD, 100 MHz) spectrum of 4-(2-tetrahydrofuryl)-butan-2-one (**3**) obtained after hydrogenation of **1** using Ru@PGS under H_2/CO_2 (crude mixture).

Summary 4-(2-tetrahydrofuryl)-butan-2-one (**3**):

^1H NMR (400 MHz, MeOD): δ 3.79-3.70 (m, 4H), 2.59 (m, 2H), 2.20 (s, 3H), 1.99-1.74 (m, 8H); ^{13}C NMR (100 MHz, MeOD): δ 202.3, 80.1, 67.7, 40.4, 32.5, 30.2, 30.0, 27.1.

References

1. Boniface, K. J. CO_2-responsive surfaces, PhD thesis (Queen's University, Canada, 2018).

2. Luska, K. L. *et al.* Bifunctional nanoparticle–SILP catalysts (NPs@SILP) for the selective deoxygenation of biomass substrates. *Chem. Sci.* **5,** 4895-4905 (2014).

3. Boniface, K. J. *et al.* CO_2-switchable drying agents. *Green Chem.* **18,** 208-213 (2016).
